# Thermally Stable Terbium(II) and Dysprosium(II) Bis-amidinate Complexes

**DOI:** 10.1021/jacs.3c07978

**Published:** 2023-11-24

**Authors:** Peng-Bo Jin, Qian-Cheng Luo, Gemma K. Gransbury, Iñigo J. Vitorica-Yrezabal, Tomáš Hajdu, Ilya Strashnov, Eric J. L. McInnes, Richard E. P. Winpenny, Nicholas F. Chilton, David P. Mills, Yan-Zhen Zheng

**Affiliations:** † Frontier Institute of Science and Technology (FIST), State Key Laboratory of Electrical Insulation and Power Equipment, MOE Key Laboratory for Nonequilibrium Synthesis of Condensed Matter, Xi’an Key Laboratory of Electronic Devices and Materials Chemistry and School of Chemistry, Xi’an Jiaotong University, 99 Yanxiang Road, Xi’an, Shaanxi 710054, P. R. China; ‡ Department of Chemistry, The University of Manchester, Oxford Road, Manchester M13 9PL, U.K.; § Photon Science Institute, The University of Manchester, Oxford Road, Manchester M13 9PL, U.K.

## Abstract

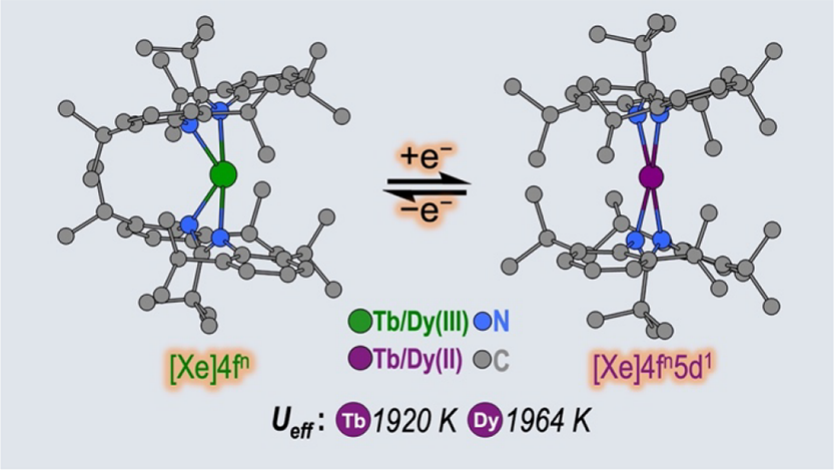

The thermostable four-coordinate divalent lanthanide (Ln) bis-amidinate complexes [Ln(Piso)_2_] (Ln = Tb, Dy; Piso = {(NDipp)_2_C^t^Bu}, Dipp = C_6_H_3_^i^Pr_2_-2,6) were prepared by the reduction of parent five-coordinate Ln(III) precursors [Ln(Piso)_2_I] (Ln = Tb, Dy) with KC_8_; halide abstraction of [Ln(Piso)_2_I] with [H(SiEt_3_)_2_][B(C_6_F_5_)] gave the respective Ln(III) complexes [Ln(Piso)_2_][B(C_6_F_5_)]. All complexes were characterized by X-ray diffraction, ICP-MS, elemental analysis, SQUID magnetometry, UV–vis-NIR, ATR-IR, NMR, and EPR spectroscopy and *ab initio* CASSCF-SO calculations. These data consistently show that [Ln(Piso)_2_] formally exhibit Ln(II) centers with 4f^*n*^5d_*z*2_^1^ (Ln = Tb, *n* = 8; Dy, *n* = 9) valence electron configurations. We show that simple assignments of the f–d coupling to either *L*–*S* or *J*–*s* schemes are an oversimplification, especially in the presence of significant crystal field splitting. The coordination geometry of [Ln(Piso)_2_] is intermediate between square planar and tetrahedral. Projecting from the quaternary carbon atoms of the CN_2_ ligand backbones shows near-linear C···Ln···C arrangements. This results in strong axial ligand fields to give effective energy barriers to magnetic reversal of 1920(91) K for the Tb(II) analogue and 1964(48) K for Dy(II), the highest values observed for mononuclear Ln(II) single-molecule magnets, eclipsing 1738 K for [Tb(C_5_^i^Pr_5_)_2_]. We tentatively attribute the fast zero-field magnetic relaxation for these complexes at low temperatures to transverse fields, resulting in considerable mixing of *m*_*J*_ states.

## Introduction

Beyond the commonly observed traditional Ln(II) ions Sm(II), Eu(II), and Yb(II), complexes containing other Ln(II) ions are relatively rare because of their high Ln(III)/Ln(II) reduction potentials making them difficult to access and often resulting in thermally unstable complexes that can react with common organic solvents.^1−3^ However, over the last 25 years, Ln(II) complexes have been isolated for all Ln except radioactive Pm, and the ligand field has been shown to be key to dictating reduction potentials, thermal sensitivity, and whether 4f^*n*+1^ or 4f^*n*^5d^1^ valence electron configurations are formally adopted.^4−18^ Ln(II) complexes have already shown record-high magnetic susceptibilities,^19^ interesting single molecule magnet (SMM) behavior,^15,20−23^ and potential as spin qubits.^24,25^ The lower charge of Ln(II) ions allows charge balance to be achieved with fewer ligands relative to their Ln(III) counterparts; this is conducive to forming low-coordinate complexes, leading potentially to linear coordination geometries and strongly axial ligand fields.^26^ This has recently been exploited in the synthesis of a series of “linear” Ln(II) metallocenes [Ln(C_5_^*i*^Pr_5_)_2_], which show relatively high thermal stabilities and formal 4f^*n*^5d^1^ configurations, common for most nontraditional Ln(II) ions.^18,20^ There remains a need to study further examples of Ln(II) complexes with other ligands to achieve a greater understanding of their electronic structure.

Lanthanide complexes have provided the best-performing SMMs to date^27−29^ due to their inherently high magnetic anisotropy and unquenched orbital moments arising from predominantly ionic Ln bonding.^1^ To achieve high energy barriers to magnetic reversal (*U*_eff_) in Tb and Dy SMMs, axial ligand fields are required to stabilize the most magnetic *m*_J_ states while simultaneously destabilizing the least magnetic states.^30^ Prior to 2017, large *U*_eff_ values were achieved for axial Dy(III) SMMs, but these did not provide high temperatures for retention of magnetization (here we use *T*_100_, which is the temperature at which the magnetization relaxation time is equal to 100 s) as other relaxation processes such as Raman relaxation and quantum tunneling of magnetization (QTM) are efficient even when the overbarrier process is slow.^27−29^ In the last 6 years, highly axial Dy and Tb complexes containing substituted cyclopentadienyl ligands (Cp^R^) and closely related charge-dense aromatic ligands have raised the temperature at which magnetization is retained to 72 K, and magnetic hysteresis (*T*_H_) has been seen up to 80 K;^18,20,22,31−39^ ligand rigidity has been proposed as a route to suppress Raman relaxation mechanisms.^31,40−43^ Again, this indicates that studies of different ligand sets are needed to test whether the rigidity is important.

Most investigations of Ln SMMs to date have focused on complexes containing Ln ions in the most common +3 oxidation state.^27−29^ The Kramers ion Tb(II) complex [Tb(C_5_^*i*^Pr_5_)_2_] has *U*_eff_ = 1738 K, *T*_H_ = 55 K, and *T*_100_ = 52 K.^20^ To the best of our knowledge, this is the only Ln(II) SMM to date with a measured energy barrier, and all these parameters are currently record-high values for any mononuclear Tb SMM;^1−3^ a *U*_eff_ value for the non-Kramers ion Dy(II) complex [Dy(C_5_^*i*^Pr_5_)_2_] could not be determined.^20^ Conversely, the mixed-valent Ln complexes [Ln_2_(C_5_^*i*^Pr_5_)_2_(μ-I)_3_] (Ln = Tb, Dy) exhibit one-electron Ln–Ln σ bonds, leading to large coercive fields and favorable SMM properties for both Tb and Dy; the Dy analog currently holds record values for *U*_eff_ (2347 K), *T*_H_ (80 K), and *T*_100_ (72 K) among all SMMs.^22^

Amidinates ({(NR_2_)_2_CR}) and guanidinates ({(NR_2_)_2_CNR_2_}) have been used extensively as supporting ligands in Ln chemistry due to their ease of preparation and facile tunability to control metal coordination spheres;^44^ these ligands have recently delivered Dy(III) SMMs, namely, [Dy{(N^i^Pr)_2_CN(SiMe_3_)_2_}_2_(μ-Cl)_2_Li(THF)_2_],^45^ [Dy{(N^i^Pr)_2_CN(SiMe_3_)_2_}_2_(μ-Cl)]_2_,^45^ [Dy{(N^i^Pr)_2_CMe}(C_5_Me_5_)(μ-Cl)}]_2_,^46,47^ [Dy{(NDipp)_2_CMe}(C_5_Me_5_)(Cl)(μ-Cl)Li(THF)_3_],^47^ and [Dy{(N^i^Pr)_2_CN(SiMe_3_)_2_}_2_{(μ-Ph)BPh_3_}].^48^ We noted that the Ln(II) bis-guanidinate complexes [Ln(Priso)_2_] and [Ln(Giso)_2_] (Ln = Sm, Eu, Yb; Priso = {(NDipp)_2_CN^i^Pr_2_}, Giso = {(NDipp)_2_CN^i^Cy_2_}; Dipp = C_6_H_3_^i^Pr_2_-2,6) are only four-coordinate,^49,50^ and reasoned that similar Tb(II/III) and Dy(II/III) complexes could exhibit interesting magnetic properties. As the related Ln(III) amidinate complex [Ce(Piso)_2_Cl] (Piso = {(NDipp)_2_C^t^Bu}) was shown to be directly prepared from CeCl_3_,^51^ we opted to use Piso as a supporting ligand.

Here we report the synthesis of four-coordinate Ln(II) and Ln(III) bis-amidinate complexes for Ln = Tb and Dy. These complexes were characterized by X-ray diffraction, ICP-MS, elemental analysis, SQUID magnetometry, UV–vis-NIR, ATR-IR, NMR, and EPR spectroscopy. Complete active space self-consistent field spin–orbit (CASSCF-SO) calculations were performed to determine the electronic structures and to rationalize the experimental data. We find that the Ln(II) Piso complexes are remarkably thermostable and formally adopt 4f^*n*^5d^1^ valence electron configurations with significant 5d-6s mixing. We project model Hamiltonians from our *ab initio* calculations to highlight the difficulty in assigning compounds to *L*–*S* or *J*–*s* coupling schemes, especially on the basis of room-temperature magnetic moments. The strong axial crystal fields (CFs) in these complexes lead to them showing record *U*_eff_ values for Ln(II) SMMs, but we observe rapid Raman relaxation and QTM, giving low *T*_H_ and *T*_100_ values. Although dramatic improvements in SMM behavior are not seen, these studies provide significant new insights into the electronic structures of exotic Ln(II) compounds.

## Results and Discussion

### Synthesis

**Scheme 1:**
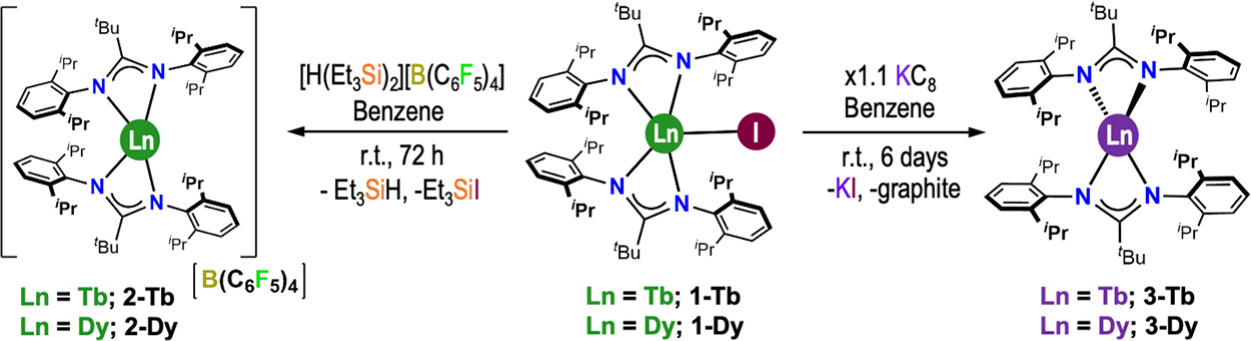
Synthesis of **2-Ln** and **3-Ln** from **1-Ln** (Ln = Tb, Dy)

The heteroleptic Ln(III) bis-amidinate iodide complexes [Ln(Piso)_2_I] (**1-Ln**, Ln = Tb, Dy) were prepared by the salt metathesis reactions of parent LnI_3_^52^ with two equivalents of KPiso^53^ in toluene at reflux by modifying literature protocols^51^ (Scheme S1). The reactions of **1-Ln** with the silylium reagent [H(SiEt_3_)_2_][B(C_6_F_5_)]^54,55^ in benzene gave the homoleptic separated ion-pair Ln(III) bis-amidinate complexes [Ln(Piso)_2_][B(C_6_F_5_)_4_] (**2-Ln**, Ln = Tb, Dy) (Scheme 1) by adapting procedures that were previously employed for the synthesis of [Dy(Cp^ttt^)_2_][B(C_6_F_5_)_4_] (Cp^ttt^ = C_5_H_2_^t^Bu_3_-1,2,4).^31−33^ The neutral homoleptic Ln(II) bis-amidinate complexes [Ln(Piso)_2_] (**3-Ln**, Ln = Tb, Dy) were synthesized by the reduction of **1-Ln** with a small excess of KC_8_ in benzene (Scheme 1); orange crystals of **3-Ln** were isolated in *ca*. 20% yields following workup and recrystallization from dark green pentane solutions at −35 °C (see Experimental Section: Synthesis for a more detailed description).

### Bulk Characterization

Samples of **1-Ln**, **2-Ln**, and **3-Ln** were assessed by a variety of analytical techniques to give complementary data on the bulk purities of samples. Powder X-ray diffraction (PXRD) patterns obtained showed excellent agreement with models, with refinement analyses generally indicating high crystalline phase purities; the sample of **1-Dy** analyzed was found to contain two polymorphs (see below), but quantitative analysis allowed their respective ratio to be determined precisely (Figures S64–S69). Elemental analysis results were generally in good agreement with expected values, although lower-than-expected carbon contents were consistently determined; this is attributed to incomplete combustion due to the formation of carbides under the experimental conditions employed.^56^ The lanthanide compositions determined by ICP-MS generally showed excellent agreement with expected values, with <6% differences between expected and found values for **2-Ln** and **3-Ln** (Table S44); these differences were slightly larger for **1-Ln**, but the total uncertainty for these measurements is ∼15–20% due to possible matrix effects. Further to this, significant amounts of Dy were sometimes detected in Tb samples and vice versa. For example, **3-Tb** and **2-Tb** were found to contain 7 and 1 ppb of ^163^Dy, respectively, whereas 2 ppb of ^159^Tb was seen in **2-Dy**. The amount of these trace impurities will inevitably vary between batches of Ln_2_O_3_ starting materials depending on the commercial source and between synthetic steps. Finally, the similarities of the vibrational modes of Tb/Dy pairs were shown by ATR-IR spectroscopy (Figures S17–S23). Together, these data, in combination with electronic spectra and magnetic susceptibility measurements, give confidence that the solid-state structures determined for **1-Ln**, **2-Ln**, and **3-Ln** are representative of bulk samples (see below).

### Structural Characterization

**Figure 1:**
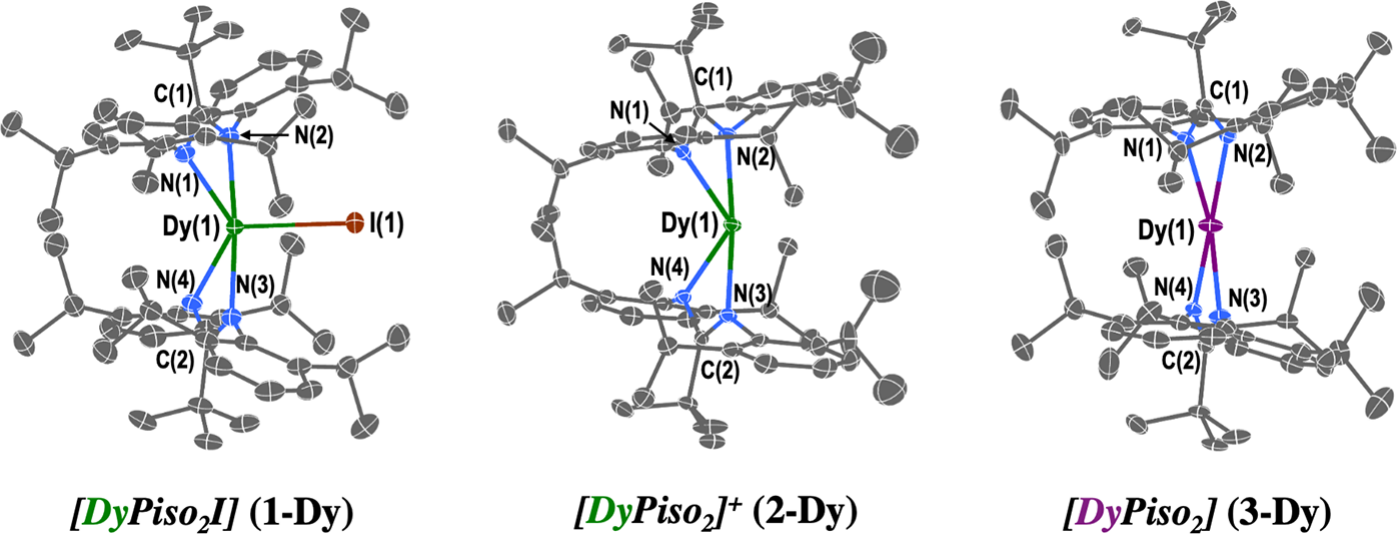
Molecular structures of **1-Dy**, **2-Dy**, and **3-Dy**, with selected atom labeling (counteranion not shown for **2-Dy**). The displacement ellipsoids are set at the 50% probability level, and hydrogen atoms and lattice solvent are omitted for clarity. Key: Dy(III) = green, Dy(II) = purple, I = brown, N = blue, and C = gray.

The solid-state structures of **1-Ln**, **2-Ln**, and **3-Ln** were determined by single-crystal X-ray diffraction (see Figure 1 for depictions of **1-Dy**, **2-Dy**, and **3-Dy** and Figures S56–S63 and Table 1 for other structures and metric parameters). Crystals of [Ln(Piso)_2_][B(C_6_F_5_)_4_] were unsolvated, whereas [Ln(Piso)_2_] contains stoichiometric pentane in the crystal lattice. Lattice toluene was also seen in crystals of [Ln(Piso)_2_I], although a solvent-free polymorph was also determined for [Dy(Piso)_2_I]; as both polymorphs were present in the PXRD pattern, we include both data sets, and as the metrical parameters of the complexes are similar, we only include the data of the solvent-free polymorph in Table 1 for brevity. Complexes **1-Ln** are five-coordinate, with the Ln centers bound by one iodide and four N-donor atoms from the two Piso ligands (Figures S56 and S57). The coordination geometry is intermediate between trigonal bipyramidal and square pyramidal (τ_5_ = 0.87 for Tb and 0.88 for Dy). Complexes **2-Ln** and **3-Ln** are four-coordinate, with the Ln centers bound by the four N-donor atoms from the two Piso ligands (Figures S58–S61). In **2-Ln**, the geometry is intermediate between tetrahedral and square planar (τ_4_ = 0.45 for **2-Tb** and 0.47 for **2-Dy**), whereas for **3-Ln**, the coordination geometry is closer to square planar (τ_4_ = 0.27 for **3-Tb** and 0.28 for **3-Dy**).^57^ The XRD data sets obtained for the Ln(II) complexes **3-Ln** each feature metal sites disordered over two positions; thus, we limit our discussion to the major (85–90%) components.

**Table 1:** Selected Bond Distances (Å) and Angles (°) for **1-Ln**, **2-Ln**, and **3-Ln**

**Complex**	**1-Dy**	**1-Tb**	**2-Dy**	**2-Tb**	**3-Dy**	**3-Tb**
N(1)-Ln(1)	2.314(2)	2.321(2)	2.260(3)	2.267(2)	2.317(2)	2.330(2)
N(2)-Ln(1)	2.404(2)	2.424(2)	2.332(2)	2.352(2)	2.363(2)	2.366(2)
N(3)-Ln(1)	2.404(2)	2.424(2)	2.313(3)	2.335(2)	2.365(2)	2.367(2)
N(4)-Ln(1)	2.314(2)	2.321(2)	2.258(3)	2.257(2)	2.314(2)	2.312(3)
I(1)-Ln(1)	2.8918(2)	2.9157(3)				
N(1)-Ln(1)-N(3)	120.72(6)	120.80(8)	119.55(9)	121.20(7)	125.86(7)	125.85(8)
N(1)-Ln(1)-N(4)	122.26(10)	122.43(13)	118.90(9)	119.85(7)	155.90(9)	156.09(10)
N(2)-Ln(1)-N(3)	174.82(9)	174.54(12)	174.44(9)	176.04(7)	164.44(9)	164.95(10)
N(2)-Ln(1)-N(4)	120.73(9)	120.81(8)	118.52(9)	119.50(7)	129.42(8)	129.14(9)
C(1)-Ln(1)-C(2)	149.70(9)	148.55(12)	148.80(9)	150.01(7)	174.82(8)	174.58(9)
τ_5_ (**1-Ln**); τ_4_ (**2-3-Ln**)^a^	0.88	0.87	0.47	0.45	0.28	0.27

a The values of τ_4_ will range from 1.00 for a perfect tetrahedral geometry to 0 for a perfect square planar geometry. Intermediate structures, including trigonal pyramidal and seesaw, fall within the range of 0 to 1.00. τ_5_ gives an index of the degree of trigonality. A trigonal bipyramidal structure with *D*_3h_ symmetry has a τ_5_ = 1, whereas a square pyramidal structure with *C*_4v_ symmetry has τ_5_ = 0.^57^

The Ln-I bond lengths in **1-Tb** and **1-Dy** are 2.916(1) and 2.892(1) Å, respectively. The LnN_4_ cores of **1-Ln** are similar to those of **2-Ln**, with the same τ_4_ values for each Ln pair (0.45 for **1-Tb** and **2-Tb**; 0.47 for **1-Dy** and **2-Dy**). Complexes **1-Ln** and **2-Ln** feature similar twist angles between LnN_2_C least squares planes: 66.79(7)° (**1-Tb**), 67.01(7)° (**1-Dy**), 70.10(7)° (**2-Tb**), and 69.90(9)° (**1-Dy**) (Tables S3 and S4), which are close to seesaw geometries. Both **1-Ln** and **2-Ln** exhibit two sets of interligand N–Dy–N angles, which fall between 118.52(9) and 122.43(13)° for one set and between 174.44(9) and 176.04(7)° for the other. The Ln–N bonds in **2-Ln** range from 2.257(2) to 2.352(2) Å for **2-Tb** and 2.258(3) to 2.332(2) Å for **2-Dy**; these are shorter than those of **1-Ln**, which range between 2.314(2) and 2.424(2) Å (Table 1), due to the variation in coordination number. It is intriguing that removing the iodide ligand has such a small effect on the metric parameters for the remaining N-Ln-N bond angles (Table 1).

The twist angles between LnN_2_C least squares planes in **3-Ln** are 42.14(11)° (**3-Tb**) and 43.0(2)° (**3-Dy**). The two sets of interligand N–Ln–N angles in **3-Ln** fall either between 125.85(8) and 129.42(8)° or between 155.90(9) and 164.95(10)°. The twist angles of **3-Ln** are *ca*. 15° smaller than those of the related bis-guanidinate complex, [Yb(Giso)_2_] (57.00(9)°),^50^ but deviate more significantly from those of square planar [Ln(Giso)_2_] (Ln = Sm, 0.93(9)°; Eu, 1.21(10)°).^50^ This is attributed to the relatively short mean Ln–N distances for the later Ln (e.g., 2.344(4) Å for **3-Tb** and 2.340(4) Å for **3-Dy**, vs. 2.546(4) Å for [Sm(Giso)_2_]),^50^ leading to greater interligand repulsion. We note that the mean Ln–N distance in [Yb(Giso)_2_] (2.378(4) Å)^50^ is longer than that seen for **3-Tb** and **3-Dy** despite Yb being a smaller Ln; we posited that the discrepancy may be due to **3-Tb** and **3-Dy** having formal 4f^*n*^5d^1^ configurations, but we cannot make firmer conclusions from these data as both the ligand backbones and coordination geometries differ between these complexes. We therefore investigated the electronic structures of **3-Tb** and **3-Dy** further by other methods (see below).

Comparing the metric parameters for the three families of complexes, we find the following. The C(1)···Ln(1)···C(2) angles of the Ln(II) complexes **3-Tb** (174.58(9)°) and **3-Dy** (174.82(8)°) are close to linearity, whereas Ln(III) **1-Ln** (Tb: 148.55(12)°; Dy: 149.70(9)°) and **2-Ln** (Tb: 150.01(7)°; Dy: 148.80(9)°) are bent. As expected, there is a greater deviation in the ranges of Ln–N distances of **1-Ln** (Tb: 2.321(2)–2.424(2) Å; Dy: 2.314(2)–2.405(2) Å) and **2-Ln** (Tb: 2.257(2)–2.352(2) Å; Dy: 2.258(3)–2.332(2) Å) than those of **3-Ln** (Tb: 2.312(3)–2.367(2) Å; Dy: 2.313(2)–2.365(2) Å). The two shortest Ln–N distances in **1-Ln** and **2-Ln** are mutually *trans* to each other (N–Ln–N: **1-Tb**, 174.54(12)°; **1-Dy**, 174.82(9)°; **2-Tb**, 176.04(7)°; **2-Dy**, 174.44(9)°), whereas all other interligand N–Ln–N angles for these complexes lie between 118.52(9) and 122.26(10)°. We attribute the geometrical differences in **1-Ln**, **2-Ln**, and **3-Ln** to the greater electrostatic attraction of the ligands to smaller, more charge-dense Ln(III) vs Ln(II) centers.^3^ The electron-deficient Ln(III) centers of cationic **2-Ln** exhibit additional strong electrostatic interactions with a single Piso methyl group (Ln···C = 3.136(3) Å for **2-Tb** and 3.119(4) Å for **2-Dy**), which is a common feature in low-coordinate Ln complexes.^26^

### UV–Vis-NIR Spectroscopy

**Figure 2:**
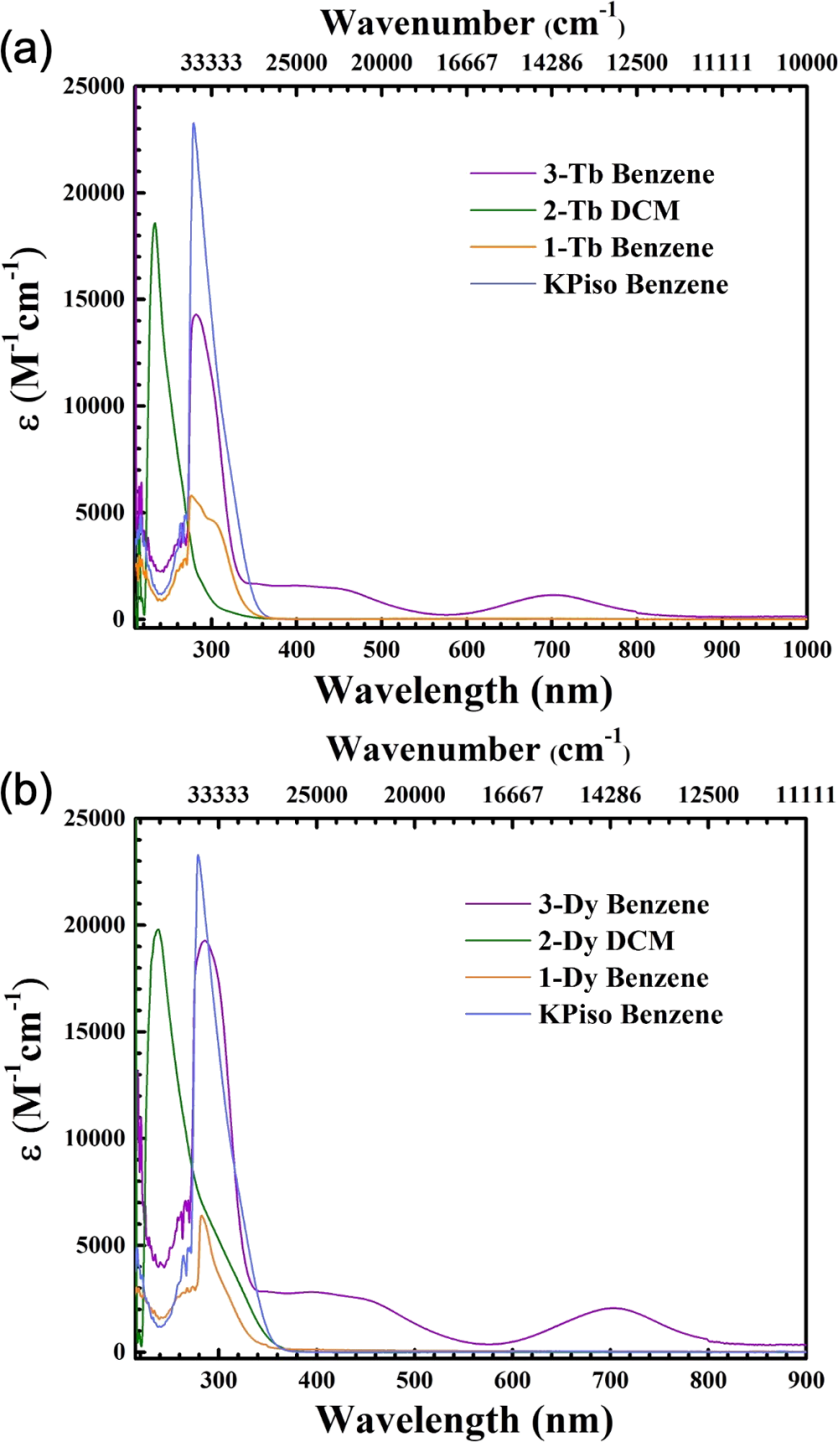
Experimental UV–vis-NIR spectra of 0.2 mM solutions of **3-Ln** (benzene), **2-Ln** (DCM), **1-Ln** (benzene), and KPiso (benzene) at room temperature; Ln = Dy (a) and Tb (b).

The UV–vis-NIR absorption spectra of separate solutions of KPiso and **1-Ln**, **2-Ln**, and **3-Ln** were measured from 250 to 1000 nm (10,000–40,000 cm^–1^); benzene was used as the solvent for KPiso, **1-Ln**, and **3-Ln**, whereas DCM was required for the separated ion pairs **2-Ln** (Figure 2 and Figures S24–S54). All complexes showed strong and sharp absorptions below 350 nm (ε > 5000 M^–1^ cm^–1^), which appear to be a feature intrinsic to both HPiso and KPiso (Figure S24) and likely arise from π to π* transitions in the ligand backbone. The Ln(III) complexes **1-Ln** and **2-Ln** are colorless, and their electronic absorption spectra are essentially featureless in the visible and NIR region, with no observable Laporte-forbidden f–f transitions.^1^ Solutions of the Ln(II) complexes **3-Tb** and **3-Dy** are dark green, and each shows two broad absorption bands stretching across most of the visible region; maxima are at ∼14,000 cm^–1^/∼700 nm (**3-Tb**, ε = 2100 M^–1^ cm^–1^; **3-Dy**, ε = 2050 M^–1^ cm^–1^) and ∼22,000 cm^–1^/∼450 nm (**3-Tb**, ε = 3050 M^–1^ cm^–1^; **3-Dy**, ε = 2800 M^–1^ cm^–1^), with the latter absorptions having shoulders at ∼350 nm bridging to π to π* transitions that are similar to those seen for **1-Ln** and **2-Ln**.

UV–vis-NIR spectroscopy was used to further probe the stability of **3-Ln** in a range of solvents (Figures S51–S52), as Tb(II) and Dy(II) complexes are often thermally unstable.^1−3,58^ THF solutions of **3-Ln** both turn from green to colorless within several seconds at room temperature, likely due to the highly reducing Ln(II) centers promoting the ring-opening of THF;^10^ the resultant UV–vis-NIR spectra are essentially featureless >350 nm, in common with the Ln(III) complexes **1-Ln** and **2-Ln**. In contrast, dark green solutions of **3-Ln** in hexane, benzene, and diethyl ether are relatively stable and give strong broad absorptions >350 nm. The high thermostability of hexane solutions of **3-Ln** was demonstrated by heating at 80 °C, first for 1 h and then for 15 h. The solutions maintained their green colors and gave UV–vis-NIR spectra with the same absorption features as fresh solutions but with lower extinction coefficients (Figures S53 and S54).

### Magnetism

**Figure 3:**
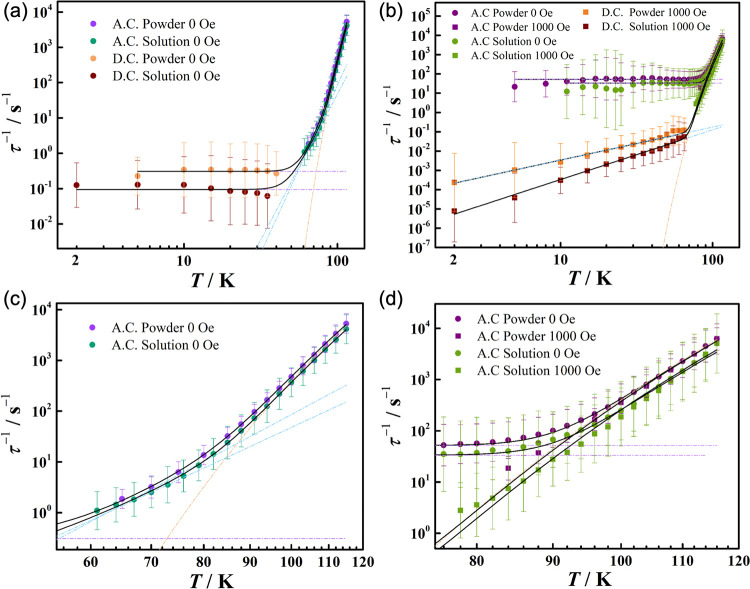
Plots of the natural log of the inverse relaxation time vs temperature for (a) powder and solution sample of **3-Tb** under 0 Oe and (b) **3-Dy** under 0 (circle points) and 1000 (square points) dc field (violet, purple, cyan, and green points are from ac data; orange and wine points are from dc data) at a temperature range from 2 to 150 K. (c) The enlarged scales of panel a from 60 to 120 K for **3-Tb**. (d) The enlarged scales of panel b from 70 to 120 K for **3-Dy**. The dashed orange, blue, and purple lines show isolated Orbach, Raman, and QTM fitting, respectively; black lines show their sum.

**Figure 4:**
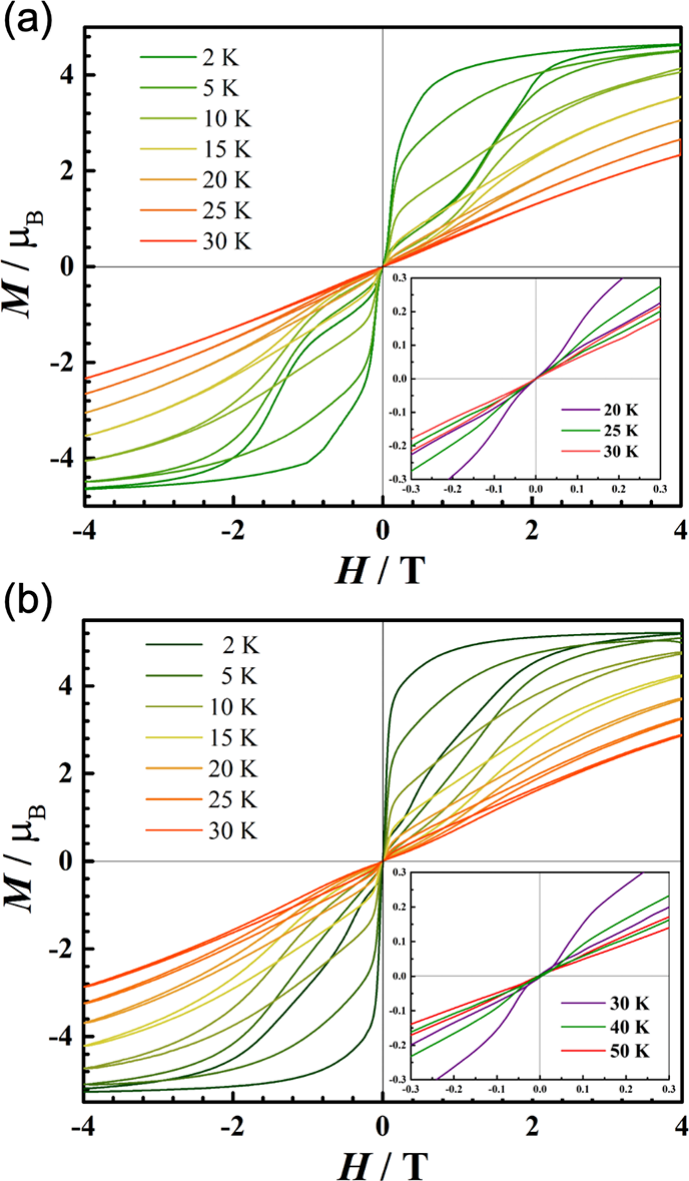
Magnetization *M* (μ_B_) vs applied dc field *H* (T) plots for (a) **3-Tb** and (b) **3-Dy**, at temperature (K) intervals between 2 and 30 K at an average sweep rate of 22 Oe/s, to show the shapes of hysteresis loops. (Inset) Expanded view of the variable-field magnetization near the zero field at 20 to 50 K.

Static and dynamic magnetic susceptibility data were collected for microcrystalline samples of **1-Ln**, **2-Ln**, and **3-Ln** suspended in eicosane (Figures 3 and 4, Figures S70–S149; Table 2). The magnetic susceptibility temperature product (χ_M_*T*) was determined for **1-Ln**, **2-Ln**, and **3-Ln** between 1.8 and 300 K under an applied dc field of 1000 Oe. The χ_M_*T* products at 300 K of the Ln(III) complexes **1-Tb**, **2-Tb**, **1-Dy**, and **2-Dy** are 11.60, 11.27, 13.62, and 13.57 cm^3^ mol^–1^ K, respectively; these values are comparable to the corresponding expected free-ion χ_M_*T* values for 4f^8^ Tb(III) (11.81 cm^3^ mol^–1^ K) and 4f^9^ Dy(III) (14.17 cm^3^ mol^–1^ K). The χ_M_*T* values of **1-Dy** and **2-Dy** show little temperature dependence until a sharp drop below 8.0 and 3.8 K, respectively, likely as a result of slow magnetic dynamics (Figures S71 and S73). For **1-Tb** and **2-Tb**, there is a more substantial, yet still gradual, decrease in χ_M_*T* as the temperature is reduced, although neither compound shows a precipitous drop at low temperature. Whereas **2-Tb** exhibits a smooth downturn in χ_M_*T* below ca. 20 K (Figures S70 and S72), the data for both **1-Tb** and **2-Tb** suggest that magnetic dynamics are faster than the timescale of the experiment.

**Table 2:** Magnetic Relaxation Parameters for **1-Ln**, **2-Ln**, and **3-Ln**^a^

complexes	*U*_eff_ (K)	log[τ_0_] (s)	log[*C*] (s^–1^ K^–*n*^)	*n*	log[τ_QTM_] (s)
**1-Tb**^b^ (1500 Oe)	491 ± 32	–8.97 ± 0.37	–1.81 ± 0.06	3.05 ± 0.05	
**1-Dy**^b^ (0 Oe, slow)	800 ± 16	–11.80 ± 0.18	–5.36 ± 0.09	4.50 ± 0.07	1.47 ± 0.02
**1-Dy**^b^ (0 Oe, fast)			–4.36 ± 0.14	6.14 ± 0.12	
**2-Tb**^b^ (1500 Oe)	725 ± 21	–8.91 ± 0.16	–2.34 ± 0.04	2.95 ± 0.03	
**2-Dy**^b^ (0 Oe)			–2.14 ± 0.14	3.57 ± 0.09	–1.26 ± 0.11
**3-Tb**^b^ (0 Oe)	1920 ± 91	–10.93 ± 0.35	–17.10 ± 1.96	9.52 ± 1.06	0.51 ± 0.02
**3-Tb**^b^ (1500 Oe)	1920^d^	–11.03 ± 0.04	–7.63 ± 0.24	4.39 ± 0.16	3.25 ± 0.08
**3-Tb**^c^ (0 Oe)	1920^d^	–10.84 ± 0.04	–14.84 ± 1.33	8.26 ± 0.72	1.02 ± 0.03
**3-Dy**^b^ (0 Oe)	1964 ± 48	–11.10 ± 0.20			–1.71 ± 0.02
**3-Dy**^b^ (1000 Oe)	1964^d^	–11.16 ± 0.03	–4.28 ± 0.09	1.82 ± 0.06	
**3-Dy**^c^ (0 Oe)	1964^d^	–10.92 ± 0.04			–1.44 ± 0.03
**3-Dy**^c^ (1000 Oe)	1964^d^	–10.94 ± 0.03	–6.05 ± 0.12	2.58 ± 0.08	

a Obtained from fitting ac and dc relaxation data with CC-FIT2^59,60^ using the equation  to define the temperature dependence of the relaxation time τ^–1^, where *U*_eff_ is the effective barrier to magnetic reversal, τ_0_ is the attempt time of the phonon bath, *C* and *n* define Raman relaxation, and τ_QTM_ is the QTM time scale.

b Crystalline samples.

c Solution samples.

d Parameter value was fixed during fitting.

The Ln(II) complexes **3-Tb** and **3-Dy** show higher χ_M_*T* products at 300 K than **1-Ln** and **2-Ln** for the respective ions (12.60 and 15.03 cm^3^ mol^–1^ K, respectively, Figures S74 and S75), which could be compared with free-ion χ_M_*T* values for 4f^9^ Tb(II) (14.17 cm^3^ mol^–1^ K) and 4f^10^ Dy(II) (14.06 cm^3^ mol^–1^ K) electron configurations, indicating that a 4f^*n*+1^ electron configuration is not the best model for **3-Tb** or **3-Dy**. The 4f^*n*^5d^1^ configuration may be a better description for their electronic structures, and a detailed study of the electronic structure is discussed below. The χ_M_*T* values of **3-Tb** and **3-Dy** decrease slowly with temperature from 300 to *ca*. 18 K and then fall sharply to reach 8.01 and 3.84 emu K mol^–1^, respectively, at 2 K. These data indicate that the Ln(II) complexes **3-Ln** exhibit slower magnetization dynamics than the Ln(III) complexes **1-Ln** and **2-Ln**. The zero-field-cooled (ZFC) and field-cooled (FC) magnetic susceptibility traces support this assertion (Figures S76–S78), showing bifurcation at 17 K for **3-Tb** and between 24 and 28 K for **3-Dy** (Figures S79 and S80), whereas bifurcation occurs below 10 K for **1-Dy** and below 5 K for **1-Tb**, **2-Tb**, and **2-Dy** (Figures S76–78). Susceptibility measurements for solution samples of **3-Ln** show ZFC-FC bifurcation at a higher temperature than in the solid state, between 30 and 40 K for **3-Tb** and 48 K for **3-Dy** (Figure S81).

The magnetic relaxation rates of **1-Ln**, **2-Ln**, and **3-Ln** were probed by ac magnetic susceptibility and dc magnetization decay experiments. Ac data were fitted with a generalized Debye model,^27^ and dc magnetization decay data were fitted with a stretched exponential model, both in CC-FIT2,^59,60^ unless stated otherwise. The temperature dependence of magnetic relaxation in SMMs is generally described with the equation , which encompasses three terms: the overbarrier Orbach mechanism (where *U*_eff_ is the effective barrier to magnetic reversal and τ_0_ is the attempt time of the phonon bath), the through-barrier two-phonon Raman mechanism (phenomenological parameters *C* and *n*), and τ_QTM_ as the QTM time scale. Functions of this type were fitted using CC-FIT2,^59,60^ and the resulting parameters and their estimated standard deviations (ESDs) are given in Table 2.

For **1-Tb** and **2-Tb**, we observed efficient QTM in zero dc field, attributed to the non-Kramers Tb(III) ion having a nonzero splitting between the lowest-lying pseudodoublet states (|+*m*_*J*_ > and |−*m*_*J*_>). Under an optimum dc field of 1500 Oe (Figures S82–S83), we could observe χ_*m*_″ peaks between 6 and 42 K for **1-Tb** (Figure S83) and up to 64 K for **2-Tb** (Figure S87). In both cases, the magnetic relaxation rates were then fitted with Orbach and Raman processes ; Figures S127 and S129; Table 2). As relaxation is rapid at higher temperatures for **1-Tb**, the extracted *U*_eff_ value is less reliable. For **1-Dy**, χ_*m*_″ peaks were observed under zero dc field between 6 and 42 K (Figures S84 and S85), and the rates fitted to Orbach and Raman processes (Figure S128; Table 2). Meanwhile, for **2-Dy** (where χ_*m*_″ peaks are observed under zero dc field between 2 and 48 K (Figure S88) and are asymmetric and thus we fit these data with the Havriliak–Negami model^61^), the rates appear to follow a combination of Raman and QTM relaxation processes (; Figure S130; Table 2). Close inspection might hint at an increase in relaxation rate at the highest temperatures above the fitted Raman contribution, possibly suggesting the onset of an Orbach process; however, this is obscured by an efficient Raman process in this case.

For **3-Tb** peaks in the out-of-phase susceptibility, χ_*m*_″ peaks were observed between 116 and 65 K at frequencies from 0.1 to 1000 Hz, whereas for **3-Dy**, peaks were observed between 116 and 2 K from 10 to 1000 Hz; however, below 88 K for **3-Dy**, the χ_*m*_″ peak maxima do not shift, indicative of QTM (Figure S93). Hence, we also collected ac susceptibility data for **3-Tb** and **3-Dy** under optimal 1500 and 1000 Oe dc fields (Figures S91, S96 and S99), respectively, showing that QTM is suppressed for **3-Dy** (Figure S96). In the absence of isostructural diamagnetic hosts, we also collected ac magnetic susceptibility data for frozen solutions of **3-Ln** in hexane (50 mM) to reduce intermolecular dipolar interactions that facilitate QTM. Regardless of using an applied dc field or the solution samples, the relaxation rates in the high-temperature Orbach regime agree with the respective microcrystalline solid samples, although the frozen solution data are noisier because of the high dilution (Figure 3 and Figures S133, S136, and S137). Hence, we have fixed the *U*_eff_ value during fitting the relaxation rates for the in-field and solution measurements to improve the reliability of the fitted values. The relaxation rates for **3-Tb** are best described by a combination of Orbach, Raman, and QTM processes irrespective of a dc field or if in frozen solution phase ; Figure 3a,c, Figures S131–S133,Table 2). The fitted values for the Raman parameters under a 1500 Oe dc field are significantly different from those in zero field, which are likely due to the small temperature range over which the Raman mechanism is observed in zero field; hence, the values are more reliable when obtained at 1500 Oe. Compound **3-Dy** on the other hand shows no obvious Raman process in zero dc field and so can be fitted with Orbach and QTM processes alone ; Figure 3b,d, Figures S134 and S136, Table 2), whereas under a 1000 Oe field, there is no QTM process, and a Raman process is now observed (; Figure 3b,d, Figures S135 and S137, Table 2).

Variable temperature magnetization vs dc field measurements of **1-Ln**, **2-Ln**, and **3-Ln** were performed with field sweep rates of 22 Oe/s; all traces showed butterfly-shaped hysteresis loops at low temperatures, confirming efficient QTM at zero field (Figure 4 and Figures S138–S149). As expected,^28^ by 2 K, the hysteresis loops for the non-Kramers Tb(III) complexes **1-Tb** and **2-Tb** are closed in the zero field region, and these loops are only slightly open at higher fields at this temperature (Figures S144 and S146). The *T*_H_ values of the Dy(III) complexes **1-Dy** (Figure S145) and **2-Dy** (Figure S147) are 10 and 4.5 K, respectively, which are substantially lower than the best-performing monometallic Dy(III) SMM to date, [Dy(C_5_^i^Pr_5_)(C_5_Me_5_)][B(C_6_F_5_)_4_] (*T*_H_ = 72 K).^35^ Hysteresis loops of **3-Tb** and **3-Dy** are already closed at zero field by 2 K for both powder and solution samples (Figure 4 and Figures S148 and S149).

### EPR Spectroscopy

Continuous wave X-band (*ca*. 9.4 GHz) EPR spectra of polycrystalline samples of **2-Tb** and **3-Tb** at 5 K give similar spectra, differing only in intensity, with a near zero-field resonance (Figures S150 and S151). Given the relative stabilities of the Tb(III) and Tb(II) oxidation states, it seems likely that the resonance in the spectrum of **3-Tb** arises from a minor impurity of [Tb(Piso)_2_]^+^ and that **3-Tb** is otherwise EPR silent under these conditions. Given the non-Kramers nature of Tb(III) in **2-Tb**, this would suggest a zero-field splitting in the ground pseudodoublet of **2-Tb** of <0.4 cm^–1^. Complex **2-Dy** also gives a very weak, but otherwise similar, spectrum to that of **2-Tb**. Again, it seems likely that **2-Dy** is EPR silent, and there is a trace impurity of [Tb(Piso)_2_]^+^ in this sample too (Figure S152); given the sensitivity of EPR spectroscopy and purity of reagent-grade lanthanide salts, it is not uncommon to detect impurities of other lanthanides in the host compound, and the ICP-MS data obtained on a sample of **2-Dy** showed the presence of a trace amount of Tb (see above).

In contrast to the other samples, the X-band EPR spectrum of **3-Dy** gives an intense low-field resonance that is quite different to the other spectra (Figure S153). This feature is also found at the S-band (ca. 3.9 GHz; Figure S154) but does not correspond to the same effective *g*-value as found at the X-band (measuring at the peak maximum), suggesting that it is a field-independent feature. Given the non-Kramers nature of Dy(II), it likely corresponds to a zero-field splitting in the ground pseudodoublet of <0.2 cm^–1^. Hence, it seems that **2-Dy** is EPR silent whereas **3-Dy** is EPR active under these conditions. We were unable to measure any significant EPR signals from frozen solutions at the X-band and 5 K: this is presumably due to the intrinsically weak spectra and the more dilute medium, but it is also possible that there are minor structural changes in the solution, for instance, a more axial geometry leading to less mixing of the non-Kramers pseudodoublets and hence weaker transition probabilities, that lead to less EPR intensity.

### *Ab Initio* Calculations

We employed OpenMolcas^62^ to perform CASSCF-SO calculations on **1-Ln**, **2-Ln**, and **3-Ln** using atomic coordinates obtained from single-crystal XRD to probe the electronic structures of these complexes (see SI for details). The electronic structure of the trivalent compounds **1-Ln** and **2-Ln** is well-approximated by standard minimal CASSCF-SO calculations (CAS(8,7)SCF for Tb(III) and CAS(9,7)SCF for Dy(III)), giving results that are typical for low-coordinate Tb(III) and Dy(III) compounds: here, the amidinate ligands function as strong axial pseudomonodentate donors, and the iodide anions in **1-Ln** are considerably weaker donors (Tables S31 and S32 cf. Tables S33 and S34). The main difference between **1-Ln** and **2-Ln** is the presence or absence of the iodide ion, as the local geometries of the [Ln(Piso)_2_]^+^ fragments are similar. The ground (pseudo)doublets for **1-Tb**, **2-Tb**, **1-Dy**, and **2-Dy** are well described as *m*_*J*_ = ±6, ±6, ±15/2, and ±15/2, respectively, and are all quantized approximately parallel to the C(1)–C(2) vector (Figures S155–S158). All four compounds have large gaps to their first-excited states (313, 391, 233, and 269 cm^–1^, respectively); in both cases, these gaps are larger for **2-Ln** than for **1-Ln**, and the low-lying (pseudo)doublets have purer *m*_*J*_ wave functions in **2-Ln**, where both features are expected as the competing equatorial CF influence of the iodide ion is absent.

**Figure 5:**
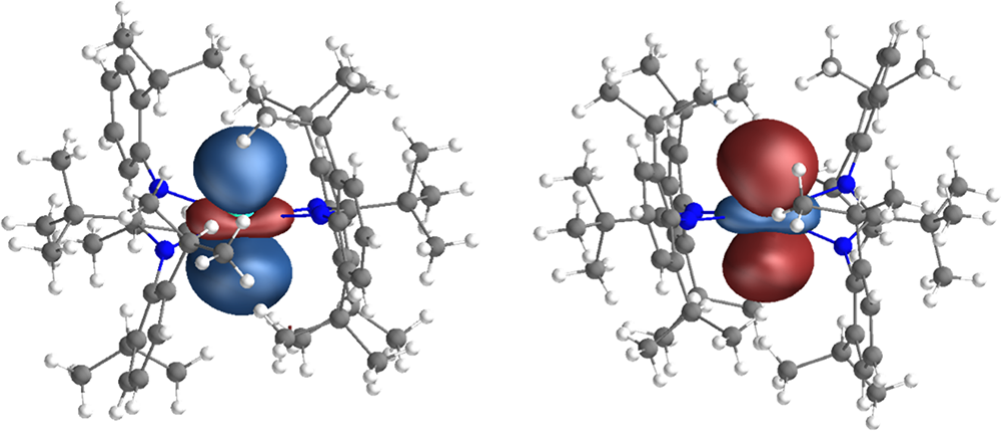
Singly degenerate 5d orbital for **3-Tb** (left) and **3-Dy** (right), calculated with CASSCF-SO, shown at an isosurface value of 0.04 au. The phases of the wave functions (colored lobes) are not observable and could be arbitrarily reversed.


To determine the electronic structure of **3-Ln**, we must carefully determine the appropriate active space. We performed our tests on **3-Dy** and started with a CAS(10,8)SCF calculation for 11 roots of the high-spin *S* = 3 configuration, which would be appropriate for the ground *L* = 5 (H) term assuming a 4f^9^5d^1^ ground configuration with a nondegenerate 5d orbital. After converging this calculation, we included the nine lowest-lying unoccupied orbitals into a restricted active space allowing one excitation out of the complete active space and performed a configuration interaction (CI) calculation for 50 roots of the *S* = 3 spin states (i.e., RAS(10;0/8/9;0,1)CI, where the first numeral indicates the total number of active electrons, the slash-delimited triplet indicates the sizes of the RAS1/RAS2/RAS3 subspaces, and the final numerals indicate the excitations out of (into) RAS1(RAS3)). This showed 11 roots below 2700 cm^–1^ (arising from the ^6^H term of the 4f^9^ configuration), a further 7 roots between 8600 and 9600 cm^–1^ (arising from the ^6^F term of the 4f^9^ configuration), and the next excited states lying above ca. 35,000 cm^–1^, thus confirming a 4f^9^5d^1^ configuration with a nondegenerate 5d orbital for the high-spin *S* = 3 state. The resulting nondegenerate state-average (pseudonatural) orbital is d_z2_-like in appearance, with its axial lobes oriented perpendicular to the C(1)–C(2) vector, and has a breakdown of 51% 5d and 17% 6s (determined using Lödwin orthogonalization, Figure 5 and Table S43), similar to that observed previously in Ln(II) metallocene complexes.^18,20^ However, we note that the orbital contribution is different when reported using Mulliken partitioning onto atomic orbitals (giving 48% 5d and 47% 6s for the ground *S* = 3 root) and may then differ depending on which spin-free root is interrogated.^63^ We subsequently performed CAS(10,8)SCF calculations for 18 roots for both the *S* = 3 and *S* = 2 states for **3-Dy** using the original active space (thus accounting for both ^6^H and ^6^F terms arising from the 4f^9^ configuration), finding average orbital occupations for both multiplicities corresponding to a 4f^9^5d^1^ configuration and giving well-separated spin-free terms ^7^H (*E* < 2400 cm^–1^), ^5^H (4300 < *E* < 6200 cm^–1^), ^7^F (8400 < *E* < 9300 cm^–1^), and ^5^F (12,300 < *E* < 13,100 cm^–1^) before spin–orbit coupling, with no indication of a ^5^I term arising from a possible 4f^10^ configuration. This confirms that the ground configuration is indeed 4f^9^5d^1^ and that the f–d coupling obeys Hund’s rules, and further indicates that f–f interelectronic repulsion > f–d coupling > CF splitting. To examine the influence of dynamic correlation on the electronic structure, we have also performed multistate complete active space second-order perturbation theory (CASPT2) calculations on the 18 roots of each multiplicity.^64^ Whereas the spread of each term is more or less unchanged, the energy of the excited terms is significantly reduced: the ^5^H term is lowered by ∼1000 cm^–1^ (3000 < *E* < 5000 cm^–1^), the ^7^F term is lowered by ∼2000 cm^–1^ (6500 < *E* < 7600 cm^–1^), and the ^5^F term is lowered by ∼3000 cm^–1^ (9300 < *E* < 10,100 cm^–1^), relative to the CASSCF results. This is due to a reduction in the interelectron repulsion, i.e., reduced Slater integrals or Racah parameters, for both 4f–4f and 4f–5d electron pairs. Upon mixing all states under spin–orbit coupling (SOC), the ground pseudodoublet (which has a tunnel splitting of 0.00002 cm^–1^ at the CASSCF-SO level or 0.0002 cm^–1^ at the CASPT2-SO level, as Dy(II) is non-Kramers) is dominated by the first and second spin-free roots, meaning that it derives from the ^7^H term. To interrogate the f–d coupling further, we project the total *ab initio* Hamiltonian onto a model space comprising of *S*_5d_ = 1/2, *S*_4f_ = 5/2, and *L*_4f_ = 5; we do so by projecting the *L*_4f_ = 5 (H) subspace from the spin-free CASSCF/CASPT2 Hamiltonians and thus removing the *L*_4f_ = 3 (F) content,^65^ which is justified as the f–f interelectronic repulsion is greater than the f–d coupling here. Then we project these reduced-dimension *ab initio* Hamiltonians onto a model Hamiltonian accounting for f–d coupling, CF splitting of the 4f states, and 4f SOC (eq 1, Tables S35 and 36, where the *R̂* operators represent the spin of the 5d electron (for which *L*_5d_ = 0), the *Ô*_*k*_^*q*^ operators are extended Stevens operators that are polynomials of the 4f orbital angular momentum operators *L̂*, and the *Ô*_*n*_^*m*^ operators are extended Stevens operators that are polynomials of the 4f spin angular momentum operators *Ŝ*; the θ_*k*_ are operator equivalent factors).^22,23,65−68^ For the CASSCF-SO {CASPT2-SO} Hamiltonians, this projection gives a root-mean-square (RMS) error of <0.4 {6.7} cm^–1^, indicating an excellent quality model. We find that the SOC constant is λ = −396 {−396} cm^–1^, as expected for a 4f^9^ configuration,^22,23,66^ but that it is smaller than the largest f–d coupling terms that are dominated by the isotropic spin–spin coupling, having an effective isotropic coupling parameter of Ω = −1310 {−877} cm^–1^ ( for the effective operator Ω*R̂* · *Ŝ*). The Ω parameter being negative indicates the parallel alignment of the d and f spin momenta (as discussed above) and allows us to give the final ordering of energy scales as f–f interelectronic repulsion > f–d coupling > SOC > CF splitting. There are several other significant anisotropic f–d coupling terms (Tables S35 and S36) that are all of a tripartite character (effective operators like *R̂*_α_*Ô*_*n*_^*m*^*Ô*_*k*_^*q*^); most of these are isotropic in the spin–spin part with even *k* and *q* (i.e., *R̂*_α_*Ŝ*_α_*Ô*_*k*_^*q*^), but two are more complicated (*R̂*_α_*Ô*_2_^± 1^*Ô*_5_^–2^). Comparing the CASSCF-SO and CASPT2-SO results, the isotropic spin–spin coupling is reduced by a factor of 0.67, but the effect on the largest tripartite terms is unpredictable: *R̂*_α_*Ô*_2_^± 1^*Ô*_5_^–2^ is not affected at all, and *R̂*_α_*Ŝ*_α_*Ô*_8_^–2^ is increased by 300%.

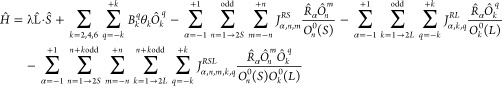
1

Given the structural and spectroscopic similarities of **3-Dy** and **3-Tb**, we proceed to calculate the electronic structure for **3-Tb** assuming a 4f^8^5d^1^ ground configuration. Analogously, we perform CAS(9,8)SCF calculations for seven *S* = 3.5 and seven *S* = 2.5 roots, corresponding to the ^8^F and ^6^F terms of Tb(II); the resulting nondegenerate state-average (pseudonatural) orbital is 53% 5d and 16% 6s (using Lödwin orthogonalization, Figure 5 and Table S43), very similar to that found for **3-Dy** (using Mulliken partitioning of the ground *S* = 3.5 root gives 51% 5d and 45% 6s). The ^8^F and ^6^F terms lie below 2500 cm^–1^ and above 4800 cm^–1^, respectively, which confirms that the 4f^8^5d^1^ ground configuration obeys Hund’s rules. Addition of CASPT2 corrections leads to a slight increase in the spread of the ^8^F term (<2800 cm^–1^) and a lowering of the ^6^F term (>3100 cm^–1^).^22^ To quantify the spin-coupling of the electronic states, we first mix the spin-free states with SOC and then project the entire set of 98 *ab initio* SO states onto a model Hamiltonian (eq 1, Tables S36 and 37) spanning *S*_5d_ = 1/2, *S*_4f_ = 3, and *L*_4f_ = 3, giving an RMS error for the projection of <0.3 cm^–1^ (for both CASSCF-SO and CASPT2-SO). Similarly to **3-Dy**, the SOC constant (λ = −297 cm^–1^, for both CASSCF-SO and CASPT2-SO) is as expected for a 4f^8^ configuration,^22,23,66^ but it is smaller than the largest isotropic spin–spin f–d coupling term, which has an effective isotropic coupling parameter of Ω = −1334 {−857} cm^–1^ for CASSCF-SO {CASPT2-SO}. The ordering of energy scales is therefore f–d coupling > SOC > CF splitting (we have not assessed the energy scale of the f–f interelectronic repulsion in this case by examining only the ^7^F states of the 4f^8^ subspace, although interelectronic repulsion is exactly treated within the complete active space). Similarly as for **3-Dy**, there are several significant anisotropic tripartite f–d coupling terms (Tables S36 and S37); again, most are isotropic in the spin–spin part with even *k* and *q*, but in this case, we see two terms like *R̂*_α_*Ô*_2_^± 1^*Ô*_3_^2^. Comparing the CASSCF-SO and CASPT2-SO results, the isotropic spin–spin coupling drops by a factor of 0.64, very similar to the effect found for **3-Dy**, and similarly, some tripartite terms are not affected at all (*R̂*_α_*Ô*_2_^± 1^*Ô*_3_^2^), and yet *R̂*_α_*Ŝ*_α_*Ô*_2_^–2^ reduced by 96%. Curiously, the more complicated terms that are unaffected by CASPT2 corrections have *k* = 3 for **3-Tb** and *k* = 5 for **3-Dy**, which match their respective values of *L*_4f_.

## Discussion

The single-crystal XRD data collected for **2-Ln** and **3-Ln** show differences in metrical parameters that are consistent with the latter having Ln(II) centers with formal 4f^*n*^5d^1^ valence electron configurations. The mean Ln–N distances of **3-Tb** (2.344(4) Å) and **3-Dy** (2.340(4) Å), respectively, increase by 0.041 and 0.049 Å from the corresponding Ln(III) cations (2.303(3) Å for **2-Tb** and 2.291(4) Å for **2-Dy**). Similar trends have previously been observed for Ln(II)/Ln(III) pairs of both trigonal and axial Cp^R^ complexes; e.g., Ln(II) centers with formal 4f^*n*^5d_*z*2_^1^ configurations in [Ln(Cp′)_3_]^−^ (Cp′ = {C_5_H_4_SiMe_3_}) and [Ln(C_5_^i^Pr_5_)_2_] show mean Ln···Cp^R^_centroid_ distances that are 0.02–0.06 Å longer than their respective Ln(III) counterparts [Ln(Cp′)_3_]^8^ and [Ln(C_5_^i^Pr_5_)_2_][B(C_6_F_5_)_4_].^18^ In contrast, the mean Ln···Cp′_centroid_ distances of [Ln(Cp′)_3_]^−^ anions for Ln(II) centers with 4f^*n*+1^ configurations are 0.123–0.156 Å longer than their corresponding parent Ln(III) [Ln(Cp′)_3_].^7,8^

The high energy absorptions in the visible absorption spectra of **3-Ln** are consistent with those previously reported for the corresponding Tb(II) and Dy(II) complexes [K(2.2.2-crypt)][Ln(Cp′)_3_]^7,8^ and [Ln(C_5_^i^Pr_5_)_2_]^20^ and are considered to be diagnostic of Ln(II) ions with 4f^*n*^5d_*z*2_^1^ electronic configurations.^9,10^ Complexes **3-Ln** show good thermal stability in solution, comparable to the axial Tb(II) and Dy(II) metallocenes [Ln(C_5_^i^Pr_5_)_2_] that are stable in solutions at room temperature,^18,20^ which is an improvement over the vast majority of trigonal Tb(II) and Dy(II) complexes of the general formula [LnL_3_]^−^ that often decompose above −30 °C.^2,3,58^

Counterintuitively, it seems that the Kramers systems **2-Dy** and **3-Tb** are EPR silent, whereas the non-Kramers systems **3-Dy** and **2-Tb** are EPR active, as measured from polycrystalline samples at the X-band and 5 K. This can be rationalized from the results of the CASSCF/CASPT2-SO calculations. For all four complexes, the lowest pair of states (either a doublet or a pseudodoublet) is isolated from the first excited pair of states by several hundred cm^–1^ (Tables S33, S34, S39–S42); hence, any EPR transitions we are observing must be within the ground (pseudo)doublet. The ground-state doublet of Kramers **2-Dy** is found to be 99% pure *m*_*J*_ = ±15/2 with *g*_x,y_ = 0 (Table S33) and therefore should be EPR silent. For **3-Tb**, we can use the projected model parameters (see *Ab Initio Calculations*, above) to rebuild the Hamiltonian in an angular momentum basis and obtain its eigenstates (Tables S39 and S40),^22,23^ showing that the ground doublet of **3-Tb** is ca. 98% *m*_*J*_ = ±13/2 from a *J* = 13/2 spin–orbit multiplet, which would indeed be expected to be EPR silent. The very low-field resonances of **3-Dy** and **2-Tb** show that the bottom pair of states are indeed near-degenerate for these non-Kramers systems given that the microwave energy in the X-band EPR experiment is ca. 0.3 cm^–1^; this is consistent with the magnetic data. The observation that the low-field resonances do not behave as effective *g*-values when measured at multiple microwave frequencies (Figures S153 and S154) indicates that the transitions are occurring within a zero-field split pseudodoublet. Our CASSCF-SO calculations give zero-field splittings of the order 10^–5^ to 10^–4^ cm^–1^, and although the accuracy of such calculations cannot be trusted on this energy scale, they do indicate a pseudodoublet ground state in both cases. Despite the ground pseudodoublet of **2-Tb** being well described as 100% *m*_*J*_ = ±6, the fact that it is non-Kramers and the molecular structure has no symmetry elements allows mixing between the two states and hence EPR intensity.^69^ This can be confirmed by calculating the magnetic moment transition operator , which is proportional to EPR intensity) between the two lowest-lying states with a 0.1 T field perpendicular to the main anisotropy axis, giving ∼8  for **2-Tb** and ∼10^–2^ for **2-Dy**; thus, the expected EPR intensity is nearly 3 orders of magnitude larger for the non-Kramers ion than for its Kramers analogue despite both having quite pure *m*_*J*_ ground states. Hence, the fact that **2-Tb** and **3-Dy** are non-Kramers and that CASSCF-SO suggest pseudodoublets is consistent with the EPR spectra.

**Figure 6:**
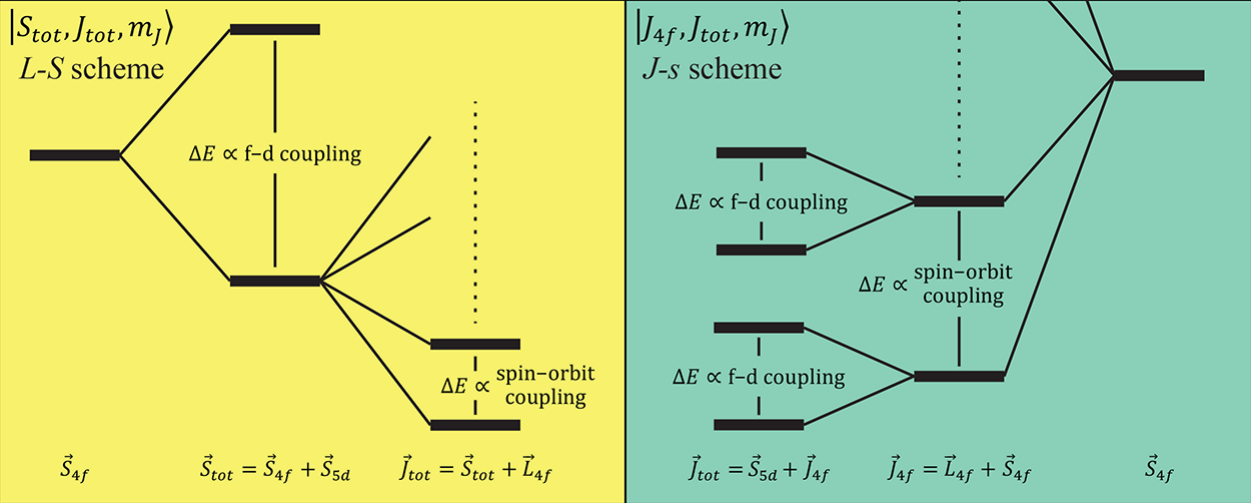
Schematic for *L*–*S* (left) and *J*–*s* (right) coupling schemes, ignoring CF splitting; energies not to scale.

**Figure 7:**
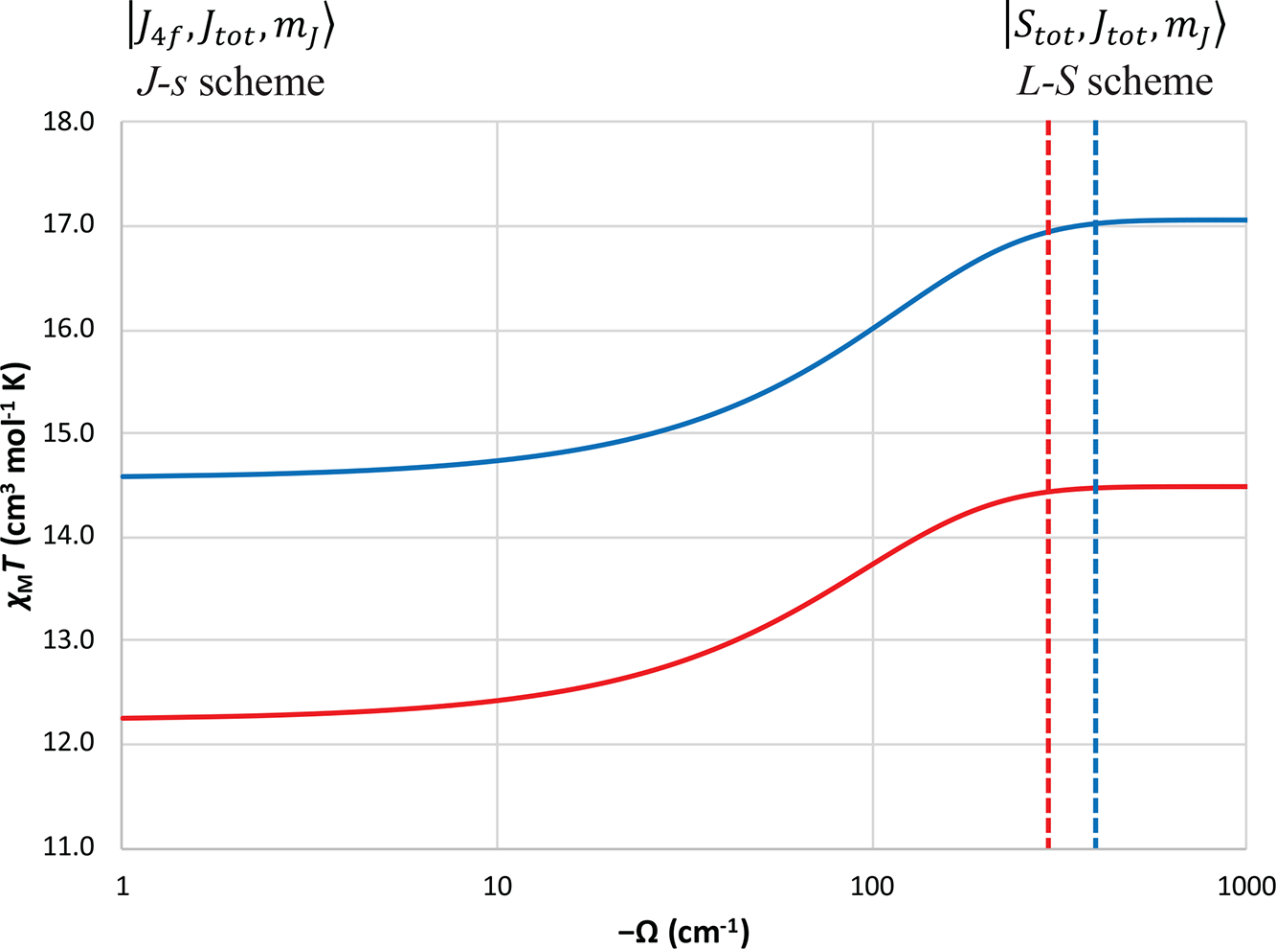
χ_M_*T* values at 300 K for 4f^8^5d^1^ Tb(II) (red) and 4f^9^5d^1^ Dy(II) (blue) as a function of the isotropic f–d coupling parameter Ω, assuming a nondegenerate 5d orbital. These curves are simulated using eq 2 with fixed λ = −297 and −396 cm^–1^ for Tb(II) and Dy(II), respectively. The dotted lines indicate where Ω = λ for each ion.

The measured χ_M_*T* products at 300 K for **3-Tb** (12.60 cm^3^ mol^–1^ K) and **3-Dy** (15.03 cm^3^ mol^–1^ K) are consistent with the corresponding values for the axial Ln(II) metallocenes [Tb(C_5_^i^Pr_5_)_2_] (12.72 cm^3^ mol^–1^ K) and [Dy(C_5_^i^Pr_5_)_2_] (15.15 cm^3^ mol^–1^ K)^20^ but differ from those for the trigonal planar [K(2,2,2-crypt)][Ln(Cp′)_3_] compounds (13.73 and 16.1 cm^3^ mol^–1^ K for Ln = Tb and Dy, respectively), which also formally exhibit 4f^*n*^5d^1^ valence electron configurations.^19^ It is commonplace in the literature to compare these data with values expected for different d–f coupling schemes to determine how the angular momenta couple.^18,19^ In the case of 4f^*n*^5d^1^ ground states with a nondegenerate d-orbital and where f–d coupling > SOC, one considers that the spin momenta couple first *S⃗*_tot_ = *S⃗*_4*f*_ + *S⃗*_5*d*_ followed by coupling of the total spin to the orbital angular momentum *J⃗*_*tot*_ = *L⃗*_4*f*_ + *S⃗*_tot_ (often called the *L*–*S* coupling scheme and herein referred to as the |*S*_tot_, *J*_tot_, *m*_*J*_⟩ basis). On the other hand, when the f–d coupling < SOC, then the angular momenta of the 4f shell could be considered to couple first *J⃗*_4*f*_ = *L⃗*_4*f*_ + *S⃗*_4*f*_ followed by coupling to the 5d spin momentum *J⃗*_*tot*_ = *J⃗*_4*f*_ + *S⃗*_5*d*_ (often called the *J*–*s* coupling scheme and herein referred to as the |*J*_4*f*_, *J*_tot_, *m*_*J*_⟩ basis). For Tb(II) and Dy(II) under the assumption of “ferromagnetic” f–d coupling (i.e., Hund’s rules are obeyed), both schemes lead to *J* = 13/2 and *J* = 8 ground multiplets, respectively, giving χ_M_*T* values at 300 K of 14.43 and 17.02 cm^3^ mol^–1^ K, respectively. The only difference between the two schemes is the energy scale of the splitting to the first excited multiplet (*J* = 11/2 or 7, respectively), which would be set by either the SOC (when f–d coupling dominates, *L*–*S* scheme) or the f–d coupling (when SOC dominates, *J*–*s* scheme) (Figure 6). As the 4f SOC is an atomic parameter and should not be greatly perturbed by coordination chemistry or the presence of a 5d electron, it can be considered to be fixed so that the only variable is the f–d coupling strength. If the f–d coupling were vanishingly weak (implying the *J*–*s* scheme) such that at 300 K both the ground and first excited *J* multiplets are thermally populated, then the χ_M_*T* values for Tb(II) and Dy(II) are 12.19 and 14.55 cm^3^ mol^–1^ K, respectively. However, both sets of values neglect CF splitting, and in fact, both coupling schemes are overly reductive: it is the combination of all terms in the Hamiltonian that determines the eigenstates (and therefore magnetic moments), and the reality is somewhere in between the two. This can be well illustrated by plotting the expected 300 K χ_M_*T* values for 4f^8^5d^1^ Tb(II) and 4f^9^5d^1^ Dy(II) with fixed SOC constants of λ = −297 and −396 cm^–1^, respectively, and variable f–d coupling for a simple Hamiltonian (eq 2) (Figure 7). Although including an axial CF based on the CASSCF-SO-calculated CF parameters for **3-Ln** reduces χ_M_*T* by ca. 3–5% across the whole range of f–d coupling strengths, the overall behavior is unchanged, and there is a smooth transition between the two regimes where the curves plateau as f–d coupling ≈ SOC.
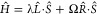
2

For **3-Tb** and **3-Dy**, the CASSCF-SO-calculated χ_M_*T* values at 300 K are 14.01 and 16.44 cm^3^ mol^–1^ K, respectively, whereas the inclusion of dynamic correlation via CASPT2-SO calculations gives slightly reduced χ_M_*T* values of 13.98 and 16.32 cm^3^ mol^–1^ K at 300 K, respectively. Based on the calculated values of Ω (from either CASSCF or CASPT2), both compounds are firmly in the f–d coupling > SOC regime; these calculated χ_M_*T* values are reduced by *ca*. 3% from the simple predicted values owing to CF splitting. However, both are higher than the experimental values by approximately 1.4 cm^3^ mol^–1^ K. Although our CASPT2-SO calculations show that CASSCF-SO does indeed overestimate the f–d coupling by ca. 50%, this has only a marginal influence on the 300 K χ_M_*T* value owing to the plateau on the right of Figure 7, hence the minimal change in calculated χ_M_*T* with CASPT2-SO. This tends to suggest that there is some uncertainty in the experimental values, which is not uncommon for air-sensitive molecules such as these. In any case, the experimental results are clearly in between the two limits (Figure 7), so χ_M_*T* on its own does not serve as a particularly useful measure to discriminate between the two regimes. To assess the coupling more directly, we can use the projected model parameters (see *Ab Initio Calculations*, above) to rebuild the Hamiltonian in either the |*S*_tot_, *J*_tot_, *m*_*J*_⟩ or the |*J*_4*f*_, *J*_tot_, *m*_*J*_⟩ basis and obtain its eigenstates (Tables S39 – S42).^22,23^ For **3-Dy**, the lowest 17 states arise predominantly from the *J* = 8 ground multiplet, and there is no difference expressing this in either basis as *J* = 8 can only be obtained by “ferromagnetic” coupling of all angular momenta. However, the next lowest-lying pseudodoublet at 2713 cm^–1^ arises from the *J* = 7 multiplet and can be decomposed as either 78%|3,7, ± 7⟩ + 16%|2,7, ±7⟩ in the |*S*_tot_, *J*_tot_, *m*_*J*_⟩ basis or 48%|13/2,7, ±7⟩ + 46%|15/2,7, ±7⟩ in the |*J*_4*f*_, *J*_tot_, *m*_*J*_⟩ basis, showing that the *L*–*S* scheme is a better description. For **3-Tb**, the lowest seven doublets arise predominantly from the *J* = 13/2 ground multiplet; however, in this case, there in non-negligible mixing from the *J* = 11/2 multiplet, and neither choice of basis is obviously the better description (although we have found that |Ω| > |λ|, which in a simplistic picture suggests an *L*–*S* description). Clearly, in the presence of significant CF effects, the reductive assignment of a coupling scheme is not particularly beneficial.

Comparing the CASSCF-SO calculated electronic states to dynamic magnetic measurements, **1-Dy** appears to show Orbach relaxation over the third excited doublet at 847 K, *cf. U*_eff_ = 817(10) K. This SMM metric is far superior for **1-Dy** compared to that for the closely related [Dy(Cp^ttt^)_2_Cl] (*U*_eff_ = 56 K, τ_0_ = 5.1 × 10^–6^ s).^40^ There is no quantifiable Orbach region for **2-Dy**, which appears to be due to more efficient Raman relaxation in this compound, and we note that the Raman exponent *n* is larger for both **1-Dy** and **2-Dy** than for the corresponding **1-Tb** and **2-Tb** compounds, explaining why the Orbach region is observed for **2-Tb** but not **2-Dy**. However, we cannot yet explain why the Dy(III) analogues show larger Raman exponents than their Tb(III) counterparts. The poor SMM properties of **2-Dy** contrast with dysprosocenium complexes, [Dy(Cp^R^)_2_][B(C_6_F_5_)_4_], which exhibit large *U*_eff_ and *T*_H_.^31−35^ The measured *U*_eff_ for **1-Tb** appears to coincide with the first excited pseudodoublet (450 K *vs U*_eff_ = 483(232) K), whereas that for **2-Tb** lies between the first and second excited pseudodoublets (563 and 1082 K vs *U*_eff_ = 714(154) K) and is the second-highest *U*_eff_ reported to date for any mononuclear Tb(III) SMM (*cf*. *U*_eff_ = 938 K for [Tb{(O-(C_6_H_4_)-*p*-^t^Bu)_8_Pc}(Pc)], Pc = phthalocyanine).^70^ Compared to the similar terbocenium complexes, [Tb(Cp^R^)_2_][B(C_6_F_5_)_4_],^20,71^ which do not have measurable energy barriers to magnetic reversal, **2-Tb** is much improved. The measured *U*_eff_ values for **3-Ln** (1920(91) K for **3-Tb** and 1964(48) K for **3-Dy**) are higher than any previously reported mononuclear Ln(II) SMMs, where the previous mononuclear Ln(II) record *U*_eff_ was held by [Tb(C_5_^i^Pr_5_)_2_] (1738 K), whereas [Dy(C_5_^i^Pr_5_)_2_] showed no obvious Orbach process.^20^ Using our CASSCF/CASPT2-SO-calculated electronic structures, we can build diagrams representing the magnetic relation barriers for **3-Tb** and **3-Dy** (Figures S159 – S162). The lowest-lying multiplet for **3-Dy** is *J* = 8, and the splitting pattern shows that the lowest-lying states with small < *J*_*z*_ > projections are the third-excited pseudodoublets that lie at 1215 or ca. 1380 cm^–1^ (ca. 1750 and 1990 K, for CASSCF-SO and CASPT2-SO, respectively), the latter being in excellent agreement with the observed *U*_eff_ barrier. Although there are differences in the description of the eigenstates for **3-Tb** whether we use the *L*–*S* or *J*–*s* basis, both show that the low-lying multiplet is dominated by *J* = 13/2 with substantial *J* = 11/2 mixing (Tables S39 and S40). The barrier for **3-Tb** appears quite linear (Figures S159 and S160), in contrast to the often parabolic-like barriers observed for Dy(III) SMMs,^31^ and is reminiscent of that calculated for [Tb(C_5_^i^Pr_5_)_2_].^63^ The results for CASSCF-SO and CASPT2-SO are very similar, and both show a total splitting of the *J* = 13/2 multiplet of ∼1900 cm^–1^ (2730 K), which is far larger than the observed *U*_eff_ barrier. This suggests that there is efficient thermally assisted QTM around the third or fourth excited doublet, which is where the mixing between the *J* = 13/2 and *J* = 11/2 becomes substantial. The high *U*_eff_ values for **3-Ln** can be attributed to the Piso ligands being more charge-dense than Cp^R^, giving rise to a stronger axial CF. However, the magnetic hysteresis loops for [Tb(C_5_^i^Pr_5_)_2_] and [Dy(C_5_^i^Pr_5_)_2_] remain open in the important zero-field region up to 55 and 10 K, respectively,^20^ whereas for **3-Tb** and **3-Dy**, they are already closed at zero field by 2 K. Similarly, for both **1-Tb** and **2-Tb**, there is no open hysteresis at 2 K observed, which likely arises from the non-Kramers nature of Tb(III). Similar observations have been made for the terbocenium complexes [Tb(Cp^ttt^)_2_][B(C_6_F_5_)_4_]^71^ and [Tb(C_5_^i^Pr_5_)_2_][B(C_6_F_5_)_4_]^20^ and other Tb(III) SMMs with large energy barriers, with Tb(III) substituted bis-phthalocyanine complexes achieving *U*_eff_ values up to 938 K.^27−29,72,73^ Only complexes **1-Dy** and **2-Dy** show open hysteresis loops, up to 10 and 4.5 K, respectively, but these are still far below the temperatures observed for the [Dy(Cp^R^)_2_][B(C_6_F_5_)_4_] family (up to 80 K)^31−35^ or related heteroatom-substituted Dy(III) metallocenes.^36−39^ This can be attributed to faster Raman and/or QTM relaxation in **3-Ln** and **2-Ln**, which we suspect arises from the transverse fields imposed by the off-axis N-donor atoms and possibly the C-*p*π and N-*p*π orbitals,^44^ leading to stronger mixing of *m*_*J*_ states, along with more low-lying intramolecular vibrational modes involving the first coordination sphere as compared to the rigid cyclopentadienyl rings in [Ln(C_5_^i^Pr_5_)_2_].^18,20^

## Conclusions

We have reported straightforward synthetic routes to the first Tb(II) and Dy(II) amidinate complexes, [Ln(Piso)_2_]. The relatively high thermal stability of these complexes can be attributed to the near-linear C···Ln···C angles set between the Ln(II) centers and the CN_2_ backbones of the two amidinate ligands, together with the four N-donor atoms being positioned between tetrahedral and square planar geometries. The arrangement of ligands in [Ln(Piso)_2_] imposes strong axial crystal fields, leading to interesting electronic structures and magnetic properties. The combination of XRD, SQUID, UV–vis-NIR, and EPR spectroscopic data consistently shows that these complexes formally exhibit 4f^*n*^5d_*z*2_^1^ valence electron configurations. The effective barriers to magnetic reversal shown by [Ln(Piso)_2_] provide new evidence for Ln(II) SMMs. However, we suspect that transverse fields introduced by the donation of electron density from off-axis N-donor atoms and the π-orbitals of CN_2_ backbones result in considerable mixing of *m*_*J*_ states and fast magnetic relaxation in zero field. The physical properties of [Ln(Piso)_2_] are best compared with the Ln(II) metallocenes [Ln(C_5_^i^Pr_5_)_2_] for Ln = Tb and Dy,^20^ which also show strong axial ligand fields and 4f^*n*^5d^1^ configurations. However, differences between the ligand fields in these complexes provide some divergent properties, e.g., a high *U*_eff_ value for [Dy(Piso)_2_] vs no barrier observed for [Dy(C_5_^i^Pr_5_)_2_], and no magnetic hysteresis above 2 K was seen for [Tb(Piso)_2_] at zero field vs open loops for [Tb(C_5_^i^Pr_5_)_2_] up to 55 K.^20^ The faster Raman relaxation in [Tb(Piso)_2_] vs [Tb(C_5_^i^Pr_5_)_2_] could be linked to the expected reduced rigidity of Piso compared to the Cp^R^ ring. These disparities reiterate that predicting and understanding the magnetic properties of Ln(II) complexes with formal 4f^*n*^5d^1^ valence electron configurations are currently nontrivial. These results emphasize that ligand donor properties and complex geometries are of equal importance in dictating both the thermal stability and SMM properties of nontraditional Ln(II) complexes. Given the large number of multidentate N-donor ligands with delocalized π-systems available to synthetic chemists, we anticipate that future studies of these systems should provide an extended family of thermostable Ln(II) SMMs and could also deliver new insights into the relationship between the lanthanide ligand field, electronic structure, and magnetic properties.

## Experimental Section

### General Methods

Dry toluene and pentane were obtained from a solvent purification system, where they were passed through columns containing alumina and molecular sieves and then stored over K mirrors. Benzene and hexane were dried by refluxing over potassium and were stored over K mirrors following distillation. Dichloromethane (DCM) was dried over CaH_2_ and was stored over 3 Å molecular sieves. All solvents were degassed before use. For NMR spectroscopy, C_6_D_6_ was dried by refluxing over K, and CD_2_Cl_2_ was dried by refluxing over CaH_2_. NMR solvents were vacuum transferred and degassed by three freeze–pump–thaw cycles before use. All experiments were performed under an atmosphere of dry argon with the rigid exclusion of air and moisture using standard Schlenk and glovebox techniques. ^1^H (400 MHz) and ^13^C{^1^H} (126 MHz), ^11^B{^1^H} (128 MHz) and ^19^F{^1^H} (376 MHz) NMR spectra were obtained on an Avance III 400 MHz spectrometer at 298 K. UV–vis-NIR spectroscopy was performed on samples in Youngs tap-appended 10 mm path length quartz cuvettes on an Agilent Technologies Cary Series UV–vis-NIR spectrophotometer from 175 to 3300 nm. ATR-IR spectra were recorded as microcrystalline powders by using a Bruker Alpha II spectrometer. Elemental analysis experiments were performed on a Flash 2000 elemental analyzer at the Microanalytical Service, Department of Chemistry, the University of Manchester. ICP-MS experiments were performed on an Agilent 8900 Triple Quadrupole ICP-MS housed in a class 1000 cleanroom, with sample preparation performed in a class 100 cleanroom. In each experiment, *ca*. 5–15 mg of sample was digested in 3 mL of concentrated HNO_3(aq)_ by heating at 95 °C for 2 h. After acid digestion, 2000× dilution was performed with 2% HNO_3(aq)_ to achieve a final concentration in the solution of *ca*. 1 ppm.

### Single-Crystal XRD

The crystal data for **1-Ln**, **2-Ln**, and **3-Ln** are compiled in Tables S1 and S2. Crystals of **1-Dy** and **1-Tb** were collected on an Oxford Diffraction SuperNova Atlas CCD diffractometer using mirror-monochromated Mo Kα radiation (λ = 0.71073 Å); **3-Dy** was collected on a Rigaku FR-X diffractometer equipped with a Hypix 6000HE photon counting pixel array detector with mirror-monochromated Cu Kα (λ = 1.5418 Å) radiation; and **2-Dy**, **2-Tb**, and **3-Tb** were collected on a Rigaku XtaLAB Synergy-DW VHF equipped with a HyPix-6000HE photon counting pixel array detector with mirror-monochromated Cu Kα (λ = 1.5418 Å) radiation. All data collections were performed at 100 K. The structures were solved by direct methods and were refined by full-matrix least-squares on all unique *F*^2^ values, with anisotropic displacement parameters for all non-hydrogen atoms and with constrained riding hydrogen geometries. CrysAlisPro^74^ was used for control and integration, and SHELXT^75,76^ was employed through OLEX2^77^ for structure solution and refinement. DIAMOND was used for crystallographic images.^78^

### Powder XRD

Microcrystalline samples of **1-Ln**, **2-Ln**, and **3-Ln** were mounted on a goniometer head with a micromount using a minimum amount of Fomblin. X-ray diffraction data were collected at 100 K using a Rigaku FR-X rotating anode single-crystal X-ray diffractometer with Cu Kα radiation (λ = 1.5418 Å), a Hypix-6000HE detector, and an Oxford Cryosystems nitrogen flow gas system. The instrument was calibrated using silver behenate as standard. Data were collected between 3 and 70° 2θ, with a detector distance of 150 mm and a beam divergence of 1.5 mrad.^79^ CrysAlisPro^74^ was used to collect, reduce, and integrate XRD data. Pawley and Rietveld refinement was performed using the TOPAS software, with unit cells and structures obtained from single-crystal XRD analysis.^80−82^

### Magnetic Measurements

Magnetic measurements were performed on a Quantum Design MPMS3 superconducting quantum interference device (SQUID) magnetometer. Samples of **1-Dy** (28 mg), **2-Dy** (27.1 mg), **3-Dy** (25.8 mg), **1-Tb** (29.1 mg), **2-Tb** (24.2 mg), and **3-Tb** (27.4 mg) were ground to powders, loaded in borosilicate glass NMR tubes, and covered in ground eicosane (19, 12.3, 9.8, 14.3, 10.2, and 11.1 mg, respectively) in a glovebox. The tubes were removed from the glovebox, kept under a protected atmosphere, and transferred onto a Schlenk line, where the eicosane powder was melted under an argon atmosphere. The tubes were flame-sealed to ∼3 cm under reduced pressure. Kapton tape was wrapped around the top of the tubes to hold them inside plastic straws by friction, and the straws were attached to the end of the sample rod. The raw data were corrected for the diamagnetic contribution of the sample holder and eicosane using calibrated blanks. The data were corrected for the sample shape using the Quantum Design MPMS3 Geometry Simulator, assuming a uniform cylinder of 4.06 mm diameter and 3.58 mm height. Finally, the data were corrected for the intrinsic diamagnetic contribution of the sample, estimated as the molecular weight (g/mol) multiplied by 0.5 × 10^–6^ cm^3^ mol^–1^ K. Further details of measurements are compiled in the Supporting Information.

### EPR Spectroscopy

Continuous wave EPR spectra were recorded on a Bruker EMX300 spectrometer operating at the X-band (*ca*. 9.4 GHz) and on a Bruker ElexSys E580E SQFT spectrometer operating at the S-band frequency (*ca*. 3.8 GHz). Modulation amplitudes of 5 G were used at microwave powers of 2–20 mW. Spectra were recorded for samples of **3-Tb**, **3-Dy**, **2-Tb**, and **2-Dy** in the crystalline phase and of **1-Tb**, **1-Dy**, **3-Tb**, and **3-Dy** in frozen toluene/hexane (90:10) solutions (5 mM for **1-Tb**, **1-Dy**; 0.5 mM for **3-Tb**, **3-Dy**). Samples were prepared in quartz tubes under an inert atmosphere and flame-sealed.

### CASSCF Calculations

CASSCF(-MSCASPT2)-SO calculations were carried out using the OpenMolcas program,^62^ version 21.06. All structures were taken from XRD without optimization. The basis sets were chosen from the ANO-RCC library:^83,84^ VTZP quality for lanthanide ions, VDZP for the nitrogen atoms located in the first coordination sphere, and VDZ for the rest of atoms. Cholesky decomposition of the two-election integrals was employed to reduce the computational demand with a threshold of 10^–8^. For Tb(III) calculations, we considered 7 septets, 140 quintets, 588 triplets, and 490 singlets in the orbital optimization and CI step, whereas 7 septets, 140 quintets, 195 triplets, and 197 singlets were mixed by spin–orbit coupling. For Dy(III) calculations, we considered 21 sextets, 224 quartets, and 490 doublets in the orbital optimization and CI step, whereas 21 sextets, 128 quartets, and 130 doublets were mixed by spin–orbit coupling. For Tb(II) calculations, we considered 7 octets and 7 sextets, and for Dy(II), we considered 18 septets and 18 quintets, in all steps. MSCASPT2 calculations were performed using an imaginary shift of 0.1 au. Projection of model Hamiltonian parameters was performed using molcas_suite,^85^ and construction of model Hamiltonians was performed using angmom_suite^86^ and PHI.^87^

### Synthesis

Anhydrous TbI_3_ was purchased from Alfa Aesar and used as received. Anhydrous DyI_3_,^52^ KPiso,^53^ and [H(SiEt_3_)_2_][B(C_6_F_5_)_4_]^54,55^ were prepared according to literature methods.

#### [Tb(Piso)_2_I]·C_7_H_8_ (**1-Tb**)

TbI_3_ (1.080 g, 2.0 mmol) and KPiso (1.832 g, 4.0 mmol) were placed in a 200 mL Rotaflo stopcock-appended flask containing a glass-coated magnetic stirring bar. Toluene (50 mL) was added, and the reaction was heated gradually to 150 °C with vigorous stirring. After 5 days at 150 °C, the reaction mixture was allowed to cool to room temperature, filtered, and concentrated under a vacuum until a white precipitate formed (∼10 mL). The precipitate was redissolved by briefly heating the mixture to 100 °C. The flask was wrapped with Al foil and allowed to cool slowly to room temperature overnight to give colorless crystals of **1-Tb** (crystal size ∼0.3 × 0.2 × 0.2 mm), which were isolated by filtration, washed with cold pentane (2 × 5 mL), and dried under a vacuum (1.46 g, 65%). Anal. Calcd for C_61.5_H_90_IN_4_Tb (**[Tb(Piso)**_**2**_**I]·0.5C**_**7**_**H**_**8**_): C, 63.07; H, 7.75; N, 4.78. Found: C, 62.42; H, 8.32; N, 5.01. ^1^H and ^13^C{^1^H} NMR spectra could not be interpreted. ATR-IR (microcrystalline, cm^–1^) ν̃ : 3059 (w), 2963 (s), 2932 (w), 2873 (w), 1460 (m), 1429 (m), 1396 (m), 1339 (s), 1305 (m), 1239 (w), 1204 (m), 1163 (m), 1100 (w), 1042 (w), 946 (m), 797 (m), 756 (s), 731 (m), 693 (w), 634 (w), 582 (w), 548 (w).

#### [Dy(Piso)_2_I]·C_7_H_8_ (1-Dy-I) and [Dy(Piso)_2_I] (**1-Dy-II**)

DyI_3_ (1.090 g, 2.0 mmol) and KPiso (1.832 g, 4.0 mmol) were placed in a 200 mL Rotaflo stopcock-appended flask containing a glass-coated magnetic stirring bar. Toluene (50 mL) was added, and the reaction was heated gradually to 150 °C with vigorous stirring. After 5 days at 150 °C, the reaction mixture was allowed to cool to room temperature, filtered, and concentrated under a vacuum until a white precipitate formed (∼10 mL). The precipitate was redissolved by briefly heating the mixture to 100 °C. The flask was wrapped with Al foil and allowed to cool slowly to room temperature overnight to give colorless crystals (crystal size ∼0.2 × 0.2 × 0.1 mm), which were confirmed to be the mixture of **1-Dy-I** and **1-Dy-II**. The crystals were isolated by filtration, washed with cold pentane (2 × 5 mL) and dried under a vacuum (0.81 g, 36%). Anal. Calcd for C_65_H_94_DyIN_4_ (**[Dy(Piso)**_**2**_**I]·C**_**7**_**H**_**8**_): C, 63.94; H, 7.76; N, 4.59. Found: C, 64.07; H, 7.86; N, 4.61. ^1^H and ^13^C{^1^H} NMR spectra could not be interpreted. ATR-IR (microcrystalline, cm^–1^) ν̃ : 3063 (w), 2957 (s), 2871 (m), 1618 (s), 1578 (m), 1458 (s), 1432 (s), 1392 (m), 1328 (s), 1247 (w), 1203 (w), 1170 (m), 1104 (w), 1048 (w), 935 (m), 802 (m), 757 (s), 715 (w).

#### [Tb(Piso)_2_][B(C_6_F_5_)_4_] (**2-Tb**)

A solution of [H(SiEt_3_)_2_][B(C_6_F_5_)_4_] (0.454 g, 0.5 mmol) in benzene (10 mL) was added dropwise to a stirred solution of **1-Tb** (0.562 g, 0.5 mmol) in benzene (15 mL) at room temperature. The colorless reaction mixture was stirred for 48 h, forming a white precipitate. The volatiles were removed under a vacuum to give a white powder, which was washed with hexane (10 mL) and benzene (10 mL). The crude material was dissolved in DCM (5 mL), and the solution was layered with pentane (10 mL). Storage at room temperature overnight gave **2-Tb** as colorless crystals (crystal size ∼0.3 × 0.2 × 0.1 mm). (0.253 g, 30%). Anal. Calcd for C_82_H_86_BF_20_N_4_Tb: C, 58.72; H, 5.17; N, 3.34. Found: C, 57.91; H, 5.17; N, 3.42. ^1^H and ^13^C{^1^H} NMR spectra could not be interpreted. ^11^B{^1^H} NMR (C_6_D_6_, 128 MHz): δ −14.07 (br). ^19^F{^1^H} NMR (C_6_D_6_, 376 MHz): δ −130.58 (br), −161.04 (br), −165.03 (br). ATR-IR (microcrystalline, cm^–1^) ν̃ : 2965 (m), 2871 (w), 1642 (m), 1509 (m), 1461 (s), 1344 (m), 1274 (w), 1207 (w), 1167 (w), 1082 (s), 975 (s), 800 (m), 760 (s), 725 (w), 685 (m), 664 (m), 607 (w), 573 (w).

#### [Dy(Piso)_2_][B(C_6_F_5_)_4_] (**2-Dy**)

A solution of [H(SiEt_3_)_2_][B(C_6_F_5_)_4_] (0.454 g, 0.5 mmol) in benzene (10 mL) was added dropwise to a stirred solution of **1-Dy** (0.564 g, 0.5 mmol) in benzene (15 mL) at room temperature. The colorless reaction mixture was stirred for 48 h, forming a white precipitate. The volatiles were removed under a vacuum to give a white powder, which was washed with hexane (10 mL) and benzene (10 mL). The crude material was dissolved in DCM (5 mL) and layered with pentane (10 mL). Storage at room temperature overnight gave **2-Dy** as colorless crystals (crystal size of ∼0.3 × 0.2 × 0.1 mm) (0.269 g, 32%). Anal. Calcd for C_82_H_86_BDyF_20_N_4_: C, 58.59; H, 5.16; N, 3.33. Found: C, 56.76; H, 5.00; N, 3.25. ^1^H and ^13^C{^1^H} NMR spectra could not be interpreted. ^11^B{^1^H} NMR (C_6_D_6_, 128 MHz): δ −22.22 (br). ^19^F{^1^H} NMR (C_6_D_6_, 376 MHz): δ −137.82 (br), −138.88 (br), −173.17 (br). ATR-IR (microcrystalline, cm^–1^) ν ∼ : 2972 (m), 2871 (w), 1642 (m), 1514 (m), 1461 (s), 1394 (w), 1336 (s), 1269 (w), 1207 (w), 1165 (m), 1084 (m), 975 (s), 805 (m), 760 (s), 720 (w), 682 (m), 665 (m), 613 (w), 571 (w).

#### [Tb(Piso)_2_]·C_5_H_12_ (**3-Tb**)

A solution of **1-Tb** (1.125 g, 1 mmol) in benzene (40 mL) was added dropwise to a stirred solution of KC_8_ (0.149 g, 1.1 mmol) in benzene (20 mL) at room temperature. The reaction mixture was allowed to stir at 25 °C for 6 days (alternatively, the reaction can be expedited by heating at 60 °C for 3 days); the color gradually changed from colorless to green, and bronze KC_8_ slowly converted to black graphite. The solution was allowed to settle overnight and filtered to another flask. The solvent was removed under a vacuum, and the residue was extracted by stirring with hexane (25 mL) for 15 min. The solution was filtered, and hexane was removed under a vacuum. The green crude product was dissolved in 10 mL of pentane, concentrated to ∼3 mL under a vacuum, and stored at −35 °C. Orange crystals of **3-Tb** formed after several days (crystal size ∼0.3 × 0.2 × 0.2 mm) (0.234 g, 22%). Anal. Calcd for C_58_H_86_N_4_Tb: C, 69.78; H, 8.68; N, 5.61. Found: C, 66.32; H, 8.58; N, 5.09. ^1^H and ^13^C{^1^H} NMR spectra could not be interpreted. UV–vis-NIR ύ_max_/ cm^–1^: ∼14,000 (ε = 2100 M^–1^ cm^–1^), ∼ 14,000 (ε = 3050 M^–1^ cm^–1^). ATR-IR (microcrystalline, cm^–1^) ν̃ : 3060 (w), 2959 (s), 2870 (m), 1615 (m), 1588 (s), 1456 (m), 1432 (s), 1384 (s), 1357 (s), 1315 (m), 1253 (m), 1204 (m), 1170 (s), 1097 (m), 1042 (m), 949 (m), 800 (s), 756 (s), 725 (w), 793 (w).

#### [Dy(Piso)_2_]·C_5_H_12_ (**3-Dy**)

A solution of **1-Dy** (0.564 g, 0.5 mmol) in benzene (20 mL) was added dropwise to a stirred solution of KC_8_ (0.074 g, 0.55 mmol) in benzene (10 mL) at room temperature. The reaction mixture was allowed to stir at 25 °C for 6 days (alternatively, the reaction can be expedited by heating at 60 °C for 3 days); the color gradually changed from colorless to green, and bronze KC_8_ slowly converted to black graphite. The solution was allowed to settle overnight and filtered to another flask. The solvent was removed under a vacuum, and the residue was extracted by stirring with hexane (25 mL) for 15 min. The solution was filtered, and hexane was removed under a vacuum. The green crude product was dissolved in 10 mL of pentane, concentrated to ∼3 mL under a vacuum, and stored at −35 °C. Orange crystals of **3-Dy** formed after several days (crystal size ∼0.2 × 0.2 × 0.1 mm) (0.103 g, 19%). Anal. Calcd for C_58_H_86_DyN_4_: C, 69.53; H, 8.65; N, 5.59. Found: C, 66.53; H, 8.54; N, 5.20. ^1^H and ^13^C{^1^H} NMR spectra could not be interpreted. UV–vis-NIR ύ_max_/ cm^–1^: ∼14,000 (ε = 2050 M^–1^ cm^–1^), ∼14,000 (ε = 2800 M^–1^ cm^–1^). ATR-IR (microcrystalline, cm^–1^) ν̃ : 3063 (w), 3020 (w), 2957 (s), 2877 (m), 1618 (w), 1586 (w), 1429 (s), 1388 (s), 1360 (s), 1314 (m), 1239 (m), 1203 (m), 1162 (s), 1104 (w), 1040 (w), 949 (m), 800 (m), 757 (s), 710 (w), 642 (w).

## Supporting Information Available

Additional experimental details for physical and computational data associated with this manuscript (PDF)
