# Supplementary material for: Thermally Stable Terbium(II) and Dysprosium(II) Bis-amidinate Complexes
Source: J Am Chem Soc. 2023 Nov 24;145(51):27993–8009. doi: 10.1021/jacs.3c07978 (PMC10755703; doi:10.1021/jacs.3c07978)
Supplement: Supplementary file 1 — ja3c07978_si_001.pdf [file ja3c07978_si_001.pdf]

Thermally stable Terbium(II) and Dysprosium(II) Bis-Amidinate Complexes

Peng-Bo Jin,[1] Qian-Cheng Luo,[1] Gemma K. Gransbury,[2] Iñigo J. Vitorica-Yrezabal,[2] Tomáš Hajdu,[2,3] Ilya Strashnov,[1] Eric J. L. McInnes,[2,3] Richard E. P. Winpenny,[2] Nicholas F. Chilton,[2,*] David P. Mills,[2,*] and Yan-Zhen Zheng[1,*]

[1] *Frontier Institute of Science and Technology (FIST), State Key Laboratory of Electrical Insulation and Power Equipment, MOE Key Laboratory for Nonequilibrium Synthesis of Condensed Matter, Xi'an Key Laboratory of Electronic Devices and Materials Chemistry and School of Chemistry, Xi'an Jiaotong University, 99 Yanxiang Road, Xi'an, Shaanxi 710054, P. R. China.*

[2]*Department of Chemistry, The University of Manchester, Oxford Road, Manchester, M13 9PL, U.K.*

[3]*Photon Science Institute, The University of Manchester, Oxford Road, Manchester, M13 9PL, U.K.*

Contents

1. NMR spectroscopy....S3

2. IR spectroscopy....S12

3. UV-vis-NIR spectroscopy....S16

4. Single crystal X-ray diffraction....S32

5. Powder X-ray diffraction....S41

6. Magnetism....S45

6.1 Temperature-swept magnetic measurements....S46

6.2 Ac magnetic measurements....S55

6.3 Dc magnetization decays....S92

6.4 Magnetic relaxation profiles....S103

6.5 Field-swept magnetic measurements....S126

7. EPR spectroscopy....S132

8. CASSCF calculations....S135

9. ICP-MS data....S154

10. References....S155

1. NMR spectroscopy

Multinuclear NMR spectra were collected for **1-3-Ln** (Figures S1-S16). We were unable to interpret the ^{1}H and $^{13}C\{^{1}H\}$ NMR spectra obtained for samples of **1-3-Ln** (Figures S1-S4, S7-S8 and S11-S16), due to their significant paramagnetism. The $[B(C_6F_5)_4]^-$ anions in **2-Ln** were additionally characterized by $^{11}B\{^{1}H\}$ and $^{19}F\{^{1}H\}$ NMR spectroscopy, and this proved more fruitful (Figures S5-S6 and S9-S10). The $^{11}B\{^{1}H\}$ NMR spectra of both **2-Tb** (δ_B: –14.07 ppm) and **2-Dy** (δ_B: –22.22 ppm) each contain one broad signal. The $^{19}F\{^{1}H\}$ NMR spectra of **2-Tb** (δ_F: –130.58, –161.04, and –165.03 ppm) and **3-Dy** (δ_F: –137.82, –138.88, and –173.17 ppm) each exhibit three signals that correspond with the three fluorine environments for *ortho*-, *meta*- and *para*- ring substituents; these resonances are paramagnetically broadened and shifted to different extents for Tb(III) and Dy(III), as expected.

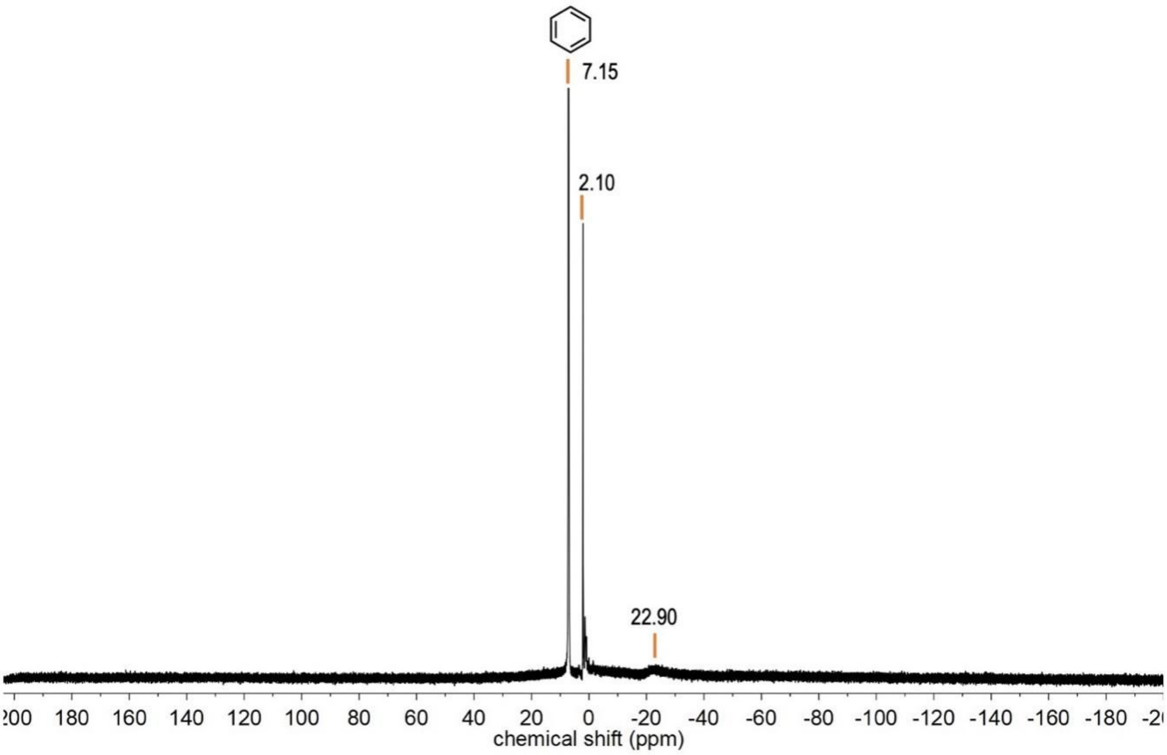

Figure S1 ^{11}H NMR spectrum of **1-Tb** in C_6D_6.

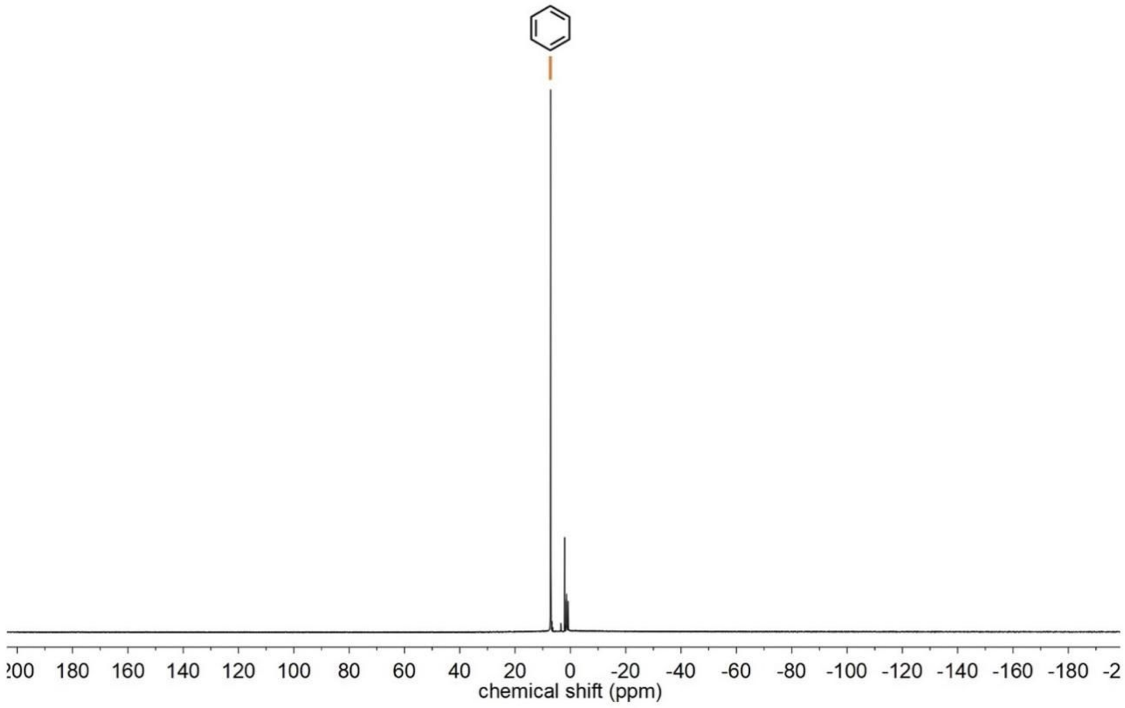

Figure S2 [11]H NMR spectrum of **1-Dy** in C_6D_6.

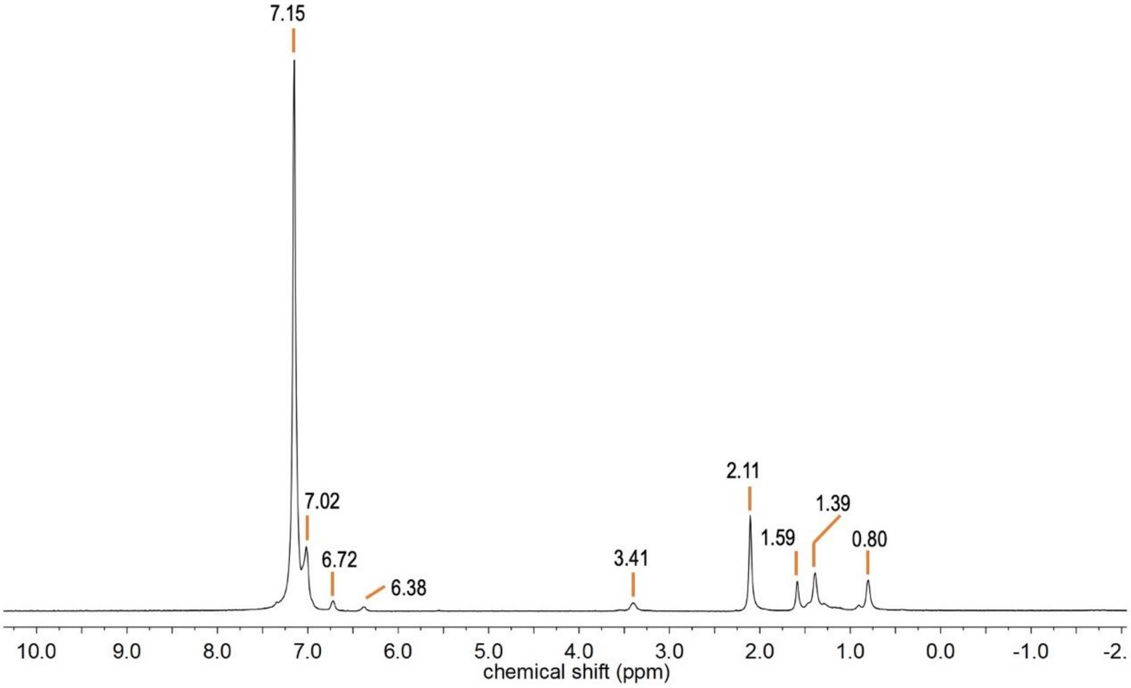

Figure S3 [11]H NMR spectrum of **1-Dy** in C_6D_6, zoomed in the region -2 to 10 ppm.

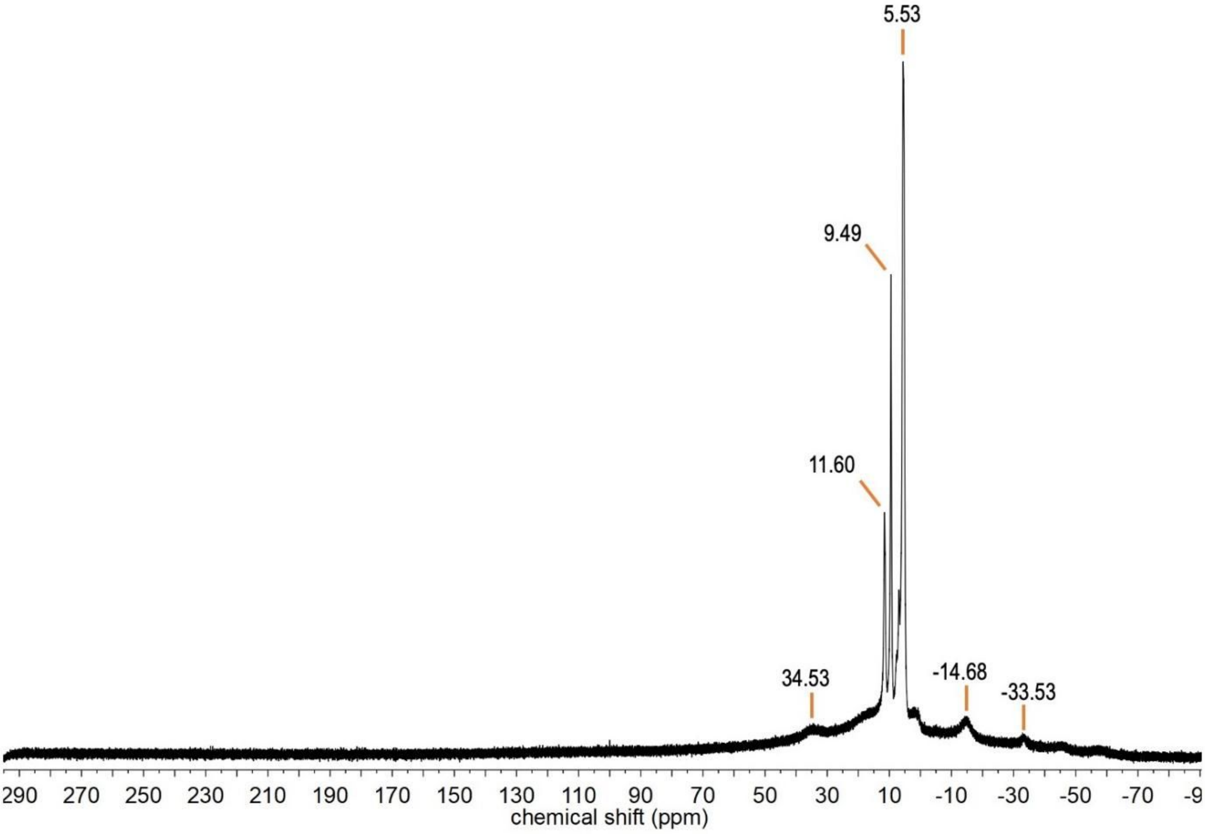

Figure S4 ^{1}H NMR spectrum of **2-Tb** in CD_2Cl_2.

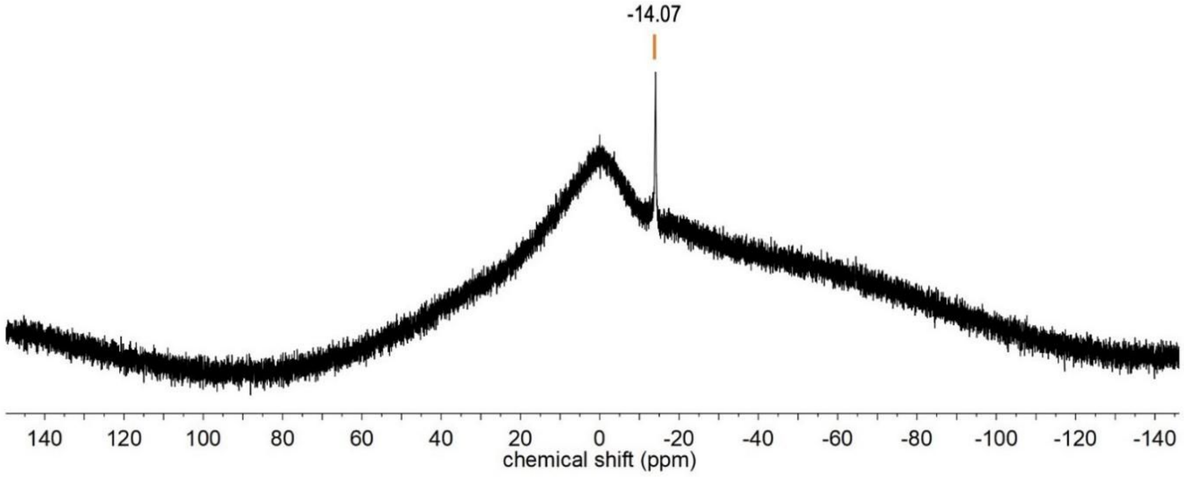

Figure S5 $^{11}B\{^{1}H\}$ NMR spectrum of **2-Tb** in CD_2Cl_2.

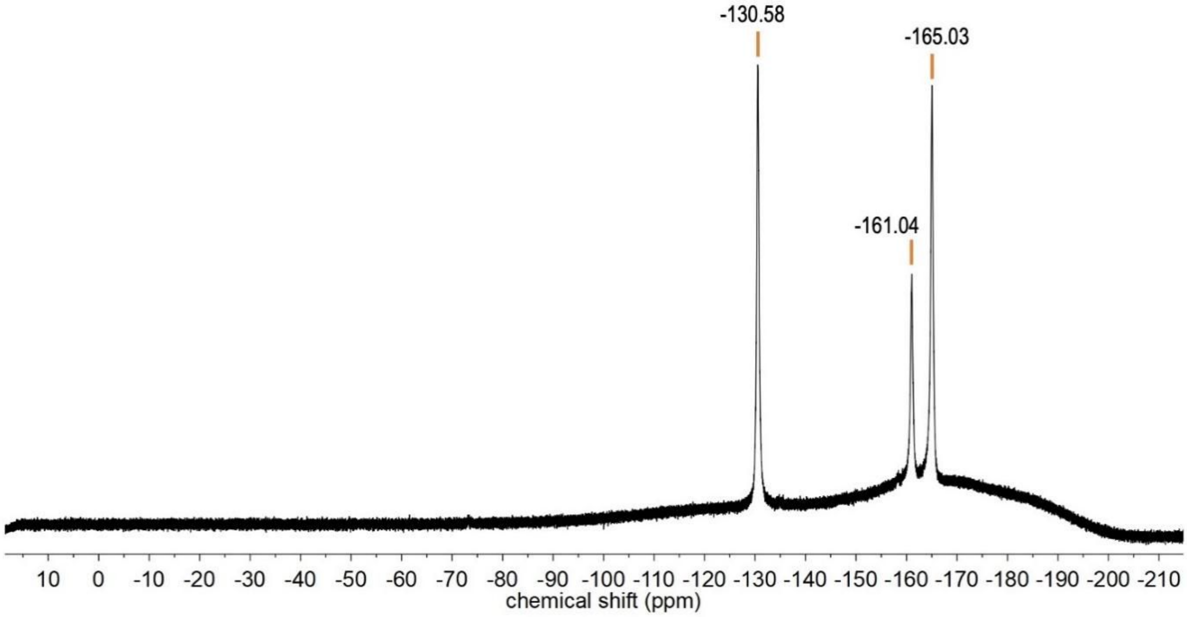

Figure S6 $^{19}F\{^{1}H\}$ NMR spectrum of **2-Tb** in CD_2Cl_2.

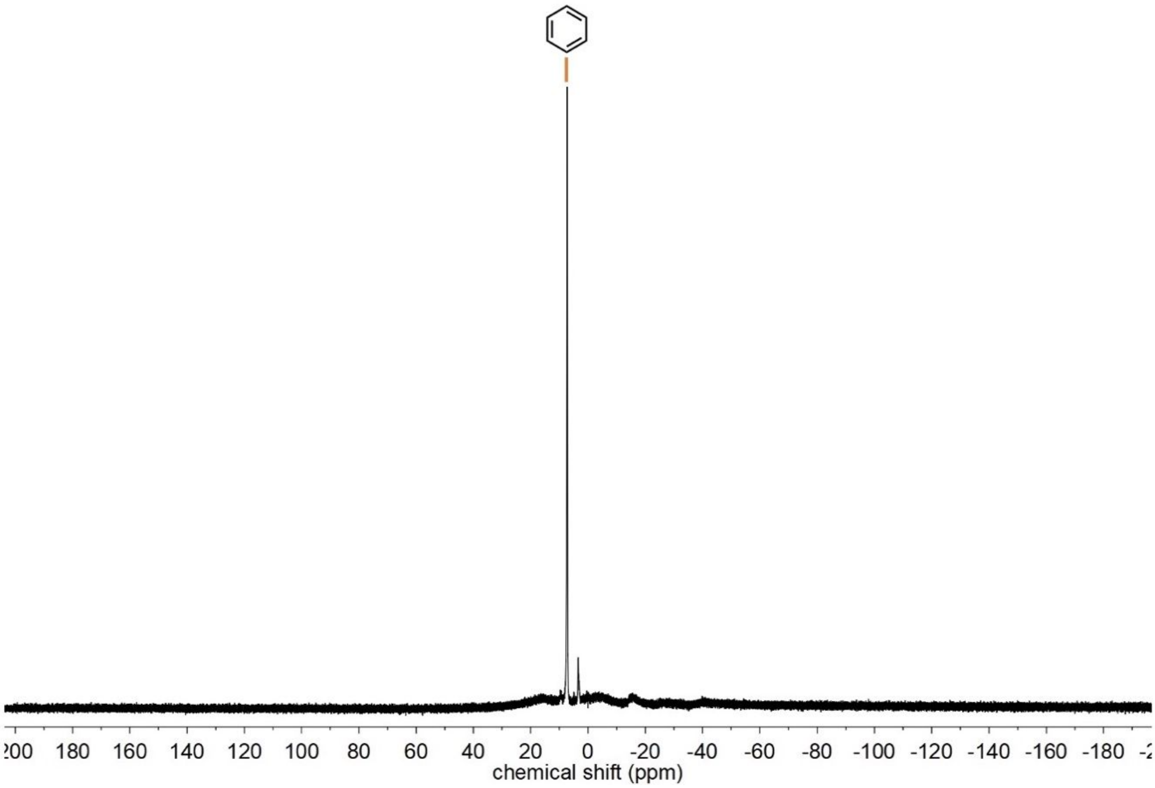

Figure S7 ^{11}H NMR spectrum of **2-Dy** in CD_2Cl_2.

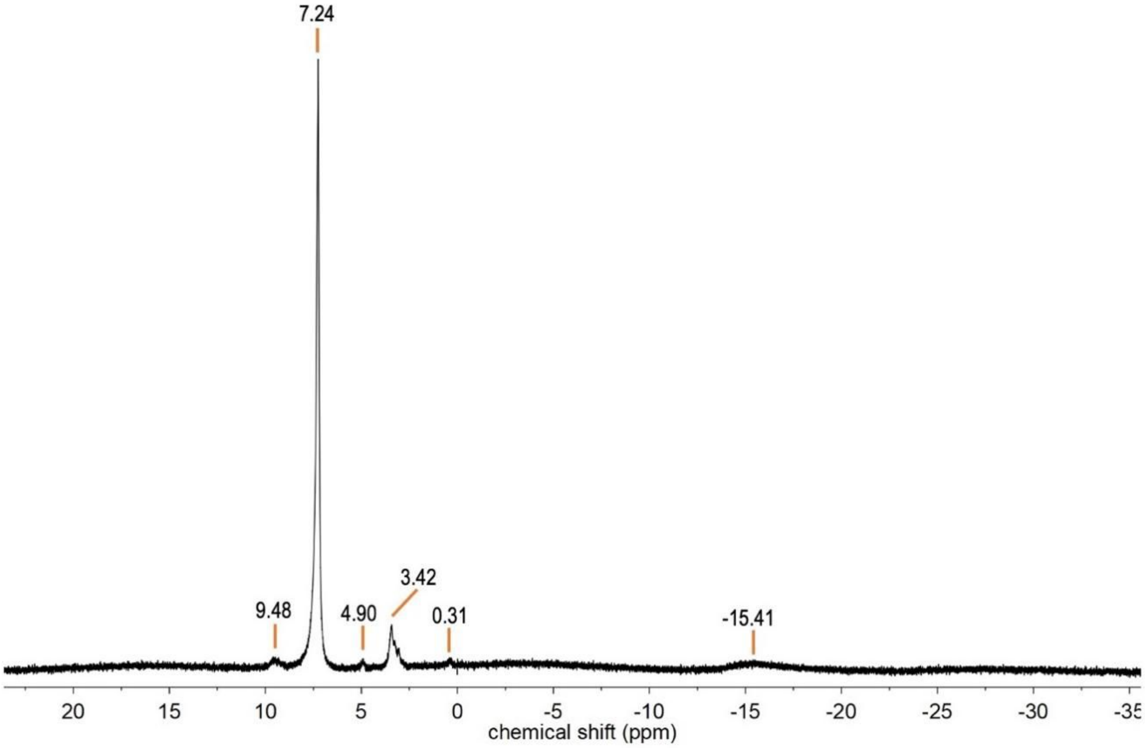

Figure S8 ^{1}H NMR spectrum of **2-Dy** in CD_2Cl_2, zoomed in the region -35 to 20 ppm.

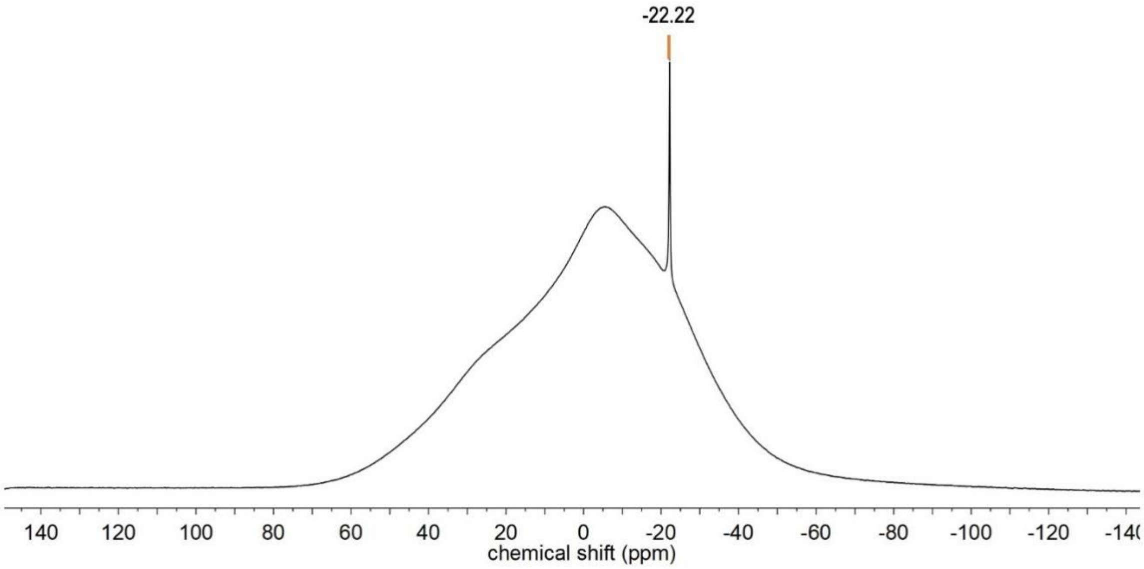

Figure S9 $^{11}B\{^{1}H\}$ NMR spectrum of **2-Dy** in CD_2Cl_2.

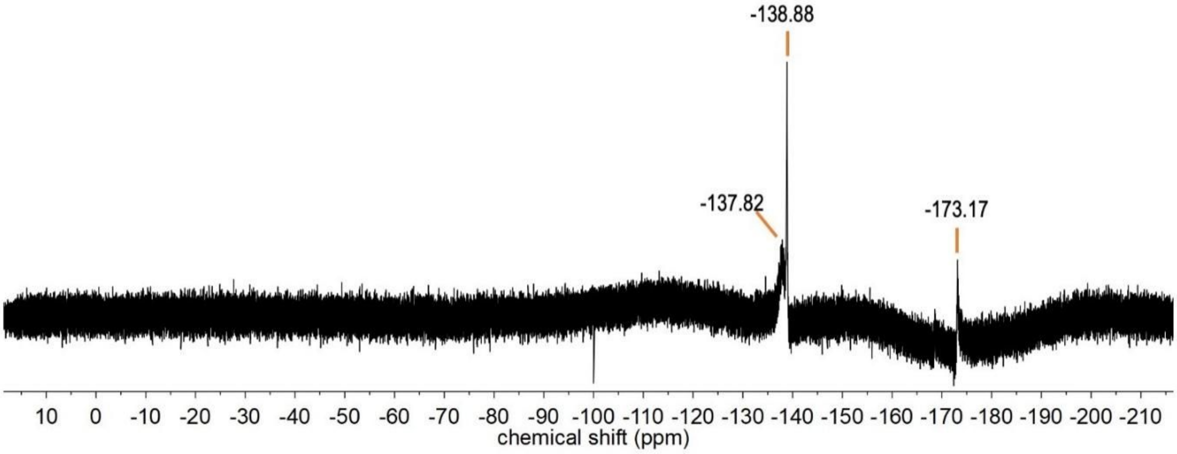

Figure S10 $^{19}F\{^{1}H\}$ NMR spectrum of **2-Dy** in CD_2Cl_2.

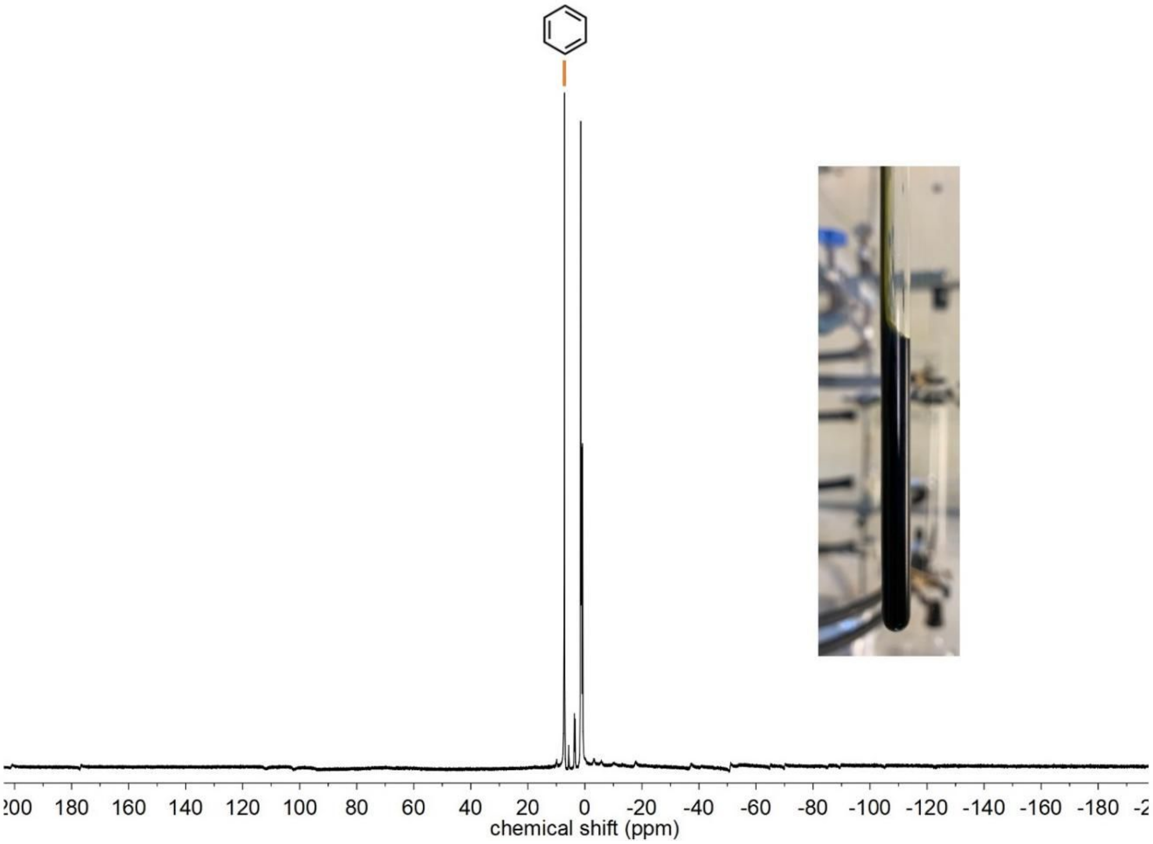

Figure S11 ^{1}H NMR spectrum of **3-Tb** in C_6D_6; Insert: A photograph of NMR sample of **3-Tb** in C_6D_6.

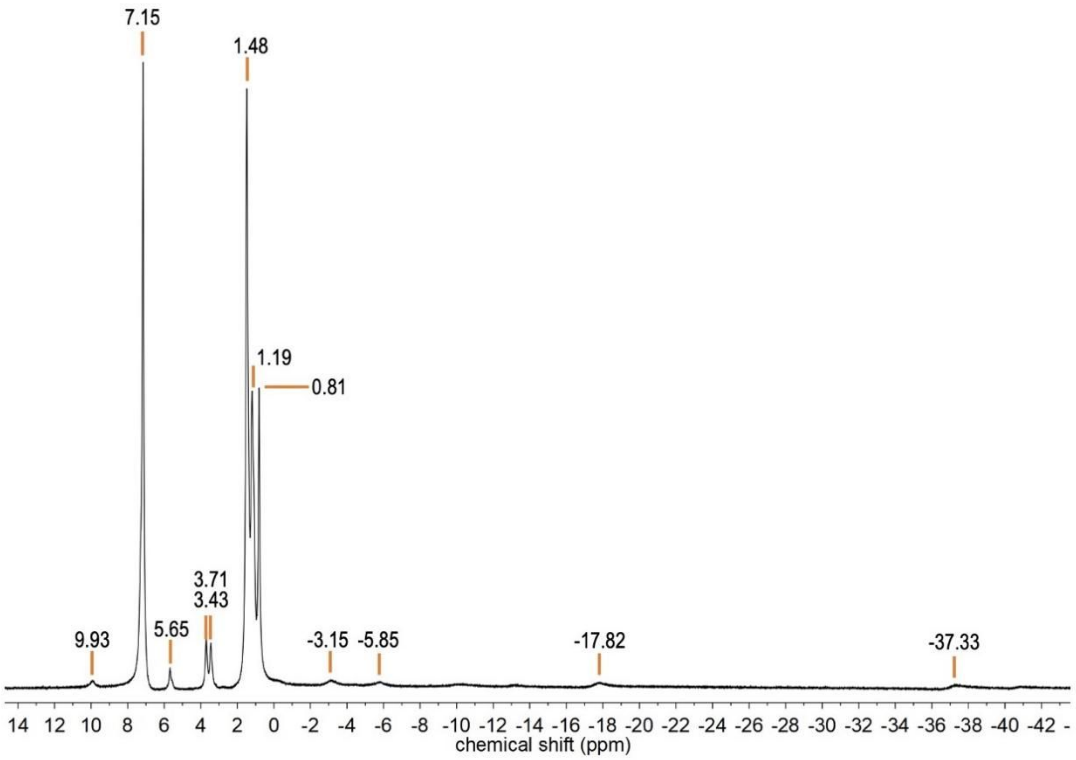

Figure S12 ^1H NMR spectrum of **3-Tb** in C_6D_6, zoomed in the region -44 to 14 ppm.

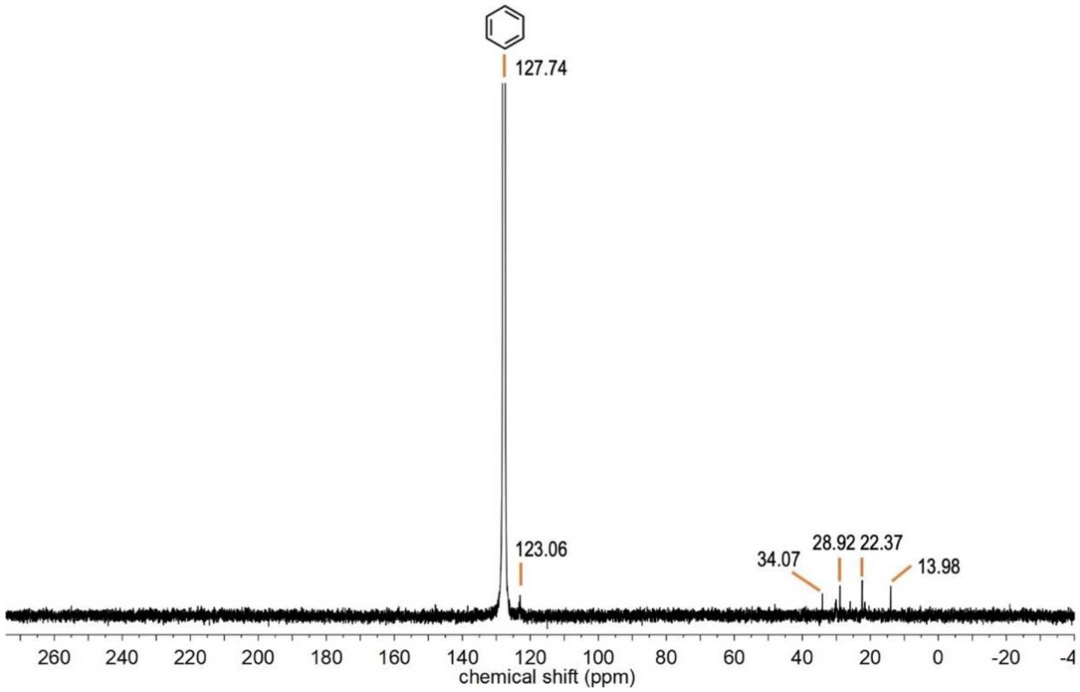

Figure S13 $^{13}C\{^1H\}$ NMR spectrum of **3-Tb** in C_6D_6.

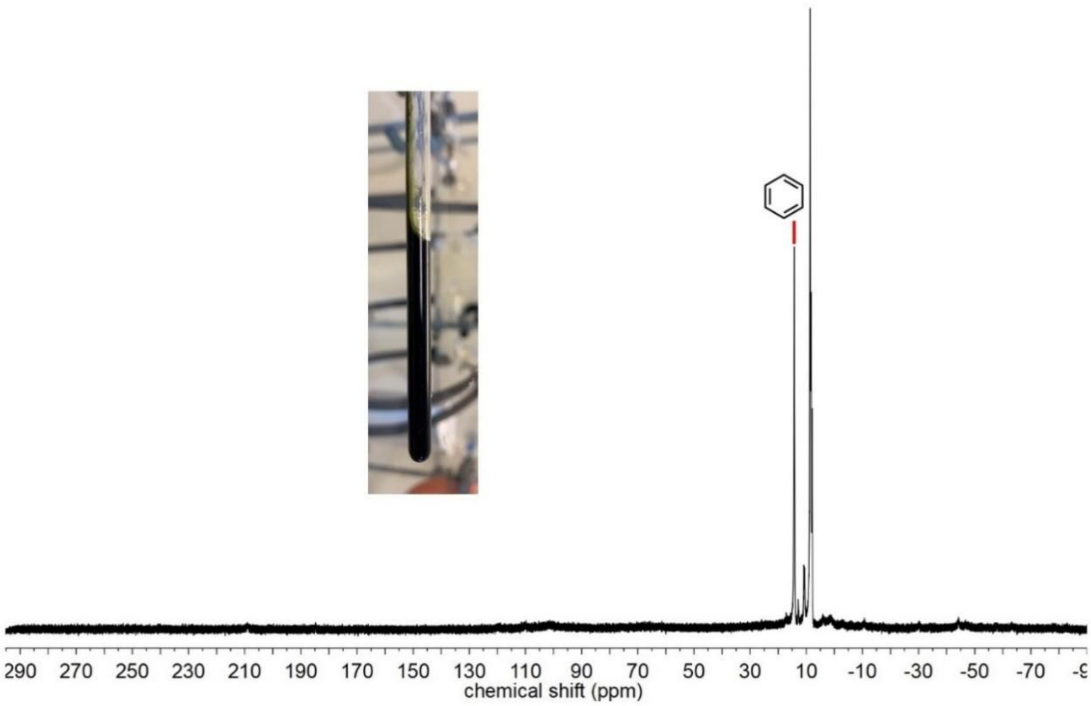

Figure S14 ^{1}H NMR spectrum of **3-Dy** in C_6D_6; Insert: A photograph of NMR sample of **3-Dy** in C_6D_6.

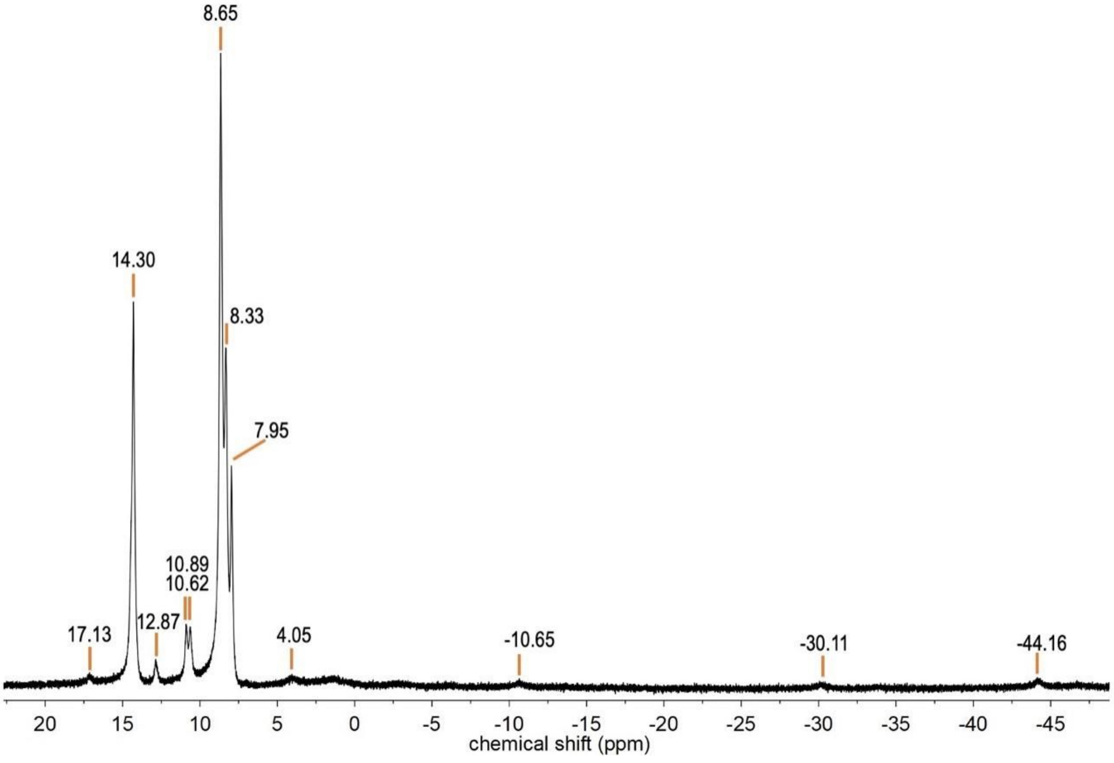

Figure S15 ^{1}H NMR spectrum of **3-Dy** in C_6D_6, zoomed in the region -45 to 20 ppm.

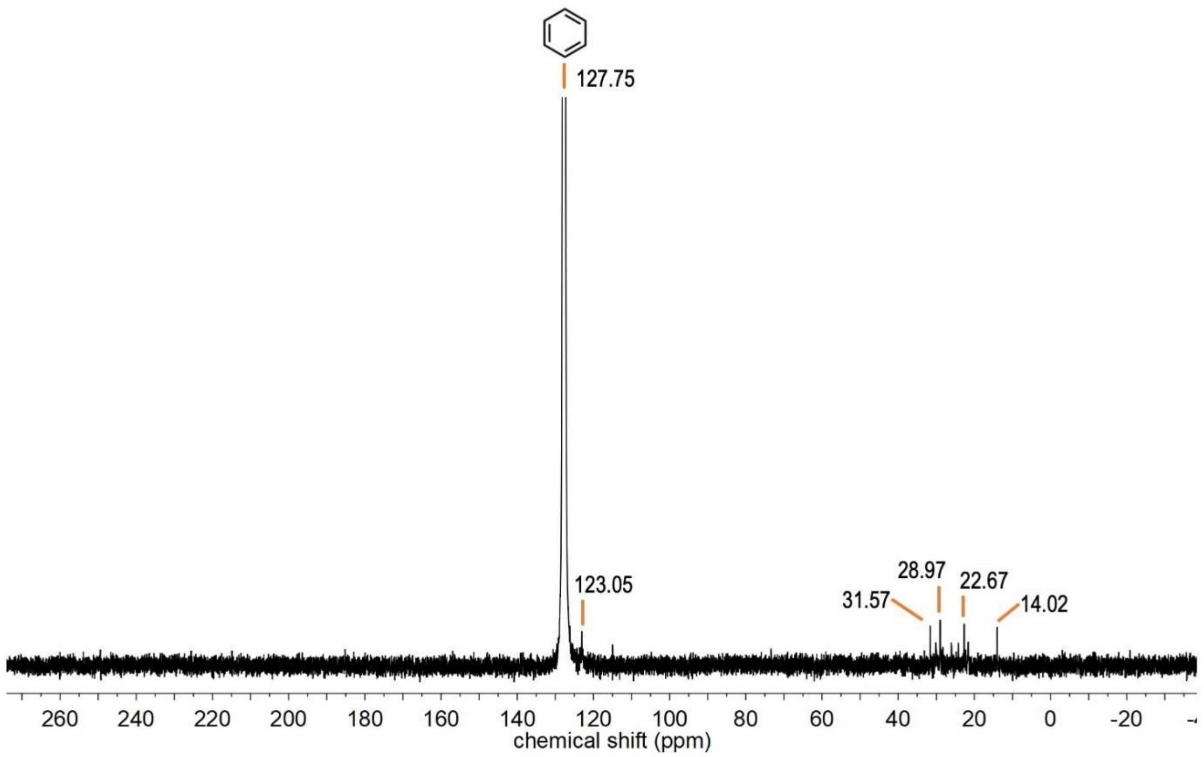

Figure S16 $^{13}C\{^1H\}$ NMR spectrum of **3-Dy** in C_6D_6.

2. IR spectroscopy

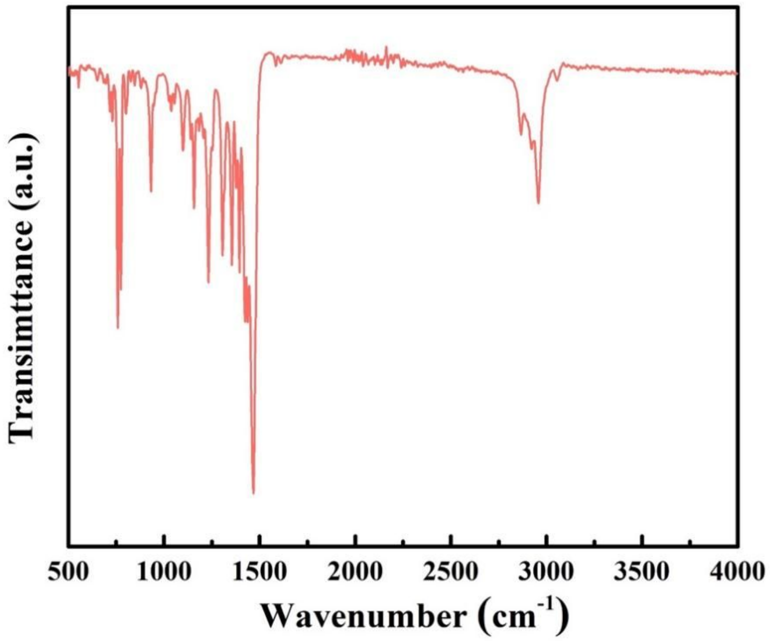

Figure S17 ATR-IR spectrum of KPiso.

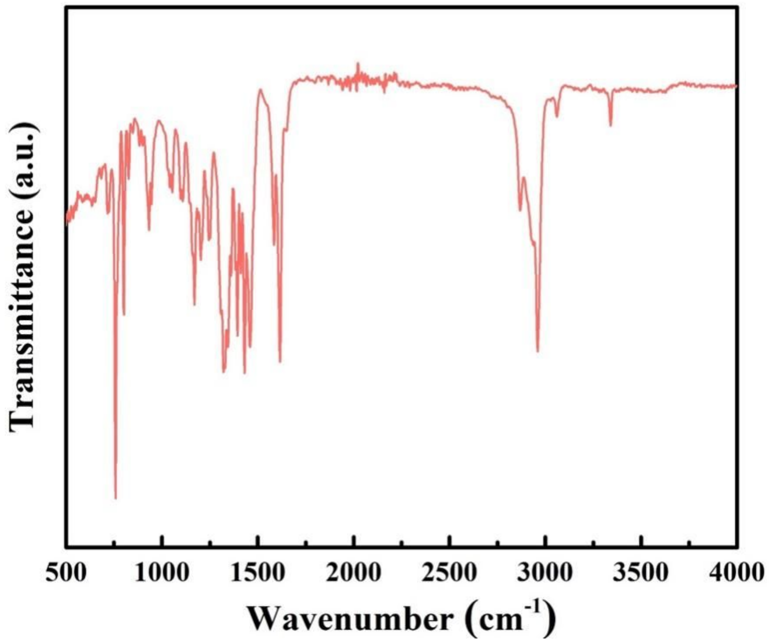

Figure S18 ATR-IR spectrum of **1-Dy**.

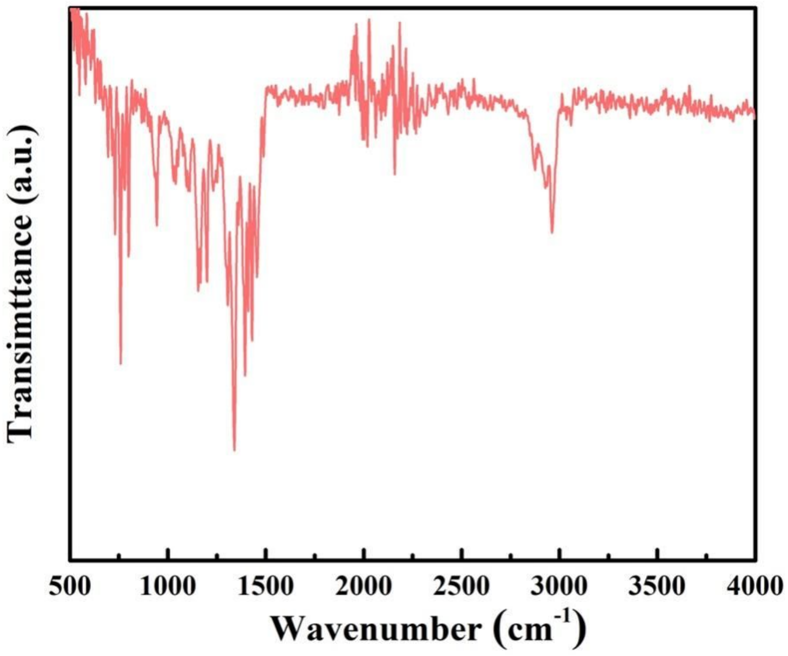

Figure S19 ATR-IR spectrum of **1-Tb**.

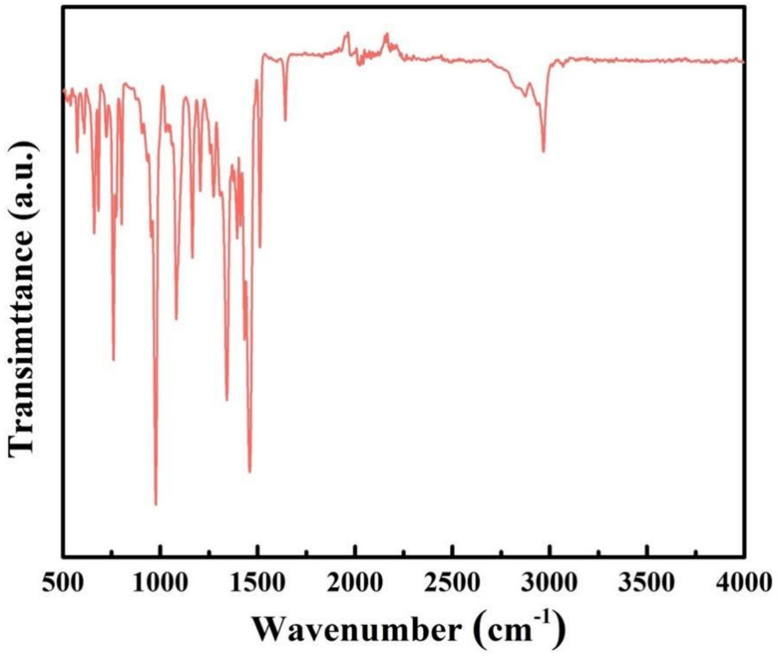

Figure S20 ATR-IR spectrum of **2-Dy**.

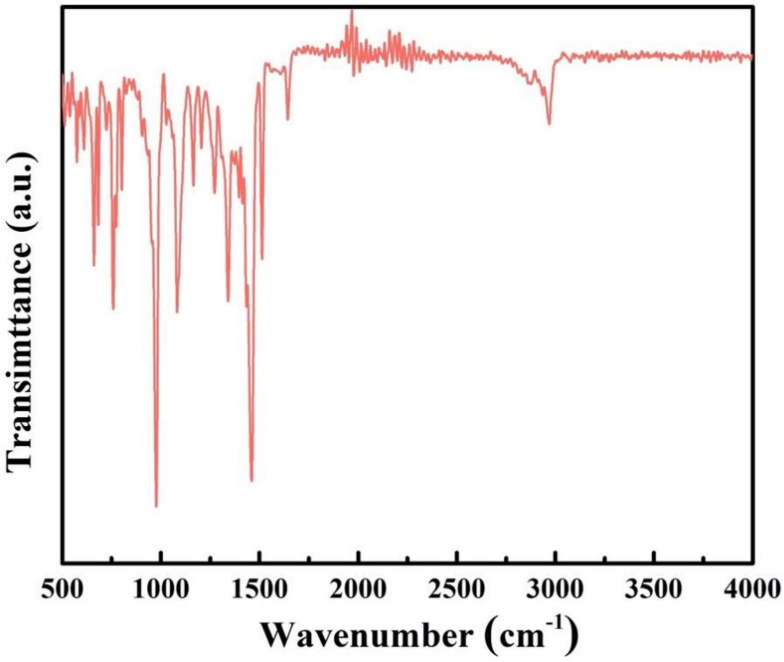

Figure S21 ATR-IR spectrum of **2-Tb**.

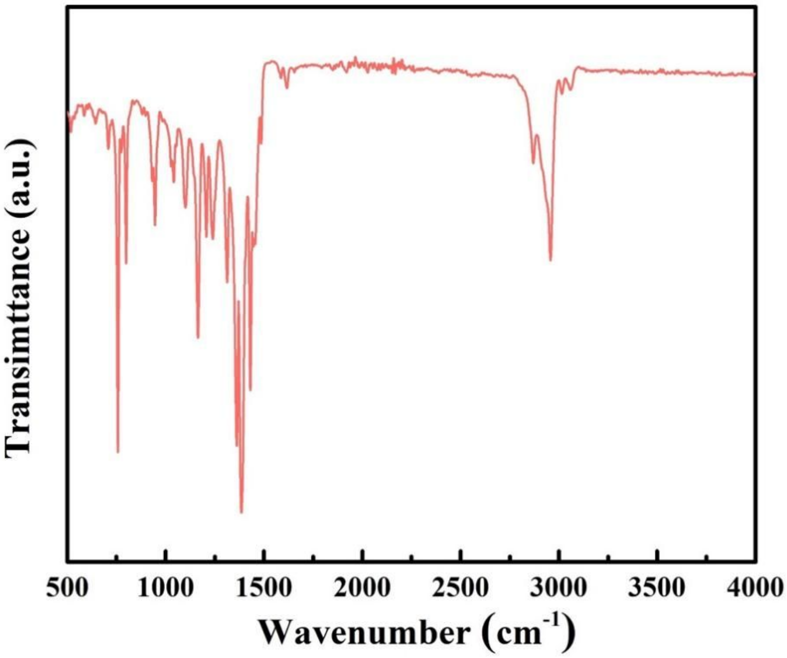

Figure S22 ATR-IR spectrum of **3-Dy**.

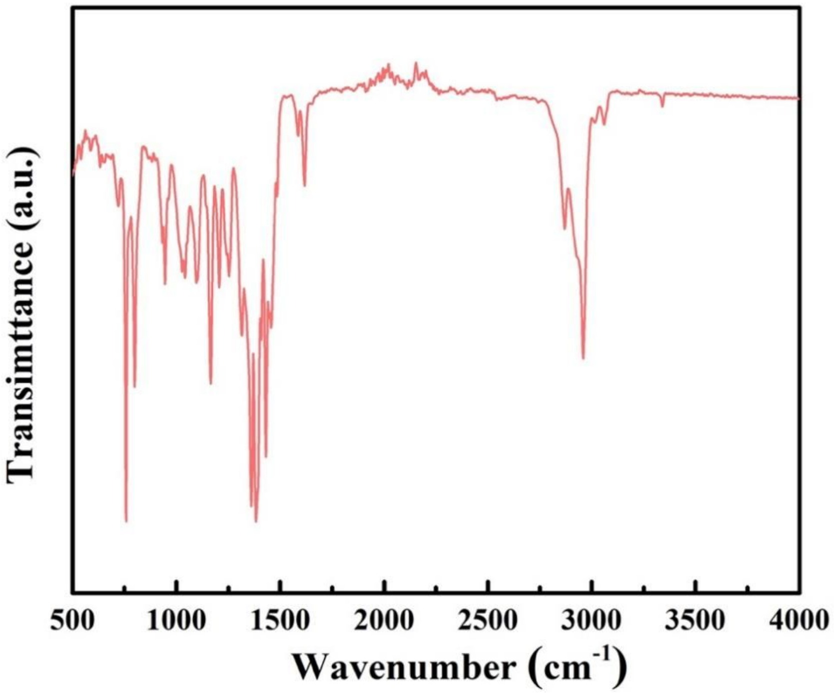

Figure S23 ATR-IR spectrum of **3-Tb**.

3. UV-vis-NIR spectroscopy

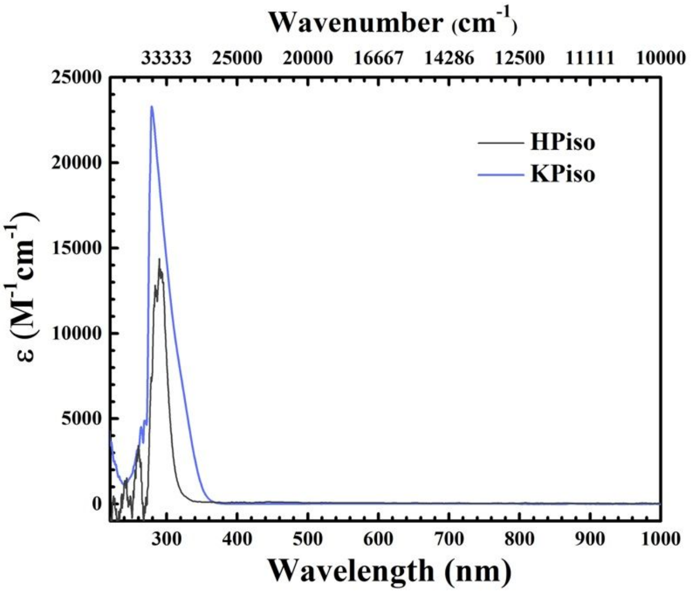

Figure S24 UV-Vis-NIR spectrum of KPiso, 0.2 mM in benzene.

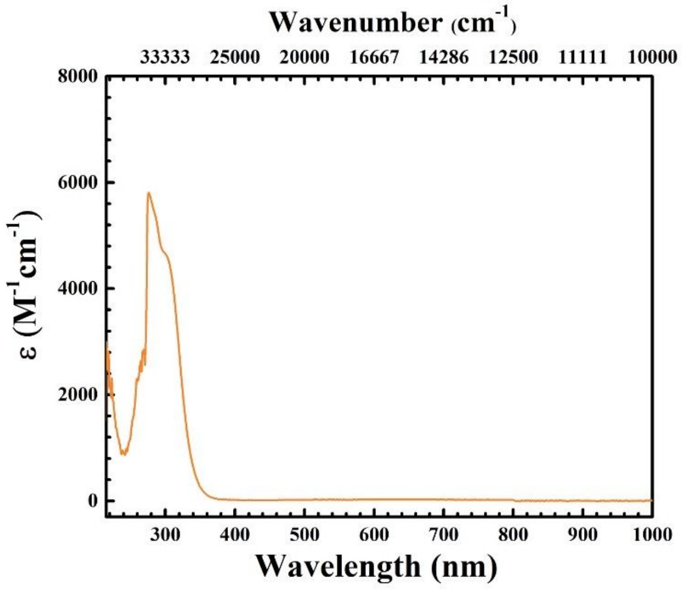

Figure S25 UV-Vis-NIR spectrum of **1-Tb**, 0.2 mM in benzene.

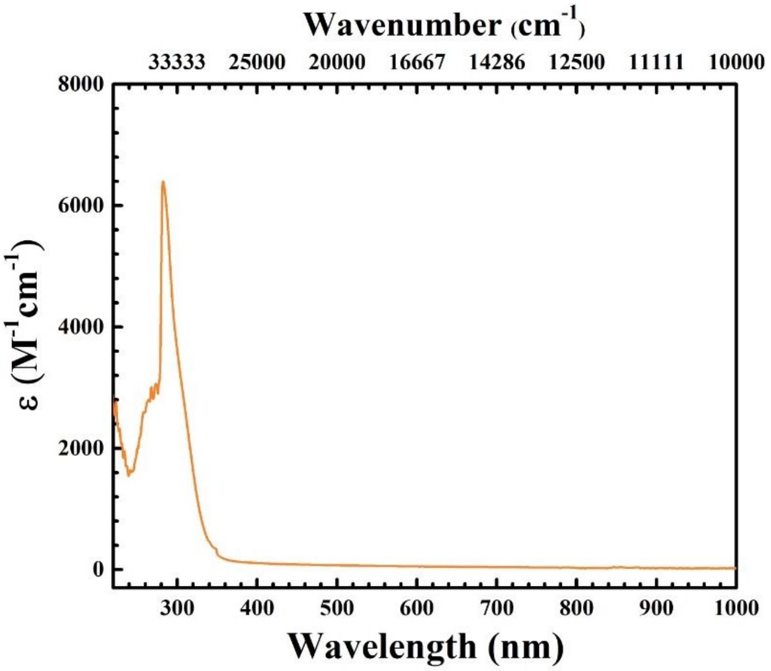

Figure S26 UV-Vis-NIR spectrum of **1-Dy**, 0.2 mM in benzene.

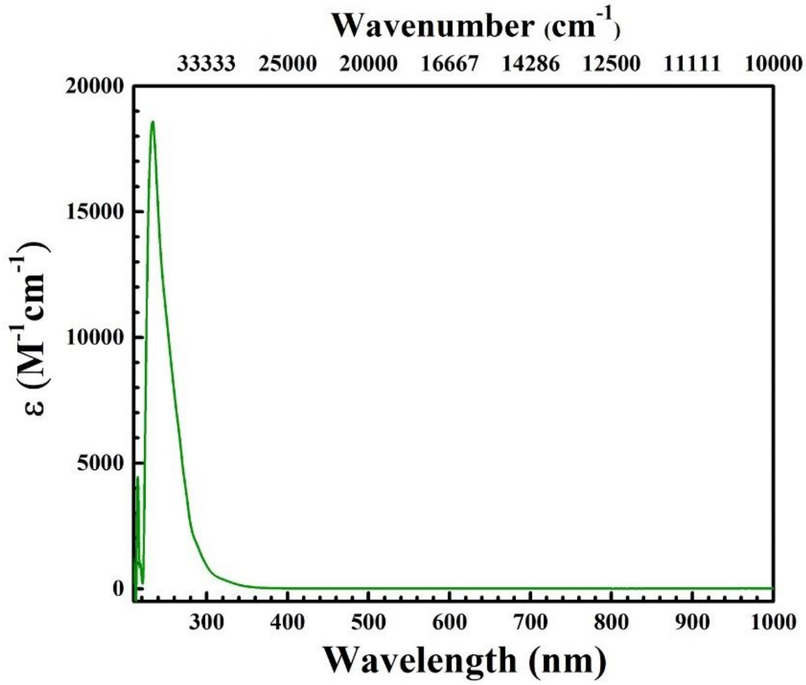

Figure S27 UV-Vis-NIR spectrum of **2-Tb**, 0.2 mM in DCM.

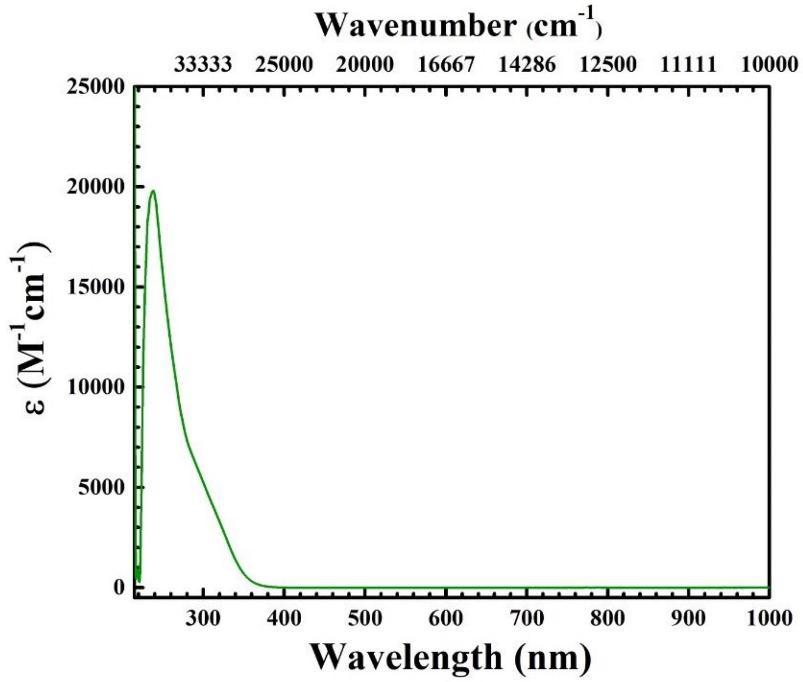

Figure S28 UV-Vis-NIR spectrum of **2-Dy**, 0.2 mM in DCM.

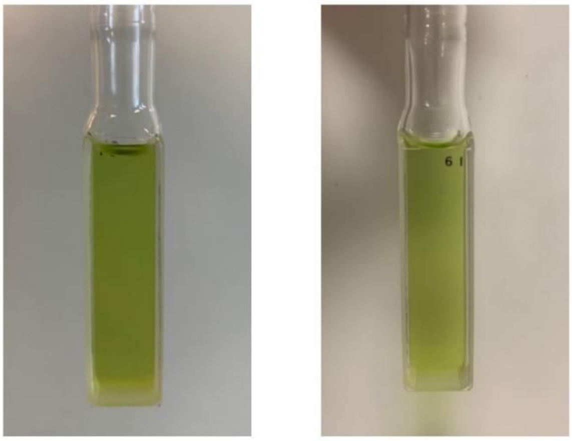

Figure S29 Comparison of solution samples of **3-Tb** (left, 0.29 mM) and **3-Dy** (right, 0.26 mM) in hexane.

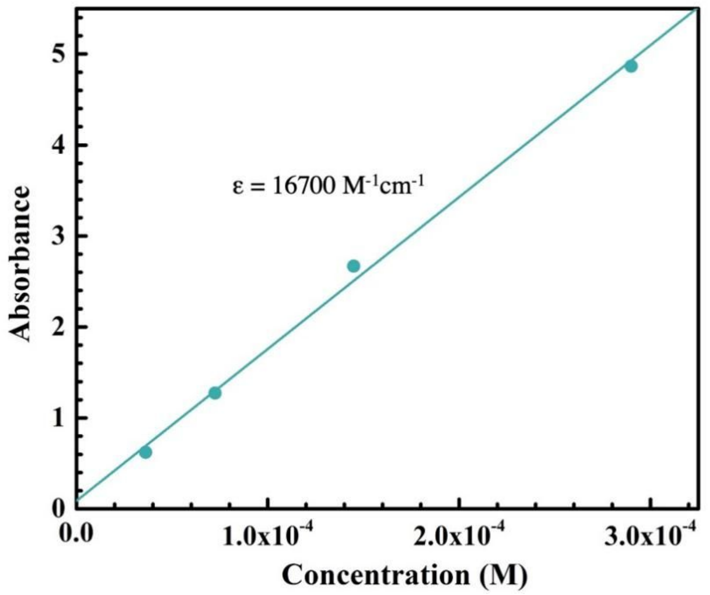

Figure S30 Plot of absorbance versus concentration for the feature at 284 nm in the UV-Vis-NIR spectrum of **3-Tb**. Purple points represent experimental data and the purple line represents the fit to the data used to extract the extinction coefficient.

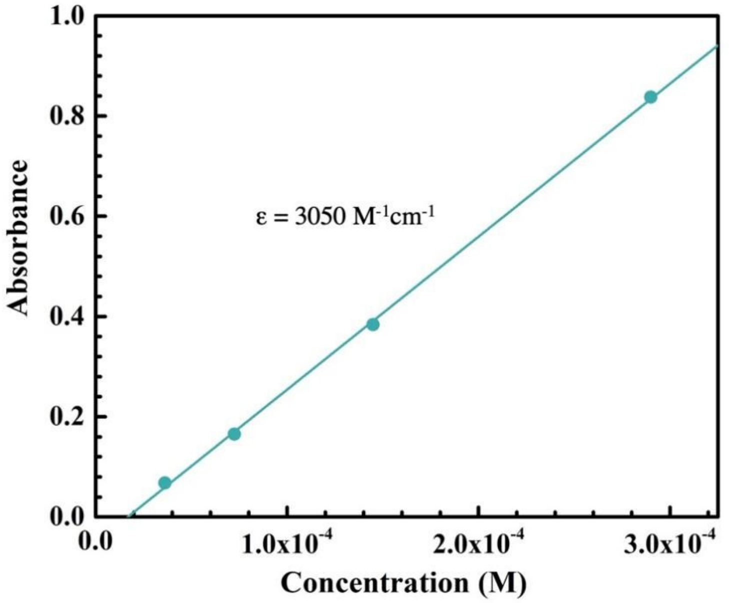

Figure S31 Plot of absorbance versus concentration for the feature at 445 nm in the UV-Vis-NIR spectrum of **3-Tb**. Purple points represent experimental data and the purple line represents the fit to the data used to extract the extinction coefficient.

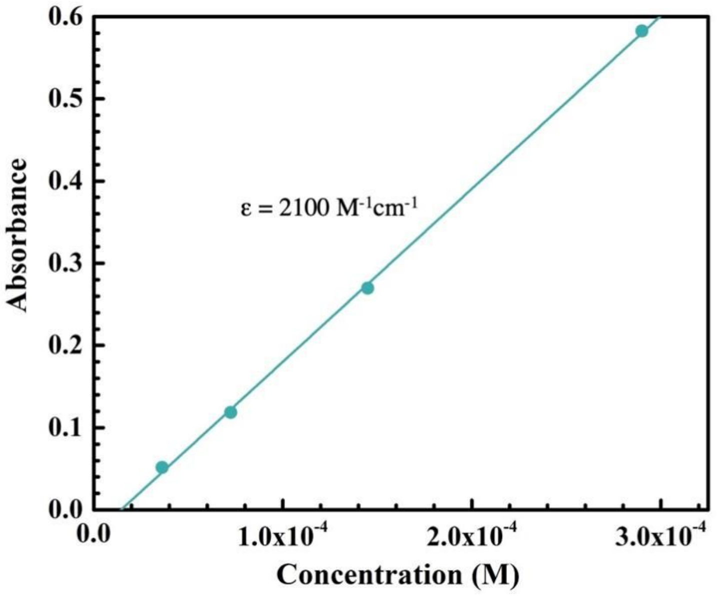

Figure S32 Plot of absorbance versus concentration for the feature at 700 nm in the UV-Vis-NIR spectrum of **3-Tb**. Purple points represent experimental data and the purple line represents the fit to the data used to extract the extinction coefficient.

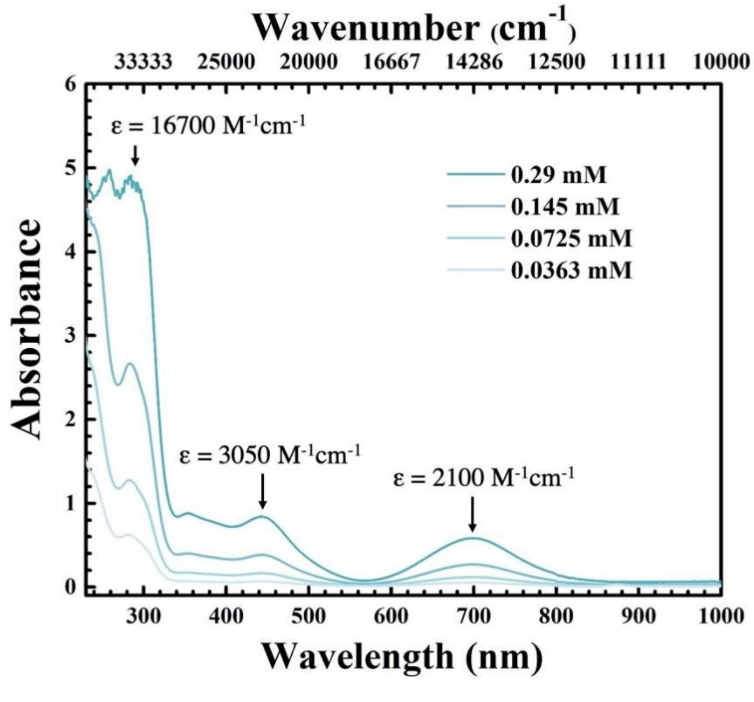

Figure S33 UV-Vis-NIR spectra of **3-Tb** in hexane with varied concentrations.

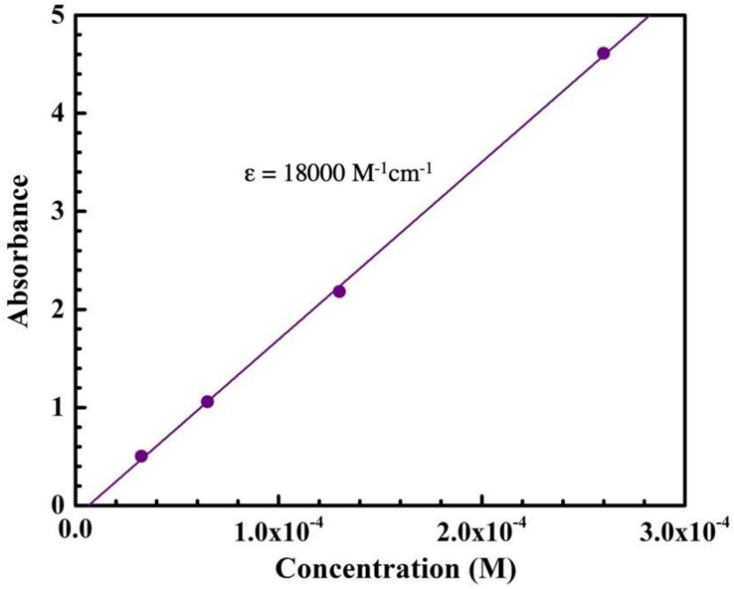

Figure S34 Plot of absorbance versus concentration for the feature at 287 nm in the UV-Vis-NIR spectrum of **3-Dy**. Purple points represent experimental data and the purple line represents the fit to the data used to extract the extinction coefficient.

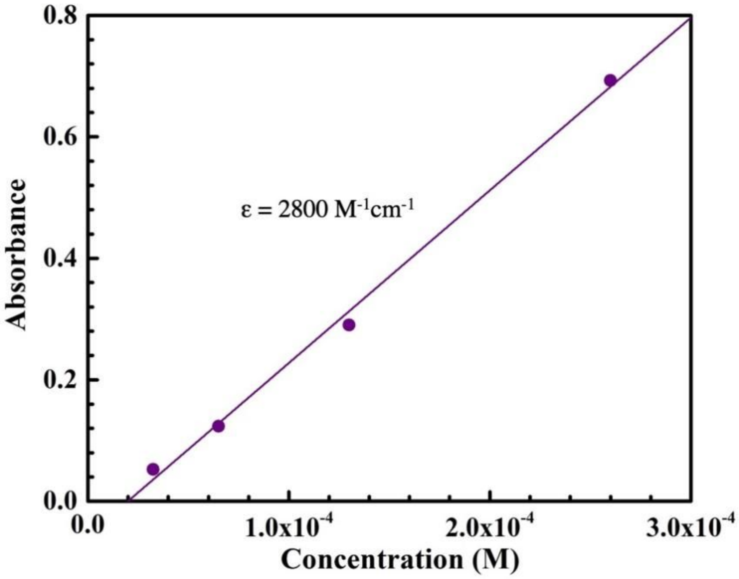

Figure S35 Plot of absorbance versus concentration for the feature at 442 nm in the UV-Vis-NIR spectrum of **3-Dy**. Purple points represent experimental data and the purple line represents the fit to the data used to extract the extinction coefficient.

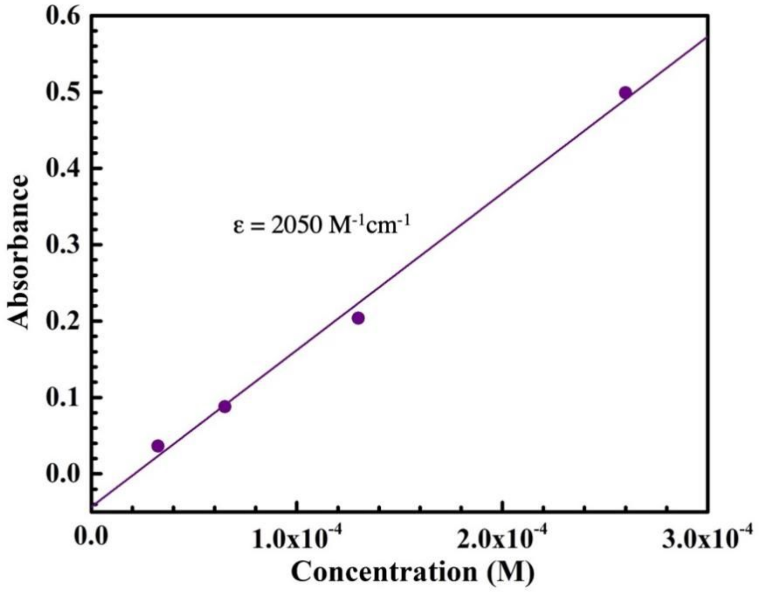

Figure S36 Plot of absorbance versus concentration for the feature at 702 nm in the UV-Vis-NIR spectrum of **3-Dy**. Purple points represent experimental data and the purple line represents the fit to the data used to extract the extinction coefficient.

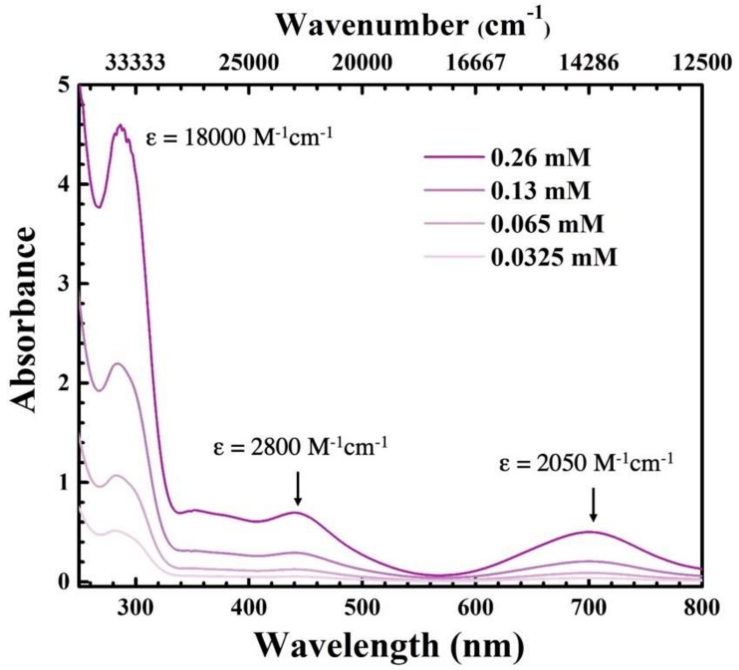

Figure S37 UV-Vis-NIR spectra of **3-Dy** in hexane with varied concentrations.

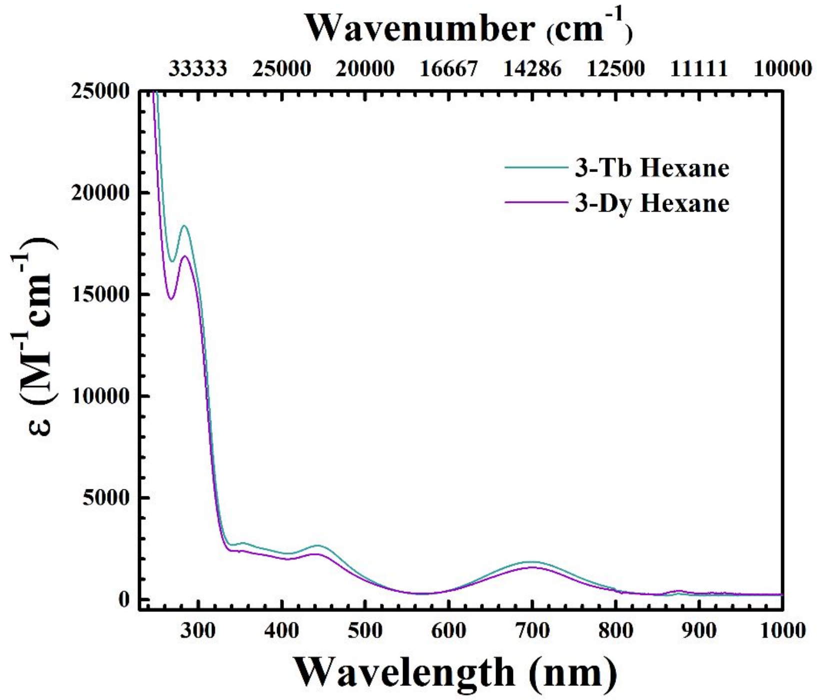

Figure S38 UV-Vis-NIR spectra of **3-Tb** (0.145 mM) and **3-Dy** (0.13 mM) in hexane.

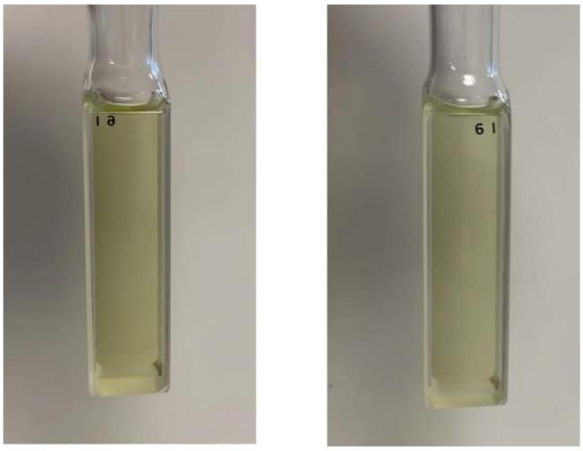

Figure S39 Comparison of solution samples of **3-Tb** (left, 0.19 mM) and **3-Dy** (right, 0.13 mM) in benzene.

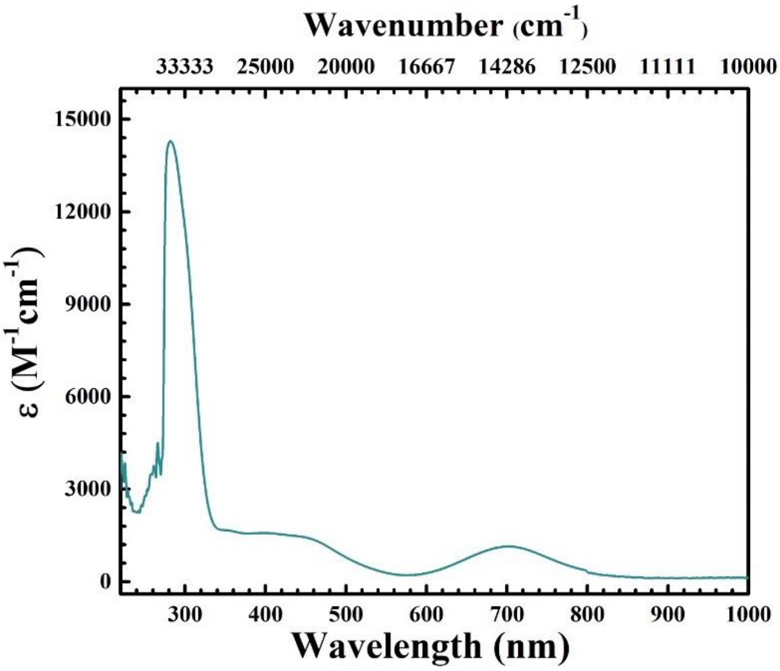

Figure S40 UV-Vis-NIR spectrum of **3-Tb**, 0.19 mM in benzene.

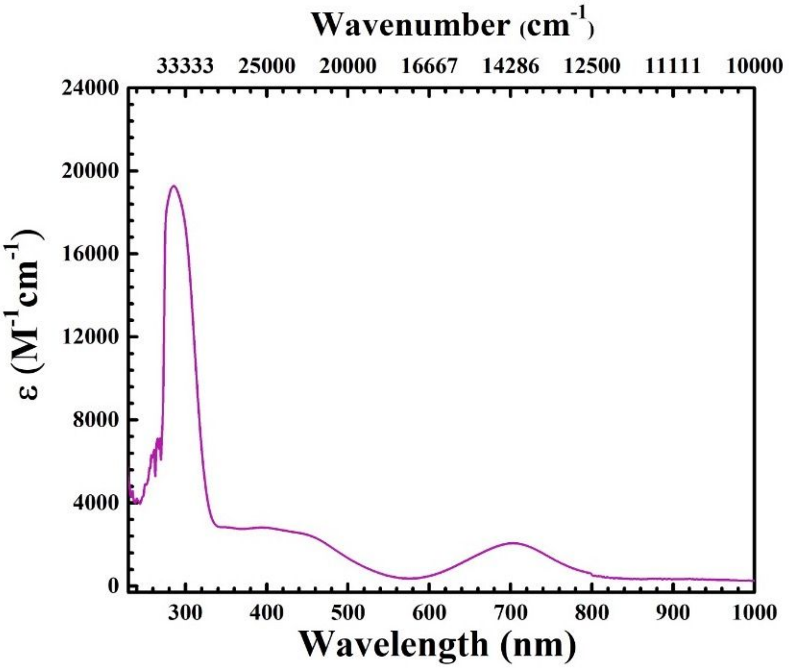

Figure S41 UV-Vis-NIR spectrum of **3-Dy**, 0.13 mM in benzene.

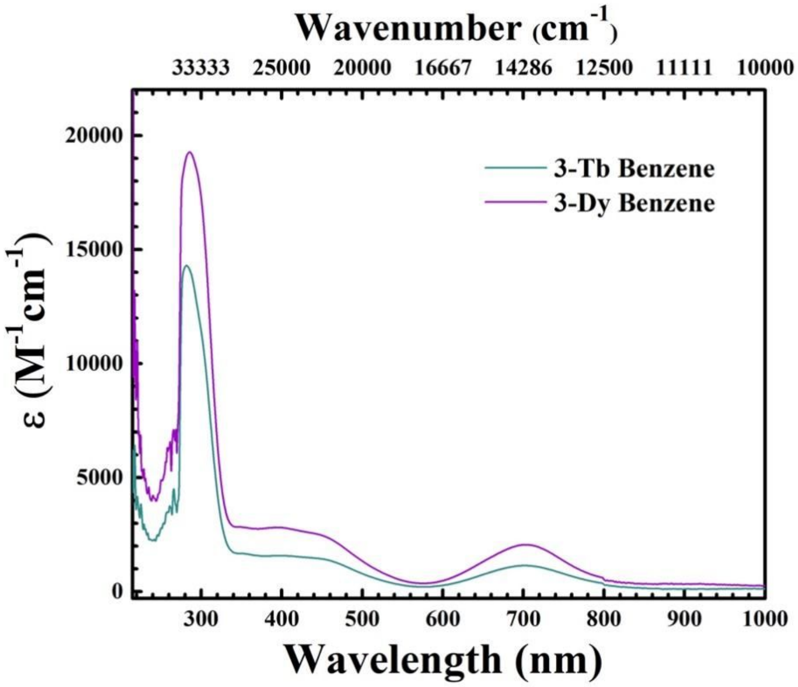

Figure S42 UV-Vis-NIR spectra of **3-Tb** (0.19 mM) and **3-Dy** (0.13 mM) in benzene.

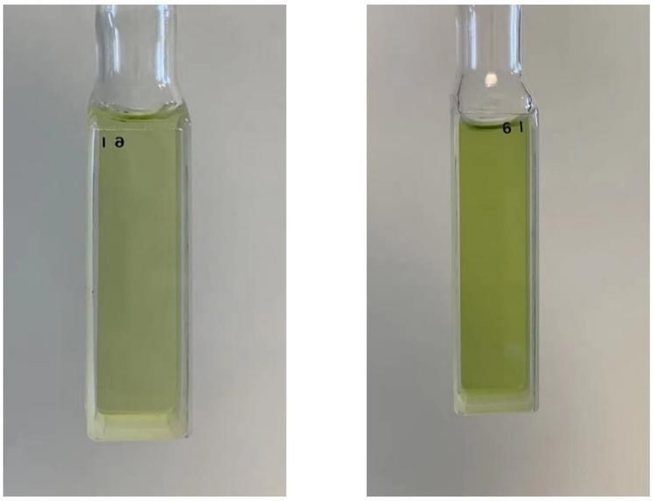

Figure S43 Comparison of solution samples of **3-Tb** (left, 0.26 mM) and **3-Dy** (right, 0.26 mM) in diethyl ether.

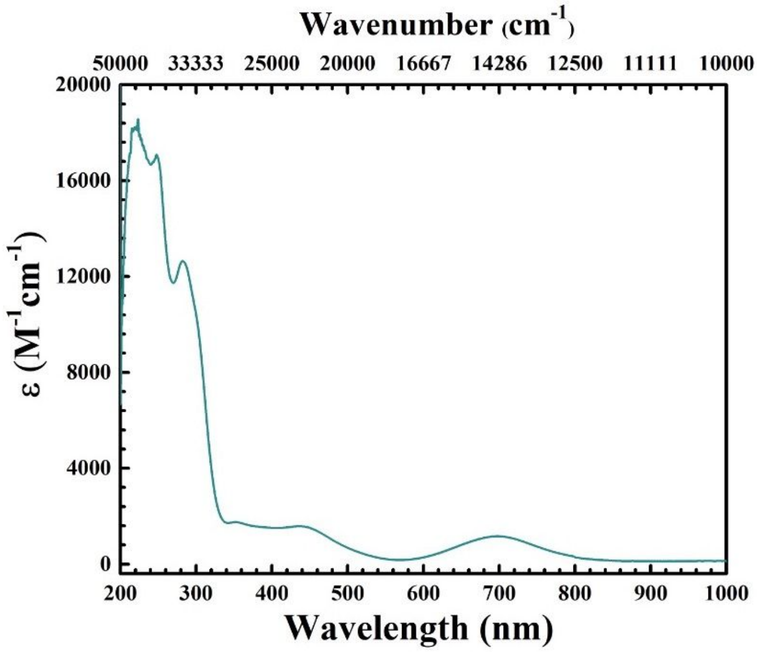

Figure S44 UV-Vis-NIR spectrum of **3-Tb**, 0.26 mM in diethyl ether.

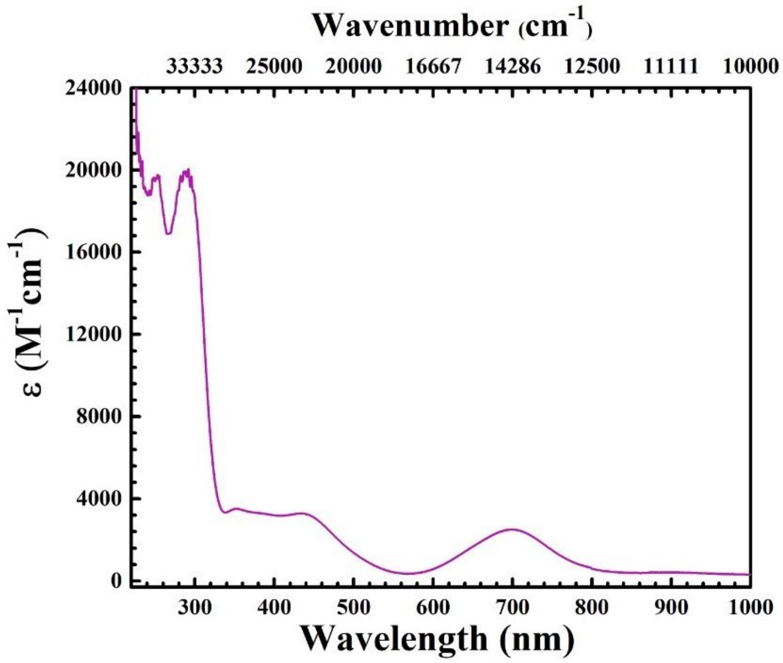

Figure S45 UV-Vis-NIR spectrum of **3-Dy**, 0.26 mM in diethyl ether.

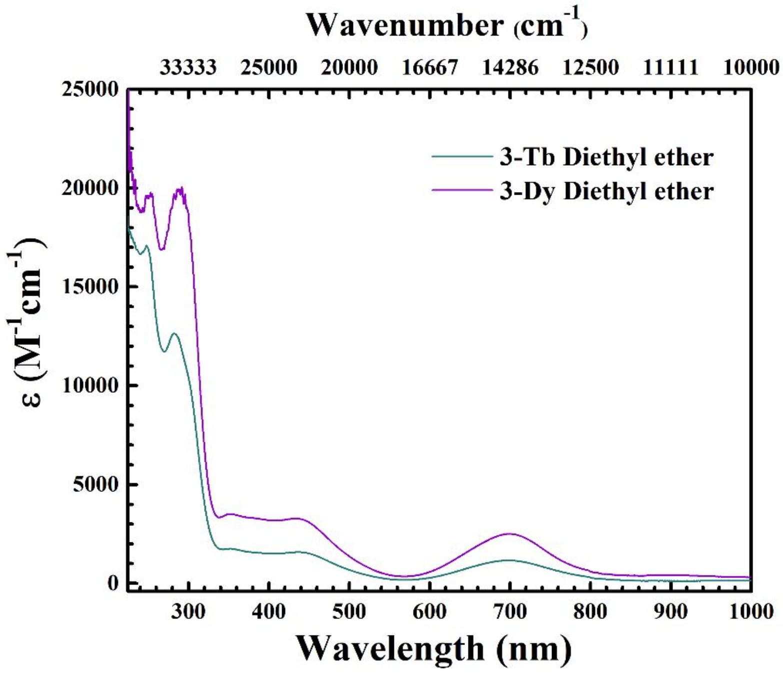

Figure S46 UV-Vis-NIR spectra of **3-Tb** (0.26 mM) and **3-Dy** (0.26 mM) in diethyl ether.

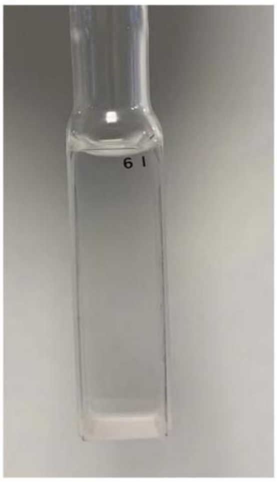

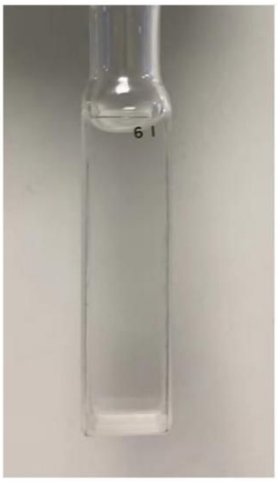

Figure S47 Comparison of solution samples of **3-Tb** (left, 0.26 mM) and **3-Dy** (right, 0.23 mM) in tetrahydrofuran (THF).

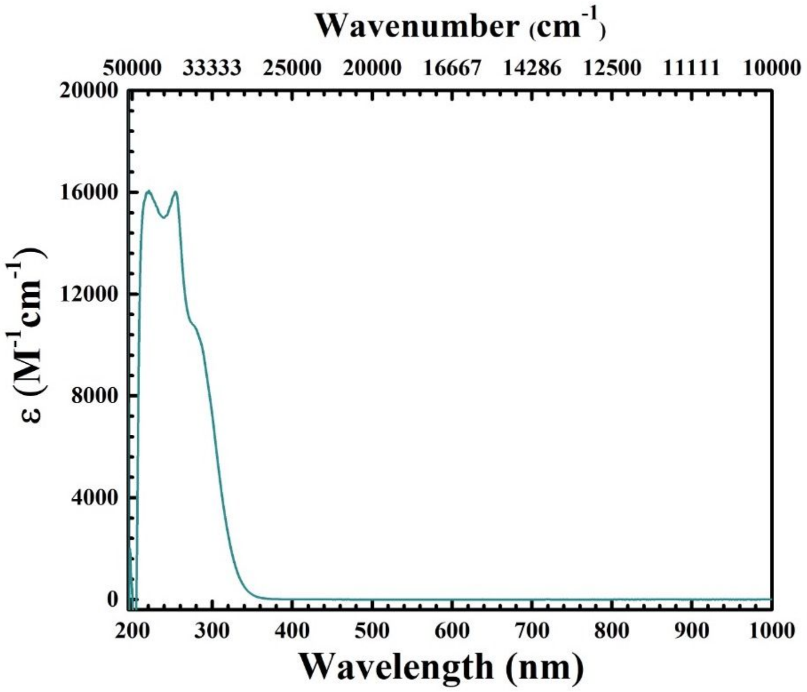

Figure S48 UV-Vis-NIR spectrum of **3-Tb**, 0.26 mM in THF.

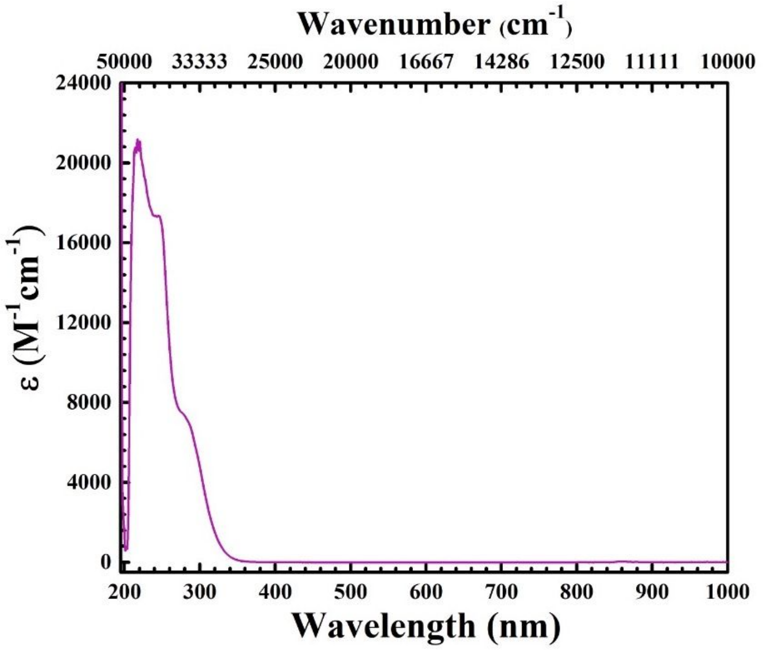

Figure S49 UV-Vis-NIR spectrum of **3-Dy**, 0.23 mM in THF.

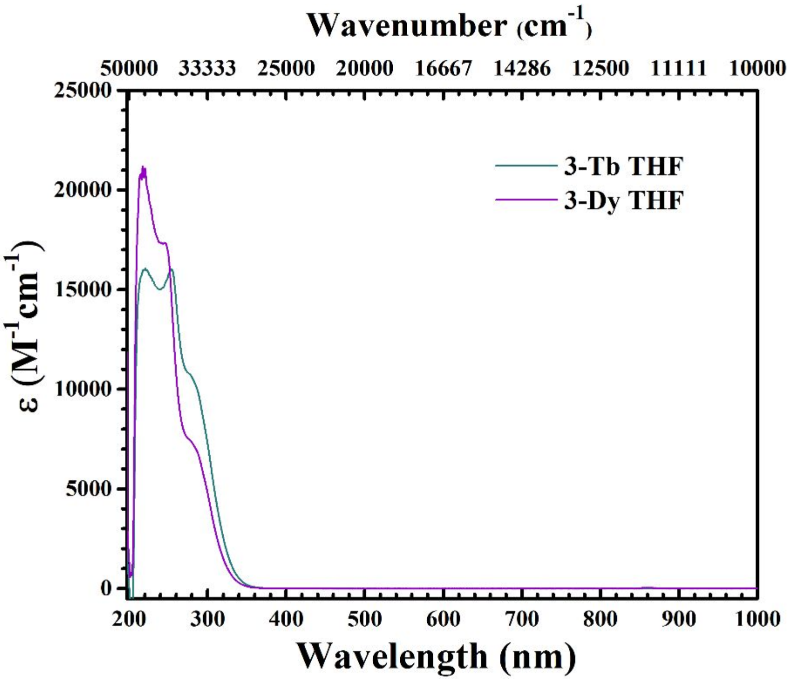

Figure S50 UV-Vis-NIR spectra of **3-Tb** (0.26 mM) and **3-Dy** (0.23 mM) in THF.

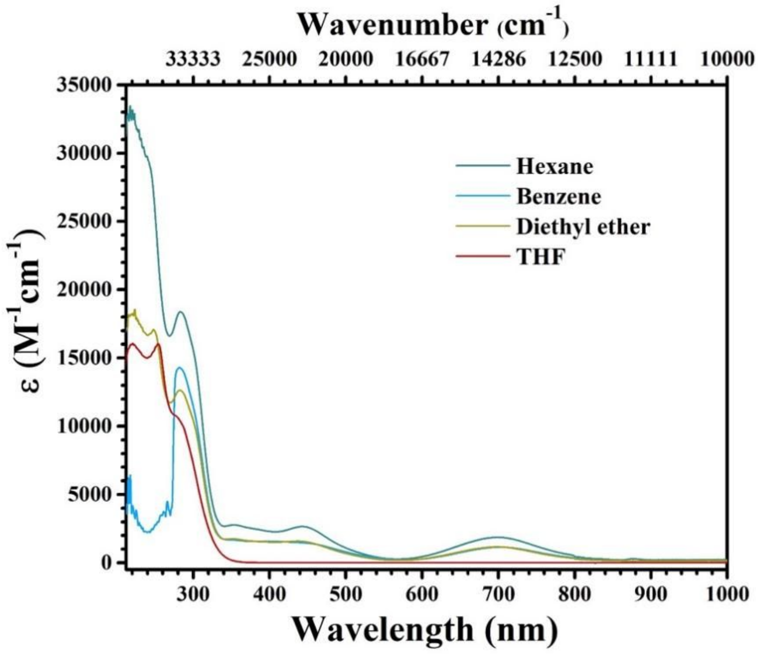

Figure S51 UV-Vis-NIR spectra of **3-Tb** in varied solvents.

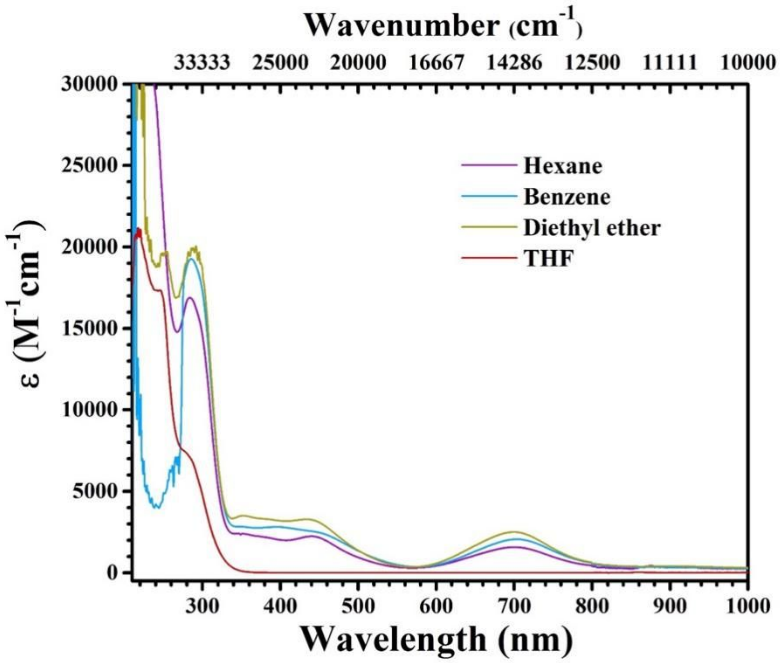

Figure S52 UV-Vis-NIR spectra of **3-Dy** in varied solvents.

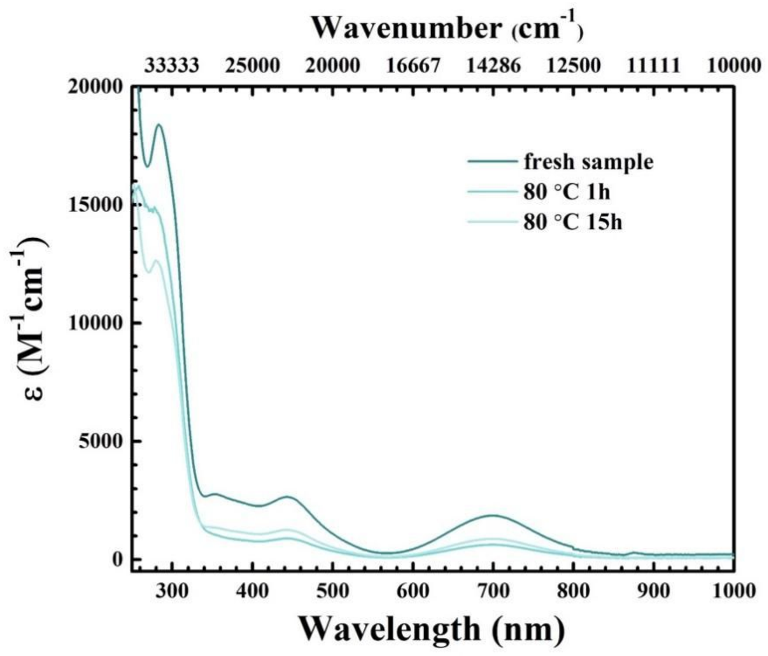

Figure S53 UV-Vis-NIR spectra to compare a fresh solution of **3-Tb** in hexane at room temperature, and aged samples after 1 hour or 15 hours at 80 °C.

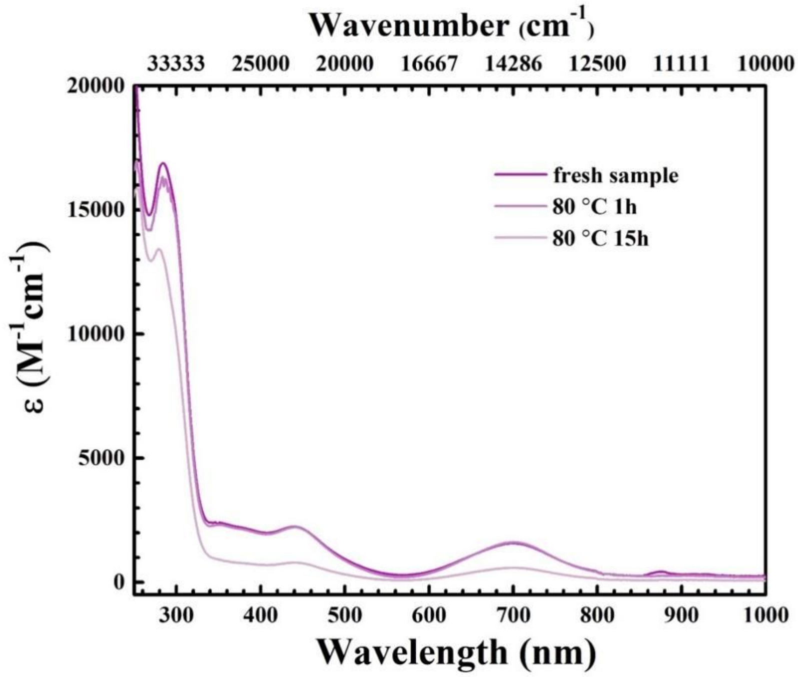

Figure S54 UV-Vis-NIR spectra to compare a fresh solution of **3-Dy** in hexane at room temperature, and aged samples after 1 hour or 15 hours at 80 °C.

4. Single crystal X-ray diffraction

Table S1 Crystallographic data for **1-Dy-I**, **1-Dy-II**, **1-Tb** and **2-Dy**.

**Compounds**	**1-Dy-I**	**1-Dy-II**	**1-Tb**	**2-Dy**
Formula	$C_{65}H_{94}DyIN_4$	$C_{58}H_{86}IN_4Dy$	$C_{65}H_{86}IN_4Tb$	$C_{82}H_{86}BDyF_{20}N_4$
M, g mol^{-1}	1220.84	1128.71	1209.19	1680.85
Temperature, K	100.00(10)	99.95(17)	100(2)	100.0(5)
Space group	$I2$/a	$C2/c$	$I2/a$	$P2_1/n$
a, Å	23.8627(9)	23.8841(6)	23.9271(7)	13.1759(2)
b, Å	10.9246(3)	10.8483(2)	10.9615(3)	28.4695(4)
c, Å	25.2391(10)	22.3545(6)	25.2374(8)	19.9236(3)
α, deg	90	90	90	90
β, deg	116.026(5)	111.886(3)	116.073(4)	91.4920(10)
γ, deg	90	90	90	90
V, Å^3	5912.4(4)	5374.6(2)	5945.6(4)	7471.03(19)
Z	4	23	4	4
d_{cal}, g cm^{-3}	1.372	1.395	1.351	1.494
Radiation	MoKα (λ = 0.71073)	MoKα (λ = 0.71073)	MoKα (λ = 0.71073)	MoKα (λ = 0.71073)
2θ range, deg	6.812 to 58.376	3.676 to 61.372	6.794 to 58.134	3.932 to 49.998
R_{int}	0.0301	0.0449	0.0305	0.0480
Final indexes [F^2>2σ(F^2)]	R_1 = 0.0339, wR_2 = 0.0825	R_1 = 0.0296, wR_2 = 0.0680	R_1 = 0.0334, wR_2 = 0.0966	R_1 = 0.0377, wR_2 = 0.0945
R indexes (all data)	R_1 = 0.0385, wR_2 = 0.0855	R_1 = 0.0350, wR_2 = 0.0698	R_1 = 0.0399, wR_2 = 0.1079	R_1 = 0.0444, wR_2 = 0.1005
S^a	1.046	1.075	0.771	1.037
Residual map, e Å^{-3}	1.04/-2.30	1.44/-1.17	1.24/-0.97	2.70/-1.22

^{a} Conventional $R= \Sigma||F_o| - |F_c||/\Sigma|F_o|$; $R_w= [\Sigma w(F_o^2 - F_c^2)^2/\Sigma w(F_o^2)^2]^{1/2}$; $S= [\Sigma w(F_o^2 - F_c^2)^2/\text{no. data} - \text{no. params})]^{1/2}$ for all data.

Table S2 Crystallographic data for **2-Tb**, **3-Dy** and **3-Tb**.

**Compounds**	**2-Tb**	**3-Dy**	**3-Tb**
Formula	$C_{82}H_{86}BF_{20}N_4Tb$	$C_{63}H_{98}N_4Dy$	$C_{63}H_{98}N_4Tb$
M, g mol^{-1}	1677.341	1073.95	1070.37
Temperature, K	100.0(5)	100.01(10)	102(3)
Space group	$P2_1/n$	$P2_1/n$	$P2_1/n$
a, Å	13.1682(1)	12.29413(14)	12.30337(9)
b, Å	28.5085(2)	23.7442(2)	23.78249(19)
c, Å	19.9105(1)	20.6226(3)	20.58510(19)
α, deg	90	90	90
β, deg	91.569(1)	99.5844(11)	99.6868(8)
γ, deg	90	90	90
V, Å^3	7471.71(9)	5935.99(12)	5937.42(9)
Z	4	4	4
d_{cal}, g cm^{-3}	1.491	1.202	1.197
Radiation	CuKα (λ = 1.54184)	CuKα (λ = 1.54184)	CuKα (λ = 1.54184)
2θ range, deg	5.42 to 133.2	5.722 to 151.908	5.726 to 155.108
R_{int}	0.0381	0.0354	0.0597
Final indexes [F^2>2σ(F^2)]	R_1 = 0.0364, wR_2 = 0.0950	R_1 = 0.0370, wR_2 = 0.0937	R_1 = 0.0499, wR_2 = 0.1319
R indexes (all data)	R_1 = 0.0386, wR_2 = 0.0963	R_1 = 0.0430, wR_2 = 0.0965	R_1 = 0.0533, wR_2 = 0.1343
S^a	1.045	1.026	1.024
Residual map, e Å^{-3}	2.81/-0.81	0.87/-0.49	2.23/-1.06

^{a} Conventional $R= \Sigma||F_o| - |F_c||/\Sigma|F_o|$; $R_w= [\Sigma w(F_o^2 - F_c^2)^2/\Sigma w(F_o^2)^2]^{1/2}$; $S= [\Sigma w(F_o^2 - F_c^2)^2$/no. data – no. params)]$^{1/2}$ for all data.

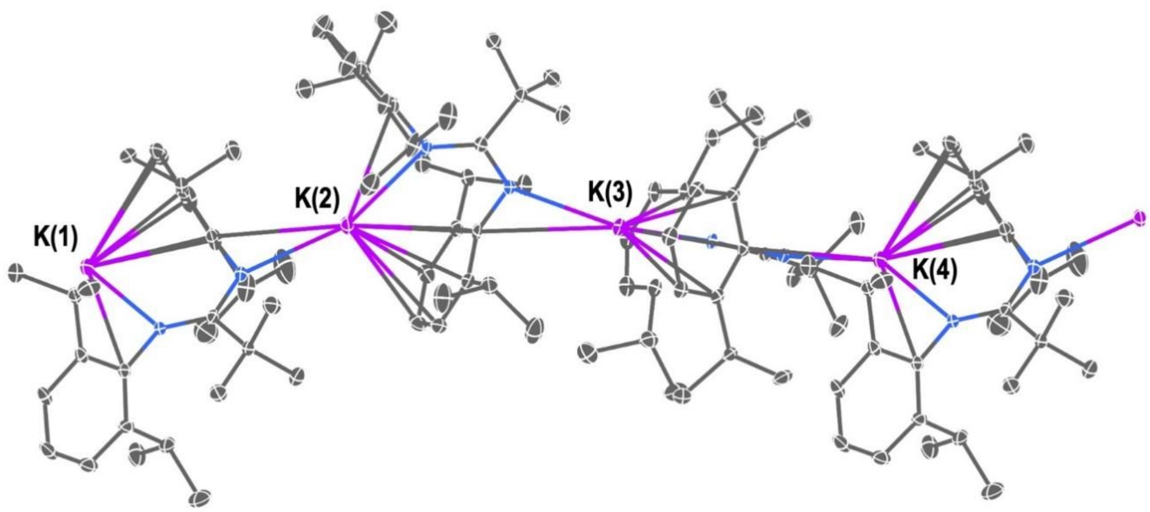

Figure S55 Molecular structure of KPiso with selective atom labelling (C = gray, N = blue, K = purple). Thermal ellipsoids set at 50 % probability level and hydrogen atoms omitted for clarity.

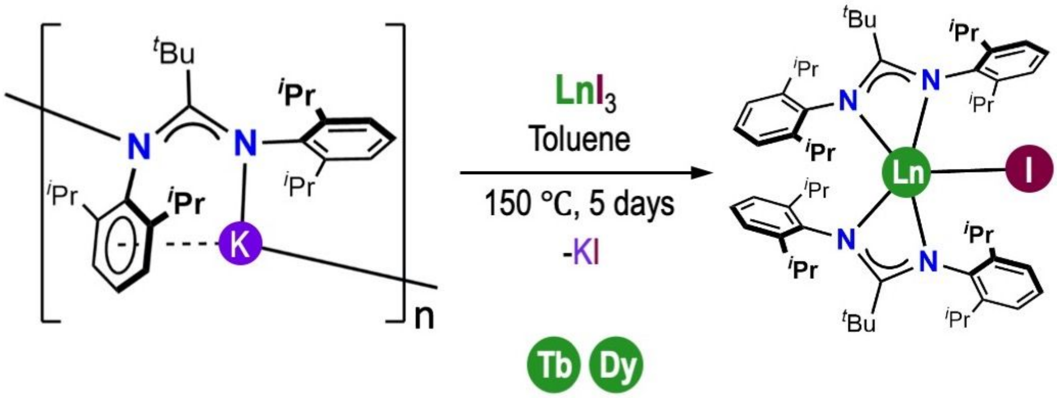

Scheme S1 Synthesis of **1-Ln**.

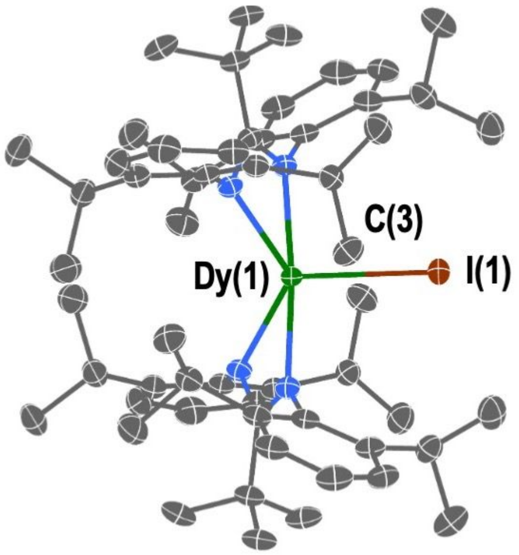

Figure S56 Molecular structure of **1-Dy** with selective atom labelling (C = gray, N = blue, I = brown, Dy = olive green). Thermal ellipsoids set at 50 % probability level and hydrogen atoms omitted for clarity. Selected distances: Dy(1)···I(1), 2.8918(2) Å; Dy(1)···C(3), 3.600 Å; Dy(1)···$H_{C(3)}$, 2.919 Å.

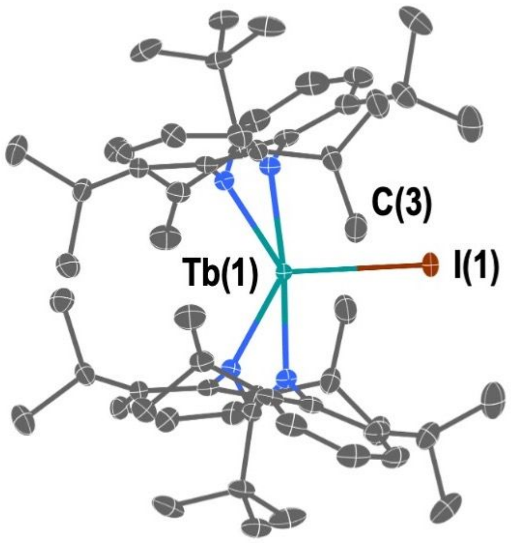

Figure S57 Molecular structure of **1-Tb** with selective atom labelling (C = gray, N = blue, I = brown, Tb = cyan). Thermal ellipsoids set at 50 % probability level and hydrogen atoms omitted for clarity. Selected distances: Tb(1)···I(1), 2.9157(3) Å; Tb(1)···C(3), 3.667 Å; Tb(1)···$H_{C(3)}$, 3.018 Å.

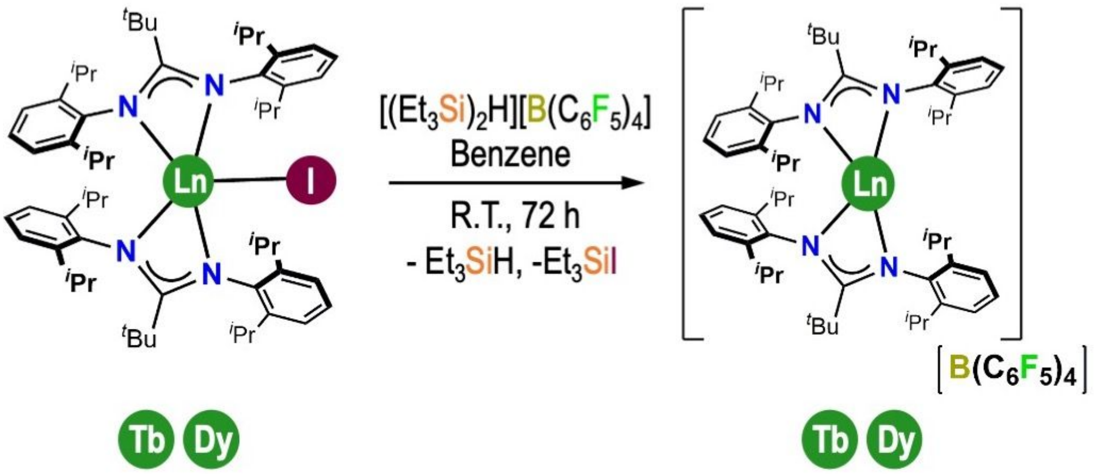

Scheme S2 Synthesis of **2-Ln** from **1-Ln**.

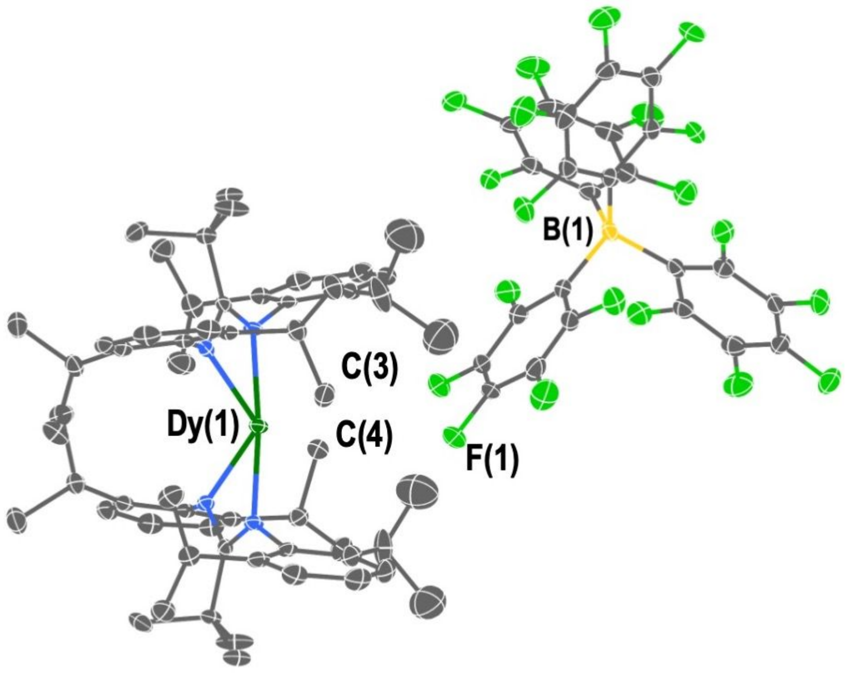

Figure S58 Molecular structure of **2-Dy** with selective atom labelling (C = gray, N = blue, F = green, B = yellow, Dy = olive green). Thermal ellipsoids set at 50 % probability level and hydrogen atoms omitted for clarity. Selected distances: Dy(1)···C(3), 3.463(4) Å; Dy(1)···C(4), 3.119(4) Å; Dy(1)···$H_{C(3)}$, 2.667 Å; Dy(1)···$H_{C(4)}$, 2.465 Å; Dy(1)···F(1), 5.719(3) Å.

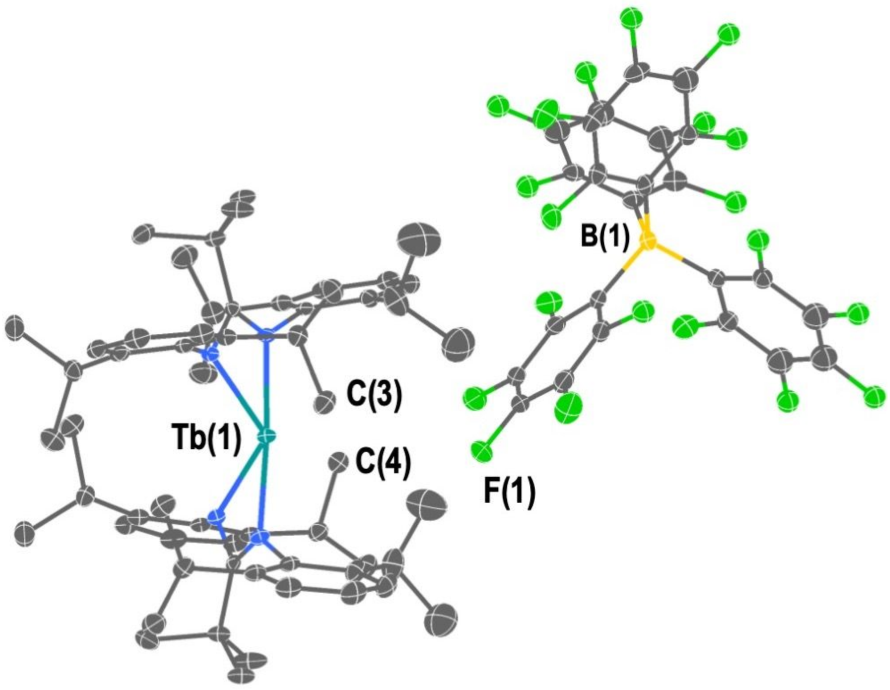

Figure S59 Molecular structure of **2-Tb** with selective atom labelling (C = gray, N = blue, F = green, B = yellow, Tb = cyan). Thermal ellipsoids set at 50 % probability level and hydrogen atoms omitted for clarity. Selected distances: Tb(1)···C(3), 3.453(3) Å; Tb(1)···C(4), 3.136(3) Å; Tb(1)···$H_{C(3)}$, 2.669(9) Å; Tb(1)···$H_{C(4)}$, 2.47(1) Å; Tb(1)···F(1), 5.766(2) Å.

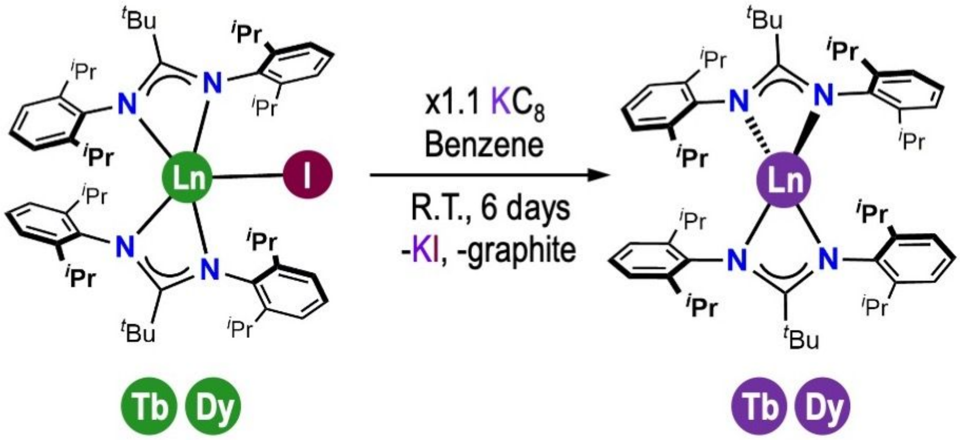

Scheme S3 Synthesis of **3-Ln** from **1-Ln**.

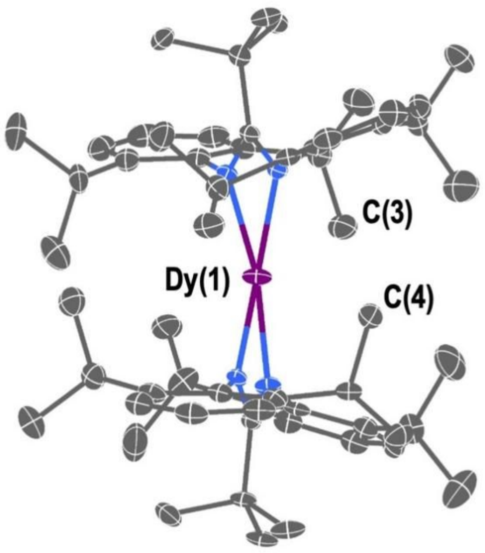

Figure S60 Molecular structure of **3-Dy** with selective atom labelling (C = gray, N = blue, Dy = purple). Dy(1) is with occupation 0.899(4). Thermal ellipsoids set at 50 % probability level and hydrogen atoms omitted for clarity. Selected distances: Dy(1)···C(3), 3.926(3) Å; Dy(1)···C(4), 4.104(4) Å; Dy(1)···$H_{C(3)}$, 3.082 Å; Dy(1)···$H_{C(4)}$, 3.308 Å.

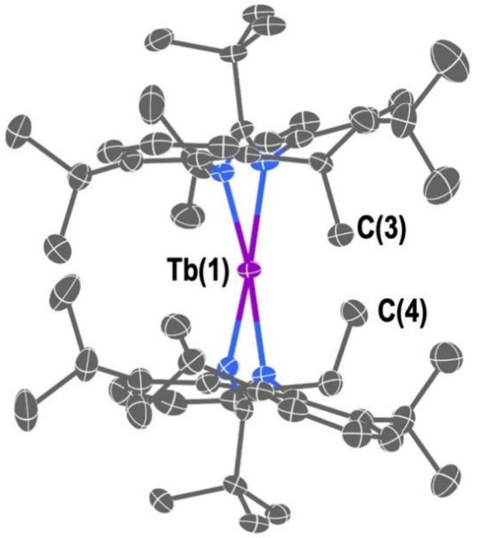

Figure S61 Molecular structure of **3-Tb** with selective atom labelling (C = gray, N = blue, Tb = purple). Tb(1) is with occupation 0.855(3). Thermal ellipsoids set at 50 % probability level and hydrogen atoms omitted for clarity. Selected distances: Tb(1)···C(3), 3.931(4) Å; Tb(1)···C(4), 4.108(4) Å; Tb(1)···$H_{C(3)}$, 3.100 Å; Tb(1)···$H_{C(4)}$, 3.325 Å.

Figure S62 and **Table S3** Molecular structure of **1-Dy**, **2-Dy** and **3-Dy** with selective atom labelling. Table shows selected bond distances (Å) and angles (°) for **1-Dy** (left), **2-Dy** (middle) and **3-Dy** (right) respectively.

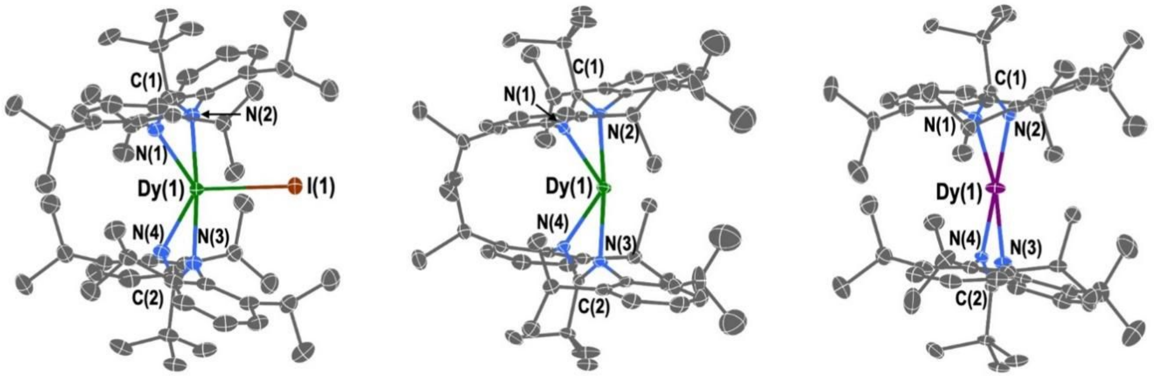

Complexes	*[Dy(Piso)$_2$I]* (1-Dy)	*[Dy(Piso)$_2$]$^+$* (2-Dy)	*[Dy(Piso)$_2$]* (3-Dy)[a]
Space Group	*C*2/*c*	*P*2$_1$/*n*	*P*2$_1$/*n*
N(1)-Dy(1)	2.314(2)	2.260(3)	2.317(2)
N(2)-Dy(1)	2.404(2)	2.332(2)	2.363(2)
N(3)-Dy(1)	2.404(2)	2.313(3)	2.365(2)
N(4)-Dy(1)	2.314(2)	2.258(3)	2.314(2)
C(1)-Dy(1)	2.832(2)	2.769(3)	2.834(2)
C(2)-Dy(1)	2.832(2)	2.758(3)	2.827(3)
Average N-Dy[b]	2.359(2)	2.291(3)	2.340(2)
N(1)-Dy(1)-N(3)	120.72(6)	119.55(9)	125.86(7)
N(1)-Dy(1)-N(4)	122.26(10)	118.90(9)	155.90(9)
N(2)-Dy(1)-N(3)	174.82(9)	174.44(9)	164.44(9)
N(2)-Dy(1)-N(4)	120.73(9)	118.52(9)	129.42(8)
C(1)-Dy(1)-C(2)	149.70(9)	148.80(9)	174.82(8)
N(1)N(2)Dy(1)-Dy(1)N(3)N(4)	67.01(7)	69.90(9)	43.03(19)

[a] Values for **3-Dy** are from the metal position of occupation 0.899(4).

[b] The standard deviation of the average values are from $\sqrt{\Sigma\sigma_i^2/N}$. ($\sigma_i$ is the standard deviation of each bond distance; *i* and *N* are the number of distances to average).

Figure S63 and **Table S4** Molecular structure of **1-Tb**, **2-Tb** and **3-Tb** with selective atom labelling. Table shows selected bond distances (Å) and angles (°) for **1-Tb** (left), **2-Tb** (middle) and **3-Tb** (right) respectively.

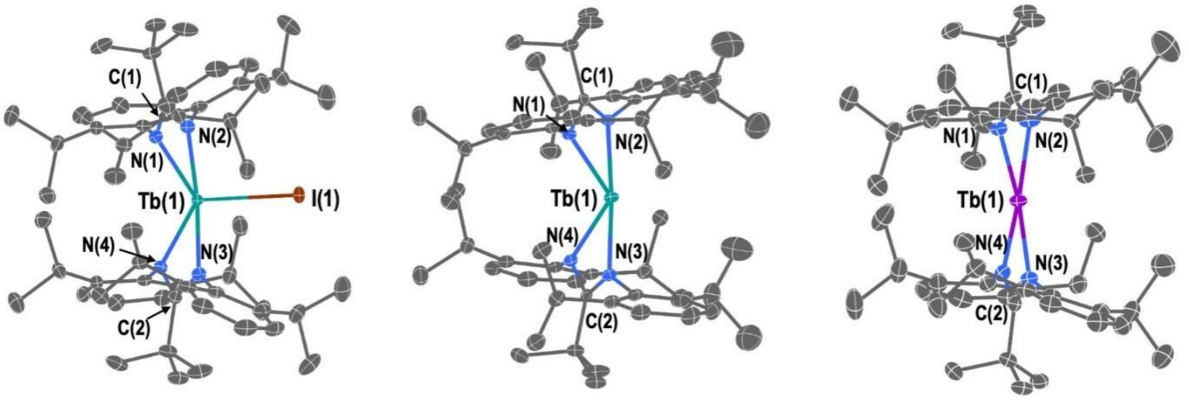

**Complexes**	***[Tb(Piso)$_2$I]*** **(1-Tb)**	***[Tb(Piso)$_2$]$^+$*** **(2-Tb)**	***[Tb(Piso)$_2$]*** **(3-Tb)^a**
Space Group	*I*2/*a*	*P*2$_1$/*n*	*P*2$_1$/*n*
N(1)-Tb(1)	2.321(2)	2.267(2)	2.330(2)
N(2)-Tb(1)	2.424(2)	2.352(2)	2.366(2)
N(3)-Tb(1)	2.424(2)	2.335(2)	2.367(2)
N(4)-Tb(1)	2.321(2)	2.257(2)	2.312(3)
C(1)-Tb(1)	2.856(3)	2.786(2)	2.840(3)
C(2)-Tb(1)	2.856(3)	2.776(2)	2.832(3)
Average N-Tb(1)^b	2.373(2)	2.303(2)	2.344(2)
N(1)-Tb(1)-N(3)	120.80(8)	121.20(7)	125.85(8)
N(1)-Tb(1)-N(4)	122.43(13)	119.85(7)	156.09(10)
N(2)-Tb(1)-N(3)	174.54(12)	176.04(7)	164.95(10)
N(2)-Tb(1)-N(4)	120.81(8)	119.50(7)	129.14(9)
C(1)-Tb(1)-C(2)	148.55(12)	150.01(7)	174.58(9)
N(1)N(2)Tb(1)-Tb(1)N(3)N(4)	66.79(75)	70.10(66)	42.14(11)

^{a} Values for **3-Tb** are from the metal position of occupation 0.855(3).

^{b} The standard deviation of the average values are calculated with $\sqrt{\sum \sigma_i^2 / N}$. ($\sigma_i$ is the standard deviation of each bond distance; *i* and *N* are the number of distances to average).

5. Powder X-ray diffraction

a)

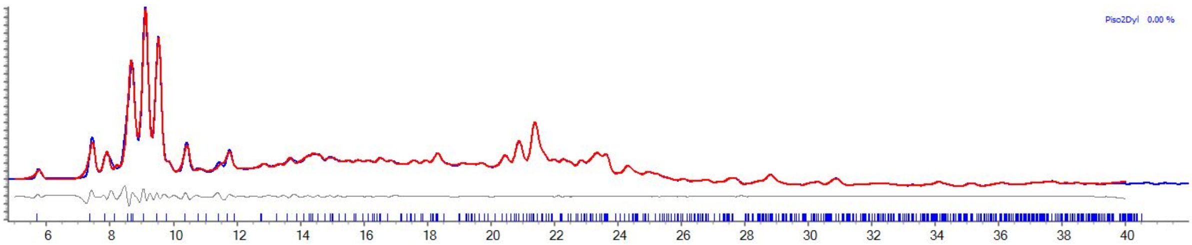

b)

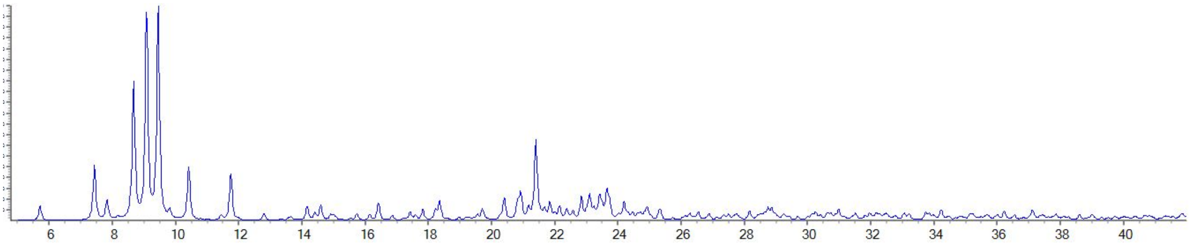

Figure S64 (a) Pawley refinement analysis of **3-Dy**. Blue experimental data, Red model, and gray difference; (b) theoretical powder X-ray diffraction pattern of **3-Dy**. Pawley refinement Rwp 1.917; Rwp'=7.995.

(a)

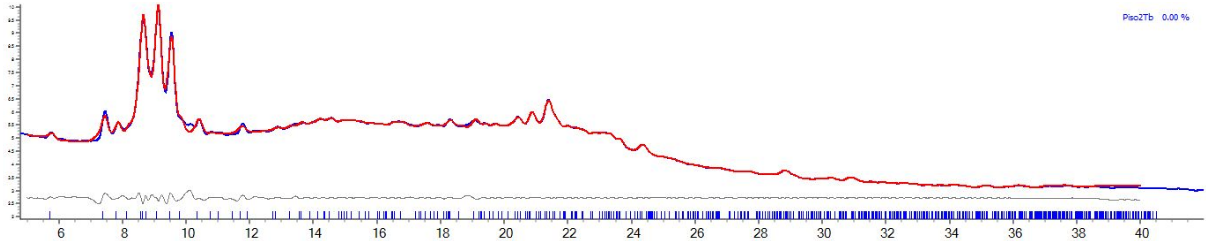

(b)

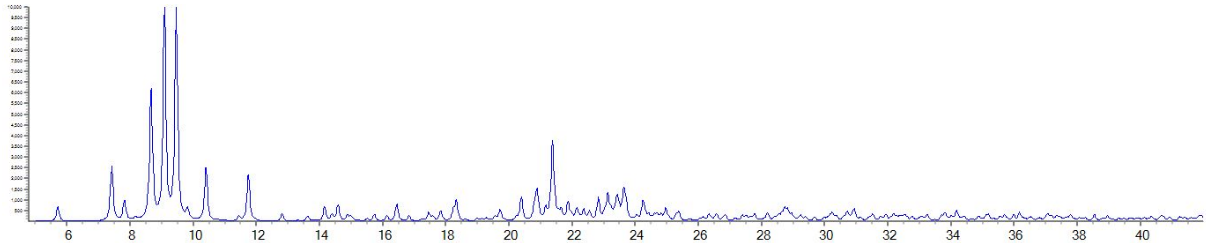

Figure S65 (a) Pawley refinement analysis of **3-Tb**. Blue experimental data, Red model, and gray difference; (b) theoretical powder X-ray diffraction pattern of **3-Tb**. Pawley refinement Rwp 0.894; Rwp'=6.957.

(a)

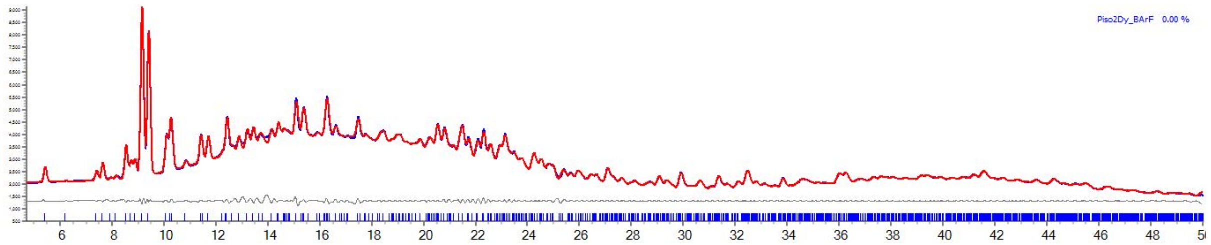

(b)

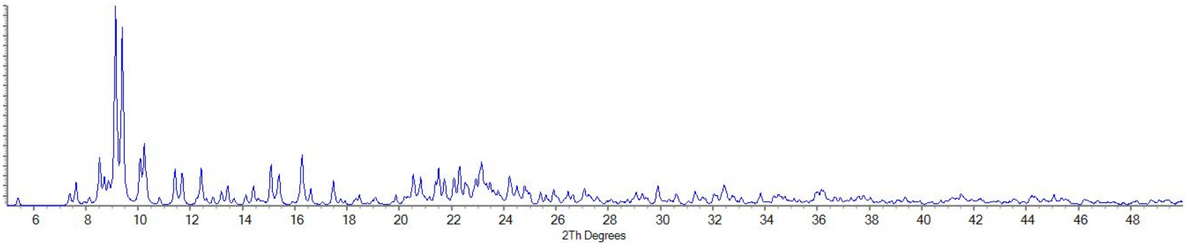

Figure S66 (a) Pawley refinement analysis of **2-Dy**. Blue experimental data, Red model, and gray difference; (b) theoretical powder X-ray diffraction pattern of **2-Dy**. Pawley refinement Rwp 1.101; Rwp'=2.587.

(a)

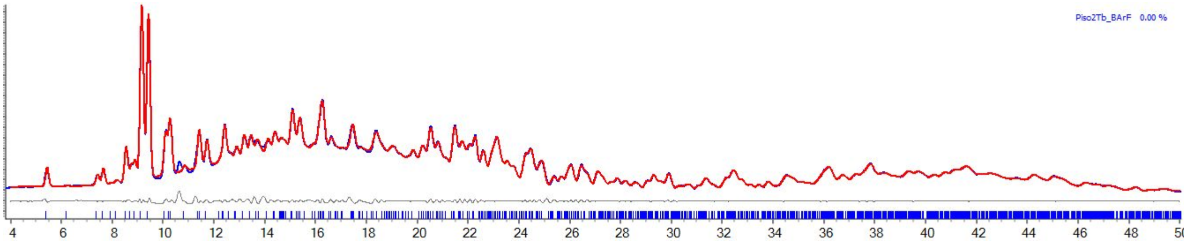

(b)

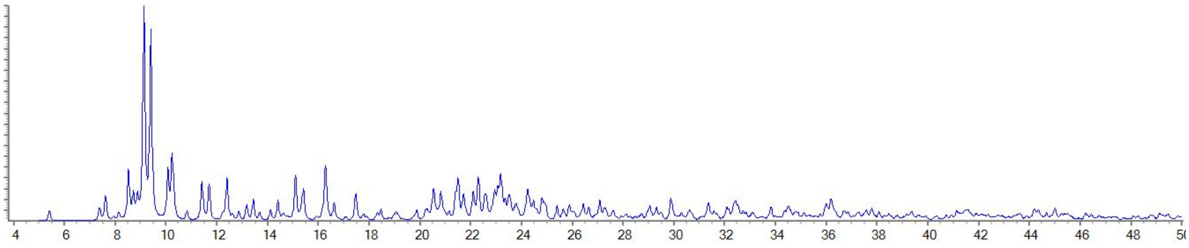

Figure S67 (a) Pawley refinement analysis of **2-Tb**. Blue experimental data, Red model, and gray difference; (b) theoretical powder X-ray diffraction pattern of **2-Tb**. Pawley refinement Rwp 1.669; Rwp'=1.595.

(a)

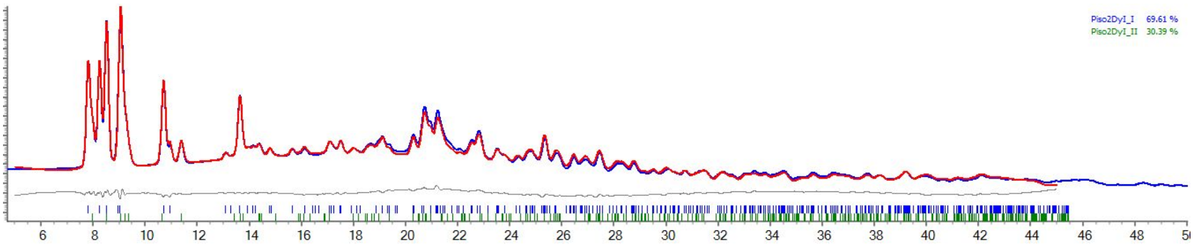

(b)

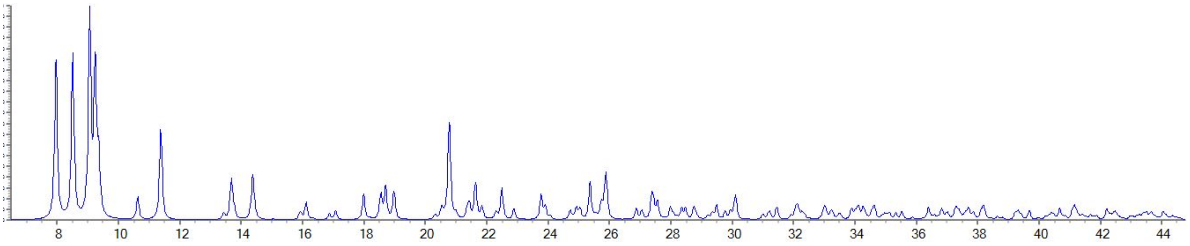

(c)

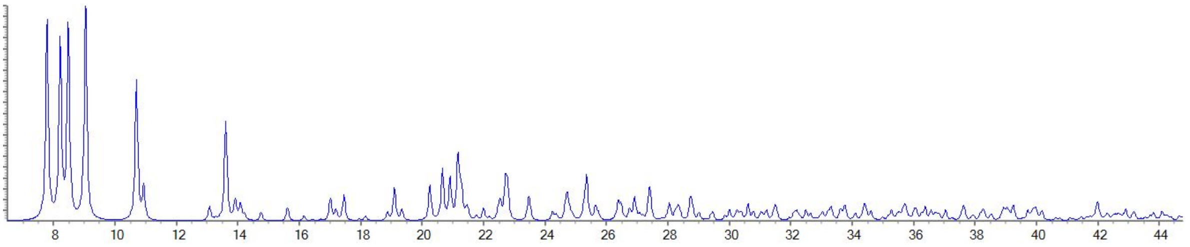

Figure S68 (a) Rietveld refinement analysis of **1-Dy**, obtaining two different compounds: **1-Dy-I** and **1-Dy-II**. Blue experimental data, Red model, and gray difference; (b) theoretical powder X-ray diffraction pattern of **1-Dy-II**; (c) theoretical model for **1-Dy-I**. The crystal structure of **1-Dy-I** was obtained using the structure of **1-Tb** as a model. No refinement of the atomic positions was done due to low resolution and the peak overlap between the two compounds. Quantitative analysis shows the presence of **1-Dy-I** in 69.61 % and **1-Dy-II in** 30.39% (Rietveld refinement Rwp 2.357; Rwp'= 9.618).

(a)

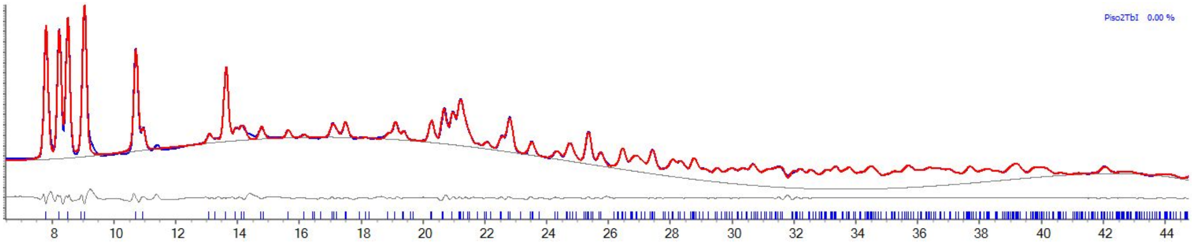

(b)

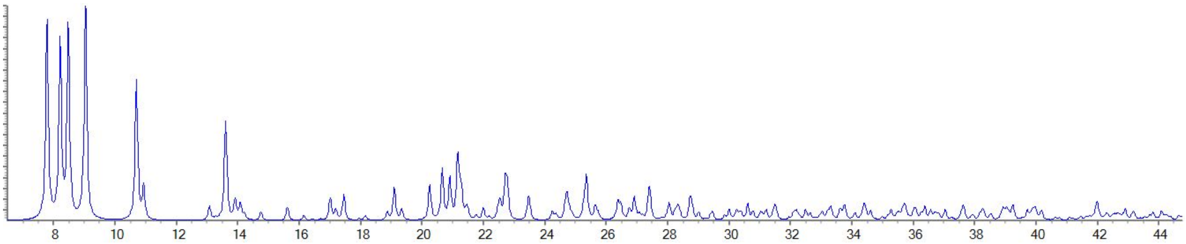

Figure S69 (a) Pawley refinement analysis of **1-Tb**. Blue experimental data, Red model, and grey difference; (b) theoretical powder X-ray diffraction pattern of **1-Tb**. Pawley refinement Rwp 1.698; Rwp'=7.069.

Table S5 Unit cell values obtained from Pawley refinement results.

Compound	a	b	c	α	β	Γ
**3-Dy**	12.340(6)	23.95(1)	20.563(9)	90	99.34(1)	90
**3-Tb**	12.37(1)	23.91(2)	20.53(1)	90	99.68(2)	90
**2-Dy**	13.188(5)	28.50(1)	19.957(7)	90	91.545(4)	90
**2-Tb**	13.207(4)	28.602(9)	19.985(6)	90	91.685(7)	90
**1-Dy-I**	23.853(2)	10.983(1)	25.190(2)	90	115.972(5)	90
**1-Dy-II**	23.992(4)	10.840(2)	22.396(3)	90	111.95(1)	90
**1-Tb**	23.89(1)	10.975(5)	25.21(1)	90	115.965(3)	90

6. Magnetism

The equilibrium susceptibilities were measured on cooling in temperature settle mode, with rates of 5 K/min between 300 and 100 K, 2 K/min 100-10 K and 1 K/min below 10 K (Figures S70-S75). The zero-field cooled (ZFC) and field cooled (FC) measurements were performed in temperature sweep mode with a sweep rate of 1 K/min. The ZFC samples were prepared by cooling from at least 150 K in zero field for **3-Dy** and **3-Tb**, and holding at 2 K for 0.5 hrs before rapidly ramping the dc field at 200 Oe/s to 0.1 T and beginning the measurement on warming. The FC measurements were performed on cooling and warming modes at the same rate (Figures S76-S81). Hysteresis measurements were performed between +4 and -4 T in field sweep mode with sweep rates of 22 Oe/s (Figures S145-S150). Magnetization measurements were performed between 0-7 T at 2 K in field settle mode. The field was ramped at 500 Oe/s between points and held constant for at least 5 min before measuring (Figures S139-S144). All ac data were collected with five measuring points, using an oscillating field amplitude of H_{ac}= 5 Oe for 0.1-600 Hz and 2 Oe for 700-1000 Hz. Data were collected at 8 log-spaced frequencies per decade in the range 0.1-1000 Hz, averaging over 2 sec or 10 cycles, whichever was longer. ac data were fit to the Generalized Debye (GD) model in CC-FIT2 to extract relaxation rates and distributions, unless stated otherwise (Figures S82-S115, Tables S6-S16).[1,6]

6.1 Temperature-swept magnetic measurements

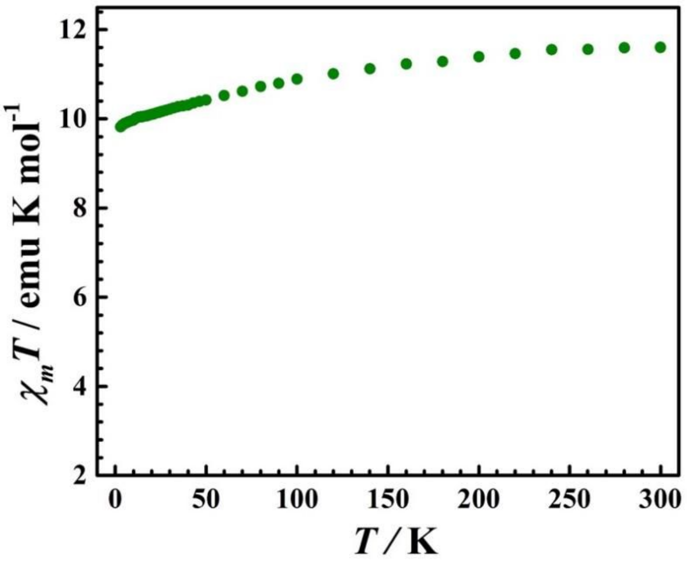

Figure S70 Temperature dependent susceptibility of $\chi_m T$ in an applied dc magnetic field of 1 kOe for **1-Tb**.

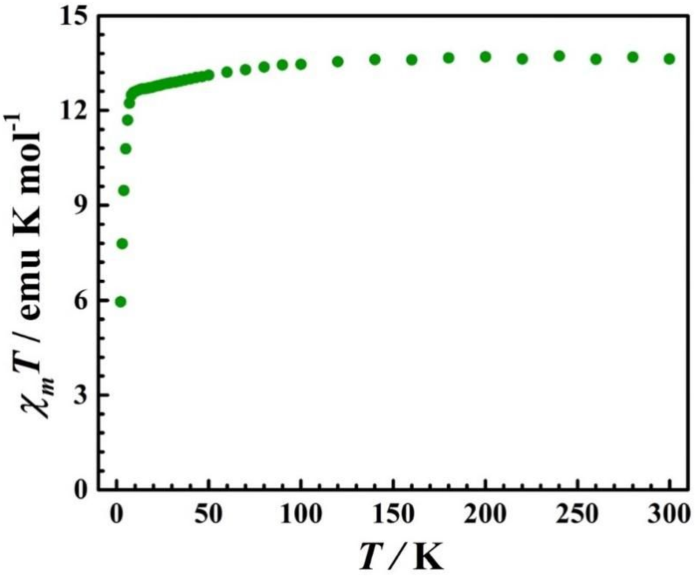

Figure S71 Temperature dependent susceptibility of $\chi_m T$ in an applied dc magnetic field of 1 kOe for **1-Dy**.

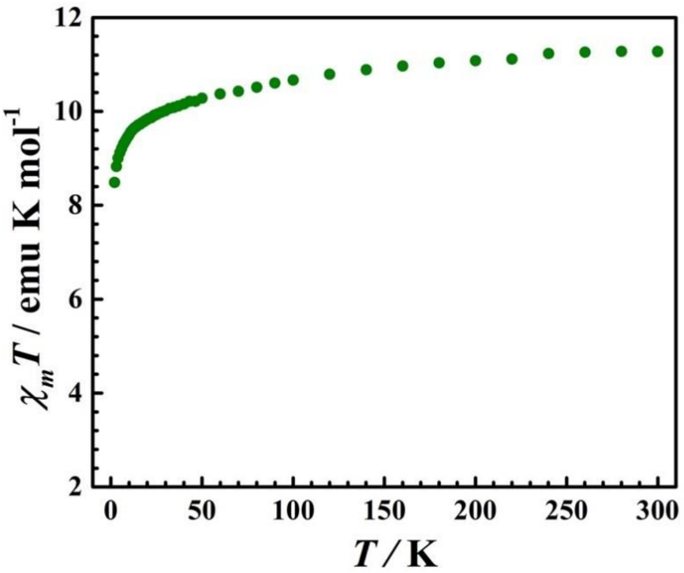

Figure S72 Temperature dependent susceptibility of $\chi_m T$ in an applied dc magnetic field of 1 kOe for **2-Tb**.

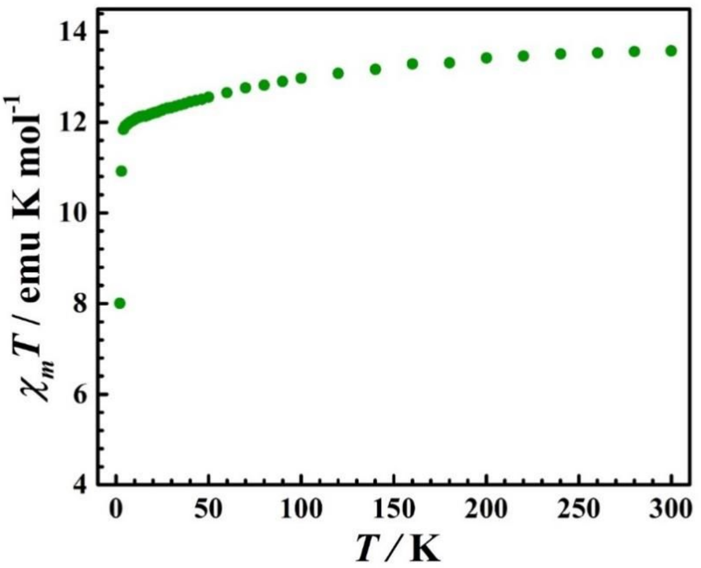

Figure S73 Temperature dependent susceptibility of $\chi_m T$ in an applied dc magnetic field of 1 kOe for **2-Dy**.

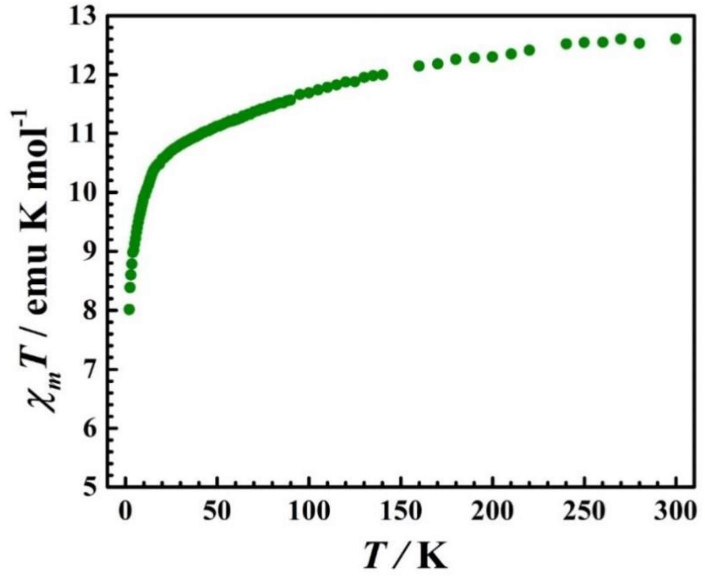

Figure S74 Temperature dependent susceptibility of $\chi_m T$ in an applied dc magnetic field of 1 kOe for **3-Tb**.

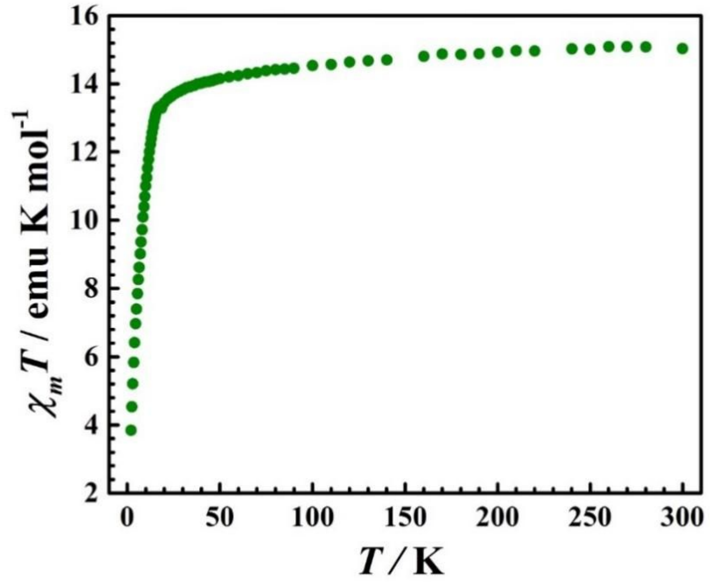

Figure S75 Temperature dependent susceptibility of $\chi_m T$ in an applied dc magnetic field of 1 kOe for **3-Dy**.

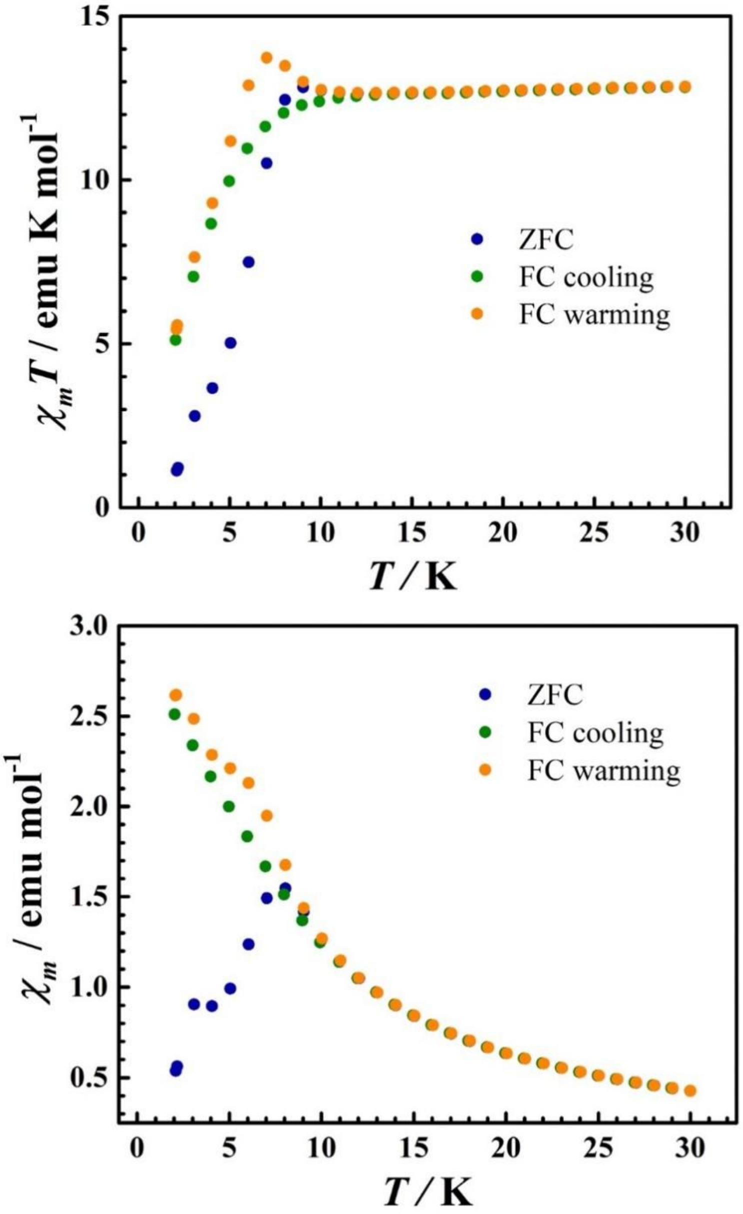

Figure S76 The χT (top) and χ (bottom) products as a function of temperature field-cooled (FC, green points) and zero-field-cooled (ZFC, blue points) variable-temperature magnetic susceptibility for **1-Dy** with 1 kOe dc field in warm mode from 2 to 30 K. The orange points represent FC variable-temperature magnetic susceptibility in cool mode from 30 to 2 K.

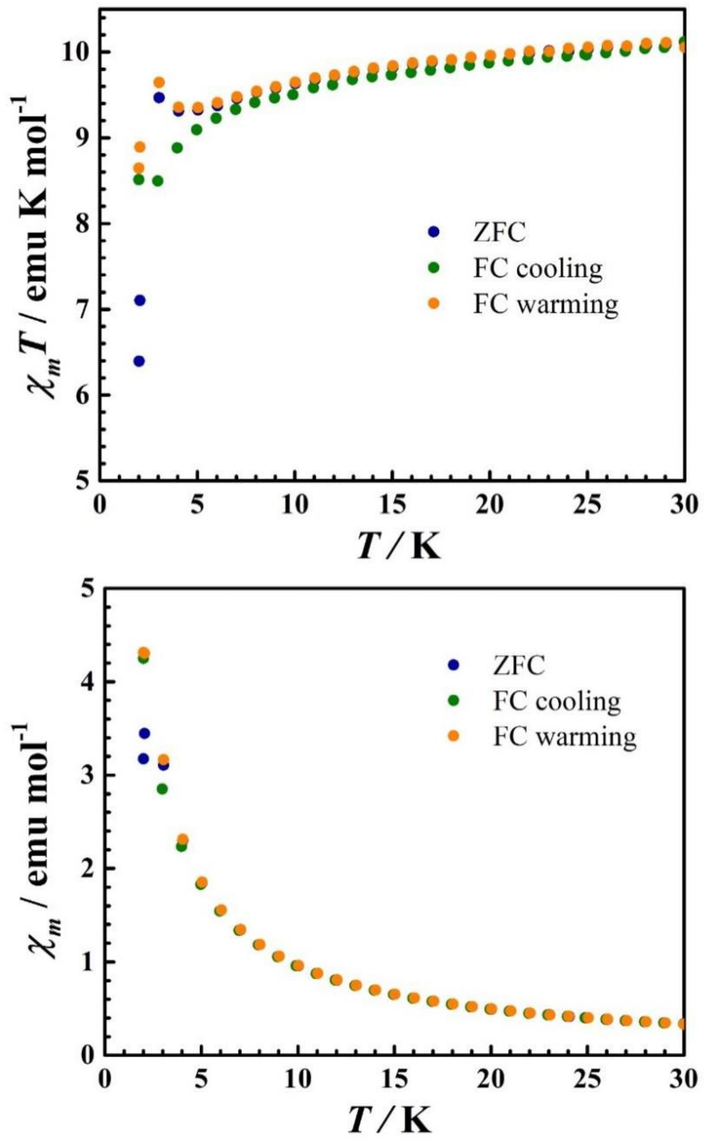

Figure S77 The χT (top) and χ (bottom) products as a function of temperature field-cooled (FC, green points) and zero-field-cooled (ZFC, blue points) variable-temperature magnetic susceptibility for **2-Tb** with 1 kOe dc field in warm mode from 2 to 30 K. The orange points represent FC variable-temperature magnetic susceptibility in cool mode from 30 to 2 K.

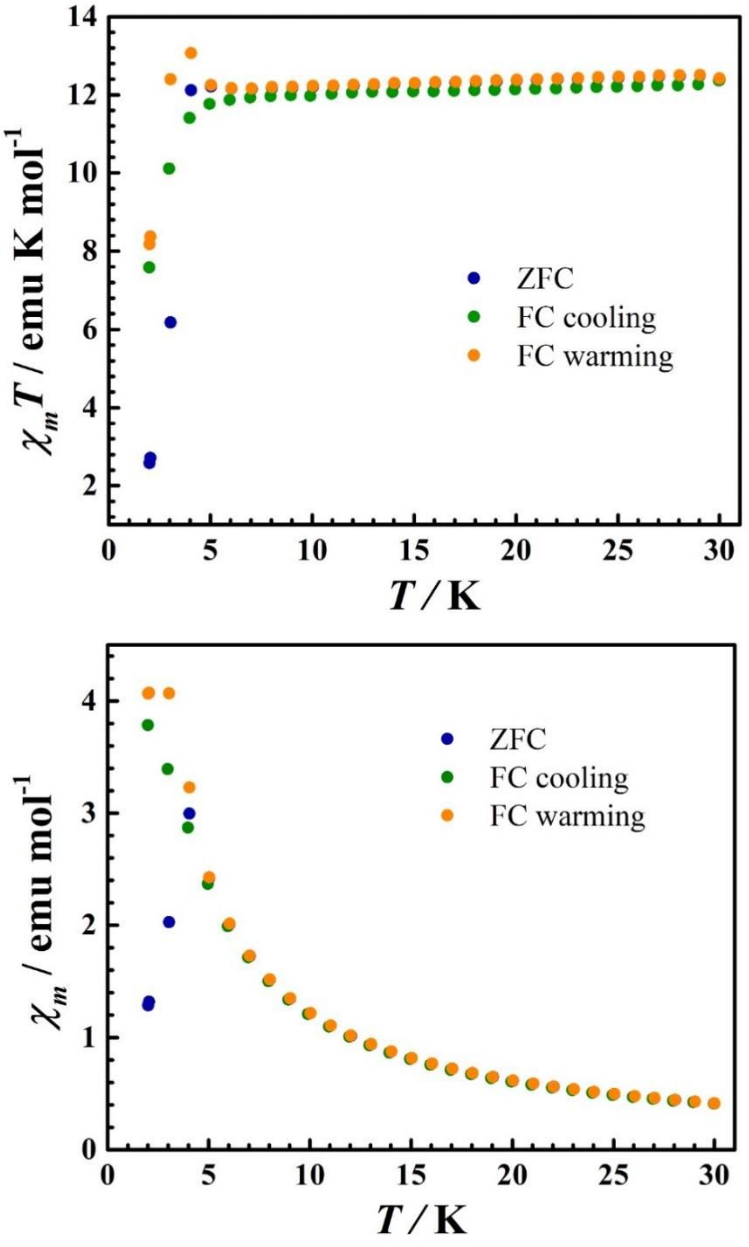

Figure S78 The χT (top) and χ (bottom) products as a function of temperature field-cooled (FC, green points) and zero-field-cooled (ZFC, blue points) variable-temperature magnetic susceptibility for **2-Dy** with 1 kOe dc field in warm mode from 2 to 30 K. The orange points represent FC variable-temperature magnetic susceptibility in cool mode from 30 to 2 K.

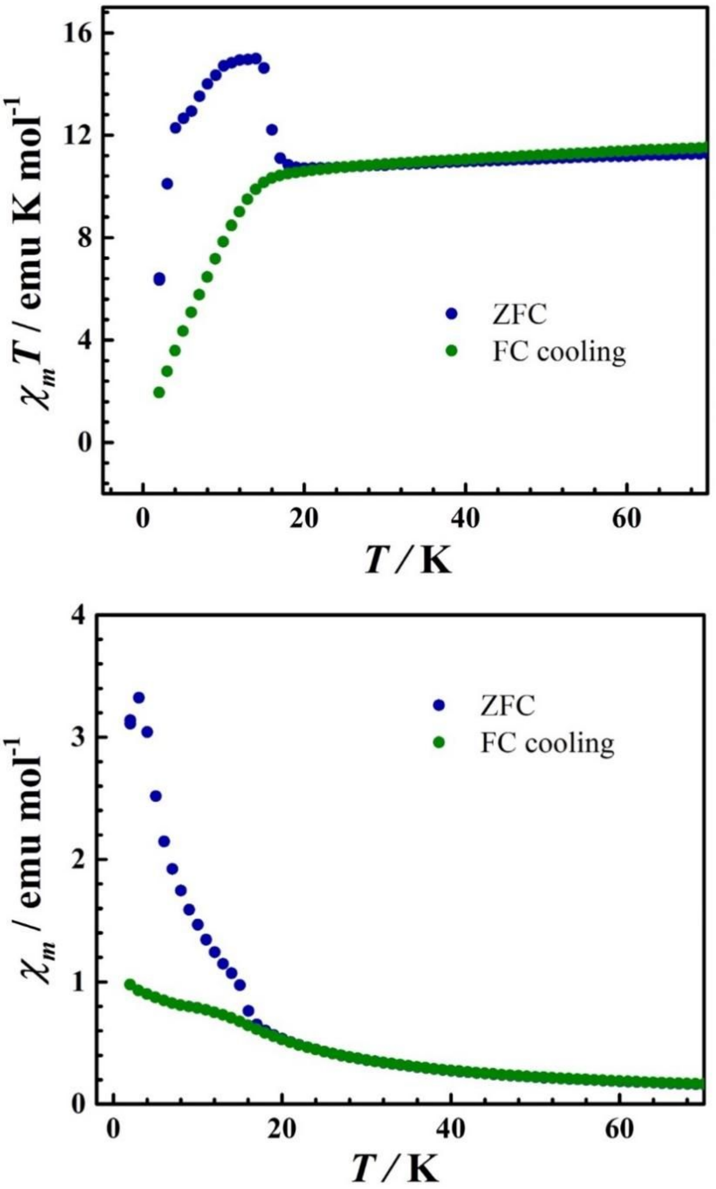

Figure S79 The χT (top) and χ (bottom) products as a function of temperature field-cooled (FC, green points) and zero-field-cooled (ZFC, blue points) variable-temperature magnetic susceptibility for **3-Tb** with 1 kOe dc field in warm mode from 2 to 70 K.

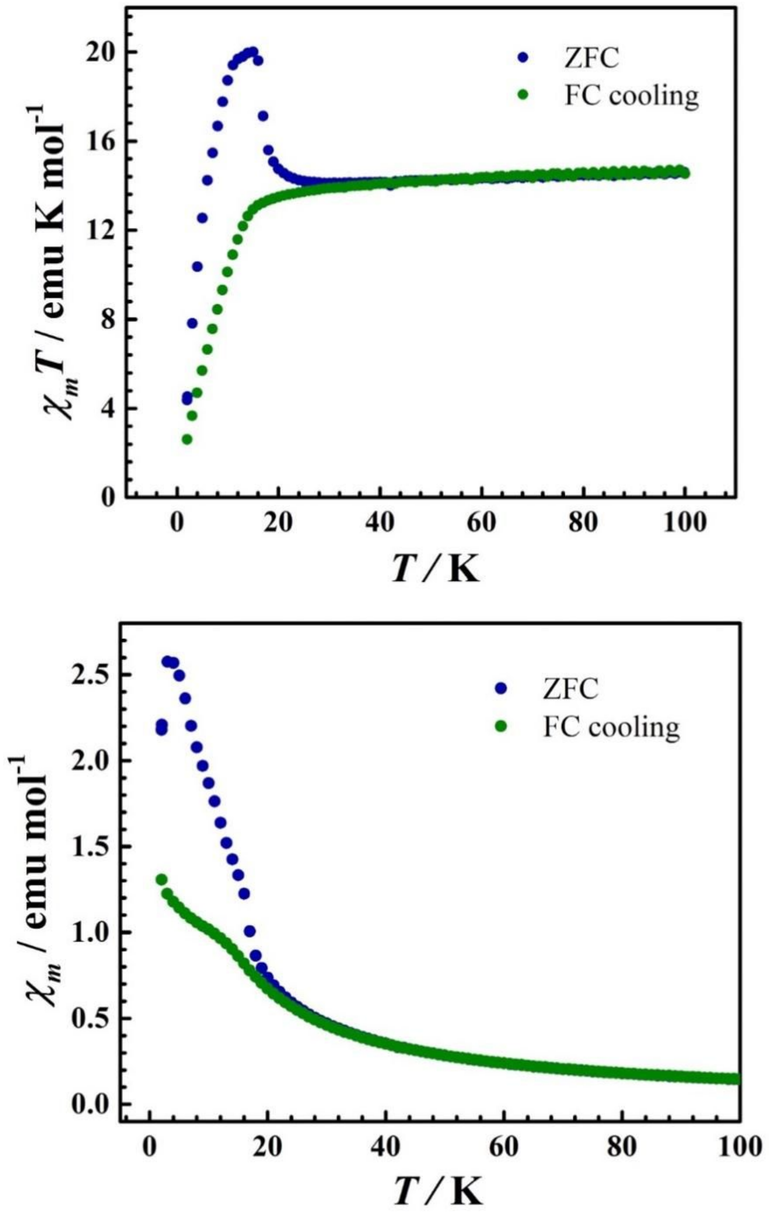

Figure S80 The χT (top) and χ (bottom) products as a function of temperature field-cooled (FC, green points) and zero-field-cooled (ZFC, blue points) variable-temperature magnetic susceptibility for **3-Dy** with 1 kOe dc field in warm mode from 2 to 100 K.

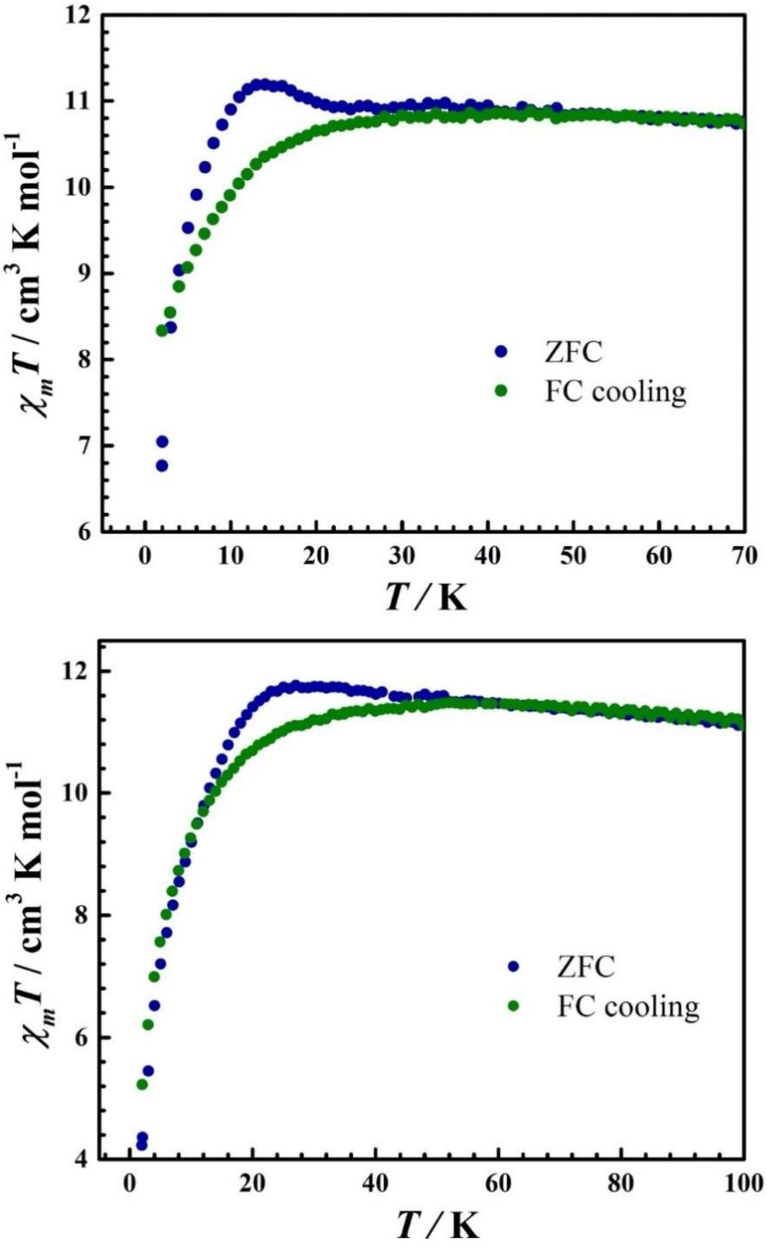

Figure S81 The $\chi_m T$ products as a function of temperature field-cooled (FC, green points) and zero-field-cooled (ZFC, blue points) variable-temperature magnetic susceptibility for a 50 mM solution of **3-Tb** (top) and **3-Dy** (bottom) in hexane with 1 kOe dc field in warm mode from 2 to 70 or 100 K.

6.2 Ac magnetic measurements

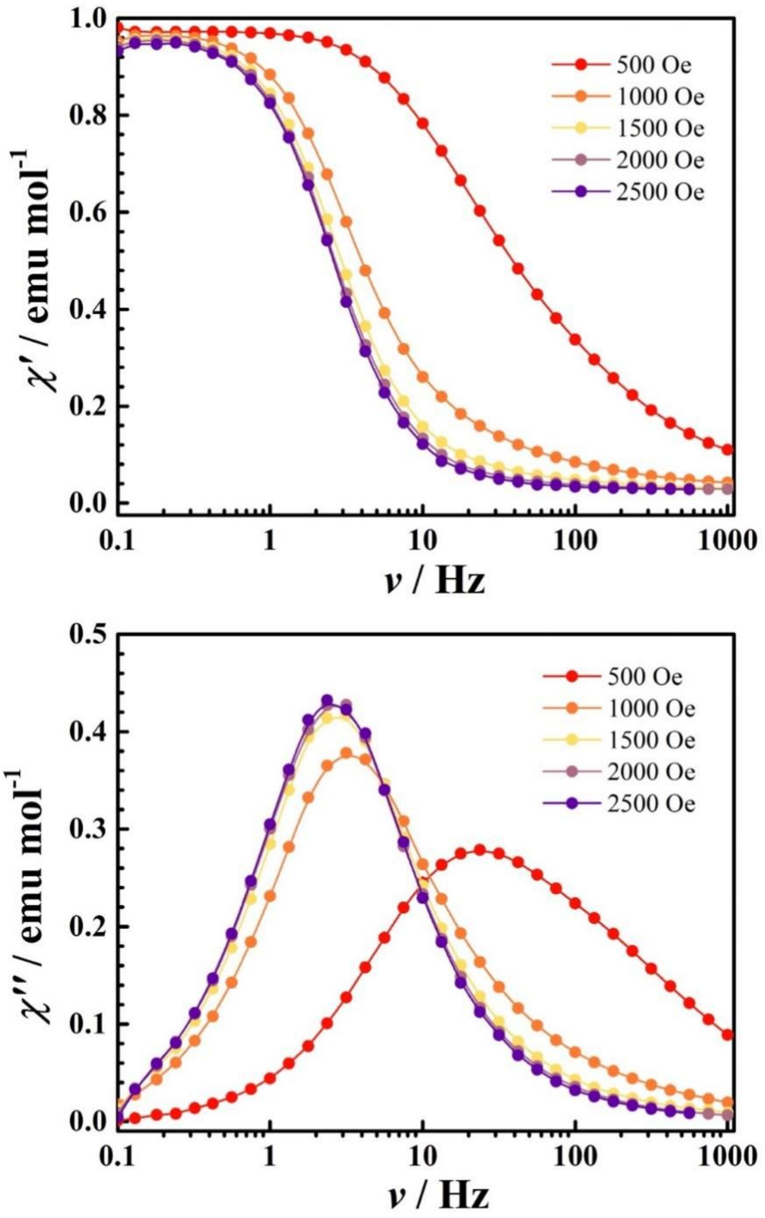

Figure S82 In-phase (χ', top) and out-of-phase (χ'', bottom) components of the ac magnetic susceptibility for **1-Tb** in dc magnetic fields of 500-2500 Oe at 10 K. The solid lines are guides for the eyes.

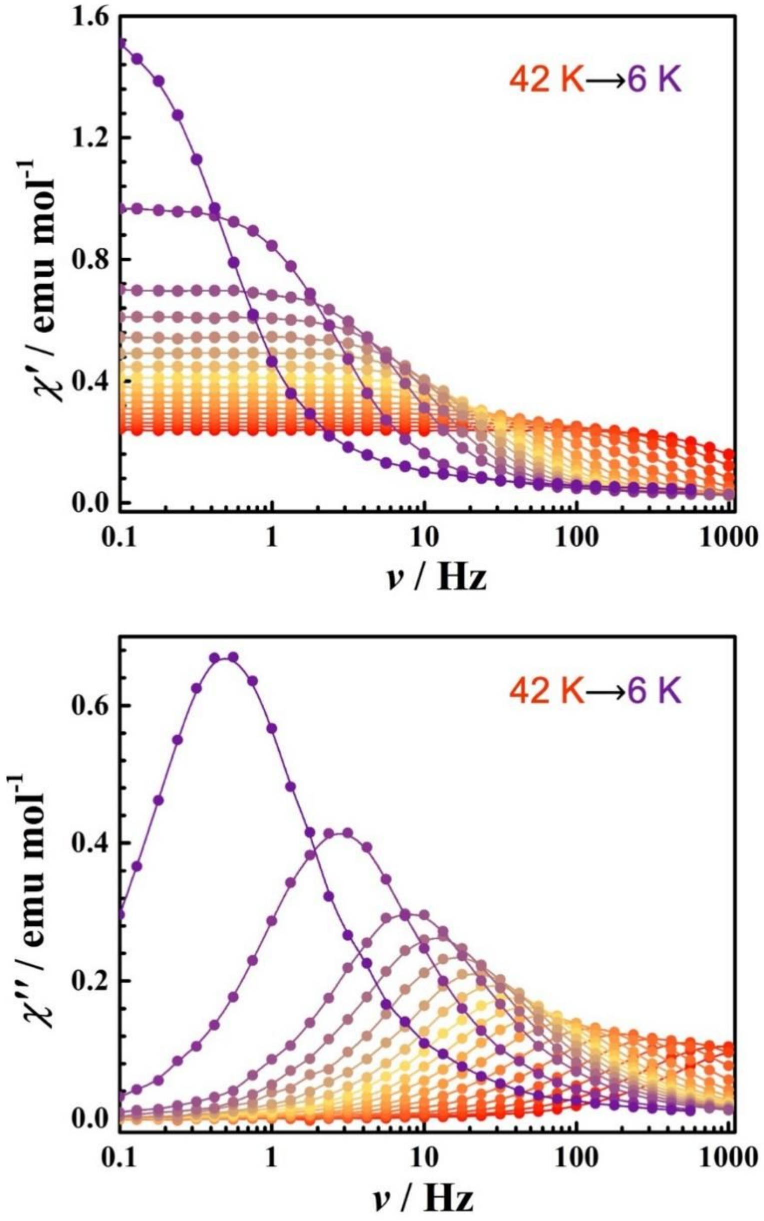

Figure S83 In-phase (χ', top) and out-of-phase (χ'', bottom) components of the ac magnetic susceptibility for **1-Tb** under 1500 Oe applied dc field at frequencies ranging from 0.1-1000 Hz and temperatures from 42-6 K. The colored lines are guides for the eye.

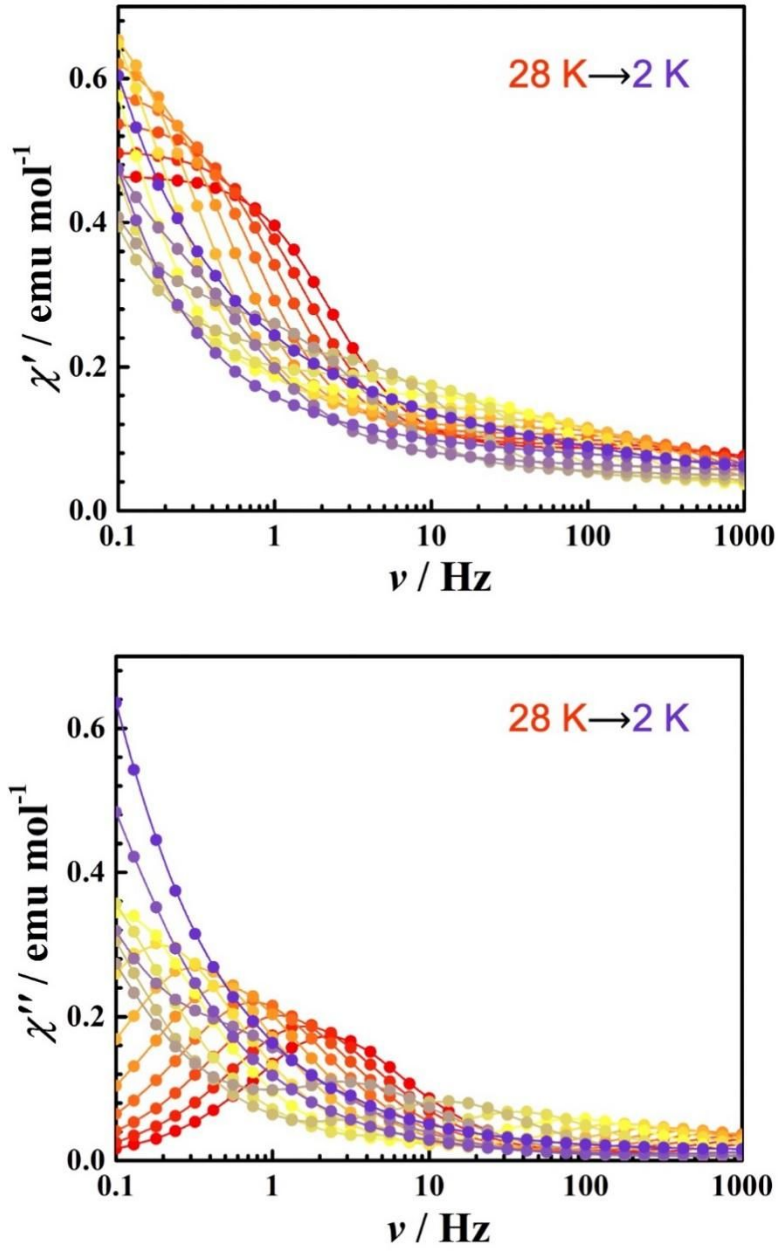

Figure S84 In-phase (χ', top) and out-of-phase (χ'', bottom) components of the ac magnetic susceptibility for **1-Dy** under zero applied dc field at frequencies ranging from 0.1-1000 Hz and temperatures from 28-2 K. The colored lines are guides for the eye.

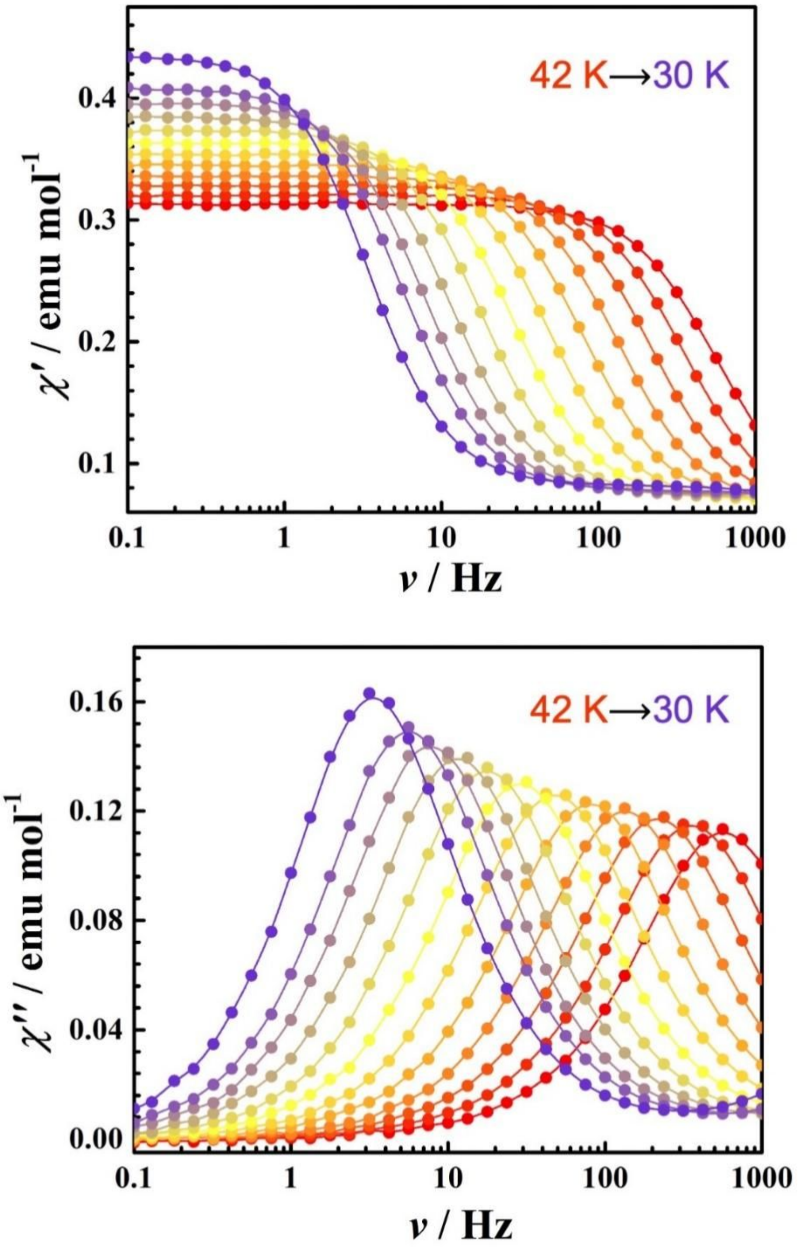

Figure S85 In-phase (χ', top) and out-of-phase (χ'', bottom) components of the ac magnetic susceptibility for **1-Dy** under zero applied dc field at frequencies ranging from 0.1-1000 Hz and temperatures from 42-30 K. The colored lines are guides for the eye.

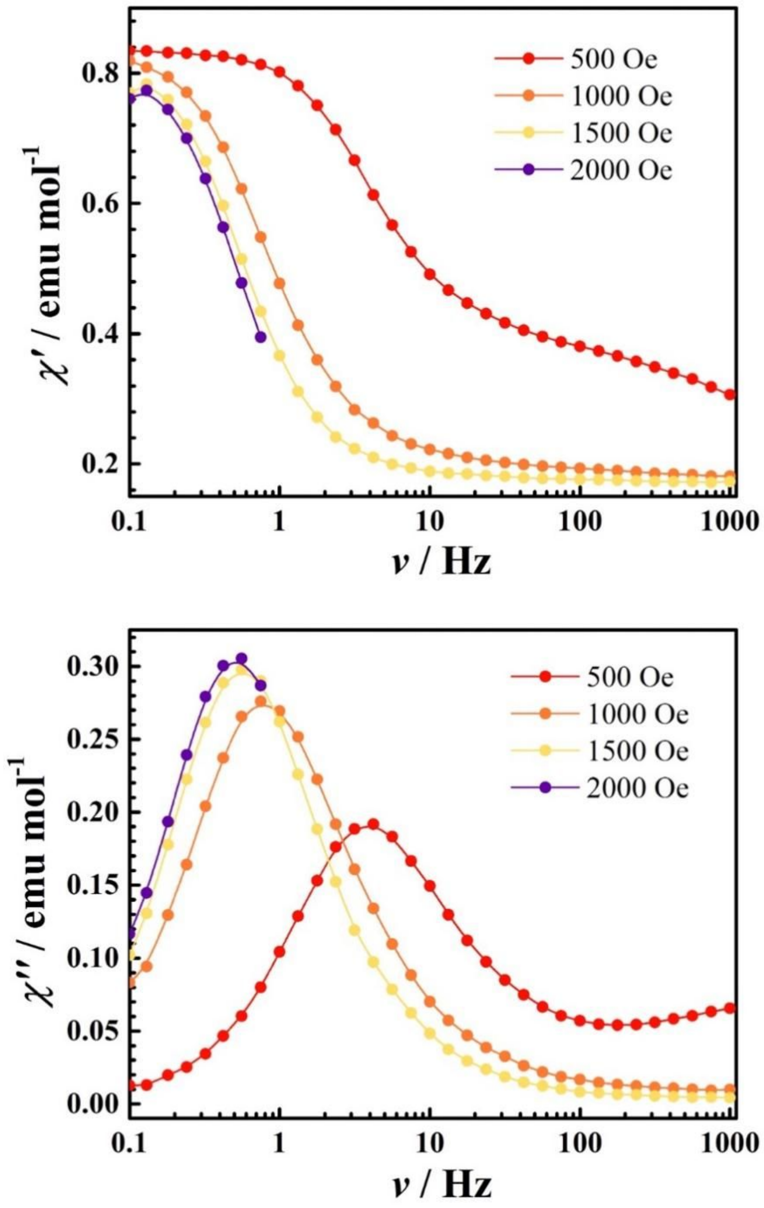

Figure S86 In-phase (χ', top) and out-of-phase (χ'', bottom) components of the ac magnetic susceptibility for **2-Tb** in the dc magnetic field of 500-2000 Oe at 10 K. The solid lines are guides for the eyes.

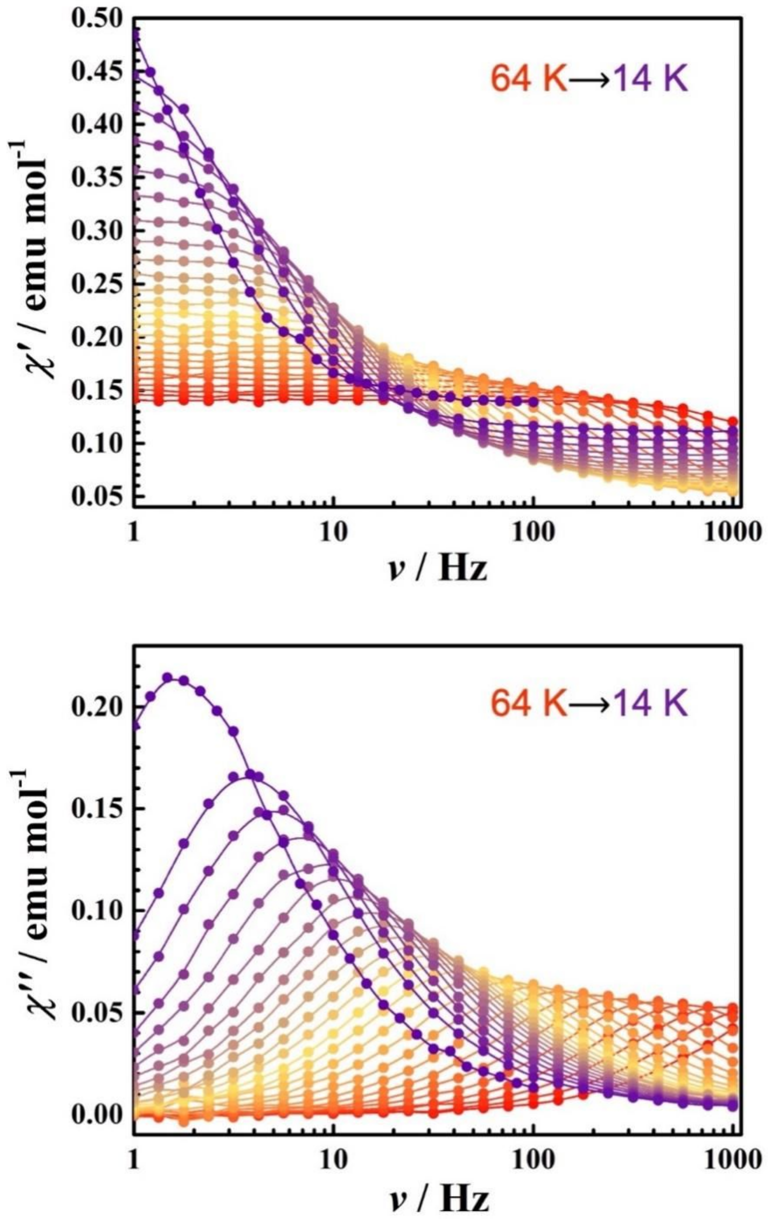

Figure S87 In-phase (χ', top) and out-of-phase (χ'', bottom) components of the ac magnetic susceptibility for **2-Tb** under 1500 Oe applied dc field at frequencies ranging from 1-1000 Hz and temperatures from 64-14 K. The colored lines are guides for the eye.

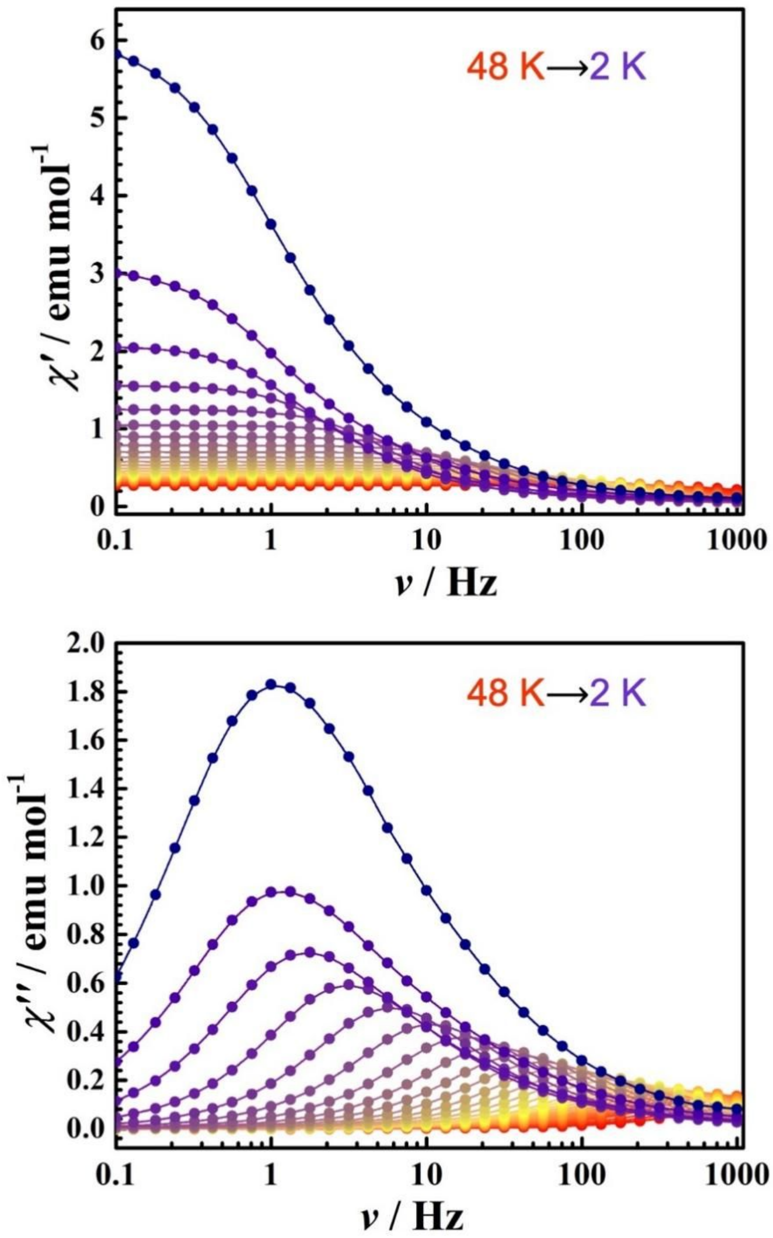

Figure S88 In-phase (χ', top) and out-of-phase (χ'', bottom) components of the ac magnetic susceptibility for **2-Dy** under zero applied dc field at frequencies ranging from 0.1-1000 Hz and temperatures from 48-2 K. The colored lines are guides for the eye.

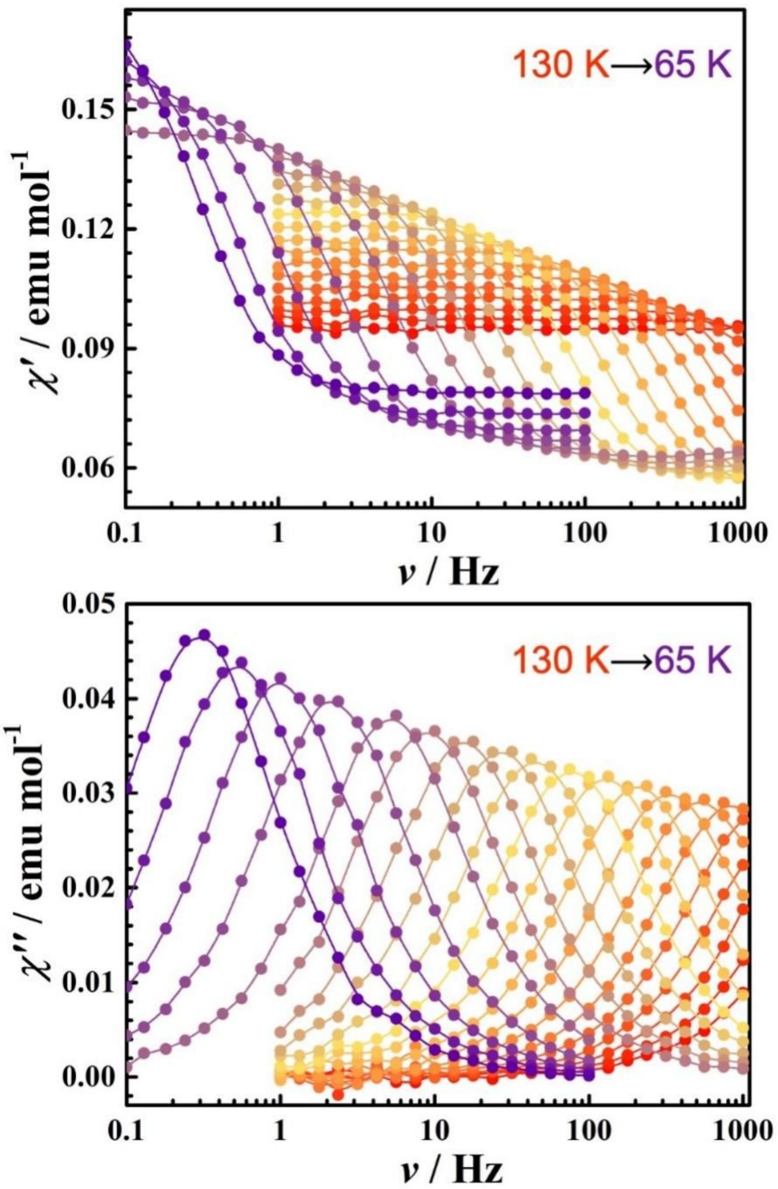

Figure S89 In-phase (χ', top) and out-of-phase (χ'', bottom) components of the ac magnetic susceptibility for **3-Tb** under zero applied dc field at frequencies ranging from 0.1-1000 Hz and temperatures from 130-65 K. The colored lines are guides for the eye.

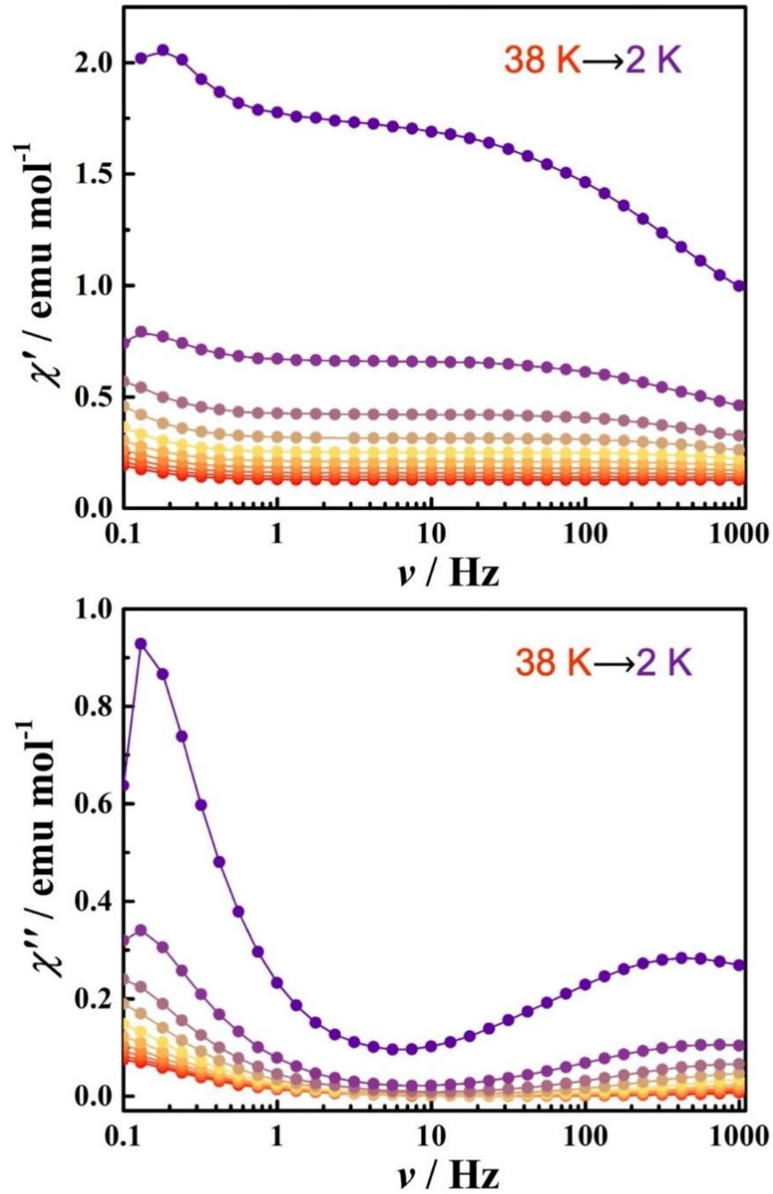

Figure S90 In-phase (χ', top) and out-of-phase (χ'', bottom) components of the ac magnetic susceptibility for **3-Tb** under zero applied dc field at frequencies ranging from 0.1-1000 Hz and temperatures from 38-2 K. The colored lines are guides for the eye.

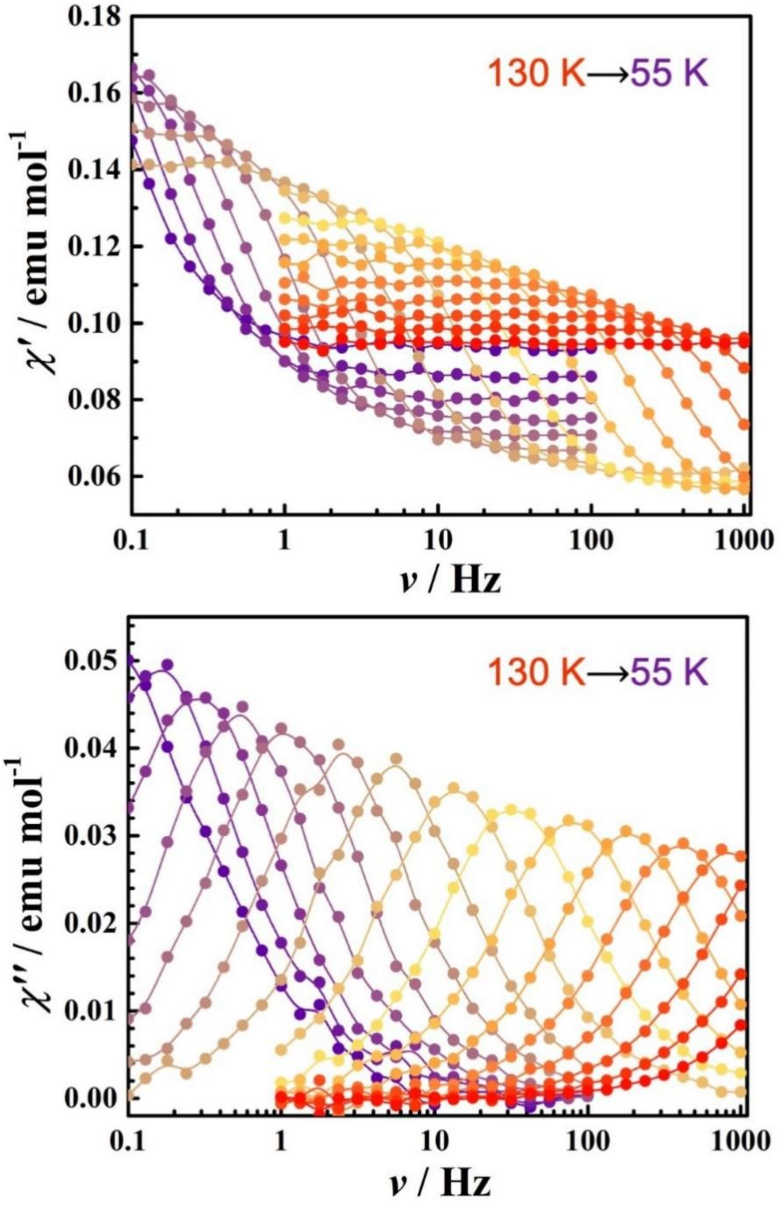

Figure S91 In-phase (χ', top) and out-of-phase (χ'', bottom) components of the ac magnetic susceptibility for **3-Tb** under 1500 Oe applied dc field at frequencies ranging from 0.1-1000 Hz and temperatures from 130-55 K. The colored lines are guides for the eye.

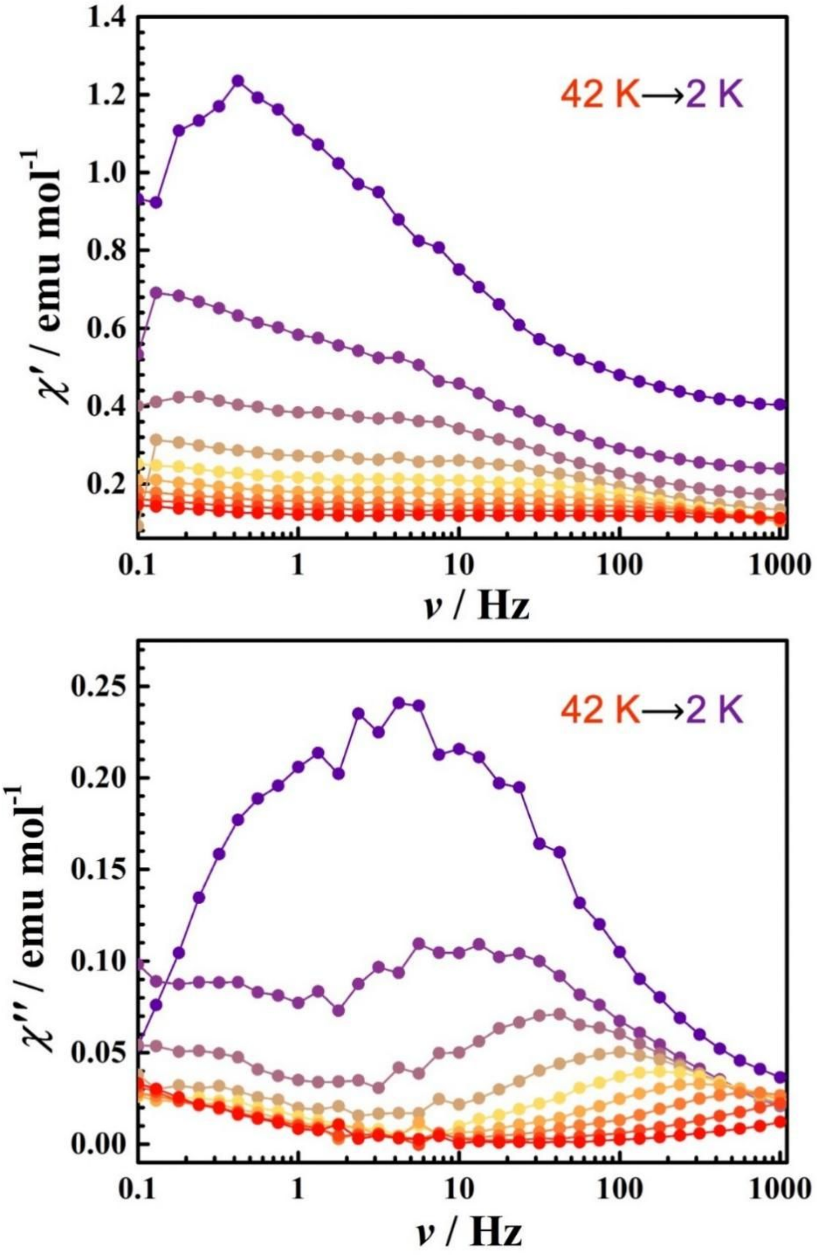

Figure S92 In-phase (χ', top) and out-of-phase (χ'', bottom) components of the ac magnetic susceptibility for **3-Tb** under 1500 Oe applied dc field at frequencies ranging from 0.1-1000 Hz and temperatures from 42-2 K. The colored lines are guides for the eye.

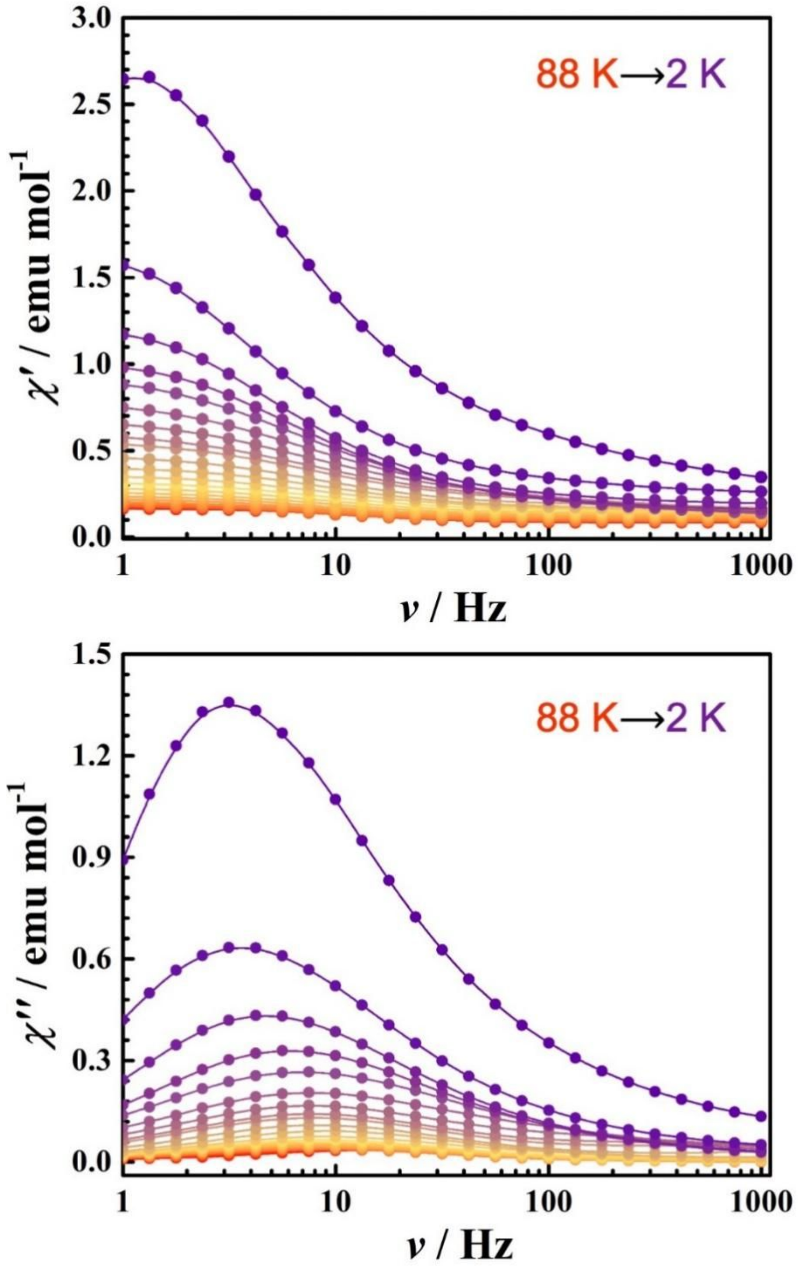

Figure S93 In-phase (χ', top) and out-of-phase (χ'', bottom) components of the ac magnetic susceptibility for **3-Dy** under zero applied dc field at frequencies ranging from 1-1000 Hz and temperatures from 88-2 K. The colored lines are guides for the eye.

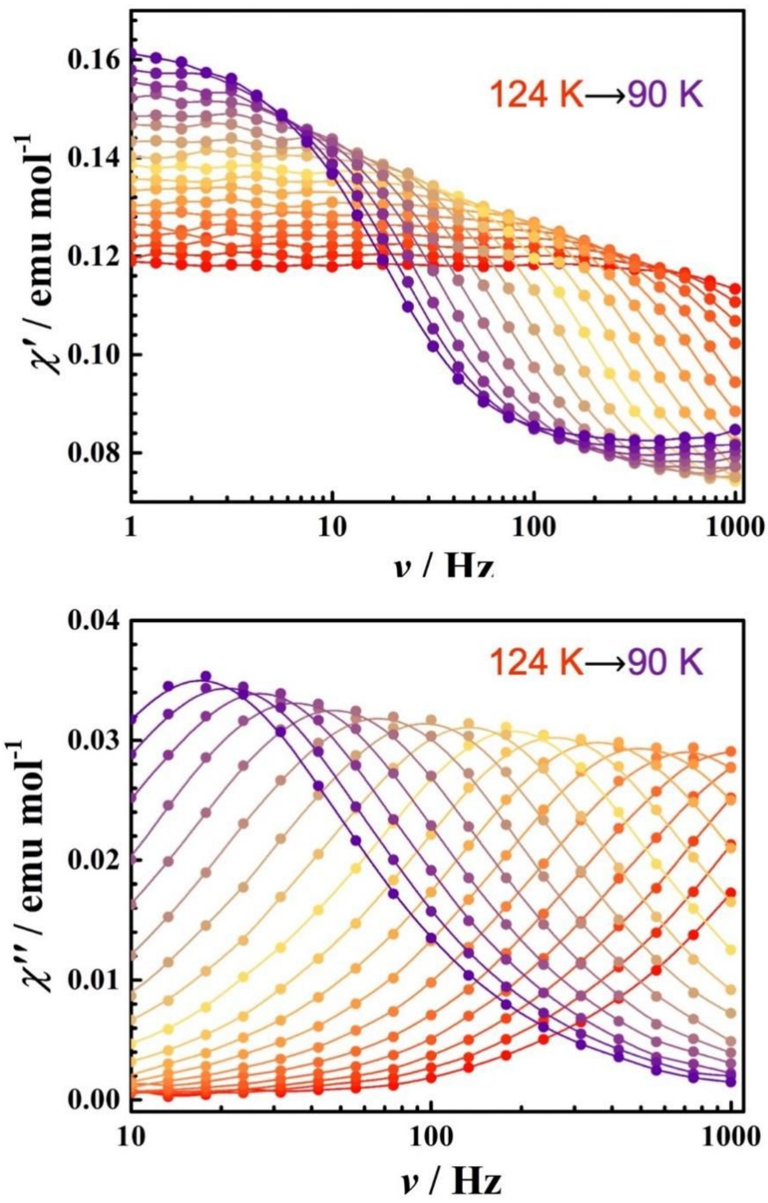

Figure S94 In-phase (χ', top) and out-of-phase (χ'', bottom) components of the ac magnetic susceptibility for **3-Dy** under zero applied dc field at frequencies ranging from 10-1000 Hz and temperatures from 124-90 K. The colored lines are guides for the eye.

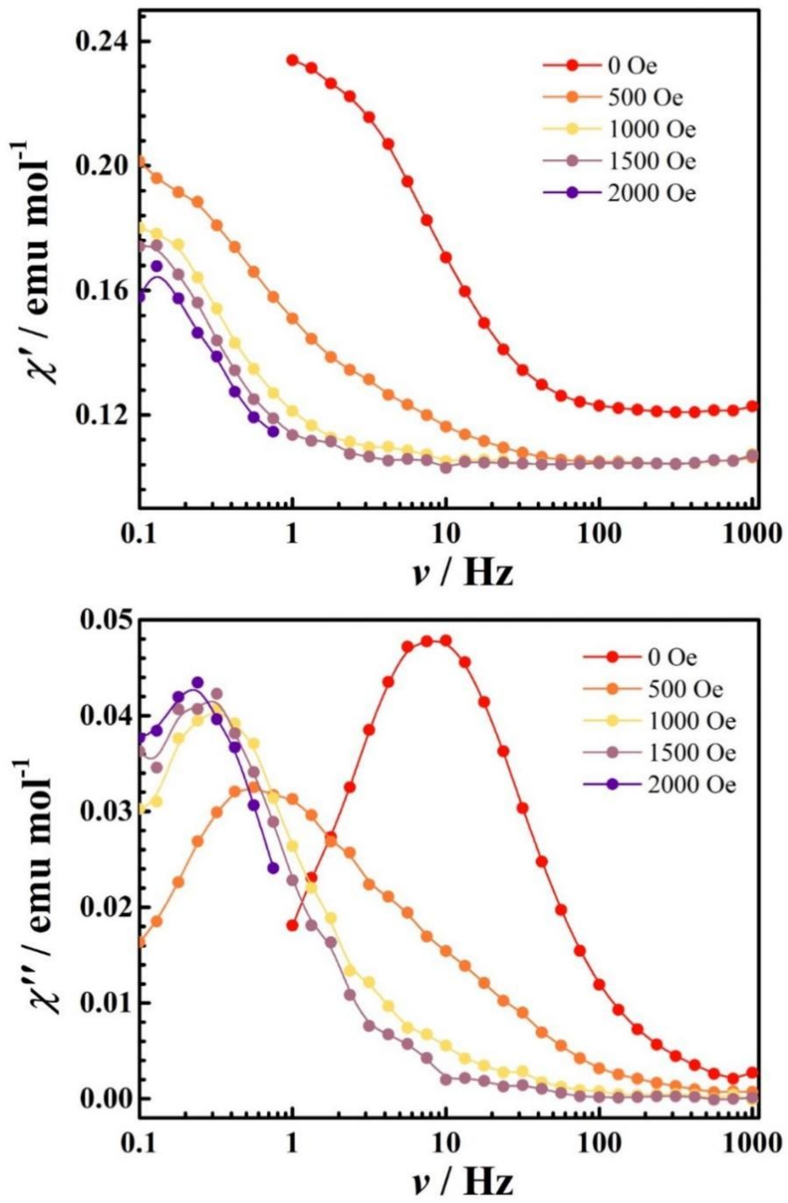

Figure S95 In-phase (χ', top) and out-of-phase (χ'', bottom) components of the ac magnetic susceptibility for **3-Dy** in dc magnetic fields of 0-2000 Oe at 60 K. The solid lines are guides for the eyes.

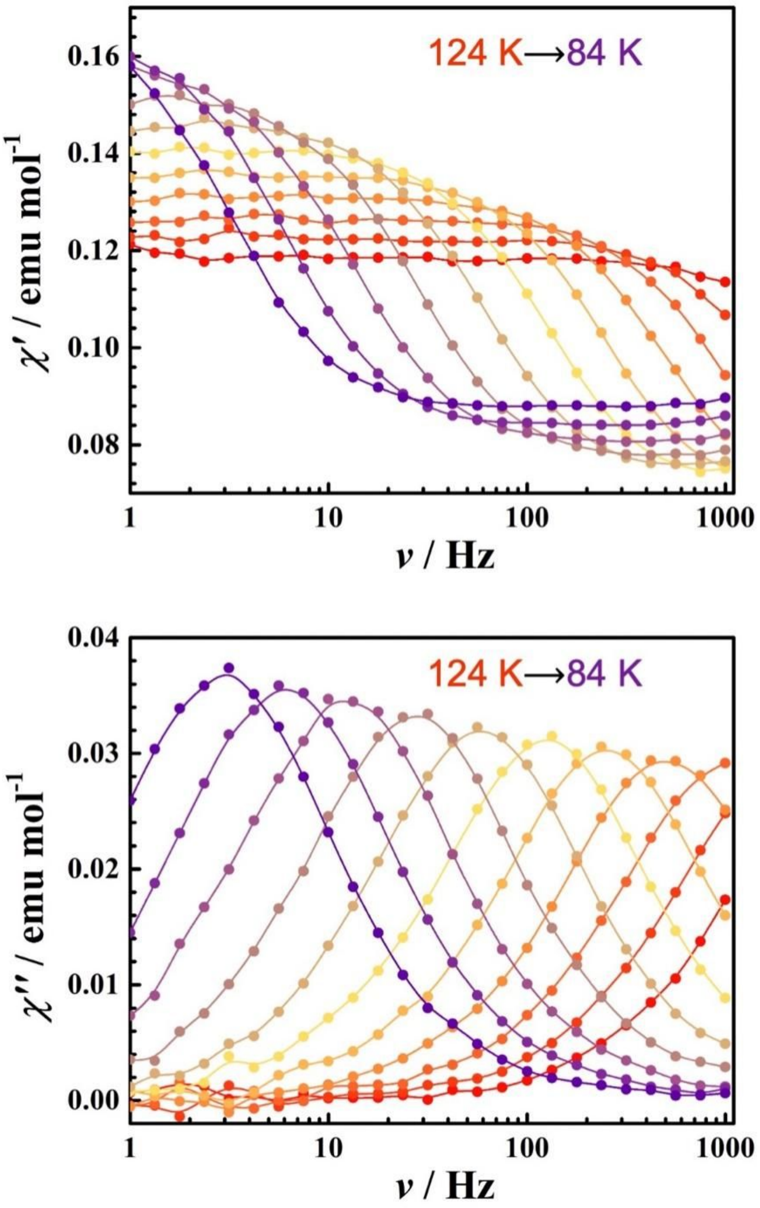

Figure S96 In-phase (χ', top) and out-of-phase (χ'', bottom) components of the ac magnetic susceptibility for **3-Dy** under 1000 Oe applied dc field at frequencies ranging from 1-1000 Hz and temperatures from 124-84 K. The colored lines are guides for the eye.

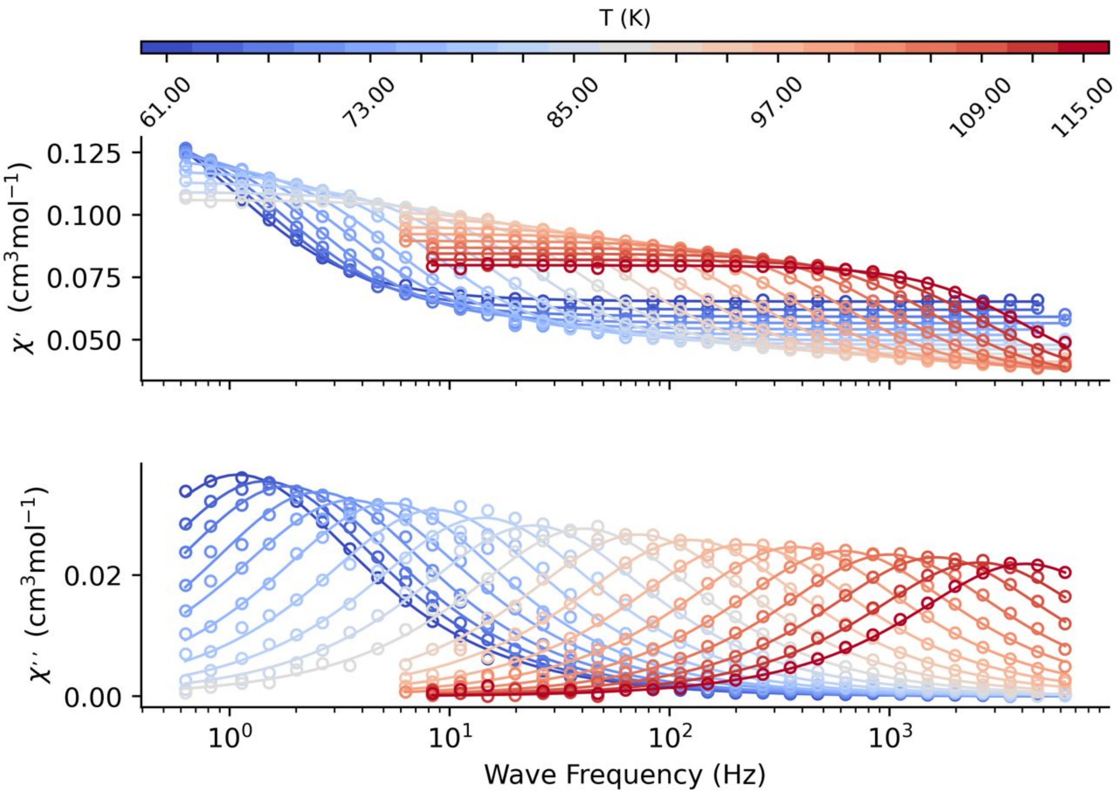

Figure S97 In-phase (χ', top) and out-of-phase (χ'', bottom) components of the ac magnetic susceptibility for a 50 mM solution of **3-Tb** in hexane under 0 Oe applied dc field at frequencies ranging from 1-1000 Hz and temperatures from 124-84 K. The colored lines are fits to generalized Debye model in CCFIT2[1,6].

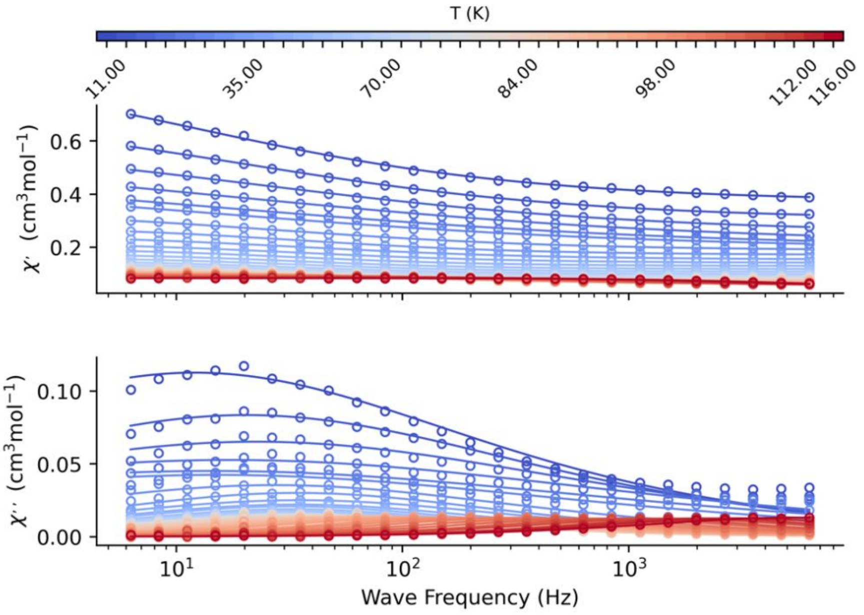

Figure S98 In-phase (χ', top) and out-of-phase (χ'', bottom) components of the ac magnetic susceptibility for a 50 mM solution of **3-Dy** in hexane under 0 Oe applied dc field at frequencies ranging from 10-1000 Hz and temperatures from 116-11 K. The colored lines are fits to generalized Debye model in CCFIT2[1,6].

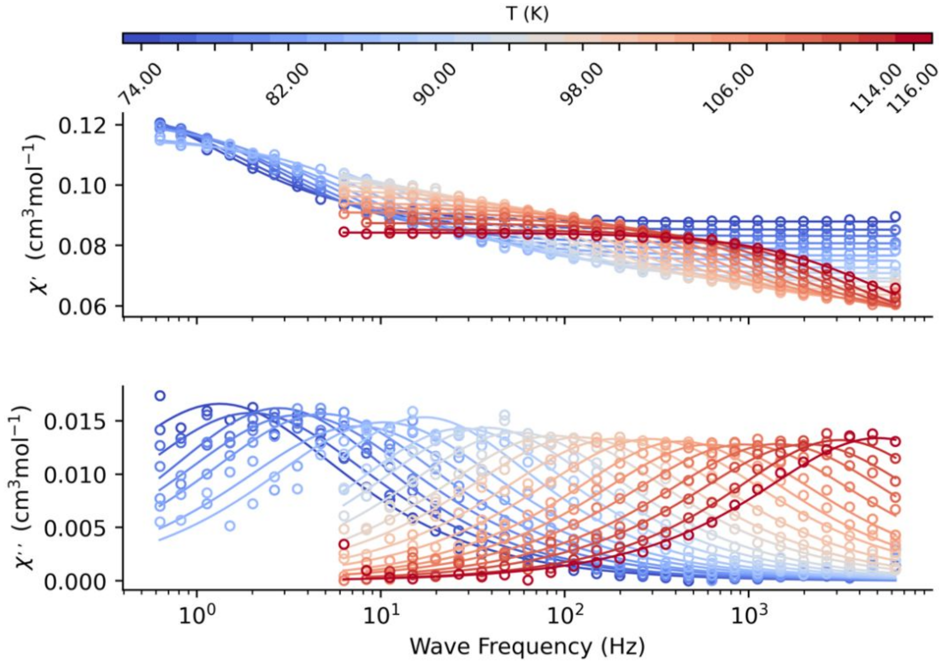

Figure S99 In-phase (χ', top) and out-of-phase (χ'', bottom) components of the ac magnetic susceptibility for a 50 mM solution of **3-Dy** in hexane under 1000 Oe applied dc field at frequencies ranging from 1-1000 Hz and temperatures from 116-74 K. The colored lines are fits to generalized Debye model in CCFIT2[1,6].

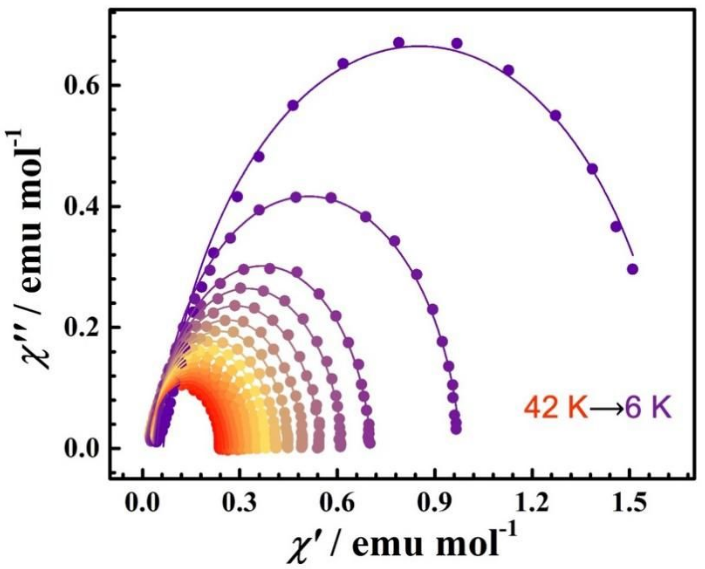

Figure S100 Cole-Cole plots for **1-Tb** from 42-6 K under 1500 Oe applied dc field. The colored lines are fits to generalized Debye model.

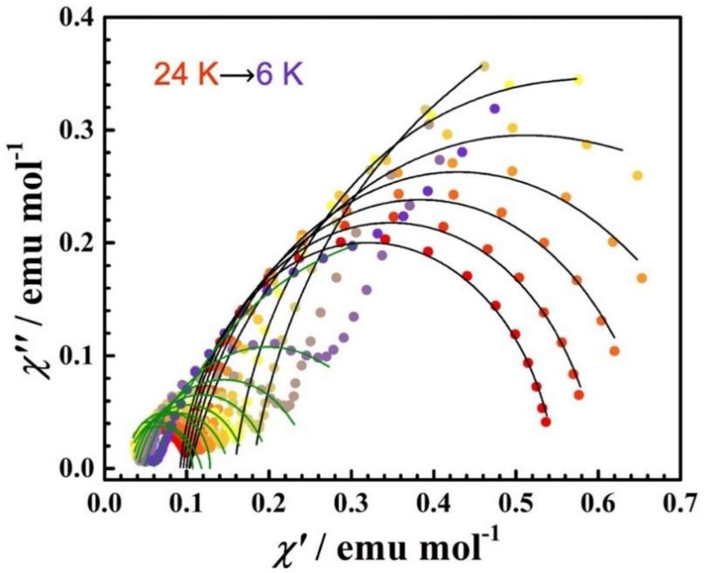

Figure S101 Cole-Cole plots for **1-Dy** from 24-6 K under zero applied dc field. The green and black lines are the best fits for fast and slow relaxation process respectively. The two relaxation plots are fits to generalized Debye model separately instead of using double generalized Debye model.

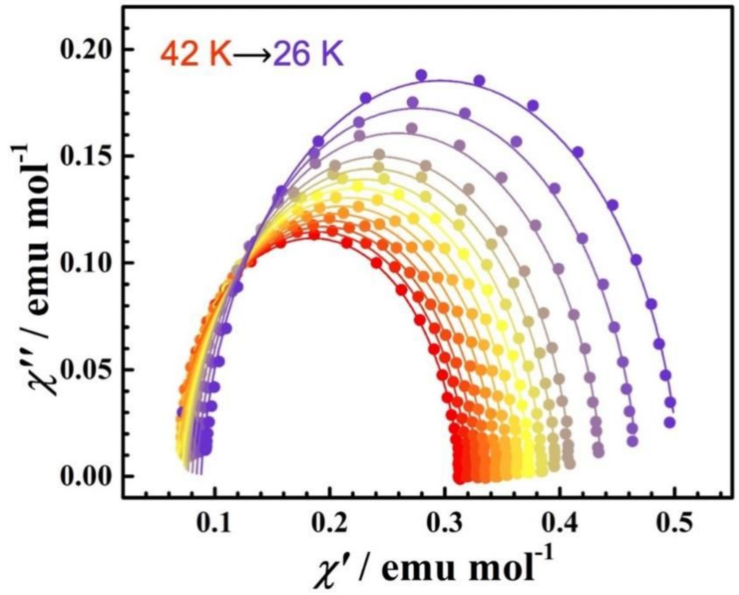

Figure S102 Cole-Cole plots for **1-Dy** from 42-26 K under zero applied dc field. The colored lines are fits to generalized Debye model.

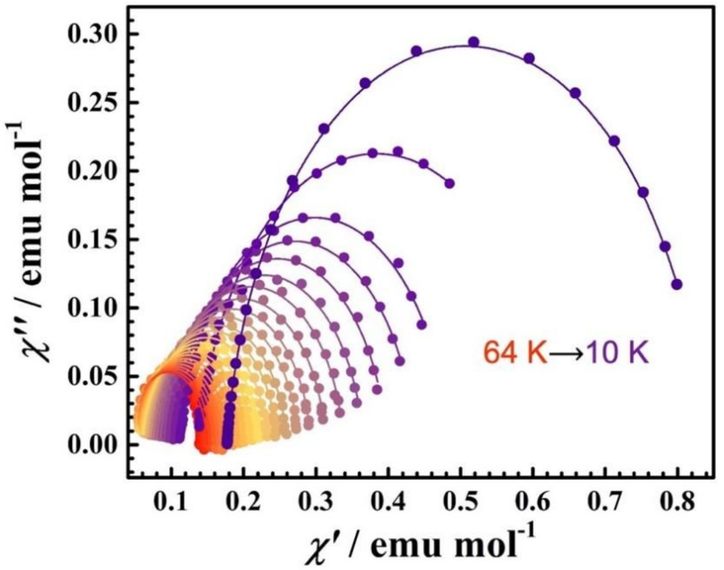

Figure S103 Cole-Cole plots for **2-Tb** from 64-10 K under 1500 Oe applied dc field. The colored lines are fits to generalized Debye model.

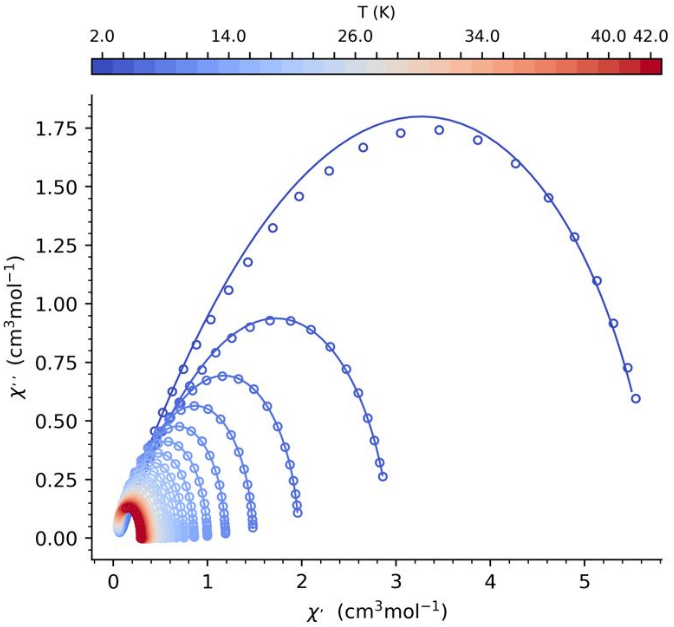

Figure S104 Cole-Cole plots for **2-Dy** from 42-2 K under zero applied dc field. The colored lines are fits to Havriliak-Negami model in CCFIT2[1,6].

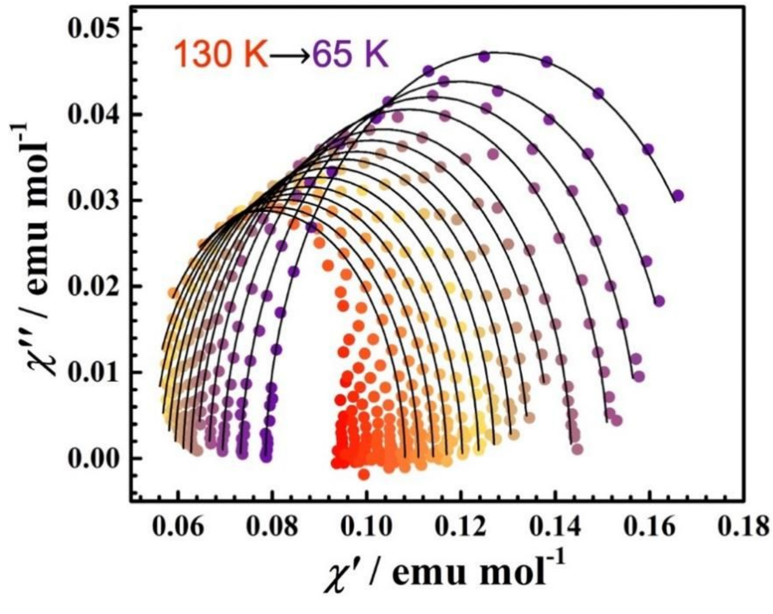

Figure S105 Cole-Cole plots for **3-Tb** from 130-65 K under zero applied dc field. The black lines are fits to generalized Debye model.

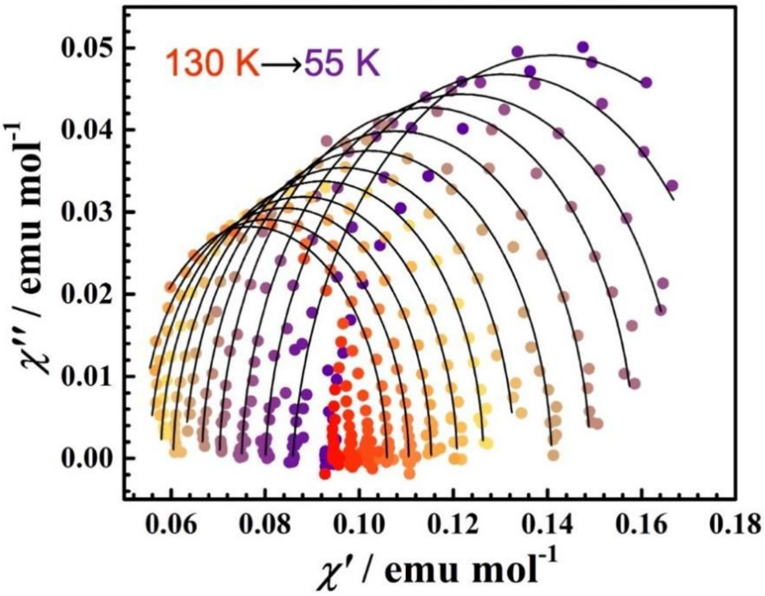

Figure S106 Cole-Cole plots for **3-Tb** from 130-55 K under 1500 Oe applied dc field. The black lines are fits to generalized Debye model.

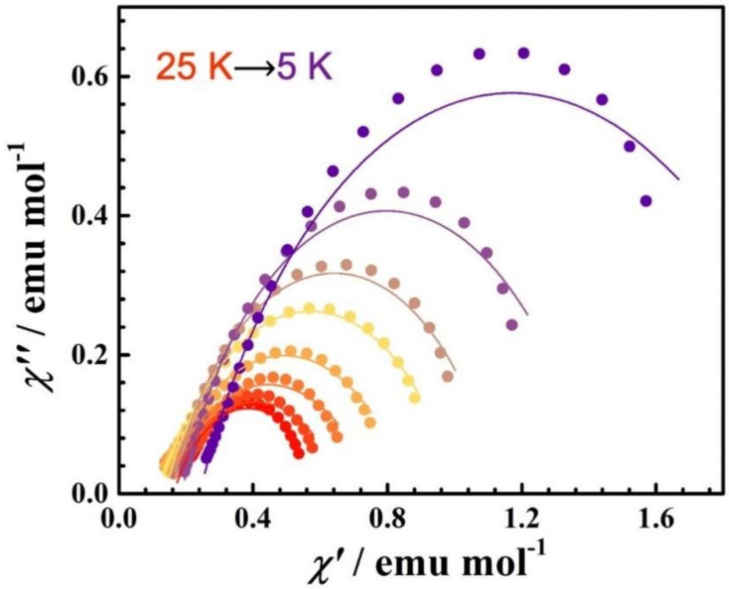

Figure S107 Cole-Cole plots for **3-Dy** from 25-5 K under zero applied dc field. The colored lines are fits to generalized Debye model.

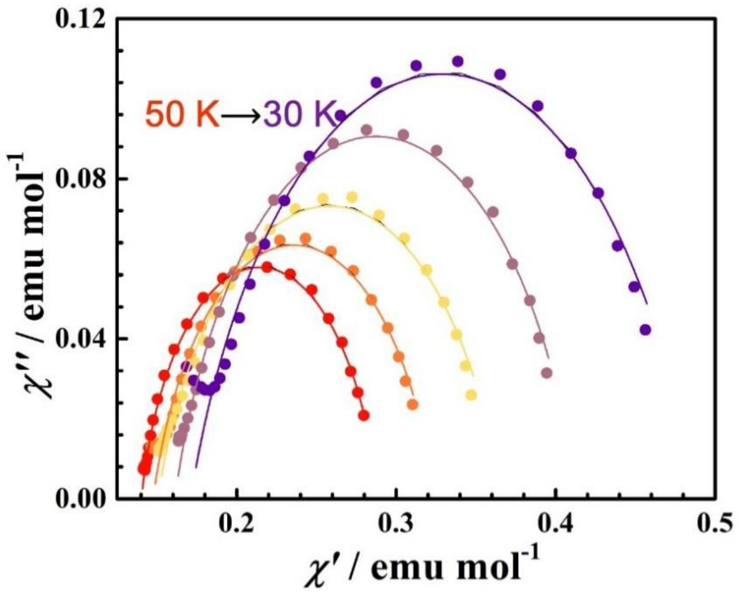

Figure S108 Cole-Cole plots for **3-Dy** from 50-30 K under zero applied dc field. The colored lines are fits to generalized Debye model.

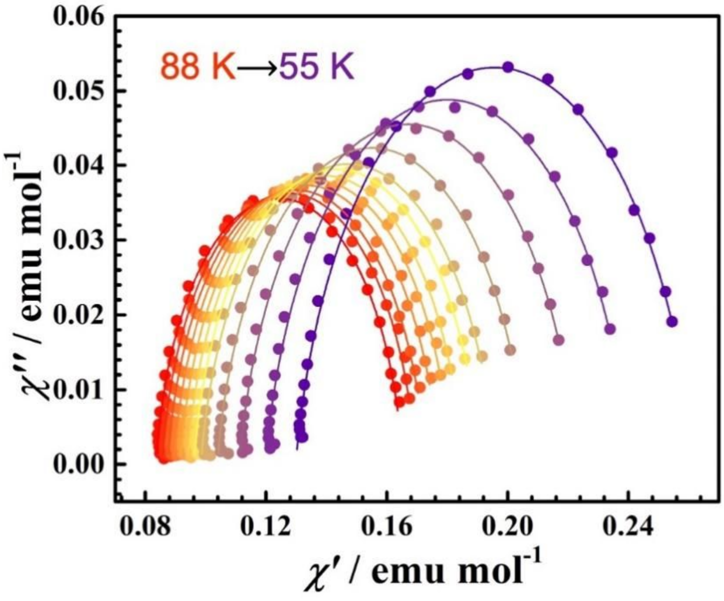

Figure S19 Cole-Cole plots for **3-Dy** from 88-55 K under zero applied dc field. The colored lines are fits to generalized Debye model.

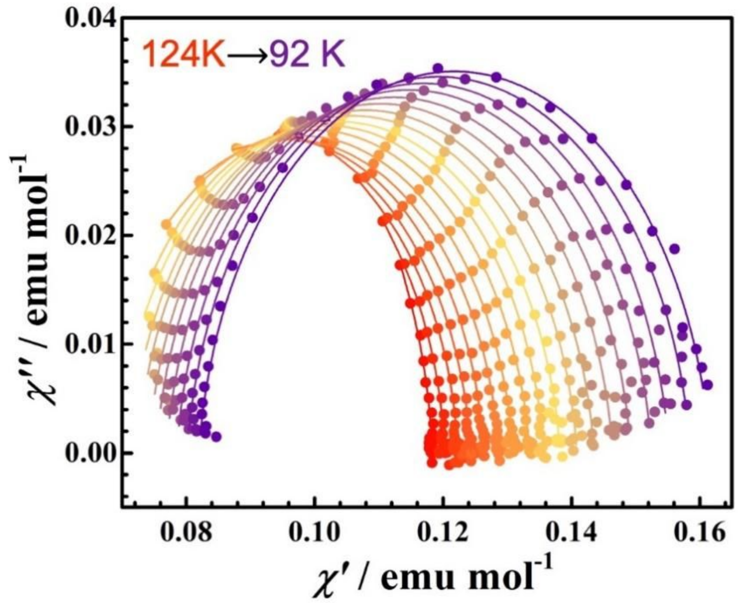

Figure S110 Cole-Cole plots for **3-Dy** from 124-92 K under zero applied dc field. The colored lines are fits to generalized Debye model.

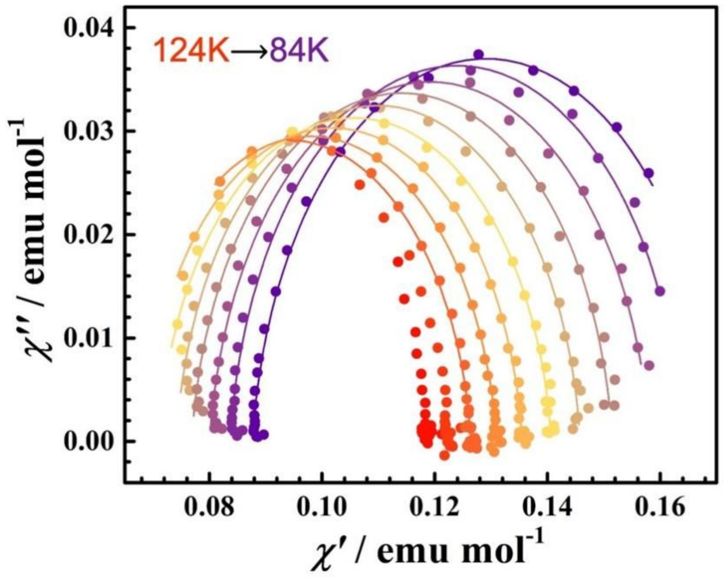

Figure S111 Cole-Cole plots for **3-Dy** from 124-84 K under 1000 Oe applied dc field. The colored lines are fits to generalized Debye model.

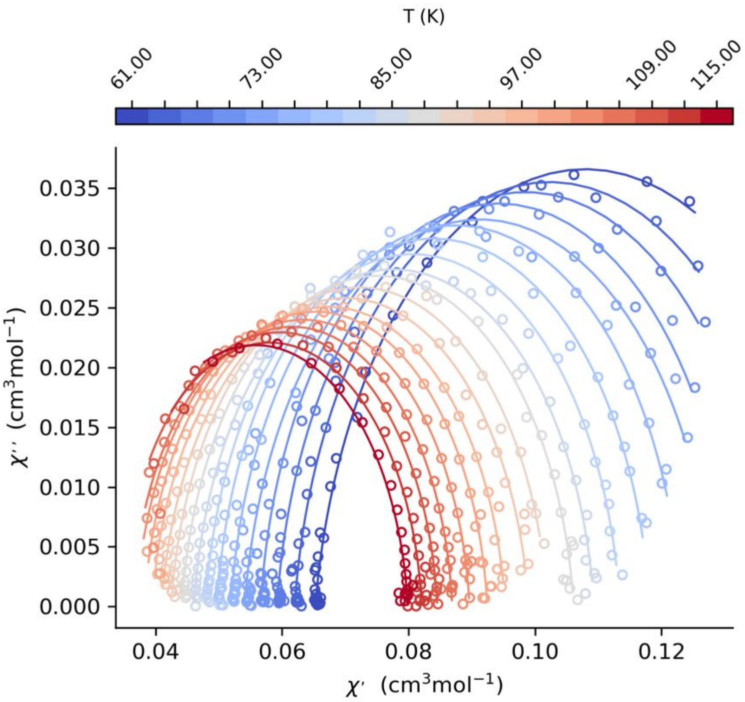

Figure S112 Cole-Cole plots for a 50 mM solution of **3-Tb** in hexane from 115-61 K under 0 Oe applied dc field. The colored lines are fits to generalized Debye model in CCFIT2[1,6].

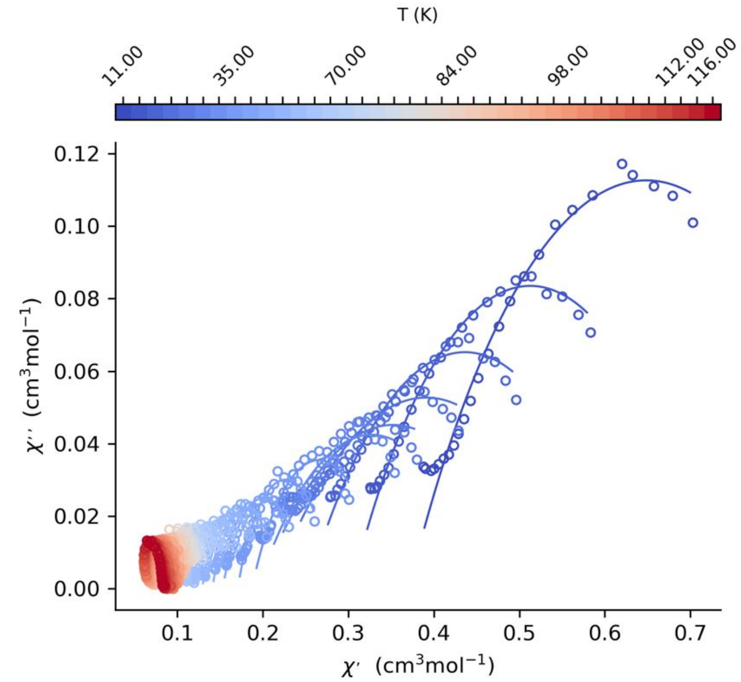

Figure S113 Cole-Cole plots for a 50 mM solution of **3-Tb** in hexane from 116-10 K under 0 Oe applied dc field. The colored lines are fits to generalized Debye model in CCFIT2[1,6].

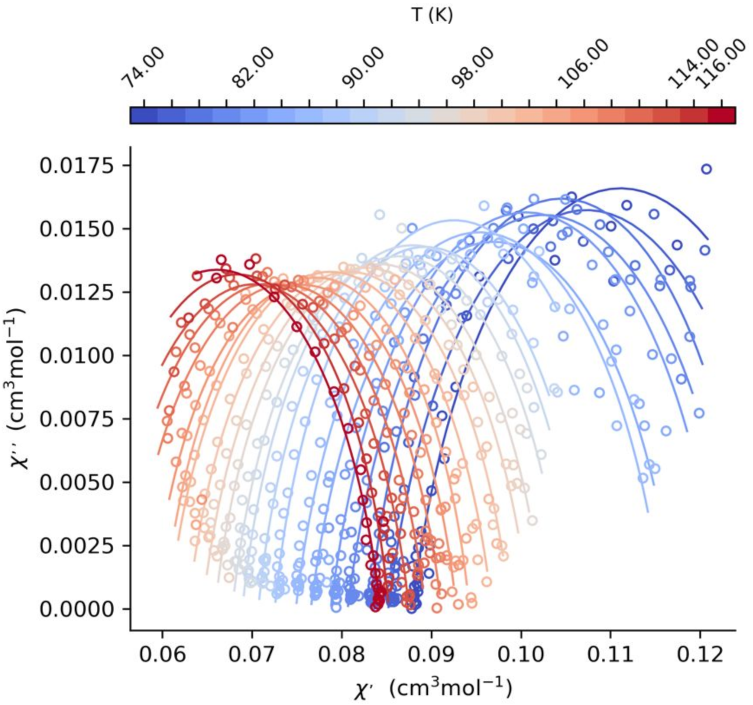

Figure S114 Cole-Cole plots for a 50 mM solution of **3-Dy** in hexane from 116-74 K under 1000 Oe applied dc field. The colored lines are fits to generalized Debye model in CCFIT2[1,6].

Table S6 Parameters used to fit ac magnetic relaxation data for **1-Tb** at 1500 Oe applied field and magnetic relaxation times extracted from these fits.

T	χ_S	χ_S^{err}	χ_T	χ_T^{err}	τ_{debye}	τ_{debye}^{err}	α	α^{err}
(K)	(cm^3 mol^{-1})		(cm^3 mol^{-1})		(s)			
6	0.06363	0.00305	1.64046	0.00991	0.30997	0.00356	0.10775	0.00575
10	0.04407	0.00197	0.97878	0.00276	0.05501	4.1844E-4	0.07255	0.00433
14	0.03399	0.00148	0.70361	0.00157	0.01904	1.3743E-4	0.06584	0.0041
16	0.0307	0.00129	0.61501	0.0012	0.01281	8.5967E-5	0.06298	0.00383
18	0.02846	0.00123	0.54826	0.00104	0.00912	6.2264E-5	0.06168	0.0039
20	0.02701	0.00102	0.49532	7.7661E-4	0.00684	4.0580E-5	0.06147	0.00339
22	0.02579	9.6306E-4	0.45016	6.7551E-4	0.00528	3.1013E-5	0.05662	0.00339
24	0.02418	8.9493E-4	0.41331	5.7752E-4	0.00417	2.3738E-5	0.05892	0.00328
26	0.02341	7.46113E-4	0.38216	4.4292E-4	0.0033	1.6179E-5	0.05699	0.00283
28	0.02257	7.39538E-4	0.35624	3.9720E-4	0.00255	1.2630E-5	0.0588	0.00285
30	0.02156	6.95323E-4	0.33237	3.5800E-4	0.00186	8.6406E-6	0.0597	0.00271
32	0.02042	5.62258E-4	0.31205	2.4761E-4	0.00126	4.6229E-6	0.06192	0.00211
34	0.01931	7.47753E-4	0.29455	2.6248E-4	8.0576E-4	3.7424E-6	0.06405	0.0026
36	0.01916	8.96157E-4	0.27784	2.1258E-4	4.9553E-4	2.6465E-6	0.05998	0.00274
38	0.01914	0.00102	0.26396	1.7478E-4	3.0360E-4	1.8261E-6	0.05962	0.00266
40	0.02081	0.00205	0.25094	1.9953E-4	1.8880E-4	2.3610E-6	0.05431	0.00429

Table S7 Parameters used to fit ac magnetic relaxation data for **1-Dy** at zero applied field and magnetic relaxation times extracted from these fits.

T	χ_S	χ_S^{err}	χ_T	χ_T^{err}	τ_{debye}	τ_{debye}^{err}	α	α^{err}
(K)	(cm^3 mol^{-1})		(cm^3 mol^{-1})		(s)			
16	0.10365	0.00647	0.92447	0.06233	0.98808	0.1419	0.20102	0.0382
18	0.10293	0.00516	0.75803	0.02586	0.51266	0.03562	0.13431	0.02892
20	0.0997	0.0041	0.66666	0.01414	0.31523	0.01428	0.10443	0.02258
22	0.09587	0.00322	0.59973	0.00855	0.20654	0.00682	0.08613	0.01781

24	0.09195	0.00247	0.54818	0.00541	0.14095	0.00352	0.07668	0.0139
26	0.08798	0.00181	0.5042	0.00337	0.09769	0.00179	0.06646	0.01042
28	0.08411	0.00127	0.46792	0.00206	0.06832	8.8893E-4	0.0613	0.00747
30	0.0803	8.3591E-4	0.43671	0.00118	0.04628	3.9946E-4	0.05856	0.00497
32	0.07647	5.4687E-4	0.40994	6.5366E-4	0.02795	1.5712E-4	0.06133	0.00321
33	0.07455	4.5384E-4	0.3975	4.8917E-4	0.02017	9.3030E-5	0.06572	0.00262
34	0.07262	3.7914E-4	0.38568	3.6192E-4	0.01374	5.1613E-5	0.06831	0.00212
35	0.07088	3.7488E-4	0.37458	3.1048E-4	0.00882	3.1326E-5	0.06787	0.00201
36	0.06873	3.2717E-4	0.36438	2.3088E-4	0.00548	1.6099E-5	0.07261	0.00165
37	0.0667	3.6669E-4	0.35485	2.1578E-4	0.00333	1.0206E-5	0.07605	0.00172
38	0.06472	3.4877E-4	0.34565	1.7711E-4	0.002	5.3539E-6	0.07812	0.00151
39	0.06241	4.5348E-4	0.33648	1.7445E-4	0.00119	3.7911E-6	0.07914	0.00174
40	0.06124	4.0429E-4	0.32849	1.1700E-4	7.2917E-4	1.8891E-6	0.07809	0.00136
41	0.05926	6.8836E-4	0.32083	1.4734E-4	4.484E-4	1.8479E-6	0.07804	0.00197
42	0.05678	0.00102	0.31342	1.5310E-4	2.8025E-4	1.6797E-6	0.08255	0.00239
6*	0.0595	1.2314E-4	0.70475	0.03235	0.44412	0.04623	0.27395	0.01256
8*	0.04847	2.3435E-4	0.34909	3.7893E-4	0.0649	3.4566E-4	0.20333	0.0256
10*	0.04013	4.1231E-4	0.25122	2.3489E-4	0.0145	2.7321E-5	0.1784	0.02634
12*	0.03548	4.5647E-4	0.20108	3.1245E-4	0.00463	1.2459E-5	0.14355	0.01120
14*	0.03118	6.3453E-4	0.17149	1.3356E-4	0.00195	3.2489E-6	0.13146	0.02306
16*	0.02549	3.3453E-4	0.15132	2.2343E-4	9.6713E-4	1.2475E-6	0.14835	0.01398
18*	0.02653	3.2344E-4	0.12845	2.3687E-4	5.4643E-4	1.2378E-6	0.15936	0.01458
20*	1.1553E-9	7.454E-11	0.11816	2.8536E-4	2.3659E-4	1.5662E-6	0.21602	0.02513

* The fitting of fast magnetic relaxation process.

Table S8 Parameters used to fit ac magnetic relaxation data for **2-Tb** at 1500 Oe applied field and magnetic relaxation times extracted from these fits.

T	χ_S	χ_S^{err}	χ_T	χ_T^{err}	τ_{debye}	τ_{debye}^{err}	α	α^{err}
(K)	(cm^3 mol^{-1})		(cm^3 mol^{-1})		(s)			
10	0.17905	0.00126	0.84208	0.00298	0.2739	0.00218	0.06787	0.00457
14	0.1379	8.4102E-4	0.62904	0.00404	0.09422	0.00115	0.09111	0.00509
18	0.11272	6.5275E-4	0.48337	0.00202	0.0409	3.61028E-4	0.07001	0.00479

20	0.10355	5.2106E-4	0.43677	0.00132	0.03038	2.08257E-4	0.07198	0.00387
22	0.09629	6.7105E-4	0.39728	0.00141	0.02283	1.97807E-4	0.06462	0.00508
24	0.08958	5.1587E-4	0.36551	9.48235E-4	0.01779	1.20522E-4	0.06804	0.00399
26	0.0843	5.5994E-4	0.33793	9.10339E-4	0.01421	1.05372E-4	0.05933	0.00446
28	0.07949	5.7874E-4	0.31377	8.5165E-4	0.01161	9.17591E-5	0.05982	0.00476
30	0.07513	5.2443E-4	0.29309	7.09192E-4	0.00974	7.18258E-5	0.05902	0.00445
32	0.07126	4.8484E-4	0.27602	6.04744E-4	0.00819	5.71099E-5	0.05853	0.00422
34	0.06801	5.8438E-4	0.25965	6.74649E-4	0.00692	5.97803E-5	0.05807	0.00523
36	0.06524	5.4055E-4	0.2461	5.80203E-4	0.0059	4.78491E-5	0.05263	0.00495
38	0.06202	5.3069E-4	0.23372	5.27746E-4	0.00497	4.04065E-5	0.05761	0.00492
40	0.05985	4.8876E-4	0.22203	4.47166E-4	0.00413	3.11447E-5	0.05211	0.00461
42	0.05719	5.3157E-4	0.21179	4.43722E-4	0.00337	2.76689E-5	0.05197	0.00502
44	0.05484	6.1171E-4	0.20235	4.59155E-4	0.00267	2.50516E-5	0.05133	0.00573
46	0.05331	4.7793E-4	0.19379	3.18288E-4	0.00206	1.47504E-5	0.04319	0.00442
48	0.17905	6.4980E-4	0.18571	3.91519E-4	0.00153	1.446E-5	0.04146	0.00586
50	0.1379	4.7043E-4	0.17854	2.51215E-4	0.00113	7.35278E-6	0.03411	0.0041
52	0.11272	4.7228E-4	0.17169	2.05353E-4	8.0358E-4	4.99476E-6	0.03036	0.00384
54	0.10355	6.6625E-4	0.16579	2.1817E-4	5.6421E-4	4.69045E-6	0.0276	0.0049
56	0.09629	9.3438E-4	0.16027	2.25107E-4	3.8959E-4	4.36458E-6	0.028	0.006
58	0.08958	8.8061E-4	0.15407	1.67834E-4	2.7132E-4	2.8201E-6	0.00801	0.00513
60	0.0843	0.00169	0.14899	1.94066E-4	1.8116E-4	3.63465E-6	0.00829	0.00771

Table S9 Parameters used to fit ac magnetic relaxation data for **2-Dy** at zero applied field and magnetic relaxation times extracted from these fits.

T	H	χ_S	χ_S^{err}	χ_T	χ_T^{err}	τ	τ^{err}	α	α^{err}	γ	γ^{err}
(K)	(Oe)	(cm^3 mol^{-1})		(cm^3 mol^{-1})		(s)					
2	0.01198	0.00724	0.0169	5.77867	0.03212	0.22329	0.0094	0.17387	0.01381	0.63579	0.022
4	0.01198	0.03717	0.00367	2.94416	0.00585	0.21849	0.00327	0.12002	0.00561	0.57796	0.0076
6	0.01198	0.05523	9.64229E-4	1.96991	0.00121	0.14591	7.41482E-4	0.04319	0.00201	0.5835	0.0027
8	0.01198	0.06163	4.72608E-4	1.48121	4.85234E-4	0.07262	2.34637E-4	0.01993	0.00116	0.65629	0.00189
10	0.01198	0.06039	6.27921E-4	1.18888	5.31018E-4	0.03502	1.81953E-4	0.0147	0.00169	0.73069	0.00333
12	0.01198	0.05669	6.30059E-4	0.99509	4.45681E-4	0.01825	1.08855E-4	0.01478	0.00178	0.7916	0.00411
14	0.01198	0.05246	6.67446E-4	0.85508	4.00553E-4	0.01045	7.27248E-5	0.01527	0.00194	0.83894	0.00508
16	0.01198	0.04845	5.92526E-4	0.74883	3.04205E-4	0.00651	4.30052E-5	0.01429	0.00175	0.87314	0.00505
18	0.01198	0.0451	6.15626E-4	0.66686	2.73486E-4	0.00434	3.1469E-5	0.01557	0.00183	0.90426	0.00577
20	0.01198	0.04216	5.64761E-4	0.60169	2.17643E-4	0.00306	2.11072E-5	0.01478	0.00167	0.92601	0.00568
22	0.01198	0.04011	5.82596E-4	0.54784	1.95669E-4	0.00223	1.63933E-5	0.01496	0.0017	0.94926	0.00626
24	0.01198	0.03747	5.55218E-4	0.5032	1.60567E-4	0.00169	1.19648E-5	0.01483	0.00157	0.96183	0.0062
26	0.01198	0.03572	6.81292E-4	0.4655	1.68849E-4	0.00131	1.13709E-5	0.01412	0.00184	0.97326	0.00785
28	0.01198	0.03435	7.1755E-4	0.43311	1.50377E-4	0.00102	9.30353E-6	0.01354	0.00183	0.98245	0.00845
30	0.01198	0.03358	8.7662E-4	0.40489	1.56908E-4	8.07997E-4	8.81715E-6	0.01113	0.00209	0.98756	0.01044
31	0.01198	0.03289	0.00482	0.39201	7.5917E-4	7.17518E-4	4.23948E-5	0.01075	0.01094	0.99099	0.05747

32	0.01198	0.03199	0.00122	0.38016	1.71901E-4	6.414E-4	9.36747E-6	0.00921	0.00262	0.98845	0.01441
33	0.01198	0.03106	0.00136	0.36901	1.68459E-4	5.75582E-4	9.05034E-6	0.00783	0.00273	0.98143	0.0157
34	0.01198	0.03217	0.00136	0.35866	1.51165E-4	5.02592E-4	7.91774E-6	0.01006	0.00259	1.00205	0.01625
35	0.01198	0.03119	0.00145	0.34862	1.46303E-4	4.44309E-4	7.23066E-6	0.01024	0.00255	1.0061	0.01719
36	0.01198	0.03033	0.00229	0.33918	1.87473E-4	4.00257E-4	9.78231E-6	0.00782	0.00365	0.99153	0.02614
37	0.01198	0.02903	0.00266	0.32986	1.87426E-4	3.60835E-4	9.67143E-6	0.0036	0.00385	0.97449	0.02909
38	0.01198	0.02989	0.00236	0.32187	1.47183E-4	3.12169E-4	7.32957E-6	0.00777	0.0031	0.99808	0.02657
39	0.01198	0.02527	0.00333	0.31342	1.68084E-4	2.90335E-4	8.62971E-6	0.00136	0.0038	0.94087	0.03327
40	0.01198	0.02727	0.00403	0.30605	1.59832E-4	2.45659E-4	8.81409E-6	0.00676	0.00402	0.98428	0.04275
41	0.01198	0.02534	0.00478	0.29861	1.48452E-4	2.20071E-4	8.69967E-6	0.0042	0.00409	0.96512	0.04812
42	0.01198	0.0237	0.00639	0.29206	1.47985E-4	1.92286E-4	9.63746E-6	0.00877	0.00454	0.96599	0.06306

Table S10 Parameters used to fit ac magnetic relaxation data for **3-Tb** at zero applied field and magnetic relaxation times extracted from these fits.

T	χ_S	χ_S^{err}	χ_T	χ_T^{err}	τ_{debye}	τ_{debye}^{err}	α	α^{err}
(K)	(cm^3 mol^{-1})		(cm^3 mol^{-1})		(s)			
65	0.07856	1.0563E-4	1.0563E-4	3.8369E-4	0.53739	0.00301	0.02728	0.00316
70	0.07325	1.1528E-4	1.1528E-4	2.8658E-4	0.31174	0.00158	0.03599	0.00306
75	0.06944	1.3817E-4	1.3817E-4	2.3447E-4	0.159	8.1997E-4	0.03295	0.00322
80	0.06654	1.9299E-4	1.9299E-4	2.2771E-4	0.07348	4.6081E-4	0.02781	0.00396
85	0.06418	1.5973E-4	1.5973E-4	1.2945E-4	0.03104	1.4182E-4	0.02232	0.0029
88	0.06274	1.1840E-4	1.1840E-4	2.1257E-4	0.01811	9.3787E-5	0.01885	0.0033
91	0.06108	1.2980E-4	1.2979E-4	1.7802E-4	0.01037	5.3671E-5	0.01701	0.00331
94	0.05934	1.3649E-4	1.3649E-4	1.4750E-4	0.00603	3.0021E-5	0.01625	0.00319
97	0.05791	1.6137E-4	1.6137E-4	1.3885E-4	0.00355	1.9180E-5	0.0155	0.00347
100	0.05626	1.684E-4	1.684E-4	1.1466E-4	0.00211	1.0877E-5	0.02069	0.00328
103	0.0547	2.0828E-4	2.0828E-4	1.1345E-4	0.00127	7.3654E-6	0.02439	0.00367
106	0.05357	2.8582E-4	2.8582E-4	1.1795E-4	7.7610E-4	5.5593E-6	0.01922	0.00448
109	0.05184	2.9368E-4	2.9368E-4	8.4799E-5	4.7716E-4	3.2128E-6	0.02238	0.00387
112	0.05043	7.5034E-4	7.5034E-4	1.5049E-4	2.9900E-4	4.9603E-6	0.02383	0.0082
115	0.04828	9.3830E-4	9.3830E-4	1.0865E-4	1.8862E-4	3.9102E-6	0.02524	0.00783

Table S11 Parameters used to fit ac magnetic relaxation data for **3-Tb** at 1500 Oe applied field and magnetic relaxation times extracted from these fits.

T	χ_S	χ_S^{err}	χ_T	χ_T^{err}	τ_{debye}	τ_{debye}^{err}	α	α^{err}
(K)	(cm^3 mol^{-1})		(cm^3 mol^{-1})		(s)			
60	0.08589	2.64E-4	0.19592	0.00169	1.02943	0.02309	0.07183	0.0089
65	0.08008	2.37322E-4	0.18104	8.8978E-4	0.56613	0.00737	0.04804	0.00693
70	0.075	2.11062E-4	0.16883	5.1694E-4	0.30467	0.00277	0.03557	0.00551
75	0.07031	2.18589E-4	0.15863	3.6130E-4	0.15305	0.00121	0.02079	0.00505

80	0.06654	3.36088E-4	0.14907	3.1601E-4	0.06954	7.4568E-4	0.02224	0.00667
85	0.063	3.46508E-4	0.1409	2.7588E-4	0.02986	2.9929E-4	0.0248	0.00635
90	0.06045	1.96069E-4	0.13297	2.8617E-4	0.01189	9.6521E-5	0.01568	0.00521
95	0.05783	1.95125E-4	0.12637	1.9238E-4	0.00487	3.4164E-5	0.00968	0.00454
100	0.05538	2.5612E-4	0.12078	1.9036E-4	0.00205	1.6435E-5	0.01644	0.00523
105	0.05323	3.06663E-4	0.11528	1.3794E-4	8.8065E-4	7.0733E-6	0.01119	0.00513
110	0.05044	3.92829E-4	0.11052	1.1321E-4	3.9616E-4	3.5464E-6	0.02067	0.00508
115	0.04793	0.00113	0.10588	1.3210E-4	1.8451E-4	4.6736E-6	0.01665	0.00967

Table S12 Parameters used to fit ac magnetic relaxation data for **3-Dy** at zero applied field and magnetic relaxation times extracted from these fits.

T	χ_S	χ_S^{err}	χ_T	χ_T^{err}	τ_{debye}	τ_{debye}^{err}	α	α^{err}
(K)	(cm^3 mol^{-1})		(cm^3 mol^{-1})		(s)			
5	0.24082	0.01078	2.09748	0.01593	0.04649	0.00255	0.29059	0.01614
8	0.18675	0.00471	1.40515	0.0097	0.03232	8.4182E-4	0.24786	0.00965
11	0.15438	0.0026	1.12706	0.00542	0.02447	3.88324E-4	0.26159	0.00606
14	0.13604	0.00168	1.0017	0.00183	0.02099	2.11306E-4	0.3035	0.00366
17	0.12183	0.00341	0.8633	0.00195	0.01796	5.00471E-4	0.37148	0.00857
21	0.12278	0.006	0.7658	0.00211	0.01786	0.00108	0.41992	0.01626
23	0.14116	0.00693	0.67242	9.45505E-4	0.01886	0.00162	0.41037	0.02345
25	0.16906	0.00487	0.59582	5.2465E-4	0.0193	0.00111	0.32409	0.0208
30	0.17091	0.00256	0.48681	5.04344E-4	0.01858	7.54456E-4	0.24389	0.01697
35	0.16119	9.39228E-4	0.41295	4.64295E-4	0.01681	2.81186E-4	0.2026	0.00773
40	0.1503	9.96107E-4	0.36592	4.81488E-4	0.01649	3.59051E-4	0.23784	0.00937
45	0.14754	0.00103	0.32376	4.72245E-4	0.01817	4.92778E-4	0.20229	0.01244
50	0.14034	4.67046E-4	0.28823	4.1206E-4	0.01911	2.66429E-4	0.1481	0.00705
55	0.12966	2.60455E-4	0.2613	3.55861E-4	0.01937	1.59803E-4	0.13068	0.00433
60	0.11971	2.47393E-4	0.24018	3.74734E-4	0.01984	1.70439E-4	0.12805	0.00453
65	0.11105	2.26619E-4	0.22276	3.86737E-4	0.02009	1.70207E-4	0.12459	0.00449
70	0.10379	2.39396E-4	0.20614	3.39597E-4	0.01971	1.87741E-4	0.11466	0.00515
74	0.0984	2.35752E-4	0.19567	3.76246E-4	0.01958	1.98623E-4	0.11281	0.00547

76	0.09596	2.09339E-4	0.19067	3.18123E-4	0.0191	1.68619E-4	0.10983	0.00482
78	0.09377	1.87181E-4	0.1849	3.60716E-4	0.01819	1.4421E-4	0.09955	0.00442
80	0.09146	2.00534E-4	0.18114	2.52279E-4	0.01764	1.50752E-4	0.09969	0.00478
82	0.08935	2.14368E-4	0.17696	2.37268E-4	0.01662	1.51627E-4	0.09594	0.00515
84	0.08735	1.97746E-4	0.17257	2.10653E-4	0.01527	1.27587E-4	0.08959	0.00478
86	0.08548	2.32532E-4	0.16927	1.59667E-4	0.01368	1.31847E-4	0.08588	0.00556
88	0.08358	2.12331E-4	0.16497	2.27014E-4	0.01182	1.0218E-4	0.08005	0.00505
90	0.08187	2.62562E-4	0.16151	1.52272E-4	0.00995	1.03333E-4	0.07456	0.00613
92	0.08004	2.03755E-4	0.15842	1.53087E-4	0.008	6.2113E-5	0.07336	0.0046
94	0.07843	2.15395E-4	0.15508	1.19717E-4	0.00622	4.91118E-5	0.07043	0.0047
96	0.07683	2.16624E-4	0.15238	8.62016E-5	0.00473	3.59446E-5	0.0727	0.0045
98	0.07524	1.8937E-4	0.14913	9.44701E-5	0.00347	2.18905E-5	0.07156	0.00374
100	0.07398	3.1394E-4	0.14629	1.10625E-4	0.0025	2.4663E-5	0.06942	0.00586
102	0.07234	2.49975E-4	0.14336	1.07312E-4	0.00177	1.29928E-5	0.06979	0.00434
104	0.07125	3.00526E-4	0.14068	8.23822E-5	0.00126	1.03967E-5	0.06597	0.00486
106	0.06944	2.79194E-4	0.13805	0.01593	8.82759E-4	6.29003E-6	0.06946	0.0041
108	0.06826	2.43491E-4	0.13548	0.0097	6.25387E-4	3.63895E-6	0.06438	0.00326
110	0.06613	3.59218E-4	0.13335	0.00542	4.39196E-4	3.59482E-6	0.07325	0.00411
112	0.06506	5.93403E-4	0.13084	0.00183	3.12809E-4	4.15799E-6	0.07085	0.00587
114	0.06366	8.0486E-4	0.12861	0.00195	2.22884E-4	3.998E-6	0.07041	0.0067
116	0.0619	8.9808E-4	0.12634	0.00211	1.60958E-4	3.18671E-6	0.06282	0.00616

Table S13 Parameters used to fit ac magnetic relaxation data for **3-Dy** at 1000 Oe applied field and magnetic relaxation times extracted from these fits.

T	χ_S	χ_S^{err}	χ_T	χ_T^{err}	τ_{debye}	τ_{debye}^{err}	α	α^{err}
(K)	(cm^3 mol^{-1})		(cm^3 mol^{-1})			(s)		
84	0.08763	1.82812E-4	0.17052	6.98612E-4	0.05368	7.01275E-4	0.07201	0.00663
88	0.08374	1.85895E-4	0.16408	4.32084E-4	0.02694	2.56095E-4	0.06378	0.00552
92	0.08003	2.3717E-4	0.15823	3.73276E-4	0.01311	1.34128E-4	0.07447	0.00601
96	0.07689	2.21843E-4	0.15152	2.41502E-4	0.00607	5.00649E-5	0.0649	0.00495

100	0.07414	2.65182E-4	0.14577	2.03553E-4	0.00282	2.42424E-5	0.06221	0.00516
104	0.07104	3.1047E-4	0.14065	1.63316E-4	0.00134	1.15857E-5	0.06799	0.00509
108	0.0683	3.2833E-4	0.13549	1.14302E-4	6.47731E-4	5.14085E-6	0.06552	0.00442
112	0.06494	4.94379E-4	0.13095	1.04264E-4	3.15634E-4	3.46965E-6	0.07164	0.005
116	0.06172	0.00133	0.12639	1.3664E-4	1.60795E-4	4.71901E-6	0.06655	0.00925

Table S14 Parameters used to fit ac magnetic relaxation data for a 50 mM solution of **3-Tb** in hexane at zero applied field and magnetic relaxation times extracted from these fits.

T	χ_S	χ_S^{err}	χ_T	χ_T^{err}	τ_{debye}	τ_{debye}^{err}	α	α^{err}
(K)	(cm^3 mol^{-1})		(cm^3 mol^{-1})		(s)			
61	0.06526	1.06063E-4	0.15068	8.27345E-4	0.91551	0.01381	0.09806	0.00561
64	0.062	1.07249E-4	0.1427	6.30248E-4	0.69613	0.00838	0.08105	0.00527
67	0.0592	1.32959E-4	0.13777	6.37846E-4	0.5487	0.00705	0.07931	0.0061
70	0.05662	1.63882E-4	0.13172	6.09562E-4	0.39533	0.00527	0.06835	0.00701
73	0.05406	2.13997E-4	0.12858	6.57357E-4	0.28048	0.0045	0.08888	0.00845
76	0.05192	1.66356E-4	0.12318	3.92352E-4	0.18822	0.00202	0.07034	0.00605
79	0.04971	1.73353E-4	0.11815	3.2656E-4	0.11673	0.00121	0.06702	0.00594
82	0.04793	1.89917E-4	0.11342	3.01E-4	0.06994	7.81203E-4	0.06663	0.00638
85	0.04606	1.78561E-4	0.10936	2.44388E-4	0.0417	4.35901E-4	0.07308	0.00588
88	0.04416	1.86052E-4	0.10612	2.04686E-4	0.02449	2.41108E-4	0.07154	0.00557
91	0.04248	1.59284E-4	0.10202	2.44088E-4	0.0138	1.19181E-4	0.069	0.00516
94	0.04097	2.35584E-4	0.09903	2.91829E-4	0.008	9.71546E-5	0.0746	0.00718
97	0.03948	2.06073E-4	0.09528	1.97198E-4	0.00457	4.40545E-5	0.06767	0.00576
100	0.03827	2.46414E-4	0.09246	1.85418E-4	0.0027	2.81148E-5	0.05974	0.00628
103	0.03696	3.13694E-4	0.08966	1.84013E-4	0.00163	1.95351E-5	0.05941	0.00721
106	0.0363	3.64315E-4	0.08691	1.79482E-4	0.001	1.27163E-5	0.04944	0.00772
109	0.03459	3.64184E-4	0.08454	1.28794E-4	6.14829E-4	7.06233E-6	0.05378	0.00656
112	0.03412	7.51029E-4	0.08208	1.75899E-4	3.88822E-4	8.86111E-6	0.05247	0.01151
115	0.03181	0.00101	0.07989	1.49279E-4	2.40876E-4	7.22934E-6	0.06002	0.01203

Table S15 Parameters used to fit ac magnetic relaxation data for a 50 mM solution of **3-Dy** in hexane at zero applied field and magnetic relaxation times extracted from these fits.

T	χ_S	χ_S^{err}	χ_T	χ_T^{err}	τ_{debye}	τ_{debye}^{err}	α	α^{err}
(K)	(cm^3 mol^{-1})		(cm^3 mol^{-1})		(s)			
11	0.00359	0.00359	0.92604	0.02248	0.08264	0.01134	0.50944	0.01609
13	0.00243	0.00243	0.7221	0.01047	0.04905	0.00417	0.51665	0.01184
17	0.00296	0.00296	0.62112	0.01131	0.04437	0.00507	0.56765	0.01378
20	0.00489	0.00489	0.57056	0.01971	0.05815	0.01395	0.63644	0.02019
23	0.00616	0.00616	0.50867	0.0272	0.06937	0.02702	0.64208	0.03007
25	0.00533	0.00533	0.46059	0.02475	0.06659	0.02558	0.60756	0.03435
30	0.00208	0.00208	0.34326	0.0075	0.03644	0.00532	0.45632	0.02785
35	8.58323E-4	8.58323E-4	0.28273	0.00263	0.02926	0.00183	0.3716	0.01679
40	6.40761E-4	6.40761E-4	0.24941	0.00194	0.0285	0.00156	0.38765	0.01417
45	5.50894E-4	5.50894E-4	0.21953	0.00168	0.02937	0.0016	0.34556	0.01583
50	3.83752E-4	3.83752E-4	0.19817	0.0012	0.0314	0.00131	0.3047	0.01332
55	2.92268E-4	2.92268E-4	0.1785	8.88922E-4	0.03071	0.00105	0.28453	0.01164
60	4.45673E-4	4.45673E-4	0.16336	0.00134	0.03043	0.0017	0.27526	0.01955
65	2.56341E-4	2.56341E-4	0.1535	8.47681E-4	0.0341	0.00126	0.29852	0.01174
70	2.54907E-4	2.54907E-4	0.14272	8.06044E-4	0.0325	0.00119	0.28239	0.01237
72	2.99909E-4	2.99909E-4	0.13673	8.87465E-4	0.03042	0.00128	0.25425	0.0156
74	2.71947E-4	2.71947E-4	0.13285	7.79177E-4	0.02883	0.00112	0.26161	0.01424
76	2.14553E-4	2.14553E-4	0.12959	6.11812E-4	0.02855	8.84198E-4	0.26449	0.01133
78	2.44886E-4	2.44886E-4	0.12717	6.79188E-4	0.02855	9.38696E-4	0.2265	0.01326
80	2.214E-4	2.214E-4	0.12375	6.01118E-4	0.02696	8.36288E-4	0.25048	0.01192
82	3.13672E-4	3.13672E-4	0.11929	7.53997E-4	0.02397	9.47842E-4	0.19183	0.01787
84	3.23068E-4	3.23068E-4	0.11816	8.19237E-4	0.02494	0.00108	0.22856	0.01786
86	3.33556E-4	3.33556E-4	0.11455	7.45067E-4	0.02074	8.68263E-4	0.21782	0.01825

88	2.7789E-4	2.7789E-4	0.11126	5.53604E-4	0.01728	5.69377E-4	0.21529	0.01479
90	2.95999E-4	2.95999E-4	0.1085	5.24948E-4	0.01477	4.75567E-4	0.17543	0.01587
92	3.11525E-4	3.11525E-4	0.10588	4.75452E-4	0.01204	3.80416E-4	0.18332	0.01568
94	4.11428E-4	4.11428E-4	0.10363	5.80056E-4	0.00968	3.95637E-4	0.18416	0.02028
95	4.00859E-4	4.00859E-4	0.10198	4.00423E-4	0.00766	2.66243E-4	0.20343	0.01692
97	3.07263E-4	3.07263E-4	0.09949	2.75321E-4	0.00547	1.34224E-4	0.17714	0.01247
99	3.96738E-4	3.96738E-4	0.0977	2.72963E-4	0.00407	1.18185E-4	0.1883	0.01395
101	4.31993E-4	4.31993E-4	0.0957	2.60022E-4	0.00299	9.46682E-5	0.17081	0.01513
103	4.78153E-4	4.78153E-4	0.09375	2.42438E-4	0.00207	7.06765E-5	0.18461	0.01573
105	5.27138E-4	5.27138E-4	0.09168	2.51456E-4	0.0014	5.31386E-5	0.18797	0.01744
107	7.53309E-4	7.53309E-4	0.08942	3.06147E-4	9.74842E-4	4.5088E-5	0.15592	0.02237
109	0.00103	0.00103	0.08846	3.89973E-4	6.85245E-4	4.03935E-5	0.18232	0.02661
111	0.00104	0.00104	0.0859	2.87299E-4	4.76857E-4	2.7103E-5	0.14079	0.02508
113	8.63463E-4	8.63463E-4	0.08503	1.64713E-4	3.36079E-4	1.54169E-5	0.1634	0.01654

Table S16 Parameters used to fit ac magnetic relaxation data for a 50 mM solution of **3-Dy** in hexane at 1000 Oe applied field and magnetic relaxation times extracted from these fits.

T	χ_S	χ_S^{err}	χ_T	χ_T^{err}	τ_{debye}	τ_{debye}^{err}	α	α^{err}
(K)	(cm^3 mol^{-1})		(cm^3 mol^{-1})		(s)			
74	0.0879	1.97804E-4	0.13436	0.00149	0.74905	0.04698	0.21048	0.01827
76	0.08521	2.22761E-4	0.12942	0.00122	0.50683	0.02837	0.2125	0.01863
78	0.08315	2.04642E-4	0.12592	8.35106E-4	0.35727	0.01382	0.17528	0.01574
80	0.08068	2.16609E-4	0.1244	7.89428E-4	0.27847	0.01097	0.21327	0.01517
82	0.0788	2.37256E-4	0.12239	7.19083E-4	0.20588	0.00772	0.20745	0.01538
84	0.07657	2.89111E-4	0.11728	6.99993E-4	0.13304	0.00568	0.21277	0.01808
86	0.0749	2.72389E-4	0.11586	5.58348E-4	0.09586	0.00342	0.20098	0.01582
88	0.07254	2.15867E-4	0.11252	9.71441E-4	0.05797	0.00255	0.16704	0.01703

90	0.07071	1.70643E-4	0.10794	5.27977E-4	0.03589	9.76243E-4	0.16675	0.0121
92	0.06893	2.67364E-4	0.10686	6.7758E-4	0.02653	9.68513E-4	0.17547	0.01685
94	0.06781	2.70867E-4	0.10485	5.45187E-4	0.01826	5.94749E-4	0.17994	0.01563
96	0.06587	2.50675E-4	0.10257	3.88684E-4	0.01149	3.0119E-4	0.18802	0.01282
98	0.06449	3.17891E-4	0.10093	4.15172E-4	0.00836	2.5491E-4	0.19018	0.01505
100	0.06289	2.37751E-4	0.09879	2.46226E-4	0.00538	1.10139E-4	0.18817	0.0102
102	0.06069	3.91131E-4	0.09679	3.19706E-4	0.00343	1.02583E-4	0.19155	0.01478
104	0.06019	3.27194E-4	0.09426	2.20206E-4	0.00235	5.54549E-5	0.16692	0.01203
106	0.05887	5.10415E-4	0.0928	2.9681E-4	0.00165	5.68132E-5	0.17847	0.01708
108	0.05623	4.1938E-4	0.09114	1.75242E-4	0.00107	2.74578E-5	0.19534	0.01154
110	0.05447	5.879E-4	0.08934	2.13391E-4	6.97008E-4	2.30438E-5	0.19259	0.01442
112	0.05293	7.54731E-4	0.08753	1.8525E-4	4.79672E-4	1.97504E-5	0.18899	0.01571
114	0.05144	9.81295E-4	0.08528	1.94073E-4	3.20397E-4	1.59755E-5	0.15679	0.01827
116	0.0479	0.00209	0.08433	2.17399E-4	1.97725E-4	2.07141E-5	0.19351	0.02646

6.3 Dc magnetization decays

Dc decay measurements were collected by magnetizing samples at 5 T and allowing 5 minutes for equilibration, then returning the field to 0 Oe or 1000 Oe (Linear, 700 Oe/s), and measuring the magnetization as a function of time. The DC decay data were fitted with stretched exponential function below[2,3] in OriginPro:[4]

$$M(t) = M_{eq} + (M_0 - M_{eq})e^{-(t/\tau^*)^{\beta}}$$

Equation S1

where $M(t)$ is the magnetization at time t, M_0 is the initial magnetization measured after the magnetic field has been removed, M_{eq} is the final value of the magnetization at t = ∞, τ^* is the magnetic relaxation time, and β is a free variable. Whilst M_{eq} should equal zero for measurements in zero field, we find non-zero values for **1-Dy** and **3-Tb** in the cases where we have collected the full decay under zero applied dc field, which likely arise due to a small remnant field in the superconducting magnet. At high temperatures, where the relaxation is relatively fast, it is simple to observe the full decay for **1-Dy**, **3-Tb** and **3-Dy**. However, when the relaxation times become longer in the lower temperature region, the full measurement of such decay curves take more time and thus we are only able to do this for a limited number of temperatures. We have collected dc magnetization decay curves to completion for **1-Dy** for temperatures 2-10 K. For **3-Dy** both crystalline sample and solution sample, experiments were conducted from 2 K to 65 K. While the relaxation rate is fast for **3-Dy** at zero field because of strong QTM, the QTM was largely suppressed and dc decay for **3-Dy** was measured under 1000 Oe applied dc field, which gives different M_{eq} as the temperature varies. In the high temperature region from 65 K to 15 K, the curves go flat gradually after 2500 s, thus better fits to the data were obtained by treating M_{eq} as a free variable. In the low temperature region, several hours was even not a sufficient duration to observe the full decay of the magnetization for measurements. Thus, the field-cooled susceptibility measurements were collected at 1000 Oe and these values were used for fixing the M_{eq} value from 2-10 K, which gives more accurate relaxation times than free fitting. For **3-Tb**, it is fast to observe the full decay at zero applied dc field, so the M_{eq} is treated as a free variable, while the relaxation time of **3-Tb** is slower and

especially long at 2-10 K under 1500 Oe applied dc field. So, M_{eq} values of 2-10 K were fixed using 1.5 times field-cooled susceptibility value collected at 1000 Oe, which gives a nice trend of M_{eq} value for temperatures 2-35 K and thus more accurate relaxation times.[5]

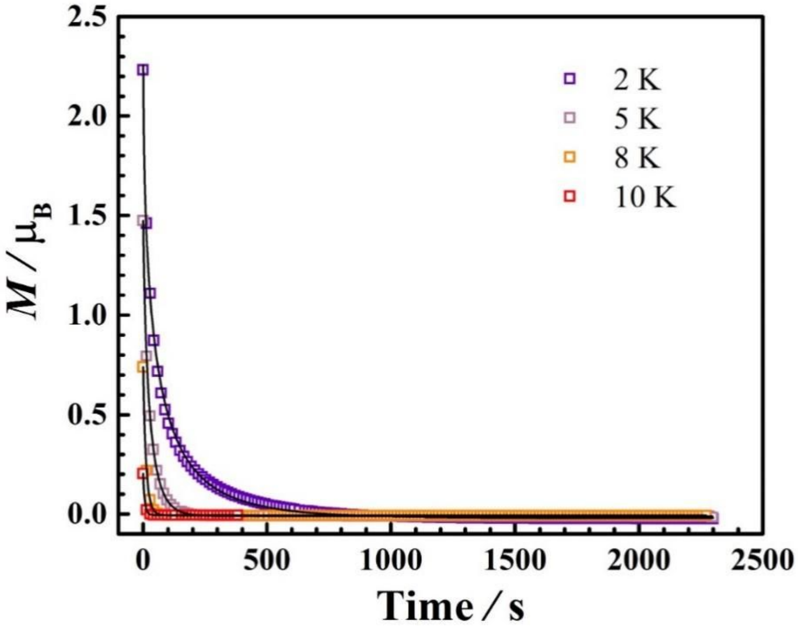

Figure S115 Dc magnetic relaxation data for **1-Dy** collected from 2-10 K under zero field. The black line represents the fit to the data using a stretched exponential function, which was used to extract τ^*.

Table S17 Parameters used to fit dc magnetic relaxation data for **1-Dy** at zero applied field and magnetic relaxation times extracted from these fits.

T	M_0	M_0^{err}	M_{eq}	M_{eq}^{err}	τ^*	$\tau^{*\,err}$	β	β^{err}
(K)	(μ_B)		(μ_B)			(s)		
2	2.2317	0	-0.02104	0.00116	51.96	0.45	0.57667	0.00437
5	1.4733	0	-0.01066	5.21E-5	25.41	0.02	0.79728	8.48E-4
8	0.7391	0	-0.0068	1.92E-6	11.45	0.001	0.90847	1.70E-4
10	0.2036	0	-0.00549	1.87E-6	6.33	0.005	0.90675	7.88E-4

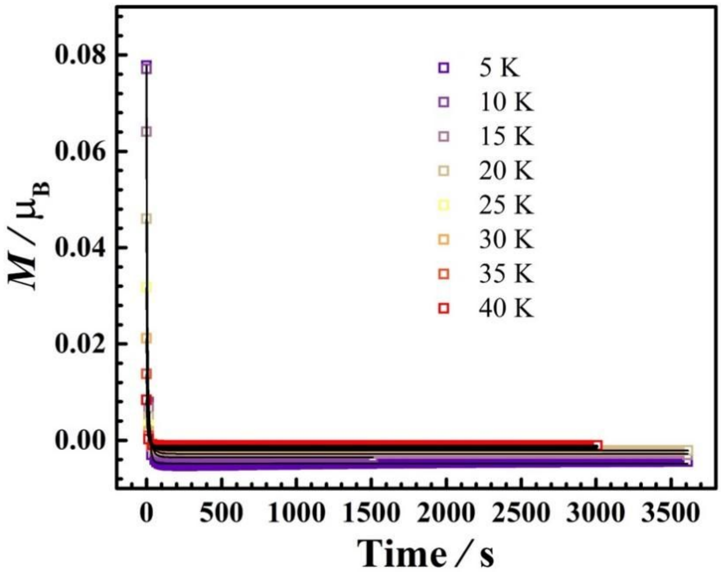

Figure S116 Dc magnetic relaxation data for **3-Tb** collected from 5-40 K under zero field. The black line represents the fit to the data using a stretched exponential function, which was used to extract τ^*.

Table S18 Parameters used to fit dc magnetic relaxation data for **3-Tb** at zero applied field and magnetic relaxation times extracted from these fits.

T	M_0	M_0^{err}	M_{eq}	M_{eq}^{err}	τ^*	$\tau^{*\,err}$	β	β^{err}
(K)	(μ B)		(μ B)			(s)		
5	0.07775	0	-0.00482	1.68E-5	5.5	0.3	0.7229	0.03875
10	0.07706	0	-0.00354	8.61E-6	4.38	0.09	0.5891	0.00842
15	0.06405	0	-0.00278	3.31E-6	4.38	0.06	0.5803	0.00584
20	0.04593	0	-0.00211	2.33E-6	4.56	0.07	0.5939	0.00678
25	0.03173	0	-0.00172	1.96E-5	4.43	0.07	0.5799	0.00611
30	0.02117	0	-0.00146	1.25E-6	4.53	0.06	0.5947	0.00611
35	0.01375	0	-0.00126	7.40E-6	4.58	0.06	0.6103	0.00596
40	0.00837	0	-0.00111	3.54E-7	4.93	0.06	0.6715	0.00731

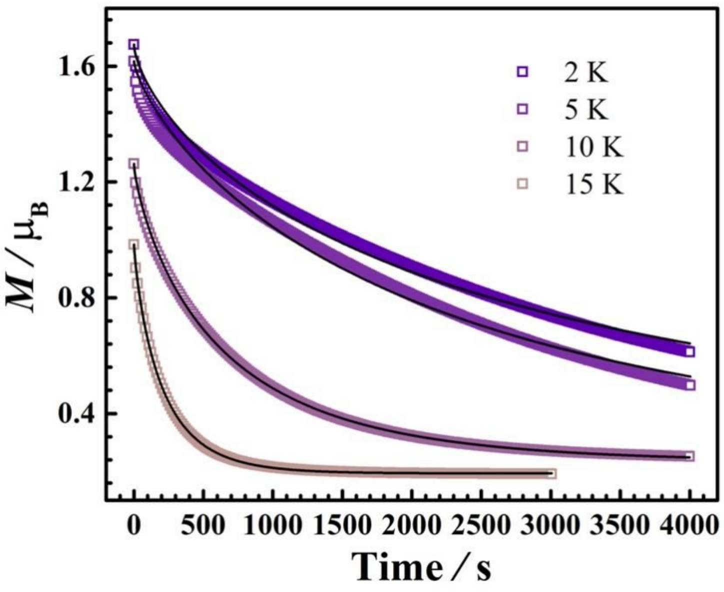

Figure S117 Dc magnetic relaxation data for **3-Tb** collected from 2-15 K under 1500 Oe field. The black line represents the fit to the data using a stretched exponential function, which was used to extract τ^*.

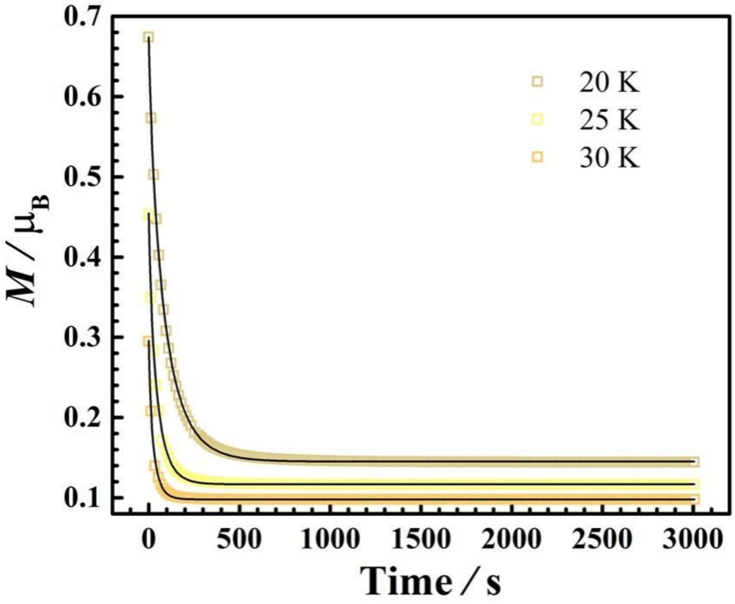

Figure S118 Dc magnetic relaxation data for **3-Tb** collected from 20-30 K under 1500 Oe field. The black line represents the fit to the data using a stretched exponential function, which was used to extract τ^*.

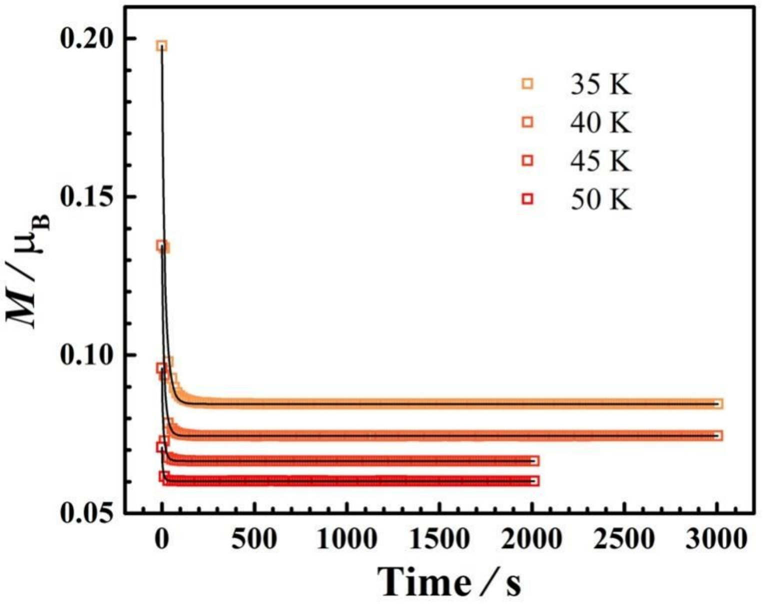

Figure S119 Dc magnetic relaxation data for **3-Tb** collected from 35-50 K under 1500 Oe field. The black line represents the fit to the data using a stretched exponential function, which was used to extract τ^*.

Table S19 Parameters used to fit dc magnetic relaxation data for **3-Tb** at 1500 Oe applied field and magnetic relaxation times extracted from these fits.

T	$\boldsymbol{M_0}$	$\boldsymbol{M_0^{err}}$	$\boldsymbol{M_{eq}}$	$\boldsymbol{M_{eq}^{err}}$	$\boldsymbol{\tau^*}$	$\boldsymbol{\tau^{*\,err}}$	β	β^{err}
(K)	(μ_B)		(μ_B)		(s)			
2*	1.67494	0	0.26262	0	2699.94	11.79	0.69446	0.00471
5*	1.61723	0	0.23376	0	2266.13	9.52	0.76863	0.0055
10*	1.26315	0	0.21087	0	692.01	1.80	0.74473	0.00224
15	0.98325	0	0.19319	1.39E-4	209.04	0.26	0.83834	0.00134
20	0.67397	0	0.14524	1.05E-4	80.86	0.23	0.84658	0.00292
25	0.45271	0	0.11685	7.20E-5	41.22	0.25	0.85314	0.0053
30	0.29505	0	0.09802	5.81E-5	24.02	0.26	0.83557	0.00978
35	0.19762	0	0.07445	8.71E-6	11.77	0.01	0.83558	0.00991

* M_{eq} values were fixed based on field-cooled susceptibility.

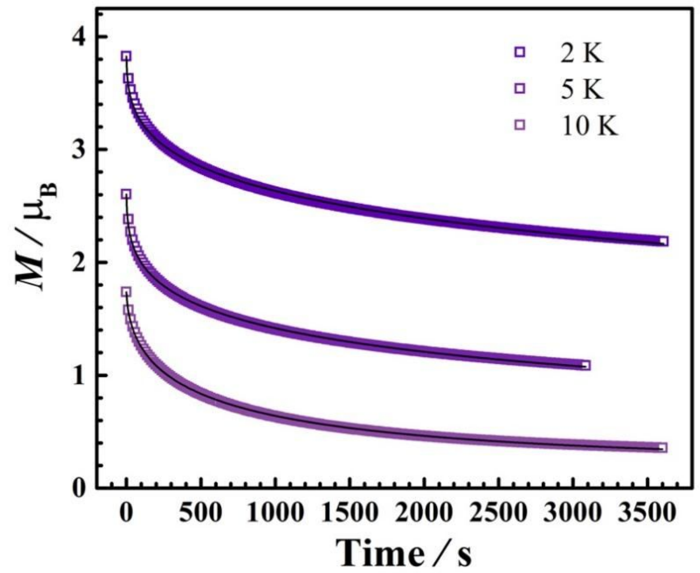

Figure S120 Dc magnetic relaxation data for **3-Dy** collected from 2-10 K under 1000 Oe field. The black line represents the fit to the data using a stretched exponential function, which was used to extract τ^*.

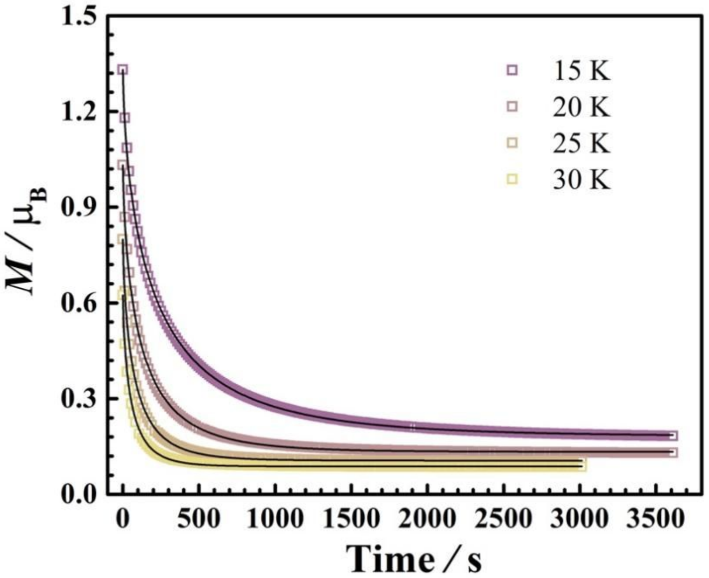

Figure S121 Dc magnetic relaxation data for **3-Dy** collected from 15-30 K under 1000 Oe field. The black line represents the fit to the data using a stretched exponential function, which was used to extract τ^*.

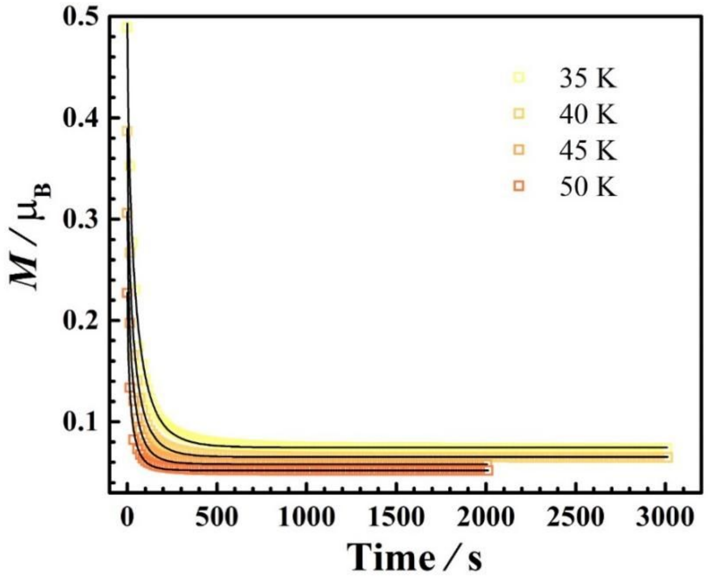

Figure S122 Dc magnetic relaxation data for **3-Dy** collected from 35-50 K under 1000 Oe field. The black line represents the fit to the data using a stretched exponential function, which was used to extract τ^*.

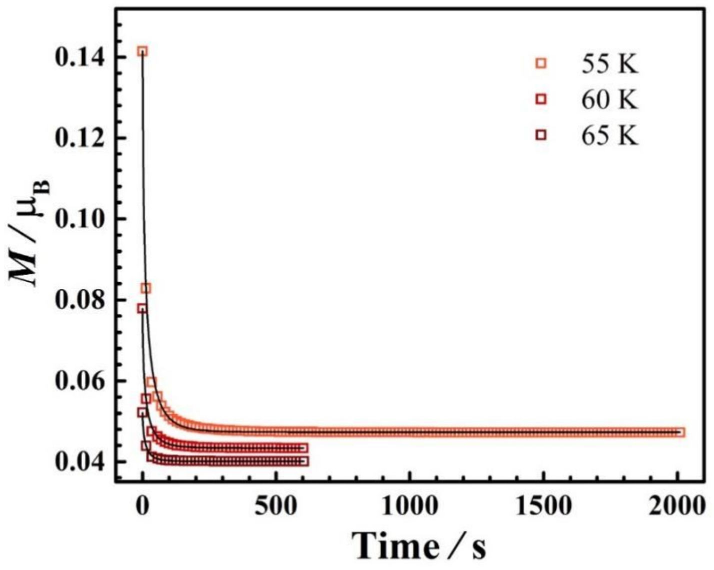

Figure S123 Dc magnetic relaxation data for **3-Dy** collected from 55-65 K under 1000 Oe field. The black line represents the fit to the data using a stretched exponential function, which was used to extract τ^*.

Table S20 Parameters used to fit dc magnetic relaxation data for **3-Dy** at 1000 Oe applied field and magnetic relaxation times extracted from these fits.

T	$\boldsymbol{M_0}$	$\boldsymbol{M_0^{err}}$	$\boldsymbol{M_{eq}}$	$\boldsymbol{M_{eq}^{err}}$	$\boldsymbol{\tau^*}$	$\boldsymbol{\tau^{*\,err}}$	β	β^{err}
(K)	(μ_B)		(μ_B)		(s)			
2*	3.8252	0	0.341	0	12551.2<br>0	131.32	0.34588	0.00174
5*	2.60403	0	0.2643	0	2605.62	11.12	0.36441	0.0016
10*	1.73788	0	0.1969	0	652.63	2.05	0.50268	0.00152
15	1.33064	0	0.18289	4.56E-4	227.65	0.59	0.63001	0.00172
20	1.03198	0	0.13356	3.12E-4	125.00	0.51	0.65942	0.00274
25	0.7996	0	0.1059	2.61E-4	81.11	0.45	0.68313	0.00384
30	0.62435	0	0.08756	1.98E-4	58.47	0.39	0.70007	0.00482
35	0.489	0	0.07475	1.51E-4	43.24	0.35	0.69576	0.00564
40	0.38697	0	0.06533	1.25E-4	33.92	0.47	0.68976	0.00804
45	0.3061	0	0.05834	1.37E-4	26.66	0.46	0.69087	0.01039
50	0.22711	0	0.05209	8.82E-5	19.29	0.38	0.6625	0.01119
55	0.14148	0	0.04729	3.55E-5	13.40	0.26	0.59023	0.00872
60	0.07786	0	0.04338	3.79E-5	12.47	0.35	0.6144	0.01488
65	0.05218	0	0.0401	1.12E-5	10.99	0.30	0.66582	0.01784

* M_{eq} values were fixed based on field-cooled susceptibility.

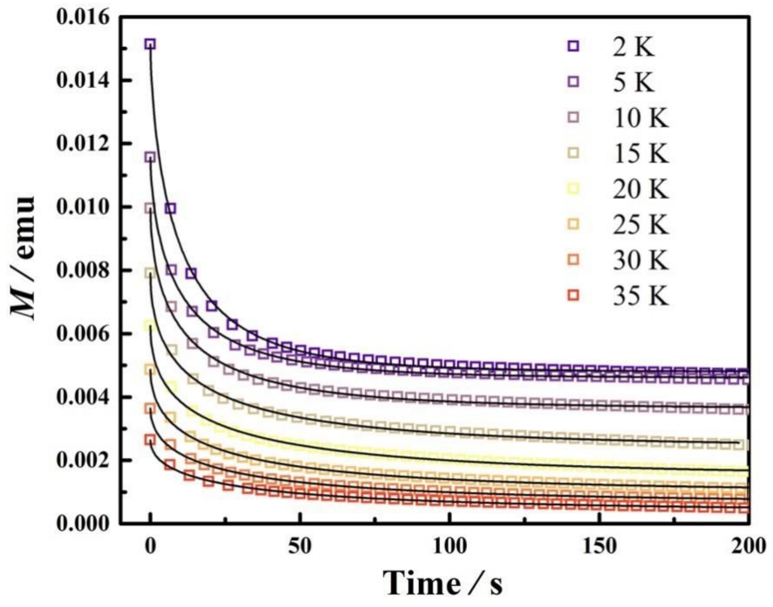

Figure S124 Dc magnetic relaxation data for a 50 mM solution of **3-Tb** in hexane collected from 2-35 K under 0 Oe field. The black line represents the fit to the data using a stretched exponential function, which was used to extract τ^*.

Table S21 Parameters used to fit dc magnetic relaxation data for a 50 mM solution of **3-Tb** in hexane at zero applied field and magnetic relaxation times extracted from these fits.

T	M_0	M_0^{err}	M_{eq}	M_{eq}^{err}	τ^*	$\tau^{*\,err}$	β	β^{err}
(K)	(emu)		(emu)		(s)			
2	0.01514	0	0.0048	1.70E-5	10.7	0.18	0.66145	0.01234
5	0.01157	0	0.0046	1.40E-5	10.9	0.20	0.62937	0.0127
10	0.00996	0	0.00364	2.24E-5	12.0	0.29	0.57227	0.01561
15	0.00791	0	0.0024	4.10E-5	16.8	0.49	0.51669	0.01654
20	0.00625	0	0.00148	3.85E-5	20.7	0.62	0.50382	0.00138
25	0.00487	0	9.90E-4	2.69E-5	21.1	0.54	0.51986	0.01233
30	0.00364	0	6.49E-4	2.07E-5	22.8	0.58	0.52166	0.01149
35	0.00264	0	3.78E-4	2.13E-5	27.6	0.95	0.5209	0.01313

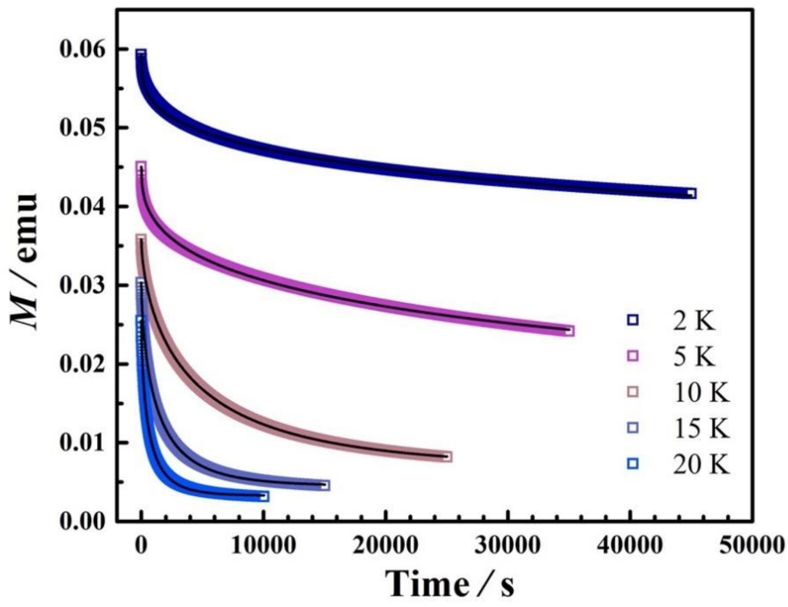

Figure S125 Dc magnetic relaxation data for a 50 mM solution of **3-Dy** in hexane collected from 2-20 K under 1000 Oe field. The black line represents the fit to the data using a stretched exponential function, which was used to extract τ^*.

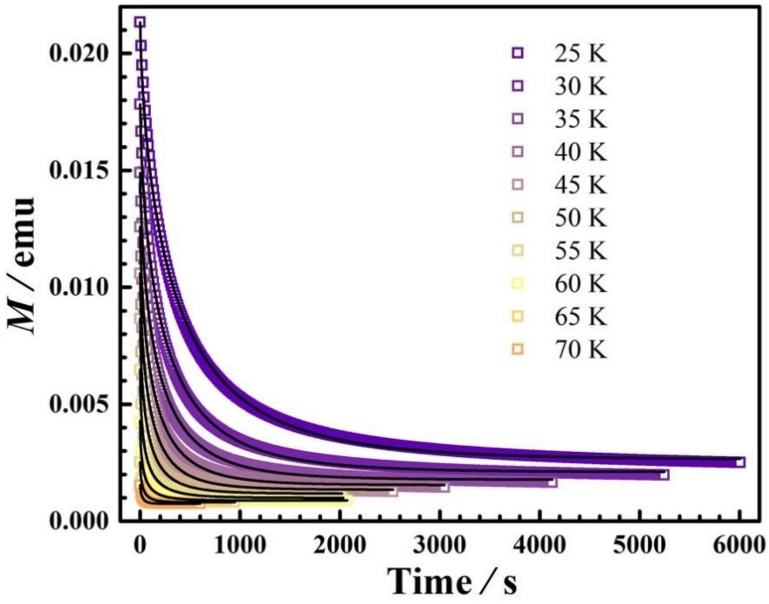

Figure S126 Dc magnetic relaxation data for a 50 mM solution of **3-Dy** in hexane collected from 25-70 K under 1000 Oe field. The black line represents the fit to the data using a stretched exponential function, which was used to extract τ^*.

Table S22 Parameters used to fit dc magnetic relaxation data for a 50 mM solution of **3-Dy** in hexane at 1000 Oe applied field and magnetic relaxation times extracted from these fits.

T	$\boldsymbol{M_0}$	$\boldsymbol{M_0^{err}}$	$\boldsymbol{M_{eq}}$	$\boldsymbol{M_{eq}^{err}}$	$\boldsymbol{\tau}^*$	$\boldsymbol{\tau}^{*\,err}$	β	β^{err}
(K)	(emu)		(emu)		(s)			
2*	0.0593	0	0.01245	0	417126.86	1280.94	0.3281	3.36E-4
5*	0.04503	0	0.00726	0	62654.37	76.51	0.39962	3.34E-4
10	0.03582	0	0.0067	4.05E-6	4564.96	2.13	0.6339	2.01E-4
15	0.03032	0	0.00448	7.50E-6	1388.15	1.90	0.65929	9.65E-4
20	0.02552	0	0.00328	7.89E-6	642.21	1.52	0.65757	0.00167
25	0.02133	0	0.00264	8.92E-6	367.98	1.24	0.6671	0.00243
30	0.01783	0	0.0021	7.89E-6	246.15	1.12	0.65582	0.00309
35	0.01489	0	0.00178	7.80E-6	179.95	1.03	0.65385	0.00386
40	0.01258	0	0.00155	7.88E-6	135.62	0.92	0.65607	0.0046
45	0.01059	0	0.00135	7.49E-6	103.94	0.85	0.64519	0.00537
50	0.00866	0	0.00118	7.10E-6	74.20	0.77	0.61805	0.00627
55	0.00648	0	0.001	4.46E-6	50.20	0.60	0.56495	0.00595
60	0.00425	0	8.83E-4	1.58E-6	34.43	0.48	0.5497	0.00479
65	0.00251	0	8.81E-4	8.24E-7	26.41	0.24	0.61063	0.00436
70	0.00153	0	7.48E-4	1.65E-7	21.29	0.07	0.72704	0.00232

* M_{eq} values were fixed based on field-cooled susceptibility.

6.4 Magnetic relaxation profiles

The representative time and width of the relaxation time distributions are defined using the expectation value ($\langle \ln\tau \rangle$) and variance ($\sigma^2_{\ln\tau}$) of the distribution of logarithmic relaxation times.[6,7] These values are calculated for relaxation time distributions characterized by different empirical formulae using equations given by Zorn.[8]

For a generalized Debye model:

$$\langle \ln\tau \rangle = \ln \tau_{\mathrm{debye}}$$

$$\sigma^2_{\ln\tau} = \frac{\pi^2}{3}\left(\frac{1}{(1-\alpha)^2} - 1\right)$$

Equation S2

For a stretched exponential function:

$$\langle \ln\tau \rangle = \ln \tau^* + (1 - \frac{1}{\beta})\mathrm{Eu}$$

$$\sigma^2_{\ln\tau} = \frac{\pi^2}{6}\left(\frac{1}{\beta^2} - 1\right)$$

Equation S3

where Eu is Euler's constant, 0.5772.... The representative (central) value of τ is given by $e^{\langle \ln\tau \rangle}$ and the one estimated standard deviation (ESD) upper and lower values are given by:[6,7]

$$\tau_{\pm} = e^{\langle \ln\tau \rangle \pm \sqrt{\sigma^2_{\ln\tau}}}$$

Equation S4

Values of $e^{\langle \ln\tau \rangle}$, $\tau_{\pm}$, $\sigma^2_{\ln\tau}$and the uncertainty $e^{\langle \ln\tau \rangle\,\mathrm{err}}$ are compared across temperatures and measurement methods in Table S23–Table S30. We use the one ESD values represent the distributions in relaxation times and are distinct from the uncertainty in the central relaxation time.

Table S23 Relaxation times and distributions for **1-Tb** at 1500 Oe applied field.

T(K)	τ_- (s)	$e^{\langle \ln\tau \rangle}$ (s)	τ_+ (s)	$e^{\langle \ln\tau \rangle}$ err (s)	$\sigma^2_{\ln\tau}$
		Ac Measurements			
6	0.12384	0.30997	0.77582	0.00356	0.84171
10	0.02648	0.05501	0.11426	4.1844E-4	0.53429
14	0.00953	0.01904	0.03806	1.3743E-4	0.47960
16	0.00652	0.01281	0.02518	8.5967E-5	0.45664
18	0.00468	0.00912	0.01779	6.2264E-5	0.44628
20	0.00351	0.00684	0.01332	4.0580E-5	0.44461
22	0.00279	0.00528	0.00999	3.1013E-5	0.40634
24	0.00217	0.00417	0.00800	2.3738E-5	0.42442
26	0.00174	0.0033	0.00626	1.6179E-5	0.40924
28	0.00133	0.00255	0.00489	1.2630E-5	0.42347
30	0.00097	0.00186	0.00358	8.6406E-6	0.43057
32	0.00065	0.00126	0.00246	4.6229E-6	0.44819
34	0.00041	8.0576E-4	0.00159	3.7424E-6	0.46521
36	0.00026	4.9553E-4	0.00096	2.6465E-6	0.43279
38	0.00016	3.0360E-4	0.00058	1.8261E-6	0.42994
40	0.00010	1.8880E-4	0.00035	2.3610E-6	0.38832

Table S24 Relaxation times and distributions for **1-Dy** at zero applied field.

T(K)	τ_- (s)	$e^{\langle \ln\tau \rangle}$ (s)	τ_+ (s)	$e^{\langle \ln\tau \rangle}$ err (s)	$\sigma^2_{\ln\tau}$
		Magnetization Decays			
2	5.52781	34.01342	209.28954	0.29457	3.30136
5	8.30954	21.94153	57.93712	0.01727	0.94280
8	5.98826	10.80313	19.48939	0.00094	0.34814
10	3.28553	5.96519	10.83037	0.00471	0.35571
		Ac Measurements			
16	0.25247	0.98808	3.86702	0.1419	1.86179
18	0.17971	0.51266	1.46250	0.03562	1.09891
20	0.12808	0.31523	0.77584	0.01428	0.81116

22	0.09230	0.20654	0.46216	0.00682	0.64869
24	0.06631	0.14095	0.29959	0.00352	0.56855
26	0.04870	0.09769	0.19596	0.00179	0.48460
28	0.03511	0.06832	0.13295	8.8893E-4	0.44326
30	0.02418	0.04628	0.08859	3.9946E-4	0.42158
32	0.01436	0.02795	0.05440	1.5712E-4	0.44350
33	0.01010	0.02017	0.04029	9.3030E-5	0.47863
34	0.00678	0.01374	0.02786	5.1613E-5	0.49959
35	0.00436	0.00882	0.01784	3.1326E-5	0.49602
36	0.00264	0.00548	0.01139	1.6099E-5	0.53479
37	0.00157	0.00333	0.00705	1.0206E-5	0.56329
38	0.00093	0.002	0.00428	5.3539E-6	0.58060
39	0.00055	0.00119	0.00256	3.7911E-6	0.58917
40	0.00034039	7.2917E-4	0.00156199	1.8891E-6	0.58034929
41	0.00020938	4.484E-4	0.00096027	1.8479E-6	0.57992988
42	0.00012768	2.8025E-4	0.00061513	1.6797E-6	0.61803683
6*	0.07976752	0.44412	2.4727179	0.04623	2.9480148
8*	0.01640291	0.0649	0.25678427	3.4566E-4	1.89169402
10*	0.00412179	0.0145	0.05100937	2.7321E-5	1.58221336
12*	0.00155245	0.00463	0.01380844	1.2459E-5	1.19404438
14*	0.00069304	0.00195	0.00548667	3.2489E-6	1.07017406
16*	0.00031693	9.6713E-4	0.00295127	1.2475E-6	1.24469284
18*	0.00017	5.4643E-4	0.00176	1.2378E-6	1.36416
20*	5.6307E-05	2.3659E-4	0.0009941	1.5662E-6	2.06068693

Table S25 Relaxation times and distributions for **2-Tb** at 1500 Oe applied field.

T(K)	τ_- (s)	$e^{\langle \ln\tau \rangle}$ (s)	τ_+ (s)	$e^{\langle \ln\tau \rangle}$ err (s)	$\sigma^2_{\ln\tau}$
		**Ac Measurements**			
10	0.13543	0.2739	0.55394	0.00218	0.49602
14	0.04101	0.09422	0.21647	0.00115	0.69193
18	0.01998	0.0409	0.08374	3.61028E-4	0.51345
20	0.01467	0.03038	0.06290	2.08257E-4	0.52960
22	0.01150	0.02283	0.04531	1.97807E-4	0.46978

24	0.00879	0.01779	0.03601	1.20522E-4	0.49740
26	0.00739	0.01421	0.02733	1.05372E-4	0.42765
28	0.00602	0.01161	0.02239	9.17591E-5	0.43152
30	0.00507	0.00974	0.01870	7.18258E-5	0.42520
32	0.00428	0.00819	0.01567	5.71099E-5	0.42134
34	0.00363	0.00692	0.01321	5.97803E-5	0.41772
36	0.00320	0.0059	0.01089	4.78491E-5	0.37530
38	0.00261	0.00497	0.00946	4.04065E-5	0.41411
40	0.00225	0.00413	0.00760	3.11447E-5	0.37129
42	0.00183	0.00337	0.00619	2.76689E-5	0.37020
44	0.00146	0.00267	0.00489	2.50516E-5	0.36527
46	0.00118754	0.00206	0.00357343	1.47504E-5	0.30340204
48	0.00089255	0.00153	0.00262271	1.446E-5	0.2904553
50	0.00069502	0.00113	0.00183721	7.35278E-6	0.23622379
52	0.00050871	8.0358E-4	0.00126937	4.99476E-6	0.20902855
54	0.0003652	5.6421E-4	0.00087167	4.69045E-6	0.18921354
56	0.00025135	3.8959E-4	0.00060387	4.36458E-6	0.19207483
58	0.00021539	2.7132E-4	0.00034177	2.8201E-6	0.05328968
60	0.00014323	1.8116E-4	0.00022913	3.63465E-6	0.05517588

Table S26 Relaxation times and distributions for **2-Dy** at zero applied field.

T(K)	τ_- (s)	$e^{\langle \ln\tau \rangle}$ (s)	τ_+ (s)	$e^{\langle \ln\tau \rangle}$ err (s)	$\sigma^2_{\ln\tau}$
		Ac Measurements			
2	0.01103	0.08085	0.59244	0.0028	3.96693
4	0.0094	0.06655	0.471	0.00255	3.8292
6	0.00977	0.05	0.25586	0.00165	2.66565
8	0.00898	0.03299	0.12125	7.04691E-4	1.69395
10	0.00685	0.01986	0.05756	2.76456E-4	1.13218
12	0.0049	0.01207	0.02973	1.14611E-4	0.8127
14	0.00352	0.00769	0.01681	5.1978E-5	0.61124
16	0.00259	0.00515	0.01026	2.57059E-5	0.4741
18	0.00197	0.00365	0.00675	1.34543E-5	0.37736
20	0.00155	0.00269	0.00466	7.54507E-6	0.30296

22	0.00126	0.00205	0.00332	4.37821E-6	0.23562
24	0.00101	0.00158	0.00247	2.81089E-6	0.19945
26	8.32262E-4	0.00125	0.00187	2.26716E-6	0.16351
28	6.86142E-4	9.91254E-4	0.00143	1.65833E-6	0.13534
30	5.71786E-4	7.91303E-4	0.0011	1.56227E-6	0.10557
31	5.19934E-4	7.06781E-4	9.60773E-4	7.64121E-6	0.09426
32	4.65939E-4	6.29119E-4	8.49448E-4	1.34238E-6	0.09016
33	4.07548E-4	5.579E-4	7.63721E-4	1.53909E-6	0.09861
34	3.92989E-4	5.04305E-4	6.47151E-4	1.07944E-6	0.0622
35	3.5605E-4	4.48815E-4	5.65748E-4	1.14959E-6	0.05361
36	3.01192E-4	3.94639E-4	5.17077E-4	1.54023E-6	0.07302
37	2.57049E-4	3.45679E-4	4.64868E-4	1.40238E-6	0.08776
38	2.45383E-4	3.11173E-4	3.94603E-4	1.11488E-6	0.05167421
39	1.74938E-4	2.62222E-4	3.93055E-4	1.19698E-6	0.00895764
40	1.79045E-4	2.39272E-4	3.19759E-4	1.1048E-6	0.04488859
41	1.47447E-4	2.07438E-4	2.91837E-4	1.32107E-6	0.02778178
42	1.23894E-4	1.8147E-4	2.65804E-4	1.46001E-6	0.05841309

Table S27 Relaxation times and distributions for crystalline sample of **3-Tb**.

T(K)	τ_- (s)	$e^{\langle \ln\tau \rangle}$ (s)	τ_+ (s)	$e^{\langle \ln\tau \rangle}$ err (s)	$\sigma^2_{\ln\tau}$
**Magnetization Decays (0 Oe)**					
5	1.29390	4.40834	15.01932	0.24915	1.50268
10	0.50421	2.92838	17.00762	0.05879	3.09483
15	0.47696	2.88519	17.45278	0.04191	3.23967
20	0.54078	3.07296	17.46208	0.04585	3.01852
25	0.48118	2.91612	17.67295	0.04403	3.24641
30	0.53987	3.05674	17.30727	0.04342	3.00599
35	0.59955	3.16811	16.74075	0.04146	2.77126
40	0.90276	3.71720	15.30590	0.04544	2.00298
**Magnetization Decays (1500 Oe)**					
2	554.58252	2094.42734	7909.78028	9.14587	1.76576
5	655.04512	1904.70481	5538.39771	8.00165	1.13930
10	179.91178	567.79158	1791.91868	1.47689	1.32086

15	81.22623	187.02123	430.61139	1.48768	0.69553
20	32.51731	72.82922	163.11603	0.23261	0.65019
25	17.03591	37.32129	81.76133	0.20716	0.61503
30	9.22623	21.44091	49.82674	0.22635	0.71107
35	4.52112	10.50631	24.41490	0.23208	0.71102
Ac Measurements (0 Oe)					
65	0.34876	0.53739	0.82805	0.00301	0.18693
70	0.18908	0.31174	0.51396	0.00158	0.24998
75	0.09866	0.159	0.25625	8.1997E-4	0.22778
80	0.04748	0.07348	0.11372	4.60807E-4	0.19072
85	0.02102	0.03104	0.04583	1.4182E-4	0.15177
88	0.01267	0.01811	0.02588	9.3787E-5	0.12750
91	0.00739	0.01037	0.01455	5.3671E-5	0.11473
94	0.00433	0.00603	0.00839	3.0021E-5	0.10947
97	0.00257	0.00355	0.00490	1.9180E-5	0.10430
100	0.00145	0.00211	0.00307	1.0877E-5	0.14034
103	0.00084	0.00127	0.00191	7.3654E-6	0.16638
106	0.00054	7.76096E-4	0.00111	5.5593E-6	0.13007
109	0.00032	4.77155E-4	0.00070	3.2128E-6	0.15220
112	0.00020	2.99004E-4	0.00045	4.9603E-6	0.16242
115	0.00012	1.88617E-4	0.00029	3.9102E-6	0.17240
Ac Measurements (1500 Oe)					
60	0.49764	1.02943	2.12951	3.7911E-6	0.52837
65	0.31598	0.56613	1.01433	1.8891E-6	0.34007
70	0.18537	0.30467	0.50076	1.8479E-6	0.24690
75	0.10513	0.15305	0.22281	1.6797E-6	0.14104
80	0.04714	0.06954	0.10259	0.04623	0.15121
85	0.01979	0.02986	0.04506	3.4566E-4	0.16928
90	0.00859	0.01189	0.01645	2.7321E-5	0.10554
95	0.00378	0.00487	0.00628	1.2459E-5	0.06456
100	0.00147	0.00205	0.00286	3.2489E-6	0.11079
105	0.00067	8.81E-04	0.00116	1.2475E-6	0.07481
110	0.00027	3.96E-04	0.00058	1.2378E-6	0.14020

115	0.00013	1.85E-04	0.00026	1.5662E-6	0.11224

Table S28 Relaxation times and distributions for crystalline sample of **3-Dy**.

T(K)	$\boldsymbol{\tau_-}$ (s)	$\boldsymbol{e^{\langle \ln\tau \rangle}}$ (s)	$\boldsymbol{\tau_+}$ (s)	$\boldsymbol{e^{\langle \ln\tau \rangle}}$ **err** (s)	$\boldsymbol{\sigma^2_{\ln\tau}}$
		Magnetization Decays (1000 Oe)			
2	129.91227	4213.22933	136640.68562	44.08194	12.10431
5	35.91873	952.12444	25238.66860	4.06338	10.74159
10	40.62496	368.69323	3346.08792	1.15812	4.86459
15	33.37898	162.20053	788.19099	0.42037	2.49927
20	21.50011	92.77703	400.35036	0.37853	2.13786
25	15.75254	62.05815	244.48209	0.34430	1.87983
30	12.34253	45.66010	168.91555	0.30456	1.71132
35	8.93834	33.59474	126.26584	0.27193	1.75303
40	6.80827	26.16420	100.54910	0.36253	1.81240
45	5.38044	20.59186	78.80852	0.35530	1.80130
50	3.37181	14.37573	61.29102	0.28319	2.10277
55	1.55345	8.97579	51.86181	0.17416	3.07670
60	1.67213	8.68044	45.06229	0.24364	2.71252
65	1.95444	8.22588	34.62114	0.22455	2.06549
		Ac Measurements (0 Oe)			
5	0.00768	0.04649	0.28155	0.00255	3.24392
8	0.00660	0.03232	0.15823	8.4182E-4	2.52299
11	0.00467	0.02447	0.12813	3.88324E-4	2.74105
14	0.00324	0.02099	0.13587	2.11306E-4	3.48826
17	0.00191	0.01796	0.16929	5.00471E-4	5.03302
21	0.00140	0.01786	0.22775	0.00108	6.48049
23	0.00157	0.01886	0.22595	0.00162	6.16667
25	0.00267	0.0193	0.13932	0.00111	3.90730
30	0.00387	0.01858	0.08923	7.54456E-4	2.46215
35	0.00426	0.01681	0.06628	2.81186E-4	1.88222
40	0.00354	0.01649	0.07691	3.59051E-4	2.37124
45	0.00461	0.01817	0.07154	4.92778E-4	1.87820
50	0.00627	0.01911	0.05825	2.66429E-4	1.24203

55	0.00691	0.01937	0.05430	1.59803E-4	1.06236
60	0.00717	0.01984	0.05491	1.70439E-4	1.03616
65	0.00738	0.02009	0.05467	1.70207E-4	1.00206
70	0.00760706	0.01971	0.05106892	1.87741E-4	0.90639915
74	0.00762687	0.01958	0.05026656	1.98623E-4	0.88893088
76	0.00755172	0.0191	0.04830817	1.68619E-4	0.86102147
78	0.00757737	0.01819	0.04366635	1.4421E-4	0.76686082
80	0.00734297	0.01764	0.04237652	1.50752E-4	0.76812154
82	0.00705365	0.01662	0.03916048	1.51627E-4	0.73455425
84	0.00669984	0.01527	0.03480275	1.27587E-4	0.67865667
86	0.00612182	0.01368	0.03056971	1.31847E-4	0.64653616
88	0.00545884	0.01182	0.02559379	1.0218E-4	0.59684402
90	0.00473671	0.00995	0.02090112	1.03333E-4	0.55090587
92	0.00383409	0.008	0.01669235	6.2113E-5	0.54097332
94	0.00303079	0.00622	0.01276511	4.91118E-5	0.51688279
96	0.00227534	0.00473	0.00983278	3.59446E-5	0.53552685
98	0.00168	0.00347	0.00716719	2.18905E-5	0.52614666
100	0.0012252	0.0025	0.00510119	2.4663E-5	0.50863123
102	0.00086561	0.00177	0.00361929	1.29928E-5	0.51165096
104	0.00062991	0.00126	0.00252035	1.03967E-5	0.48064683
106	0.00043252	8.82759E-4	0.00180166	6.29003E-6	0.50895752
108	0.00031557	6.25387E-4	0.00123938	3.63895E-6	0.46785376
110	0.00021062	4.39196E-4	0.00091584	3.59482E-6	0.54006477
112	0.00015206	3.12809E-4	0.0006435	4.15799E-6	0.52032205
114	0.00010862	2.22884E-4	0.00045737	3.998E-6	0.51671913
116	8.1969E-05	1.60958E-4	0.00031607	3.18671E-6	0.45536529
			Ac Measurements (1000 Oe)		
84	0.02592	0.05368	0.11116	7.01275E-4	0.52985
88	0.01364	0.02694	0.05320	2.56095E-4	0.46304
92	0.00624	0.01311	0.02753	1.34128E-4	0.55016
96	0.00305	0.00607	0.01207	5.00649E-5	0.47203
100	0.00144	0.00282	0.00552	2.42424E-5	0.45050
104	0.00066	0.00134	0.00271	1.15857E-5	0.49699

108	0.00032	6.48E-04	0.00129	5.14085E-6	0.47702
112	0.00015	3.16E-04	0.00065	3.46965E-6	0.52680
116	0.00008	1.61E-04	0.00032	4.71901E-6	0.48533

Table S29 Relaxation times and distributions for 50 mM solution sample of **3-Tb** in hexane.

T(K)	τ_- (s)	$e^{\langle\ln\tau\rangle}$ (s)	τ_+ (s)	$e^{\langle\ln\tau\rangle}$ err (s)	$\sigma^2_{\ln\tau}$
		Magnetization Decays (0 Oe)			
2	1.86009	7.96308	34.09010	0.13554	2.11467
5	1.59247	7.75901	37.80446	0.14429	2.50770
10	1.24065	7.79507	48.97677	0.18820	3.37771
15	1.16917	9.79104	81.99383	0.28507	4.51637
20	1.30055	11.72457	105.69822	0.34987	4.83517
25	1.50485	12.38114	101.86534	0.31790	4.44146
30	1.64870	13.43002	109.39870	0.34056	4.39953
35	1.98422	16.23117	132.77330	0.56011	4.41718
		Ac Measurements (0 Oe)			
61	0.38431	0.91551	2.18094	0.01381	0.75348
64	0.31975	0.69613	1.51557	0.00838	0.60530
67	0.25443	0.5487	1.18330	0.00705	0.59060
70	0.19494	0.39533	0.80173	0.00527	0.49992
73	0.12353	0.28048	0.63686	0.0045	0.67248
76	0.09176	0.18822	0.38608	0.00202	0.51615
79	0.05800	0.11673	0.23492	0.00121	0.48913
82	0.03483	0.06994	0.14044	7.81203E-4	0.48598
85	0.02002	0.0417	0.08687	4.35901E-4	0.53866
88	0.01186	0.02449	0.05058	2.41108E-4	0.52598
91	0.00678	0.0138	0.02809	1.19181E-4	0.50521
94	0.00381	0.008	0.01681	9.71546E-5	0.55124
97	0.00226	0.00457	0.00923	4.40545E-5	0.49440
100	0.00140	0.0027	0.00521	2.81148E-5	0.43089
103	0.00085	0.00163	0.00314	1.95351E-5	0.42828
106	0.00055	0.001	0.00181	1.27163E-5	0.35077
109	0.0003308	6.14829E-4	0.00114275	7.06233E-6	0.38420811

112	0.00021093	3.88822E-4	0.00071675	8.86111E-6	0.37406521
115	0.00012473	2.40876E-4	0.00046516	7.22934E-6	0.43310586

Table S30 Relaxation times and distributions for 50 mM solution sample of **3-Dy** in hexane.

T(K)	τ_- (s)	$e^{\langle \ln\tau \rangle}$ (s)	τ_+ (s)	$e^{\langle \ln\tau \rangle}$ err (s)	$\sigma^2_{\ln\tau}$
Magnetization Decays (1000 Oe)					
2	3186.20772	127915.59676	5135383.91983	392.81144	13.63485
5	1388.90534	26323.47898	498900.48304	32.14476	8.65504
10	684.04478	3270.87010	15640.19142	1.52618	2.44856
15	238.60008	1030.12964	4447.47159	1.40997	2.13935
20	109.39076	475.48631	2066.78549	1.12539	2.15917
25	65.87564	275.88690	1155.41315	0.92967	2.05126
30	41.54216	181.82080	795.78916	0.82730	2.17950
35	30.05410	132.56960	584.76880	0.75880	2.20258
40	22.91856	100.21047	438.16614	0.67979	2.17659
45	16.57108	75.67090	345.54694	0.61882	2.30656
50	10.16285	51.93844	265.43752	0.53898	2.66121
55	4.94500	32.18600	209.49200	0.38469	3.50871
60	3.05597	21.45807	150.67193	0.29915	3.79862
65	3.46396	18.27784	96.44440	0.16610	2.76649
70	5.10572	17.14203	57.55299	0.05636	1.46694
Ac Measurements (0 Oe)					
11	0.00330	0.08264	2.06897	0.50944	10.37042
14	0.00184	0.04905	1.30802	0.51665	10.78089
17	0.00101	0.04437	1.94592	0.56765	14.29542
20	0.00056	0.05815	6.05278	0.63644	21.57832
23	0.00061	0.06937	7.85534	0.64208	22.36812
25	0.00095	0.06659	4.66328	0.60756	18.05332
30	0.00222	0.03644	0.59840	0.45632	7.83210
35	0.00310	0.02926	0.27599	0.3716	5.03619
40	0.00274	0.0285	0.29603	0.38765	5.47820
45	0.00362	0.02937	0.23853	0.34556	4.38705
50	0.00482	0.0314	0.20454	0.3047	3.51166

55	0.00523	0.03071	0.18034	0.28453	3.13376
60	0.00543	0.03043	0.17054	0.27526	2.97057
65	0.00541	0.0341	0.21511	0.29852	3.39241
70	0.00559	0.0325	0.18879	0.28239	3.09553
72	0.00602	0.03042	0.15365	0.25425	2.62298
74	0.00550528	0.02883	0.15097669	0.26161	2.74137207
76	0.00537482	0.02855	0.15165198	0.26449	2.78867084
78	0.00646372	0.02855	0.12610415	0.2265	2.2065654
80	0.00543688	0.02696	0.13368729	0.25048	2.56367847
82	0.00639603	0.02397	0.08983089	0.19183	1.7453735
84	0.00559105	0.02494	0.11124994	0.22856	2.23594135
86	0.00489394	0.02074	0.08789386	0.21782	2.08532595
88	0.00412682	0.01728	0.07235548	0.21529	2.05074274
90	0.00425771	0.01477	0.05123712	0.17543	1.54720327
92	0.0033441	0.01204	0.04334842	0.18332	1.64105254
94	0.002678	0.00968	0.03498971	0.18416	1.65120478
96	0.00193508	0.00766	0.03032199	0.20343	1.89299424
98	0.00156417	0.00547	0.01912895	0.17714	1.56731429
100	0.00110423	0.00407	0.01500129	0.1883	1.70170217
102	0.0008809	0.00299	0.01014882	0.17081	1.49348904
104	0.00057146	0.00207	0.00749818	0.18461	1.65665639
106	0.00038042	0.0014	0.00515213	0.18797	1.69764867
108	0.00030816	9.74842E-4	0.00308387	0.15592	1.32633248
110	0.00019122	6.85245E-4	0.00245554	0.18232	1.6290073
112	0.00016202	4.76857E-4	0.00140348	0.14079	1.16530511
114	0.00010254	3.36079E-4	0.00110151	0.1634	1.40918805
Ac Measurements (1000 Oe)					
74	0.18302	0.74905	3.06569	0.04698	1.98591
76	0.12266	0.50683	2.09430	0.02837	2.01299
78	0.10306	0.35727	1.23849	0.01382	1.54545
80	0.06715	0.27847	1.15489	0.01097	2.02337
82	0.05103	0.20588	0.83062	0.00772	1.94567
84	0.03216	0.13304	0.55045	0.00568	2.01663

86	0.02450	0.09586	0.37509	0.00342	1.86128
88	0.01739	0.05797	0.19329	0.00255	1.45032
90	0.01078	0.03589	0.11951	9.76243E-4	1.44702
92	0.00765	0.02653	0.09205	9.68513E-4	1.54767
94	0.00515	0.01826	0.06470	5.94749E-4	1.60052
96	0.00312	0.01149	0.04229	3.0119E-4	1.69826
98	0.00225	0.00836	0.03109	2.5491E-4	1.72489
100	0.00146	0.00538	0.01982	1.10139E-4	1.70010
102	0.00092	0.00343	0.01284	1.02583E-4	1.74189
104	0.00071	0.00235	0.00783	5.54549E-5	1.44895
106	0.00046888	0.00165	0.00580643	5.68132E-5	1.5830431
108	0.00028083	0.00107	0.00407688	2.74578E-5	1.78936851
110	0.00018532	6.97008E-4	0.00262151	2.30438E-5	1.75485084
112	0.00012972	4.79672E-4	0.00177374	1.97504E-5	1.71019368
114	0.00010086	3.20397E-4	0.00101775	1.59755E-5	1.33585624
116	5.2344E-05	1.97725E-4	0.00074689	2.07141E-5	1.76635927

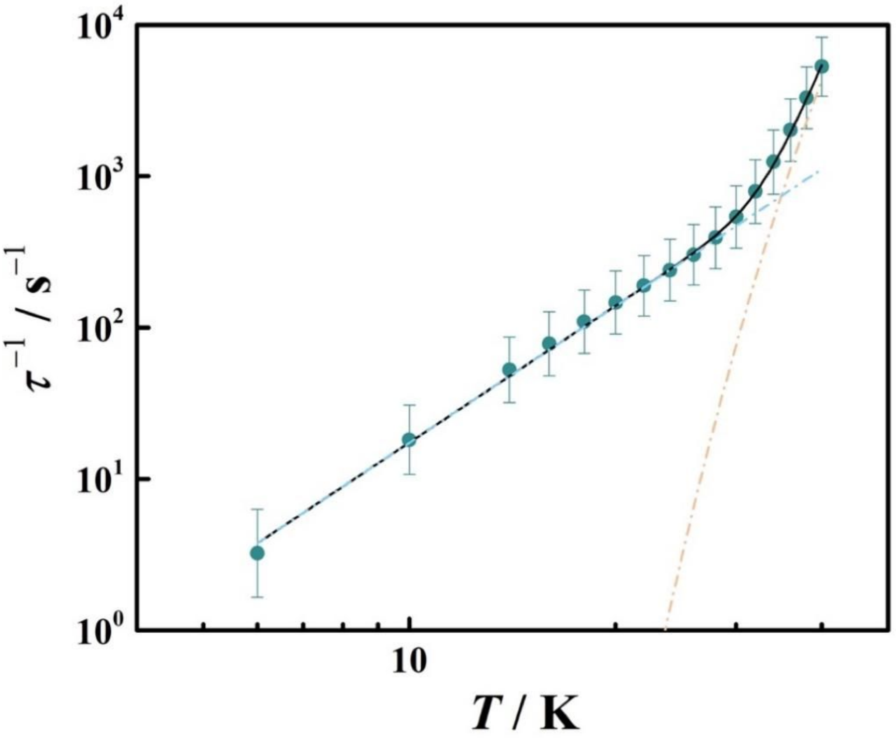

Figure S127 Plot of natural log of the inverse relaxation time *vs*. temperature for crystalline **1-Tb** under 1500 Oe dc magnetic field (cyan points are from ac data). The dashed orange line shows $\tau^{-1} = \tau_0{}^{-1}e^{-U_{eff}/T}$, where U_{eff} = 491 ± 32 K and τ_0 = $10^{-8.970 \pm 0.370}$ (1.27×10^{-9}) s, the dashed blue line shows $\tau^{-1} = CT^n$, where C = $10^{-1.813 \pm 0.058}$ (1.54×10^{-2}) s^{-1} K^{-n} and n = 3.049 ± 0.047. Black solid line shows their sum with $\tau^{-1} = \tau_0{}^{-1}e^{-U_{eff}/T} + CT^n$.

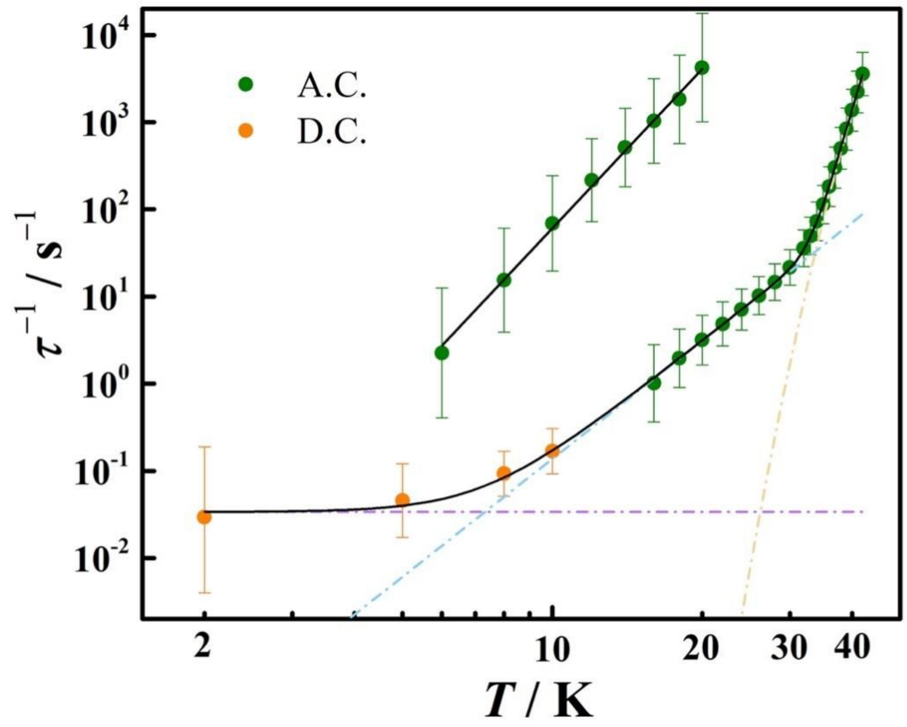

Figure S128 Plot of natural log of the inverse relaxation time *vs*. temperature for crystalline **1-Dy** under zero dc magnetic field (green points are from ac data; orange points are from dc data). The dashed orange line shows $\tau^{-1} = \tau_0{}^{-1}e^{-U_{eff}/T}$, where U_{eff} = 800 ± 16 K and $\tau_0 = 10^{-11.802 \pm 0.177}$ (1.58 × 10^{-12}) s, the dashed blue line shows $\tau^{-1} = CT^n$, where $C = 10^{-5.360 \pm 0.088}$ (4.94 × 10^{-6}) s^{-1} K^{-n} and n = 4.498 ± 0.066, the dashed purple line shows $\tau^{-1} = \tau^{-1}{}_{QTM}$, where $\tau_{QTM} = 10^{1.471 \pm 0.022}$ (29.6) s. Black solid line shows their sum with $\tau^{-1} = \tau_0{}^{-1}e^{-U_{eff}/T} + CT^n + \tau^{-1}{}_{QTM}$ for slow relaxation process and $\tau^{-1} = CT^n$, where $C = 10^{-4.356 \pm 0.136}$ (4.41 × 10^{-5}) s^{-1} K^{-n} and n = 6.135 ± 0.124 for fast relaxation respectively.

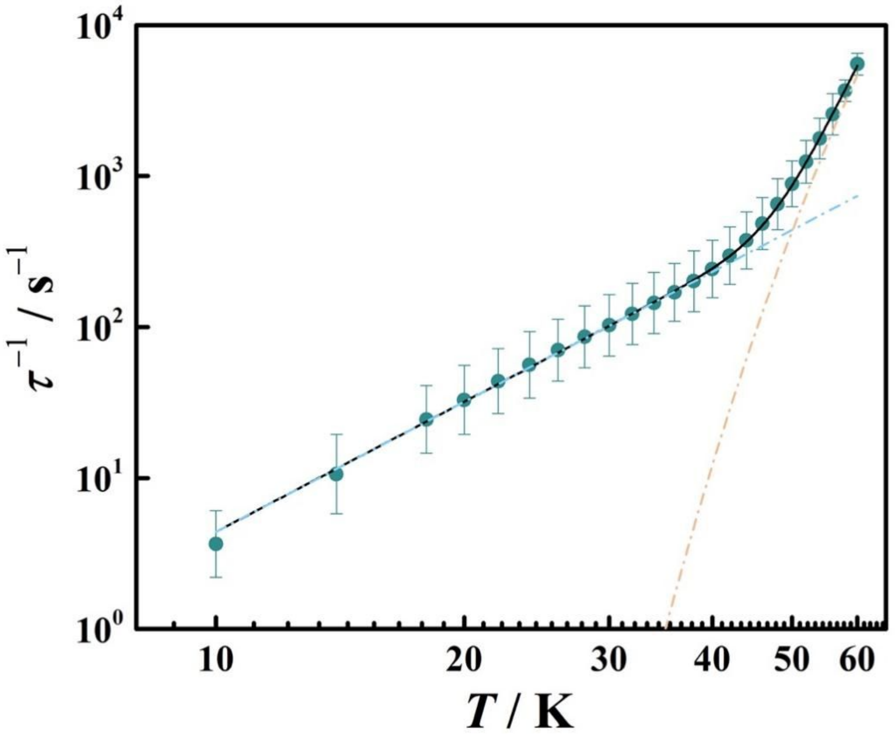

Figure S129 Plot of natural log of the inverse relaxation time *vs*. temperature for crystalline **2-Tb** under 1500 Oe dc magnetic field (cyan points are from ac data). The dashed orange line shows $\tau^{-1} = \tau_0{}^{-1}e^{-U_{eff}/T}$, where U_{eff} = 725 ± 21 K and τ_0 = $10^{-8.913 \pm 0.164}$ (1.22×10^{-9}) s, the dashed blue line shows $\tau^{-1} = CT^n$, where $C = 10^{-2.344 \pm 0.042}$ (4.53×10^{-3}) s^{-1} K^{-n} and n = 2.949 ± 0.03. Black solid line shows their sum with $\tau^{-1} = \tau_0{}^{-1}e^{-U_{eff}/T} + CT^n$.

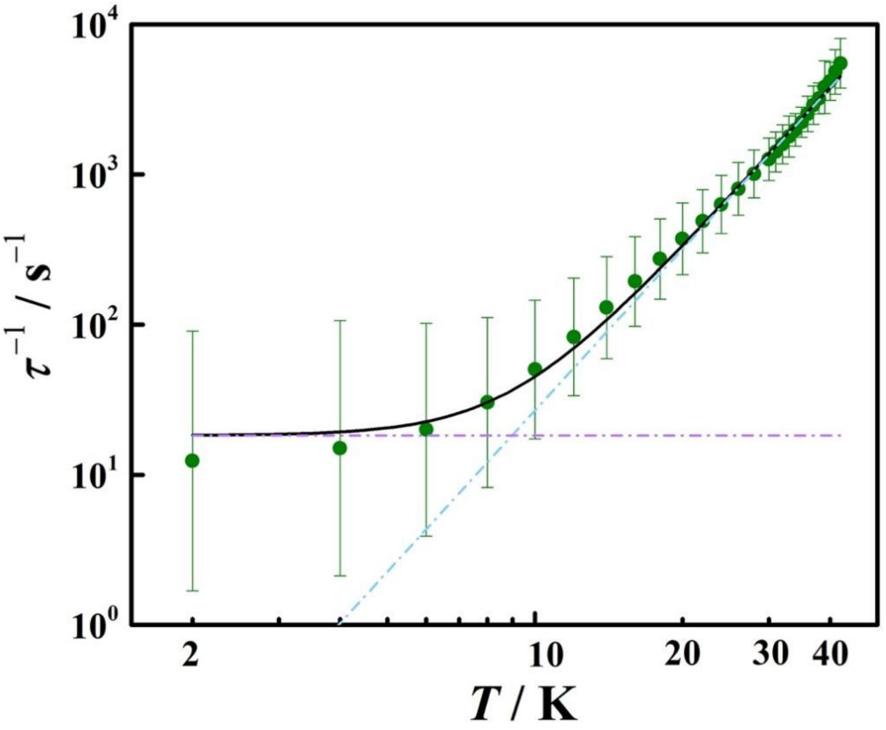

Figure S130 Plot of natural log of the inverse relaxation time *vs*. temperature for crystalline **2-Dy** under zero dc magnetic field (green points are from ac data). The dashed blue line shows $\tau^{-1} = CT^n$, where $C = 10^{-2.137 \pm 0.144}$ (7.29×10^{-3}) s^{-1} K^{-n} and $n = 3.567 \pm 0.094$, the dashed purple line shows $\tau^{-1} = \tau^{-1}{}_{QTM}$, where $\tau_{QTM} = 10^{-1.262 \pm 0.107}$ (5.47×10^{-2}) s. Black solid line shows their sum with $\tau^{-1} = CT^n + \tau^{-1}{}_{QTM}$.

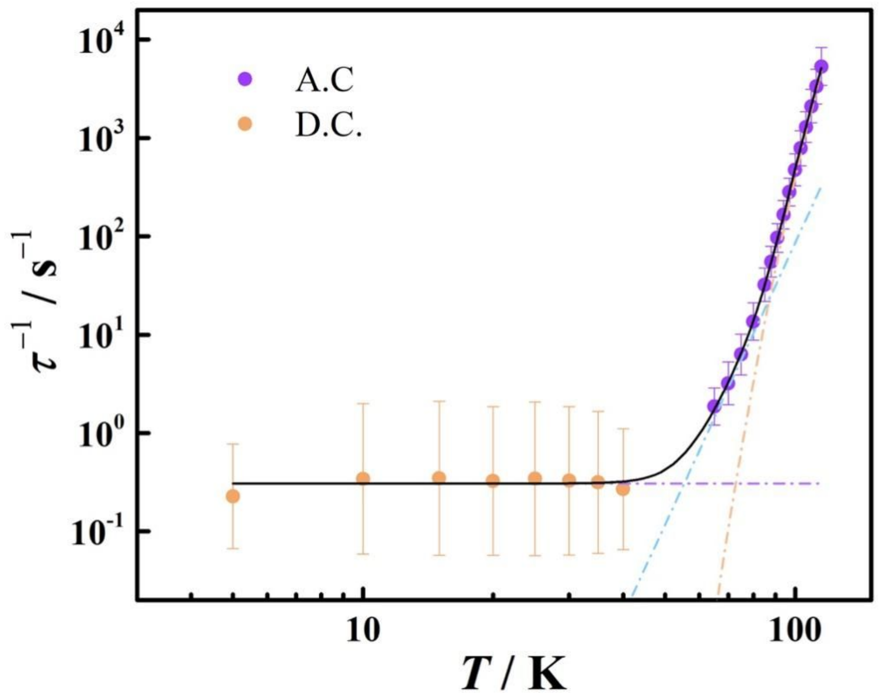

Figure S131 Plot of natural log of the inverse relaxation time *vs*. temperature for crystalline **3-Tb** under zero dc magnetic field (blue points are from ac data; orange points are from dc data). The dashed orange line shows $\tau^{-1} = \tau_0{}^{-1} e^{-U_{eff}/T}$, where U_{eff} = 1920 ± 91 K and $\tau_0 = 10^{-10.933 \pm 0.351}$ (1.17×10^{-11}) s, the dashed blue line shows $\tau^{-1} = CT^n$, where $C = 10^{-17.097 \pm 1.960}$ (8.0×10^{-18}) s^{-1} K^{-n} and n = 9.515 ± 1.063, the dashed purple line shows $\tau^{-1} = \tau^{-1}{}_{QTM}$, where $\tau_{QTM} = 10^{0.512 \pm 0.016}$ (3.25) s. Black solid line shows their sum with $\tau^{-1} = \tau_0{}^{-1} e^{-U_{eff}/T} + CT^n + \tau^{-1}{}_{QTM}$.

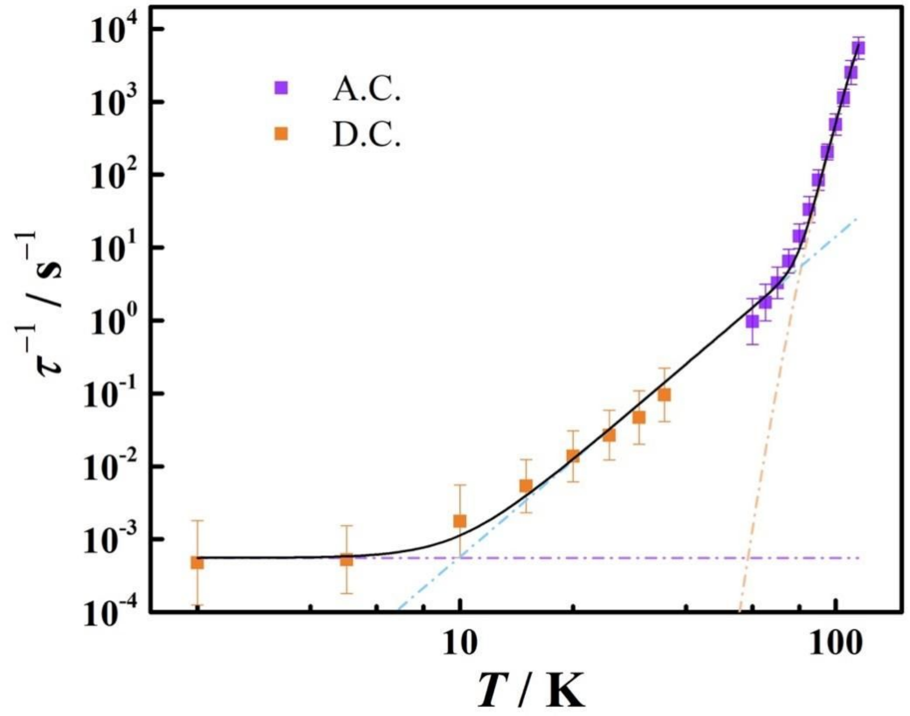

Figure S132 Plot of natural log of the inverse relaxation time *vs*. temperature for crystalline **3-Tb** under 1500 Oe dc magnetic field (blue points are from ac data; orange points are from dc data). The dashed orange line shows $\tau^{-1} = \tau_0{}^{-1}e^{-U_{eff}/T}$, where U_{eff} = 1920(fixed) K and τ_0 = $10^{-11.034 \pm 0.036}$ (9.25×10^{-12}) s, the dashed blue line shows $\tau^{-1} = CT^n$, where $C = 10^{-7.633 \pm 0.244}$ (2.33×10^{-8}) s^{-1} K^{-n} and n = 4.392 ± 0.155, the dashed purple line shows $\tau^{-1} = \tau^{-1}{}_{QTM}$, where $\tau_{QTM} = 10^{3.254 \pm 0.077}$ (1.79×10^3) s. Black solid line shows their sum with $\tau^{-1} = \tau_0{}^{-1}e^{-U_{eff}/T} + CT^n + \tau^{-1}{}_{QTM}$.

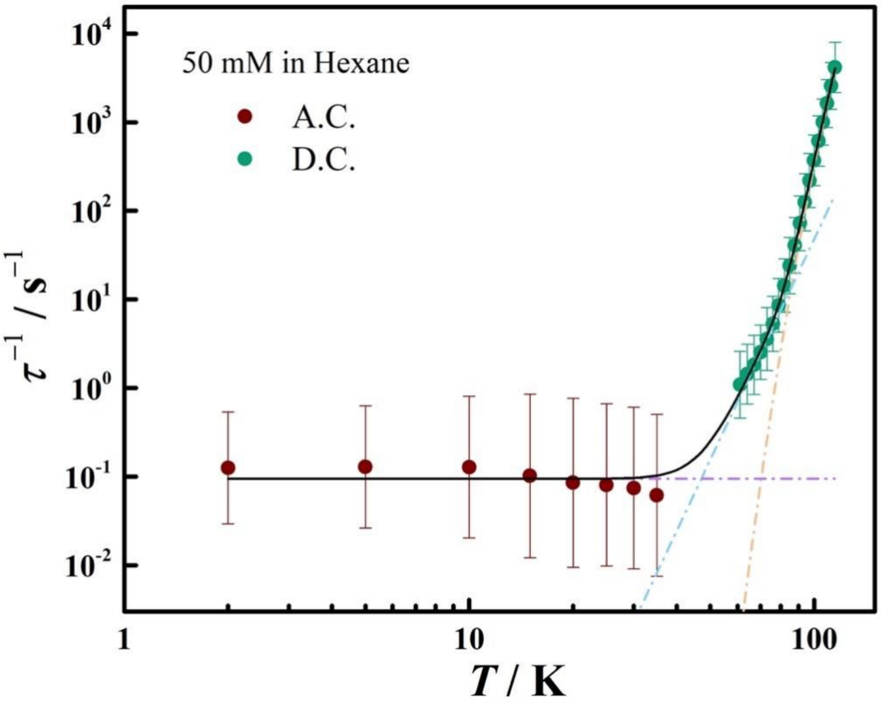

Figure S133 Plot of natural log of the inverse relaxation time *vs*. temperature for a 50 mM solution of **3-Tb** in hexane under 0 Oe dc magnetic field (brick red points are from ac data; brown points are from dc data). The dashed orange line shows $\tau^{-1} = \tau_0{}^{-1} e^{-U_{eff}/T}$, where U_{eff} = 1920(fixed) K and $\tau_0 = 10^{-10.844 \pm 0.035}$ (1.43×10^{-11}) s, the dashed blue line shows $\tau^{-1} = CT^n$, where $C = 10^{-14.842 \pm 1.328}$ (1.44×10^{-15}) s^{-1} K^{-n} and $n = 8.261 \pm 0.719$, the dashed purple line shows $\tau^{-1} = \tau^{-1}{}_{QTM}$, where $\tau_{QTM} = 10^{1.024 \pm 0.028}$ (10.6) s. Black solid line shows their sum with $\tau^{-1} = \tau_0{}^{-1} e^{-U_{eff}/T} + CT^n + \tau^{-1}{}_{QTM}$.

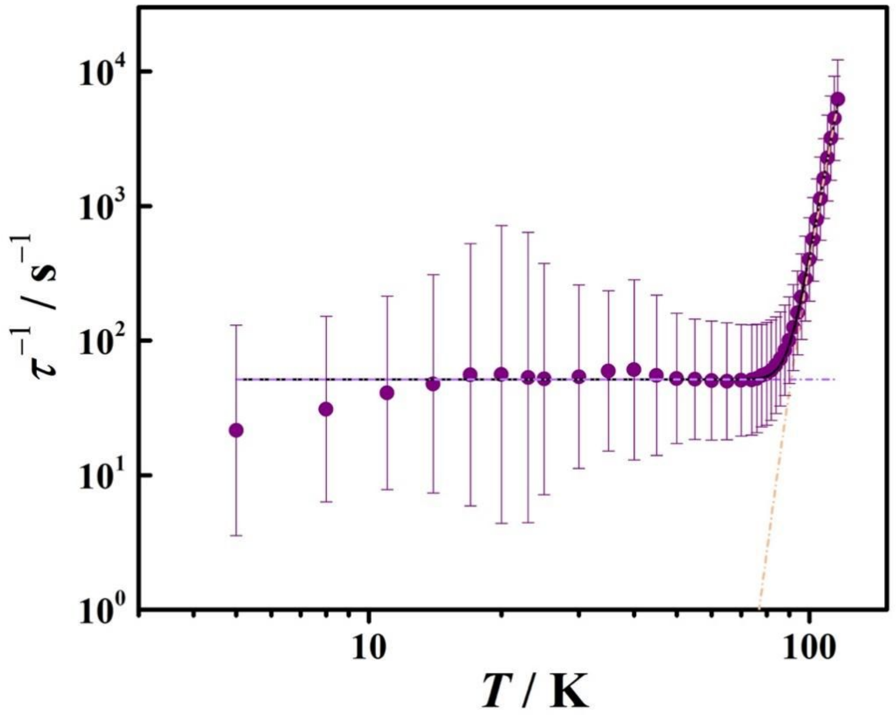

Figure S134 Plot of natural log of the inverse relaxation time *vs*. temperature for crystalline **3-Dy** under zero dc magnetic field (purple points are from ac data). The dashed orange line shows $\tau^{-1} = \tau_0{}^{-1}e^{-U_{eff}/T}$, where $U_{\text{eff}} = 1964 \pm 48$ K and $\tau_0 = 10^{-11.104 \pm 0.198}$ (7.87×10^{-12}) s, the dashed purple line shows $\tau^{-1} = \tau^{-1}{}_{QTM}$, where $\tau_{QTM} = 10^{-1.710 \pm 0.016}$ (1.95×10^{-2}) s. Black solid line shows their sum with $\tau^{-1} = \tau_0{}^{-1}e^{-U_{eff}/T} + \tau^{-1}{}_{QTM}$.

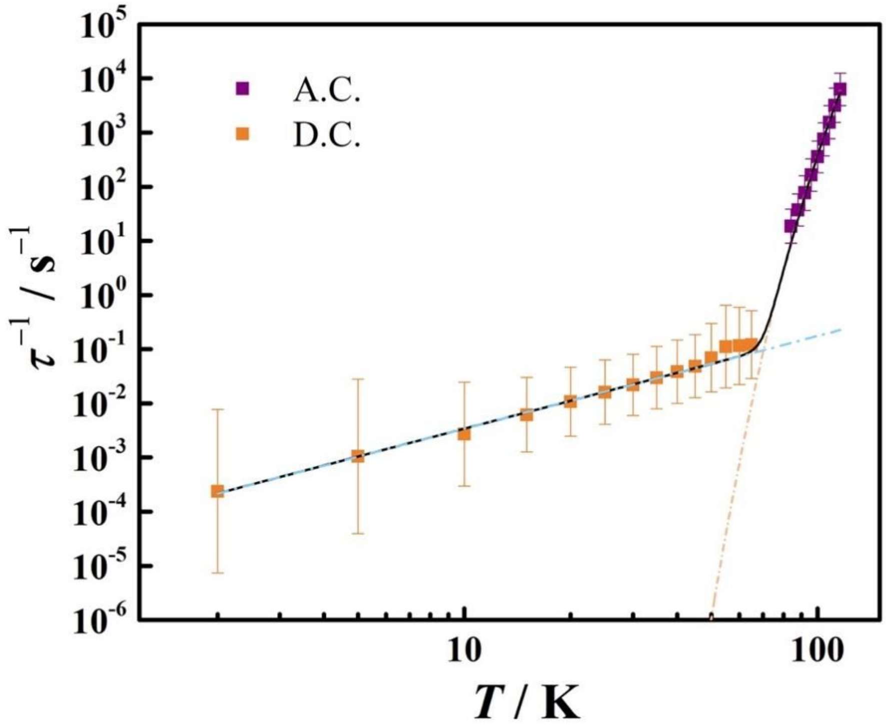

Figure S135 Plot of natural log of the inverse relaxation time *vs*. temperature for crystalline **3-Dy** under 1000 Oe dc magnetic field (purple points are from ac data; orange points are from dc data). The dashed orange line shows $\tau^{-1} = \tau_0{}^{-1}e^{-U_{eff}/T}$, where U_{eff} = 1964(fixed) K and τ_0 = $10^{-11.159 \pm 0.032}$ (6.93×10^{-12}) s, the dashed blue line shows $\tau^{-1} = CT^n$, where $C = 10^{-4.284 \pm 0.087}$ (5.20×10^{-5}) s^{-1} K^{-n} and n = 1.820 ± 0.061. Black solid line shows their sum with $\tau^{-1} = \tau_0{}^{-1}e^{-U_{eff}/T} + CT^n$.

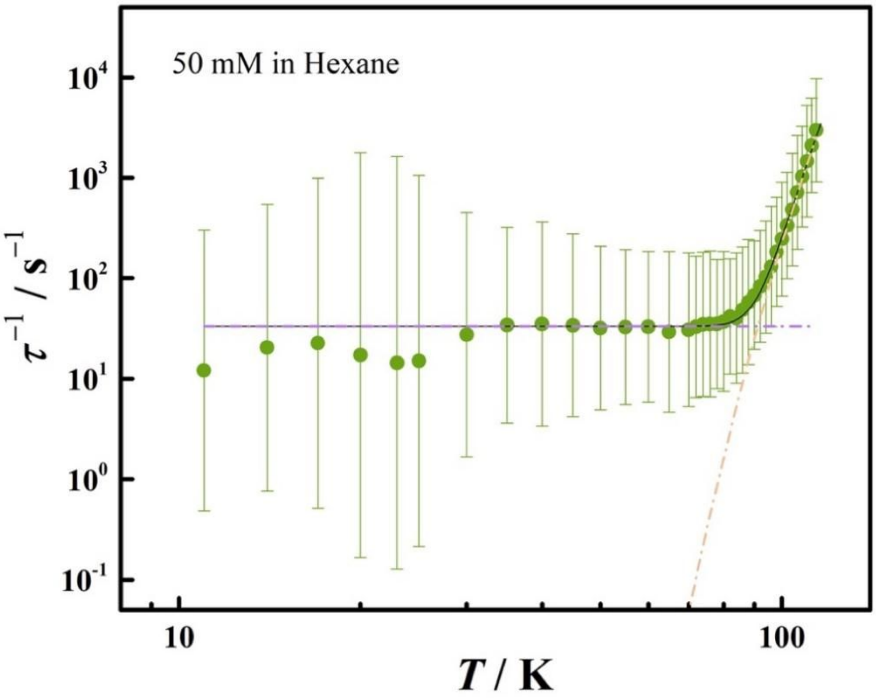

Figure S136 Plot of natural log of the inverse relaxation time *vs*. temperature for a 50 mM solution of **3-Dy** in hexane under zero dc magnetic field (green points are from ac data). The dashed orange line shows $\tau^{-1} = \tau_0{}^{-1}e^{-U_{eff}/T}$, where U_{eff} = 1964(fixed) K and τ_0 = $10^{-10.919 \pm 0.038}$ (1.21×10^{-11}) s, the dashed purple line shows $\tau^{-1} = \tau^{-1}{}_{QTM}$, where τ_{QTM} = $10^{-1.444 \pm 0.025}$ (0.036) s. Black solid line shows their sum with $\tau^{-1} = \tau_0{}^{-1}e^{-U_{eff}/T} + \tau^{-1}{}_{QTM}$.

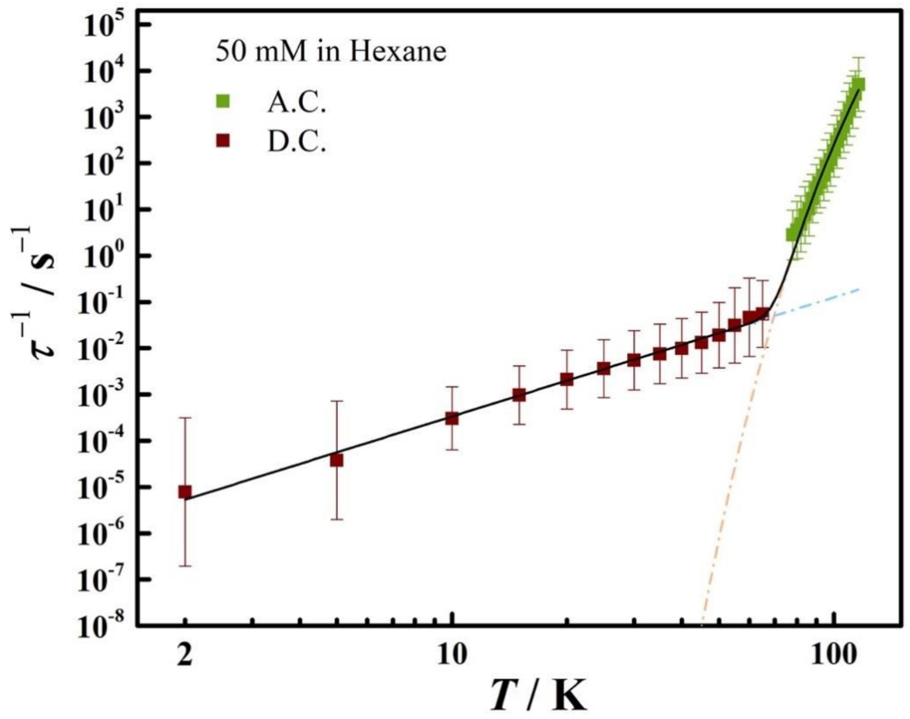

Figure S137 Plot of natural log of the inverse relaxation time *vs*. temperature for a 50 mM solution of **3-Dy** in hexane under 1000 Oe dc magnetic field (pink points are from ac data; brown points are from dc data). The dashed orange line shows $\tau^{-1} = \tau_0{}^{-1}e^{-U_{eff}/T}$, where U_{eff} = 1964(fixed) K and τ_0 = $10^{-10.935 \pm 0.029}$ (1.16×10^{-11}) s, the dashed blue line shows $\tau^{-1} = CT^n$, where $C = 10^{-6.049 \pm 0.116}$ (8.93×10^{-7}) s^{-1} K^{-n} and n = 2.575 ± 0.081. Black solid line shows their sum with $\tau^{-1} = \tau_0{}^{-1}e^{-U_{eff}/T} + CT^n$.

6.5 Field-swept magnetic measurements

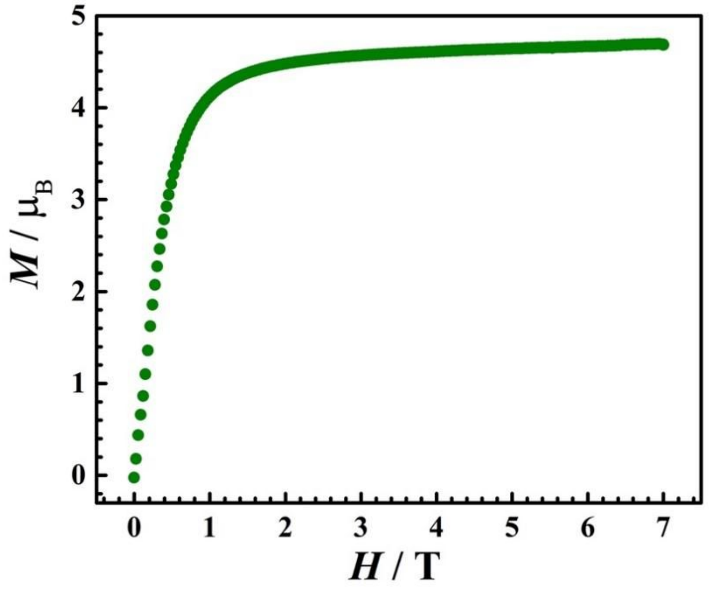

Figure S138 Field dependent magnetization at 2.0 K up to 7 Tesla for **1-Tb**.

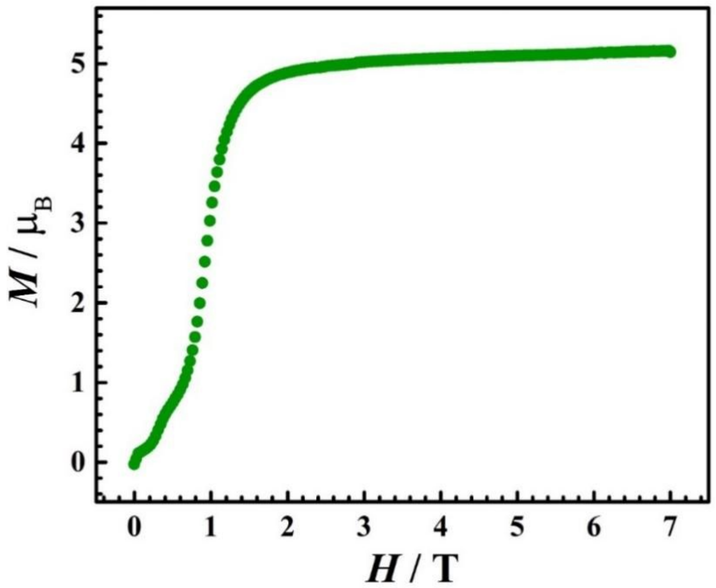

Figure S139 Field dependent magnetization at 2.0 K up to 7 Tesla for **1-Dy**.

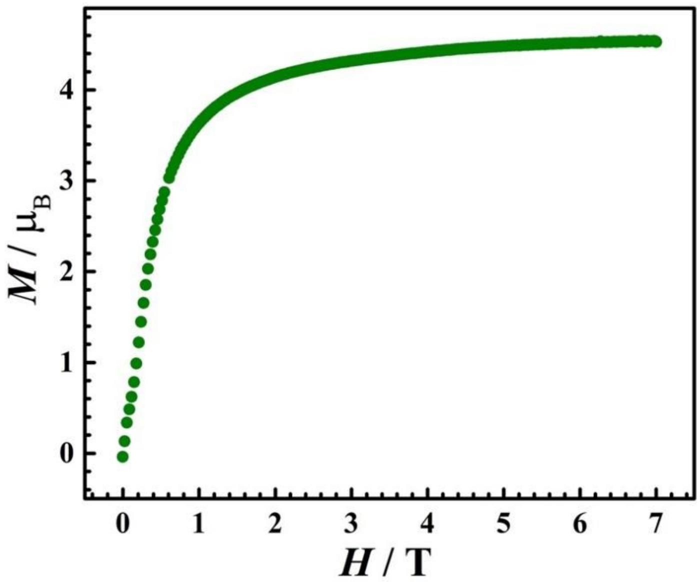

Figure S140 Field dependent magnetization at 2.0 K up to 7 Tesla for **2-Tb**.

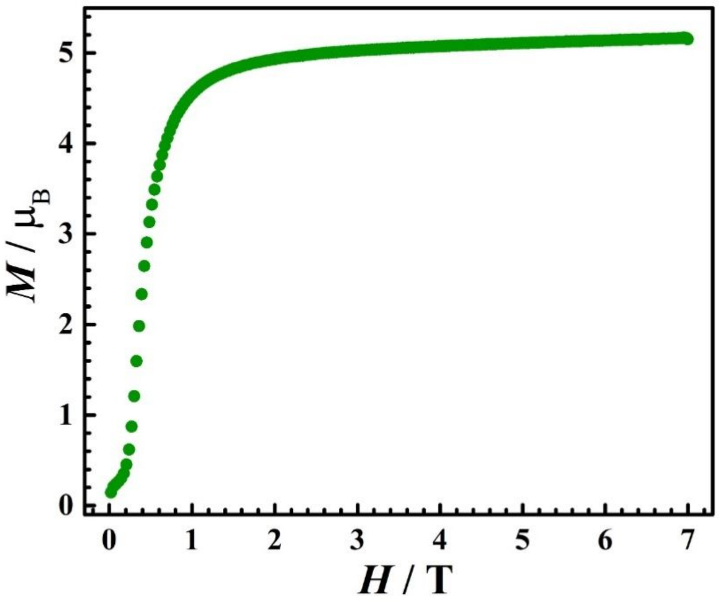

Figure S141 Field dependent magnetization at 2.0 K up to 7 Tesla for **2-Dy.**

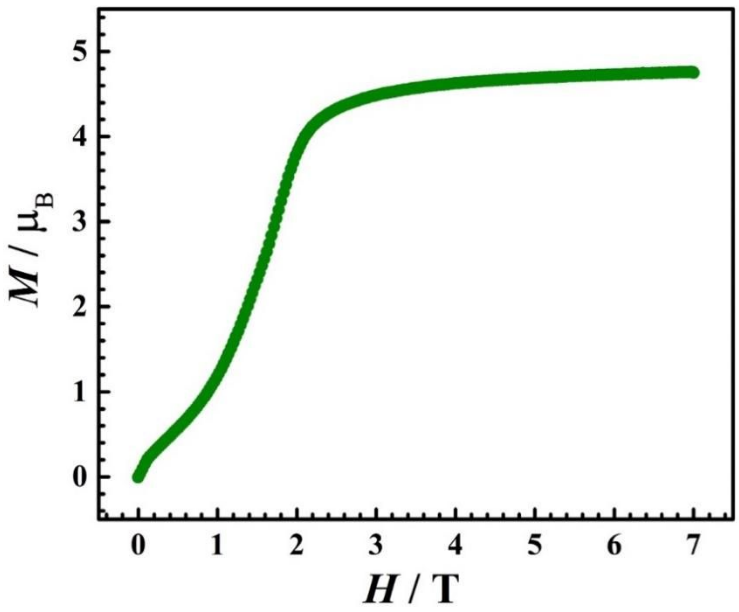

Figure S142 Field dependent magnetization at 2.0 K up to 7 Tesla for **3-Tb.**

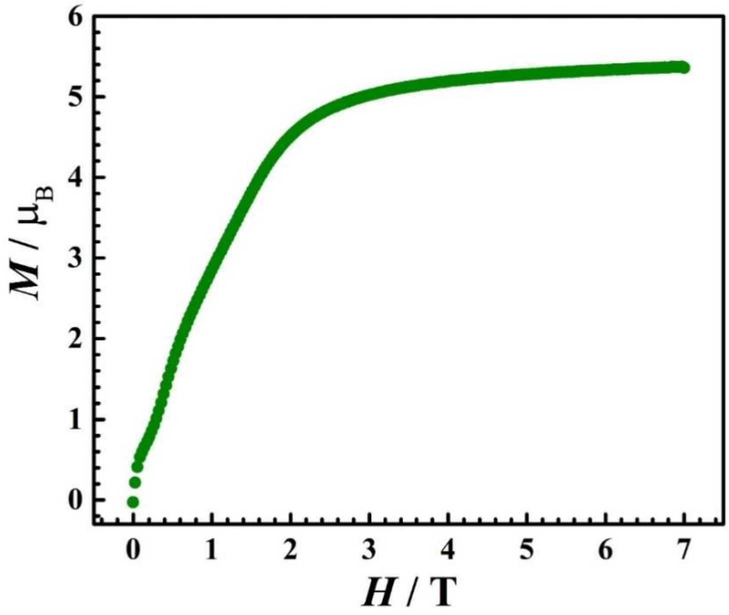

Figure S143 Field dependent magnetization at 2.0 K up to 7 Tesla for **3-Dy.**

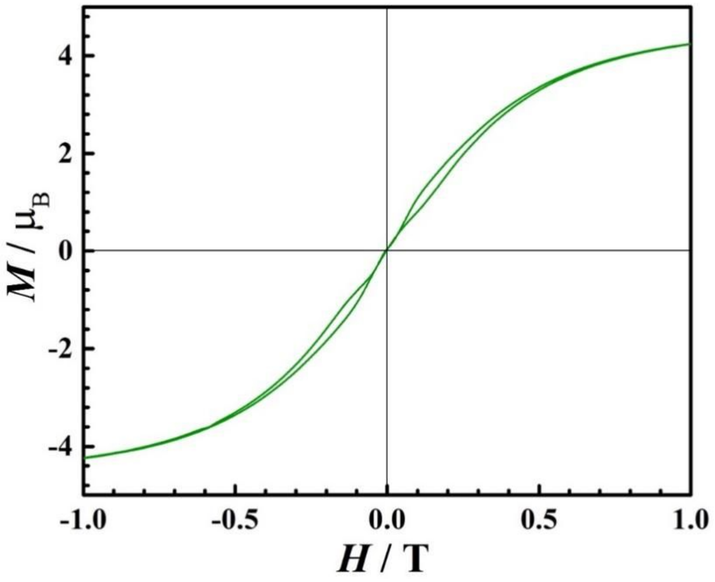

Figure S144 Magnetic hysteresis loop measurements for **1-Tb** at an average sweep rate of 22 Oe/s at 2 K.

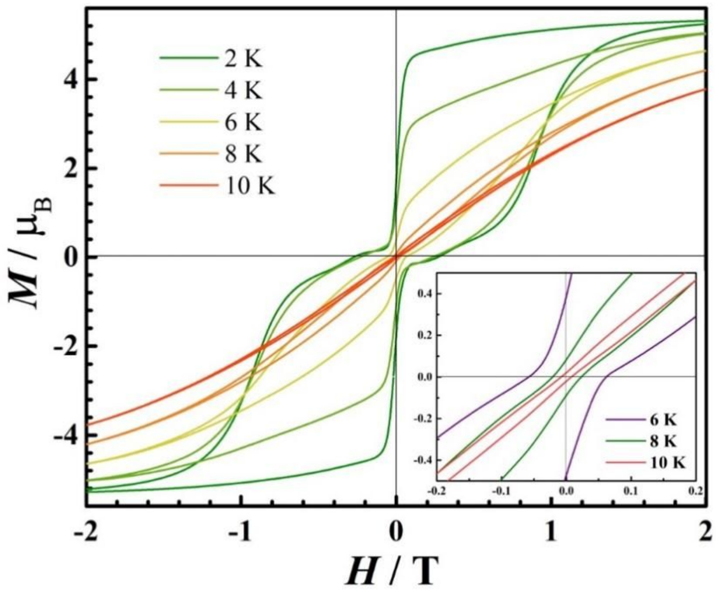

Figure S145 Magnetic hysteresis loop measurements for **1-Dy** at an average sweep rate of 22 Oe/s in a temperature range of 2 K to 10 K.

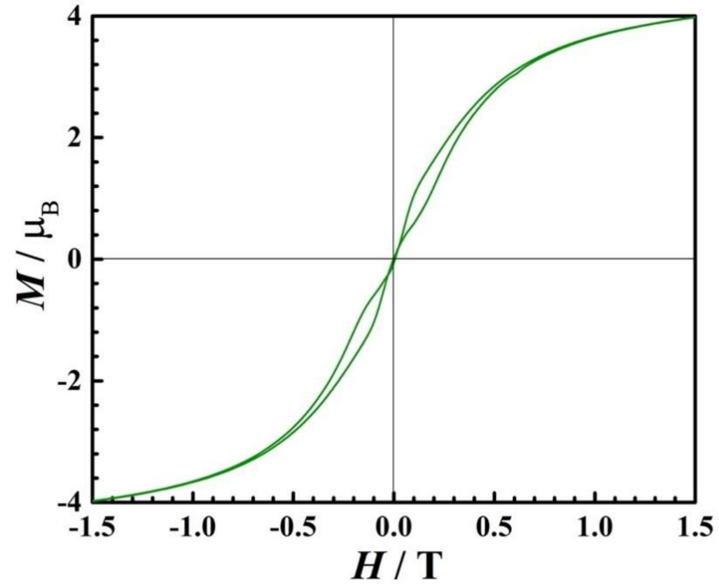

Figure S146 Magnetic hysteresis loop measurements for **2-Tb** at an average sweep rate of 22 Oe/s at 2 K.

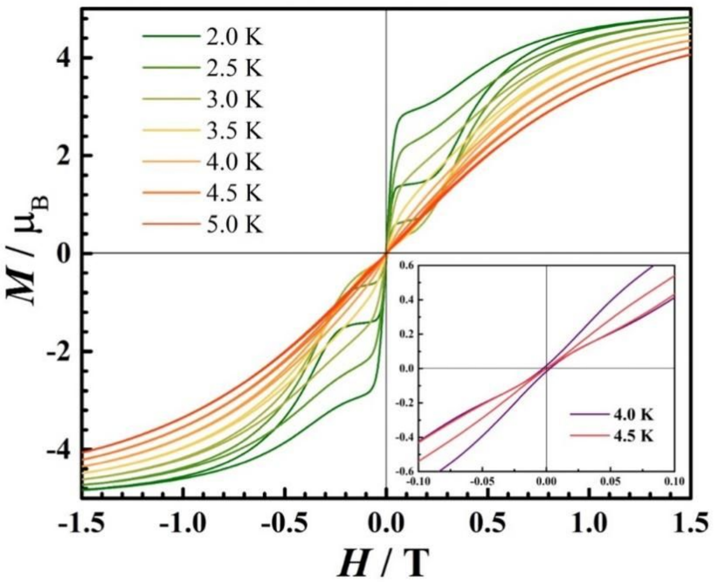

Figure S147 Magnetic hysteresis loop measurements for **2-Dy** at an average sweep rate of 22 Oe/s in a temperature range of 2 K to 5 K.

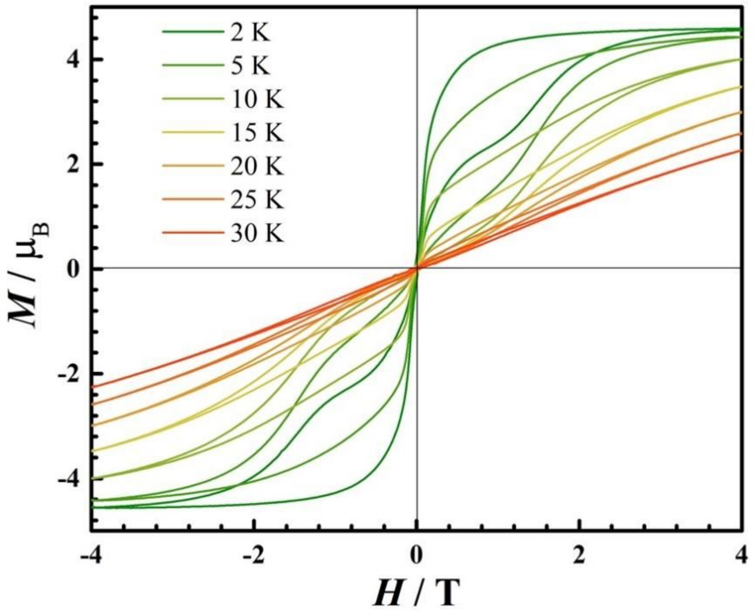

Figure S148 Magnetic hysteresis loop measurements for a 50 mM solution of **3-Tb** in hexane at an average sweep rate of 22 Oe/s in a temperature range of 2 K to 30 K.

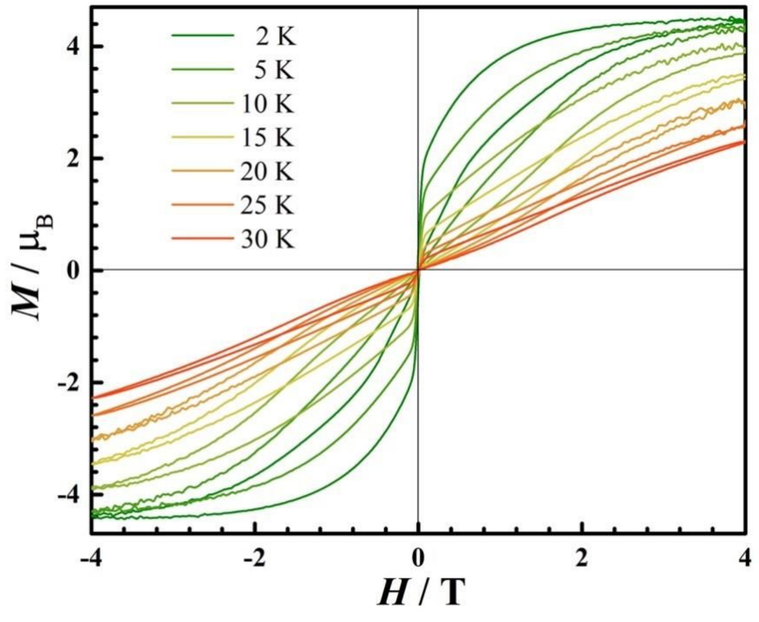

Figure S149 Magnetic hysteresis loop measurements for a 50 mM solution of **3-Dy** in hexane at an average sweep rate of 22 Oe/s in a temperature range of 2 K to 30 K.

7. EPR spectroscopy

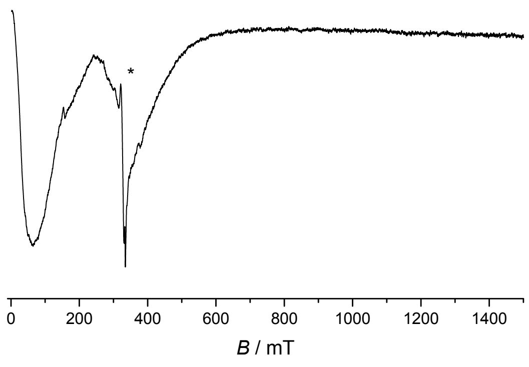

Figure S150 X–band (9.38 GHz) cw EPR spectrum of polycrystalline **2-Tb** at 5 K. *marks an impurity signal.

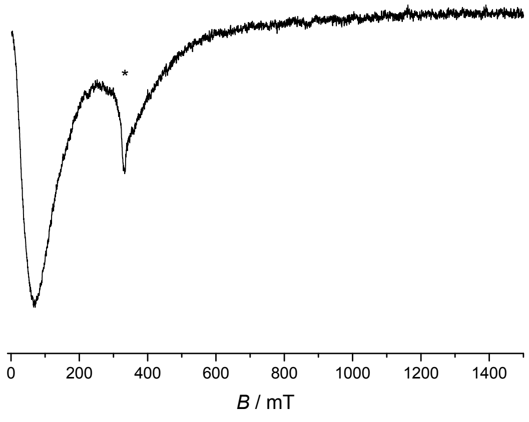

Figure S151 X–band (9.37 GHz) cw EPR spectrum of polycrystalline **3-Tb** at 5 K. *marks an impurity signal.

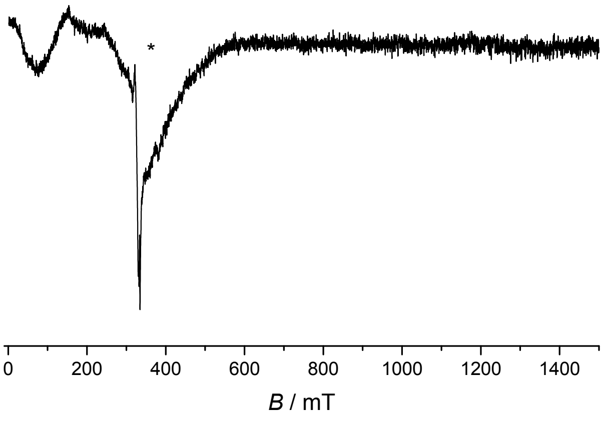

Figure S152 X–band (9.38 GHz) cw EPR spectrum of polycrystalline **2-Dy** recorded at 5 K with the acquisition parameters of modulation amplitude 5 G and microwave power 2.158 mW.

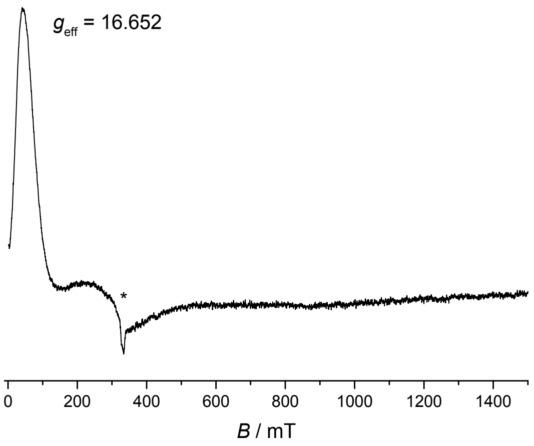

Figure S153 X–band (9.38 GHz) cw EPR spectrum of polycrystalline **3-Dy** at 5 K (modulation amplitude 5 G; microwave power 7.0 mW). *marks an impurity signal.

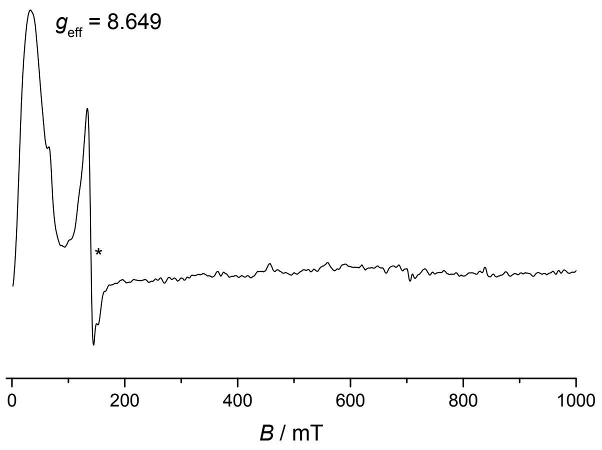

Figure S154 S–band (3.88 GHz) cw EPR spectrum of polycrystalline **3-Dy** at 5 K. *marks an impurity signal.

8. CASSCF calculations

Table S31 CASSCF-SO-calculated electronic states for **1-Dy**. Crystal field wavefunction calculated including 0.1 T field along the main anisotropy axis, contributions ≥ 10% shown, rounded to nearest percent.

**Energy (cm^{-1})**	**Energy (K)**	g_x	g_y	g_z	**g_z Angle (°)**	**Wavefunction**
0	0	0.00	0.00	19.88	-	100%|±15/2>
233	335	0.00	0.00	17.37	13.0	91%|±13/2>
446	642	0.00	0.00	14.55	3.8	88%|±11/2>
589	847	0.22	0.40	13.65	32.2	87%|±9/2>
691	994	2.08	2.71	9.39	35.2	58%|±7/2> + 27%|±5/2>
803	1155	4.22	4.83	7.35	81.6	37%|±3/2> + 25%|±5/2> + 17%|±7/2> + 12%|∓1/2>
945	1360	1.07	2.47	13.41	77.1	29%|±5/2> + 18%|∓1/2> + 15%|±3/2> + 15%|±1/2> + 13%|±7/2>
1205	1734	0.07	0.18	18.67	83.0	34%|±1/2> + 33%|±3/2> + 14%|∓1/2> + 13%|±5/2>

Table S32 CASSCF-SO-calculated electronic states for **1-Tb**. Crystal field wavefunction calculated including 0.1 T field along the main anisotropy axis, contributions ≥ 10% shown, rounded to nearest percent.

<table>
<tr><th>Energy (cm^{-1})</th><th>Energy (K)</th><th>g_z</th><th>g_z Angle (°)</th><th>Δ_{tun} (cm^{-1})</th><th>Wavefunction</th></tr>
<tr><td>0</td><td>0</td><td rowspan="2">17.89</td><td rowspan="2">-</td><td rowspan="2">0.0002</td><td>100%|–6></td></tr>
<tr><td>0</td><td>0</td><td>100%|+6></td></tr>
<tr><td>313</td><td>450</td><td rowspan="2">14.44</td><td rowspan="2">1.8</td><td rowspan="2">0.05</td><td>97%|–5></td></tr>
<tr><td>313</td><td>450</td><td>97%|+5></td></tr>
<tr><td>602</td><td>866</td><td rowspan="2">11.11</td><td rowspan="2">6.7</td><td rowspan="2">1.4</td><td>63%|–4> + 30%|+4></td></tr>
<tr><td>603</td><td>868</td><td>63%|+4> + 30%|–4></td></tr>
<tr><td>839</td><td>1207</td><td>-</td><td>-</td><td>-</td><td>43%|–3> + 40%|+3></td></tr>
<tr><td>852</td><td>1226</td><td>-</td><td>-</td><td>-</td><td>45%|+3> + 42%|–3></td></tr>
<tr><td>1003</td><td>1443</td><td>-</td><td>-</td><td>-</td><td>29%|–2> + 29%|+2> + 16%|–1> + 16%|+1></td></tr>
<tr><td>1039</td><td>1495</td><td>-</td><td>-</td><td>-</td><td>40%|–2> + 40%|+2></td></tr>
<tr><td>1108</td><td>1594</td><td>-</td><td>-</td><td>-</td><td>44%|0> + 18%|–1> + 18%|+1></td></tr>
<tr><td>1148</td><td>1652</td><td>-</td><td>-</td><td>-</td><td>41%|–1> + 41%|+1></td></tr>
<tr><td>1172</td><td>1686</td><td>-</td><td>-</td><td>-</td><td>48%|0> + 13%|–1> + 13%|+1> + 13%|+2> + 12%|–2></td></tr>
</table>

Table S33 CASSCF-SO-calculated electronic states for **2-Dy**. Crystal field wavefunction calculated including 0.1 T field along the main anisotropy axis, contributions ≥ 10% shown, rounded to nearest percent.

**Energy (cm^{-1})**	**Energy (K)**	g_x	g_y	g_z	**g_z Angle (°)**	**Wavefunction**
0	0	0.00	0.00	19.82	-	99%|±15/2>
269	387	0.00	0.00	17.05	9.4	93%|±13/2>
572	822	0.02	0.03	14.49	19.6	82%|±11/2> + 11%|±7/2>
770	1108	0.00	0.05	12.77	36.5	51%|±9/2> + 21%|±5/2> + 11%|±7/2>
987	1420	0.16	0.19	9.87	33.4	38%|±9/2> + 24%|±3/2> + 19%|±7/2>
1185	1704	2.97	3.49	6.85	42.2	46%|±7/2> + 33%|±1/2>
1363	1961	2.07	4.35	9.78	87.6	43%|±5/2> + 17%|∓3/2> + 15%|∓1/2>
1487	2139	0.92	3.81	16.81	88.0	37%|±3/2> + 33%|∓1/2>

Table S34 CASSCF-SO-calculated electronic states for **2-Tb**. Crystal field wavefunction calculated including 0.1 T field along the main anisotropy axis, contributions ≥ 10% shown, rounded to nearest percent.

**Energy (cm^{-1})**	**Energy (K)**	g_z	g_z **Angle (°)**	Δ_{tun} **(cm^{-1})**	**Wavefunction**
0	0	17.94	-	0.0001	100%|–6>
0	0				100%|+6>
391	563	14.50	1.8	0.009	100%|–5>
391	563				100%|+5>
752	1082	11.25	8.2	0.5	82%|–4> + 16%|+4>
752	1082				82%|+4> + 16%|–4>
1018	1465	-	-	-	39%|–3> + 38%|+3>
1038	1493	-	-	-	43%|+3> + 42%|–3>
1147	1650	-	-	-	32%|0> + 29%|–2> + 29%|+2>
1287	1852	-	-	-	25%|–1> + 25%|+1> + 14%|–2> + 14%|+2> + 11%|–3> + 11%|+3>
1332	1917	-	-	-	33%|–2> + 33%|+2> + 16%|–1> + 16%|+1>
1657	2384	-	-	-	68%|0> + 15%|–2> + 15%|+2>
1672	2405	-	-	-	47%|–1> + 47%|+1>

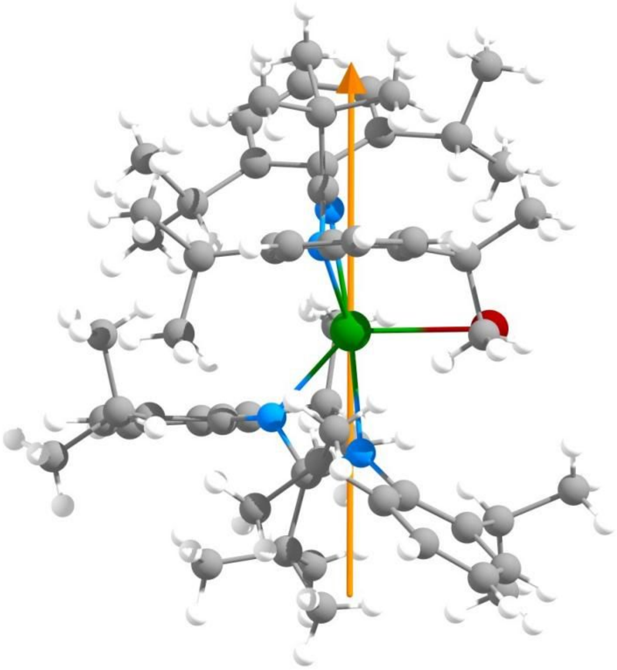

Figure S155 Principal anisotropy axis of the ground doublet (orange arrow) for **1-Dy**, calculated with CASSCF-SO.

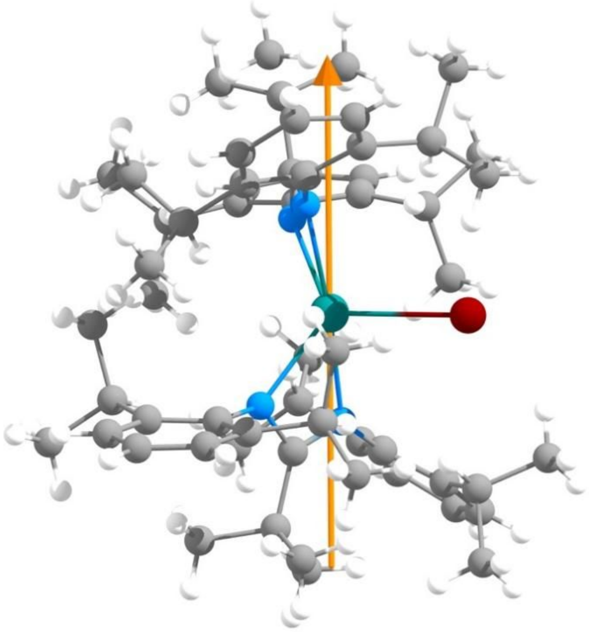

Figure S156 Principal anisotropy axis of the ground pseudo-doublet (orange arrow) for **1-Tb**, calculated with CASSCF-SO.

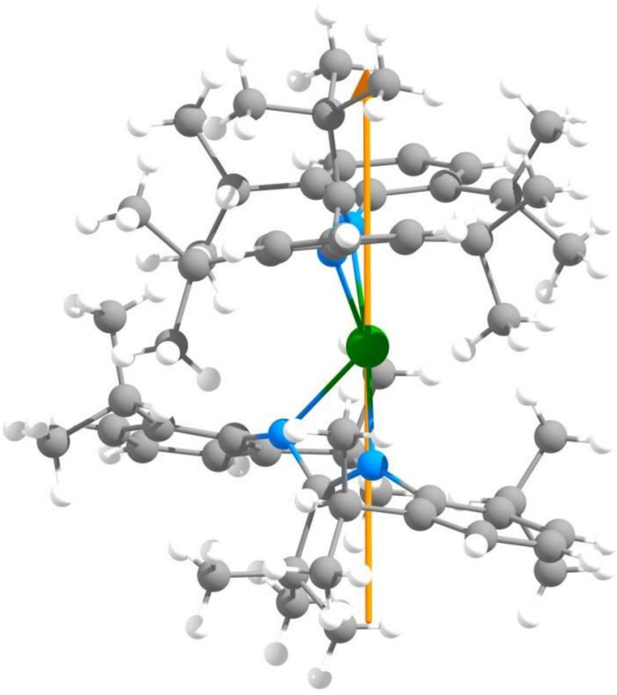

Figure S157 Principal anisotropy axis of the ground doublet (orange arrow) for **2-Dy**, calculated with CASSCF-SO.

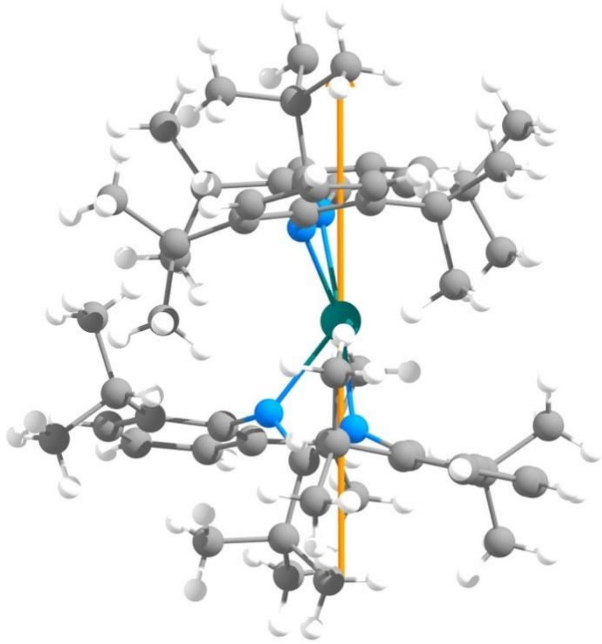

Figure S158 Principal anisotropy axis of the ground pseudo-doublet (orange arrow) for **2-Tb**, calculated with CASSCF-SO.

Table S35 Projected CF splitting, SOC and f-d coupling (parameters > 220 cm[-1], except for $\hat{R}_\alpha\hat{S}_\alpha\hat{O}_8^{-2}$ which are shown for comparison to Table S37) parameters for **3-Dy**, from CASSCF-SO calculations. The quantization axis is the principal anisotropy axis of the ground pseudo-doublet.

Parameter	Value (cm⁻¹)	Parameter	Value (cm⁻¹)	Effective Operator
λ	−396	$J^{RS}_{+1,1,+1}$	3276	$\hat{R}_x\hat{S}_x$
B_2^{-2}	39	$J^{RS}_{-1,1,-1}$	3276	$\hat{R}_y\hat{S}_y$
B_2^{-1}	−16	$J^{RS}_{0,1,0}$	3276	$\hat{R}_z\hat{S}_z$
B_2^{0}	1682	$J^{RSL}_{+1,1,+1,4,-2}$	−694	$\hat{R}_x\hat{S}_x\hat{O}_4^{-2}$
B_2^{+1}	−21	$J^{RSL}_{-1,1,-1,4,-2}$	−694	$\hat{R}_y\hat{S}_y\hat{O}_4^{-2}$
B_2^{+2}	57	$J^{RSL}_{0,1,0,4,-2}$	−694	$\hat{R}_z\hat{S}_z\hat{O}_4^{-2}$
B_4^{-4}	17	$J^{RSL}_{+1,1,+1,2,-2}$	616	$\hat{R}_x\hat{S}_x\hat{O}_2^{-2}$
B_4^{-3}	−99	$J^{RSL}_{-1,1,-1,2,-2}$	616	$\hat{R}_y\hat{S}_y\hat{O}_2^{-2}$
B_4^{-2}	−675	$J^{RSL}_{0,1,0,2,-2}$	616	$\hat{R}_z\hat{S}_z\hat{O}_2^{-2}$
B_4^{-1}	117	$J^{RSL}_{+1,1,+1,4,4}$	−614	$\hat{R}_x\hat{S}_x\hat{O}_4^{4}$
B_4^{0}	−55	$J^{RSL}_{-1,1,-1,4,4}$	−614	$\hat{R}_y\hat{S}_y\hat{O}_4^{4}$
B_4^{+1}	−78	$J^{RSL}_{0,1,0,4,4}$	−614	$\hat{R}_z\hat{S}_z\hat{O}_4^{4}$
B_4^{+2}	−39	$J^{RSL}_{+1,2,+1,5,-2}$	−612	$\hat{R}_x\hat{O}_2^{+1}\hat{O}_5^{-2}$
B_4^{+3}	−208	$J^{RSL}_{-1,2,-1,5,-2}$	−612	$\hat{R}_y\hat{O}_2^{-1}\hat{O}_5^{-2}$
B_4^{+4}	−117	$J^{RSL}_{+1,1,+1,8,-2}$	−1	$\hat{R}_x\hat{S}_x\hat{O}_8^{-2}$
B_6^{-6}	−11	$J^{RSL}_{-1,1,-1,8,-2}$	−1	$\hat{R}_y\hat{S}_y\hat{O}_8^{-2}$
B_6^{-5}	6	$J^{RSL}_{0,1,0,8,-2}$	−1	$\hat{R}_z\hat{S}_z\hat{O}_8^{-2}$
B_6^{-4}	3			
B_6^{-3}	−57			
B_6^{-2}	−403			
B_6^{-1}	84			
B_6^{0}	−60			
B_6^{+1}	−48			
B_6^{+2}	−15			
B_6^{+3}	−112			
B_6^{+4}	−26			
B_6^{+5}	−2			

B_6^{+6}	−3			

Table S36 Projected CF splitting, SOC and f-d coupling (parameters > 230 cm^{-1}, except for $\hat{R}_\alpha \hat{S}_\alpha \hat{O}_2^{-2}$ which are shown for comparison to Table S36) parameters for **3-Dy**, from CASPT2-SO calculations. The quantization axis is the principal anisotropy axis of the ground doublet from CASSCF-SO calculations.

Parameter	Value (cm^{-1})	Parameter	Value (cm^{-1})	Effective Operator	CASPT2/CASSCF Ratio
λ	−396	$J^{RS}_{+1,1,+1}$	2193	$\hat{R}_x\hat{S}_x$	0.67
B_2^{-2}	89	$J^{RS}_{-1,1,-1}$	2193	$\hat{R}_y\hat{S}_y$	0.67
B_2^{-1}	39	$J^{RS}_{0,1,0}$	2193	$\hat{R}_z\hat{S}_z$	0.67
B_2^{0}	1695	$J^{RSL}_{+1,2,+1,5,-2}$	−612	$\hat{R}_x\hat{O}_2^{+1}\hat{O}_5^{-2}$	1.00
B_2^{+1}	−75	$J^{RSL}_{-1,2,-1,5,-2}$	−612	$\hat{R}_y\hat{O}_2^{-1}\hat{O}_5^{-2}$	1.00
B_2^{+2}	72	$J^{RSL}_{+1,1,+1,4,-2}$	−557	$\hat{R}_x\hat{S}_x\hat{O}_4^{-2}$	0.80
B_4^{-4}	−1	$J^{RSL}_{-1,1,-1,4,-2}$	−557	$\hat{R}_y\hat{S}_y\hat{O}_4^{-2}$	0.80
B_4^{-3}	−11	$J^{RSL}_{0,1,0,4,-2}$	−557	$\hat{R}_z\hat{S}_z\hat{O}_4^{-2}$	0.80
B_4^{-2}	−988	$J^{RSL}_{+1,1,+1,4,4}$	−415	$\hat{R}_x\hat{S}_x\hat{O}_4^{4}$	0.68
B_4^{-1}	128	$J^{RSL}_{-1,1,-1,4,4}$	−415	$\hat{R}_y\hat{S}_y\hat{O}_4^{4}$	0.68
B_4^{0}	60	$J^{RSL}_{0,1,0,4,4}$	−415	$\hat{R}_z\hat{S}_z\hat{O}_4^{4}$	0.68
B_4^{+1}	−57	$J^{RSL}_{+1,1,+1,8,-2}$	−303	$\hat{R}_x\hat{S}_x\hat{O}_8^{-2}$	303
B_4^{+2}	−45	$J^{RSL}_{-1,1,-1,8,-2}$	−303	$\hat{R}_y\hat{S}_y\hat{O}_8^{-2}$	303
B_4^{+3}	−115	$J^{RSL}_{0,1,0,8,-2}$	−303	$\hat{R}_z\hat{S}_z\hat{O}_8^{-2}$	303
B_4^{+4}	78	$J^{RSL}_{+1,1,+1,2,-2}$	98	$\hat{R}_x\hat{S}_x\hat{O}_2^{-2}$	0.16
B_6^{-6}	140	$J^{RSL}_{-1,1,-1,2,-2}$	98	$\hat{R}_y\hat{S}_y\hat{O}_2^{-2}$	0.16
B_6^{-5}	−58	$J^{RSL}_{0,1,0,2,-2}$	98	$\hat{R}_z\hat{S}_z\hat{O}_2^{-2}$	0.16
B_6^{-4}	−1				
B_6^{-3}	−61				
B_6^{-2}	−552				
B_6^{-1}	79				
B_6^{0}	−95				
B_6^{+1}	−26				
B_6^{+2}	−23				
B_6^{+3}	−125				

B_6^{+4}	1				
B_6^{+5}	14				
B_6^{+6}	54				

Table S37 Projected CF splitting, SOC and f-d coupling (parameters > 180 cm^{-1} parameters for **3-Tb**, from CASSCF-SO calculations. The quantization axis is the principal anisotropy axis of the ground doublet.

**Parameter**	**Value (cm^{-1})**	**Parameter**	**Value (cm^{-1})**	**Effective Operator**
λ	−297	$J^{RS}_{+1,1,+1}$	4001	$\hat{R}_x\hat{S}_x$
B_2^{-2}	−41	$J^{RS}_{-1,1,-1}$	4001	$\hat{R}_y\hat{S}_y$
B_2^{-1}	−121	$J^{RS}_{0,1,0}$	4001	$\hat{R}_z\hat{S}_z$
B_2^0	1671	$J^{RSL}_{+1,1,+1,2,-2}$	548	$\hat{R}_x\hat{S}_x\hat{O}_2^{-2}$
B_2^{+1}	−42	$J^{RSL}_{-1,1,-1,2,-2}$	548	$\hat{R}_y\hat{S}_y\hat{O}_2^{-2}$
B_2^{+2}	31	$J^{RSL}_{0,1,0,2,-2}$	548	$\hat{R}_z\hat{S}_z\hat{O}_2^{-2}$
B_4^{-4}	34	$J^{RSL}_{+1,1,+1,4,2}$	492	$\hat{R}_x\hat{S}_x\hat{O}_4^2$
B_4^{-3}	−167	$J^{RSL}_{-1,1,-1,4,2}$	492	$\hat{R}_y\hat{S}_y\hat{O}_4^2$
B_4^{-2}	−111	$J^{RSL}_{0,1,0,4,2}$	492	$\hat{R}_z\hat{S}_z\hat{O}_4^2$
B_4^{-1}	−92	$J^{RSL}_{+1,1,+1,4,4}$	−446	$\hat{R}_x\hat{S}_x\hat{O}_4^4$
B_4^0	−65	$J^{RSL}_{-1,1,-1,4,4}$	−446	$\hat{R}_y\hat{S}_y\hat{O}_4^4$
B_4^{+1}	−27	$J^{RSL}_{0,1,0,4,4}$	−446	$\hat{R}_z\hat{S}_z\hat{O}_4^4$
B_4^{+2}	−721	$J^{RSL}_{+1,2,+1,3,2}$	280	$\hat{R}_x\hat{O}_2^{+1}\hat{O}_3^2$
B_4^{+3}	23	$J^{RSL}_{-1,2,-1,3,2}$	280	$\hat{R}_y\hat{O}_2^{-1}\hat{O}_3^2$
B_4^{+4}	122			
B_6^{-6}	5			
B_6^{-5}	1			
B_6^{-4}	12			
B_6^{-3}	−95			
B_6^{-2}	−68			
B_6^{-1}	−32			
B_6^0	−67			
B_6^{+1}	−15			
B_6^{+2}	−451			
B_6^{+3}	10			

B_6^{+4}	31			
B_6^{+5}	0			
B_6^{+6}	12			

Table S38 Projected CF splitting, SOC and f-d coupling (parameters > 180 cm-1, except for $\hat{R}_\alpha \hat{S}_\alpha \hat{O}_2^{-2}$ which are shown for comparison to Table S37) parameters for **3-Tb**, from CASPT2-SO calculations. The quantization axis is the principal anisotropy axis of the ground doublet from CASSCF-SO calculations.

**Parameter**	**Value (cm^{-1})**	**Parameter**	**Value (cm^{-1})**	**Effective Operator**	**CASPT2/CASSCF Ratio**
λ	−297	$J^{RS}_{+1,1,+1}$	2572	$\hat{R}_x\hat{S}_x$	0.64
B_2^{-2}	−30	$J^{RS}_{-1,1,-1}$	2572	$\hat{R}_y\hat{S}_y$	0.64
B_2^{-1}	−133	$J^{RS}_{0,1,0}$	2572	$\hat{R}_z\hat{S}_z$	0.64
B_2^0	1739	$J^{RSL}_{+1,1,+1,4,2}$	356	$\hat{R}_x\hat{S}_x\hat{O}_4^2$	0.82
B_2^{+1}	−44	$J^{RSL}_{-1,1,-1,4,2}$	356	$\hat{R}_y\hat{S}_y\hat{O}_4^2$	0.82
B_2^{+2}	143	$J^{RSL}_{0,1,0,4,2}$	356	$\hat{R}_z\hat{S}_z\hat{O}_4^2$	0.82
B_4^{-4}	39	$J^{RSL}_{+1,1,+1,4,4}$	−366	$\hat{R}_x\hat{S}_x\hat{O}_4^4$	0.72
B_4^{-3}	−182	$J^{RSL}_{-1,1,-1,4,4}$	−366	$\hat{R}_y\hat{S}_y\hat{O}_4^4$	0.72
B_4^{-2}	−141	$J^{RSL}_{0,1,0,4,4}$	−366	$\hat{R}_z\hat{S}_z\hat{O}_4^4$	0.72
B_4^{-1}	−102	$J^{RSL}_{+1,2,+1,3,2}$	280	$\hat{R}_x\hat{O}_2^{+1}\hat{O}_3^2$	1.00
B_4^0	−92	$J^{RSL}_{-1,2,-1,3,2}$	280	$\hat{R}_y\hat{O}_2^{-1}\hat{O}_3^2$	1.00
B_4^{+1}	−49	$J^{RSL}_{+1,1,+1,2,-2}$	20	$\hat{R}_x\hat{S}_x\hat{O}_2^{-2}$	0.04
B_4^{+2}	−928	$J^{RSL}_{-1,1,-1,2,-2}$	20	$\hat{R}_y\hat{S}_y\hat{O}_2^{-2}$	0.04
B_4^{+3}	32	$J^{RSL}_{0,1,0,2,-2}$	20	$\hat{R}_z\hat{S}_z\hat{O}_2^{-2}$	0.04
B_4^{+4}	158				
B_6^{-6}	14				
B_6^{-5}	12				
B_6^{-4}	36				
B_6^{-3}	−99				
B_6^{-2}	−98				
B_6^{-1}	−43				
B_6^0	−81				
B_6^{+1}	−28				

B_6^{+2}	−595				
B_6^{+3}	9				
B_6^{+4}	112				
B_6^{+5}	−43				
B_6^{+6}	20				

Table S39 Electronic states for **3-Tb** obtained from a model Hamiltonian (Equation 1, main text) using projected parameters from a CASSCF-SO calculation (Table S37), and including 0.01 T field along the main anisotropy axis. Crystal field wavefunction calculated also including 0.01 T field along the main anisotropy axis, contributions ≥ 10% shown, rounded to nearest percent.

<table>
<tr><th>Ab initio Energy (cm $^{-1}$)</th><th>Wavefunction in $|S_{tot}, J_{tot}, m_J\rangle$ basis</th><th>Wavefunction in $|J_{4f}, J_{tot}, m_J\rangle$ basis</th></tr>
<tr><td>0</td><td>98%|7/2, 13/2, ±13/2></td><td>98%|6, 13/2, ±13/2></td></tr>
<tr><td>330</td><td>89%|7/2, 13/2, ±11/2></td><td>89%|6, 13/2, ±11/2></td></tr>
<tr><td>682</td><td>79%|7/2, 13/2, ±9/2> + 14%|7/2, 11/2, ±9/2></td><td>79%|6, 13/2, ±9/2> + 13%|5, 11/2, ±9/2></td></tr>
<tr><td>1044</td><td>71%|7/2, 13/2, ±7/2> + 17%|7/2, 11/2, ±7/2></td><td>71%|6, 13/2, ±7/2> + 15%|5, 11/2, ±7/2></td></tr>
<tr><td>1400</td><td>68%|7/2, 13/2, ±5/2> + 14%|7/2, 11/2, ±5/2></td><td>68%|6, 13/2, ±5/2> + 12%|5, 11/2, ±5/2></td></tr>
<tr><td>1714</td><td>65%|7/2, 13/2, ±3/2> + 18%|7/2, 13/2, ±1/2></td><td>65%|6, 13/2, ±3/2> + 18%|6, 13/2, ∓1/2></td></tr>
<tr><td>1899</td><td>69%|7/2, 13/2, ±1/2> + 13%|7/2, 13/2, ±3/2> + 10%|7/2, 13/2, ±5/2></td><td>69%|6, 13/2, ±1/2> + 13%|6, 13/2, ∓3/2> + 10%|6, 13/2, ±5/2></td></tr>
<tr><td>2317</td><td>87% |7/2, 11/2, ±11/2></td><td>73%|5, 11/2, ±11/2> + 17%|6, 11/2, ±11/2></td></tr>
<tr><td>2566</td><td>60%|7/2, 11/2, ±9/2> + 14%|7/2, 9/2, ±9/2> + 13%|7/2, 13/2, ±9/2></td><td>51%|5, 11/2, ±9/2> + 13%|6, 13/2, ±9/2> + 11%|6, 11/2, ±9/2> + 11%|4, 9/2, ±9/2></td></tr>
<tr><td>2693</td><td>29%|7/2, 11/2, ±7/2> + 28%|7/2, 11/2, ±3/2> + 13%|7/2, 11/2, ∓1/2></td><td>25%|5, 11/2, ±7/2> + 24%|5, 11/2, ±3/2> + 11%|5, 11/2, ∓1/2></td></tr>
<tr><td>2844</td><td>29%|7/2, 11/2, ±7/2> + 20%|7/2, 11/2, ±3/2> + 17%|7/2, 11/2, ∓1/2></td><td>25%|5, 11/2, ±7/2> + 17%|5, 11/2, ±3/2> + 14%|5, 11/2, ∓1/2></td></tr>
<tr><td>2864</td><td>56%|7/2, 11/2, ±5/2> + 15%|7/2, 13/2, ±5/2></td><td>49%|5, 11/2, ±5/2> + 15%|6, 13/2, ±5/2></td></tr>
<tr><td>3177</td><td>55%|7/2, 11/2, ±1/2> + 27%|7/2, 11/2, ∓3/2></td><td>47%|5, 11/2, ±1/2> + 23%|5, 11/2, ∓3/2></td></tr>
</table>

Table S40 Electronic states for **3-Tb** obtained from a model Hamiltonian (Equation 1, main text) using projected parameters from a CASPT2-SO calculation (Table S38), and including 0.01 T field along the main anisotropy axis. Crystal field wavefunction calculated also including 0.01 T field along the main anisotropy axis, contributions ≥ 10% shown, rounded to nearest percent.

<table>
<tr><th>Ab initio Energy (cm $^{-1}$)</th><th>Wavefunction in $|S_{tot}, J_{tot}, m_J\rangle$ basis</th><th>Wavefunction in $|J_{4f}, J_{tot}, m_J\rangle$ basis</th></tr>
<tr><td>0</td><td>99%|7/2, 13/2, ±13/2></td><td>99%|6, 13/2, ±13/2></td></tr>
<tr><td>353</td><td>88%|7/2, 13/2, ±11/2></td><td>88%|6, 13/2, ±11/2></td></tr>
<tr><td>723</td><td>78%|7/2, 13/2, ±9/2> + 16%|7/2, 11/2, ±9/2></td><td>78%|6, 13/2, ±9/2> + 14%|5, 11/2, ±9/2></td></tr>
<tr><td>1101</td><td>70%|7/2, 13/2, ±7/2> + 18%|7/2, 11/2, ±7/2></td><td>70%|6, 13/2, ±7/2> + 15%|5, 11/2, ±7/2></td></tr>
<tr><td>1464</td><td>66%|7/2, 13/2, ±5/2> + 14%|7/2, 11/2, ±5/2></td><td>66%|6, 13/2, ±5/2> + 12%|5, 11/2, ±5/2></td></tr>
<tr><td>1806</td><td>73%|7/2, 13/2, ±3/2></td><td>73%|6, 13/2, ±3/2></td></tr>
<tr><td>1912</td><td>83%|7/2, 13/2, ±1/2></td><td>83%|6, 13/2, ±1/2></td></tr>
<tr><td>2282</td><td>72% |7/2, 11/2, ±11/2> + 18% |5/2, 11/2, ±11/2></td><td>50%|5, 11/2, ±11/2> + 40%|6, 11/2, ±11/2></td></tr>
<tr><td>2544</td><td>52%|7/2, 11/2, ±9/2> + 15%|7/2, 13/2, ±9/2> + 12%|7/2, 9/2, ±9/2></td><td>39%|5, 11/2, ±9/2> + 22%|6, 11/2, ±9/2> + 15%|6, 13/2, ±9/2></td></tr>
<tr><td>2673</td><td>33%|7/2, 11/2, ±7/2> + 25%|7/2, 11/2, ±3/2> + 10%|7/2, 13/2, ±7/2></td><td>26%|5, 11/2, ±7/2> + 20%|5, 11/2, ±3/2> + 10%|6, 13/2, ±7/2></td></tr>
<tr><td>2855</td><td>52%|7/2, 11/2, ±5/2> + 15%|7/2, 13/2, ±5/2></td><td>41%|5, 11/2, ±5/2> + 15%|6, 13/2, ±5/2> + 14%|6, 11/2, ±5/2></td></tr>
<tr><td>2885</td><td>24%|7/2, 11/2, ±3/2> + 19%|7/2, 11/2, ±7/2> + 15%|7/2, 11/2, ∓ 1/2></td><td>19%|5, 11/2, ±3/2> + 15%|5, 11/2, ±7/2> + 12%|5, 11/2, ∓ 1/2></td></tr>
<tr><td>3172</td><td>55%|7/2, 11/2, ±1/2> + 20%|7/2, 11/2, ∓ 3/2></td><td>44%|5, 11/2, ±1/2> + 16%|5, 11/2, ∓ 3/2> + 15%|6, 11/2, ±1/2></td></tr>
</table>

Table S41 Electronic states for **3-Dy** obtained from a model Hamiltonian (Equation 1, main text) using projected parameters from a CASSCF-SO calculation (Table S35), and including 0.01 T field along the main anisotropy axis. Crystal field wavefunction calculated also including 0.01 T field along the main anisotropy axis, contributions ≥ 10% shown, rounded to nearest percent.

**Energy (cm $^{-1}$)**	**Wavefunction in $\vert S_{tot}, J_{tot}, m_J\rangle$ basis**	**Wavefunction in $\vert J_{4f}, J_{tot}, m_J\rangle$ basis**
0	100%|3, 8, ±8>	100%|15/2, 8, ±8>
0		
526	94%|3, 8, ±7>	94%|15/2, 8, ±7>
526		
919	73%|3, 8, ±6> + 21%|3, 8, ∓6>	73%|15/2, 8, ±6> + 21%|15/2, 8, ∓6>
919		
1215	52%|3, 8, ±5> + 43%|3, 8, ∓5>	52%|15/2, 8, ±5> + 43%|15/2, 8, ∓5>
1216		
1426	41%|3, 8, −4> + 41%|3, 8, +4>	41%|15/2, 8, −4> + 41%|15/2, 8, +4>
1447	47%|3, 8, +4> + 47%|3, 8, −4>	47%|15/2, 8, +4> + 47%|15/2, 8, −4>
1510	30%|3, 8, −3> + 30%|3, 8, +3> + 20%|3, 8, −1> + 20%|3, 8, +1>	30%|15/2, 8, −3> + 30%|15/2, 8, +3> + 20%|15/2, 8, −1> + 20%|15/2, 8, +1>
1612	30%|3, 8, −2> + 30%|3, 8, +2> + 25%|3, 8, 0>	30%|15/2, 8, −2> + 30%|15/2, 8, +2> + 25%|15/2, 8, 0>
1633	47%|3, 8, +3> + 47%|3, 8, −3>	47%|15/2, 8, +3> + 47%|15/2, 8, −3>
1898	29%|5, 3, 8, −1> + 29%|3, 8, +1> + 18%|3, 8, +3> + 18%|3, 8, −3>	29%|15/2, 8, −1> + 29%|15/2, 8, +1> + 18%|15/2, 8, +3> + 18%|15/2, 8, −3>
1905	48%|3, 8, +2> + 48%|3, 8, −2>	48%|15/2, 8, +2> + 48%|15/2, 8, −2>
2290	63%|3, 8, 0> + 13%|3, 8, −2> + 13%|3, 8, +2>	63%|15/2, 8, 0> + 13%|15/2, 8, −2> + 13%|15/2, 8, +2>
2290	44%|3, 8, +1> + 44%|3, 8, −1>	44%|15/2, 8, +1> + 44%|15/2, 8, −1>
2713	78%|3, 7, ±7> + 16%|2, 7, ±7>	48%|13/2, 7, ±7> + 46%|15/2, 7, ±7>
2713		
2905	77%|3, 7, ±6> + 15%|2, 7, ±6>	49%|13/2, 7, ±6> + 43%|15/2, 7, ±6>

<table>
<tr><td>2905</td><td></td><td></td></tr>
<tr><td>3229</td><td rowspan="2">61%|3, 7, ±5> + 14%|3, 7, ∓5> + 13%|2, 7, ±5></td><td rowspan="2">38%|13/2, 7, ±5> + 37%|15/2, 7, ±5></td></tr>
<tr><td>3229</td></tr>
<tr><td>3543</td><td rowspan="2">37%|3, 7, ±4> + 36%|3, 7, ∓4></td><td rowspan="2">24%|15/2, 7, ±4> + 23%|15/2, 7, ∓4> + 23%|13/2, 7, ±4> + 22%|13/2, 7, ∓4></td></tr>
<tr><td>3545</td></tr>
<tr><td>3782</td><td>34%|3, 7, −3> + 34%|3, 7, +3></td><td>21%|13/2, 7, −3> + 21%|15/2, 7, −3> + 21%|13/2, 7, +3> + 21%|15/2, 7, +3></td></tr>
<tr><td>3788</td><td>37%|3, 7, +3> + 37%|3, 7, −3></td><td>23%|15/2, 7, +3> + 23%|15/2, 7, −3> + 23%|13/2, 7, +3> + 22%|13/2, 7, −3></td></tr>
<tr><td>3912</td><td>31%|3, 7, −2> + 31%|3, 7, +2> + 20%|3, 7, 0></td><td>20%|13/2, 7, −2> + 20%|13/2, 7, +2> + 17%|15/2, 7, −2> + 17%|15/2, 7, +2> + 14%|13/2, 7, 0></td></tr>
<tr><td>3996</td><td>38%|3, 7, +2> + 38%|3, 7, −2></td><td>23%|15/2, 7, +2> + 23%|15/2, 7, −2> + 23%|13/2, 7, +2> + 23%|13/2, 7, −2></td></tr>
<tr><td>4028</td><td>36%|3, 7, −1> + 36%|3, 7, +1></td><td>23%|13/2, 7, −1> + 23%|13/2, 7, +1> + 19%|15/2, 7, −1> + 19%|15/2, 7, +1></td></tr>
<tr><td>4157</td><td>39%|3, 7, +1> + 39%|3, 7, −1></td><td>24%|13/2, 7, +1> + 24%|13/2, 7, −1> + 23%|15/2, 7, +1> + 23%|15/2, 7, −1></td></tr>
<tr><td>4161</td><td>64%|3, 7, 0> + 12%|2, 7, 0></td><td>41%|13/2, 7, 0> + 35%|15/2, 7, 0></td></tr>
<tr><td>5148</td><td rowspan="2">80%|2, 7, ±7> + 16%|3, 7, ±7></td><td rowspan="2">50%|15/2, 7, ±7> + 46%|13/2, 7, ±7></td></tr>
<tr><td>5148</td></tr>
</table>

Table S42 Electronic states for **3-Dy** obtained from a model Hamiltonian (Equation 1, main text) using projected parameters from a CASPT2-SO calculation (Table S36), and including 0.01 T field along the main anisotropy axis. Crystal field wavefunction calculated also including 0.01 T field along the main anisotropy axis, contributions ≥ 10% shown, rounded to nearest percent.

<table>
<tr><th>Energy (cm $^{-1}$)</th><th>Wavefunction in $|S_{tot},J_{tot},m_J\rangle$ basis</th><th>Wavefunction in $|J_{4f},J_{tot},m_J\rangle$ basis</th></tr>
<tr><td>0</td><td rowspan="2">100%|3, 8, ±8></td><td rowspan="2">100%|15/2, 8, ±8></td></tr>
<tr><td>0</td></tr>
<tr><td>631</td><td rowspan="2">88%|3, 8, ±7></td><td rowspan="2">88%|15/2, 8, ±7></td></tr>
<tr><td>631</td></tr>
<tr><td>1086</td><td>45%|3, 8, −6> + 40%|3, 8, +6></td><td rowspan="2">45%|15/2, 8, ±6> + 40%|15/2, 8, ∓6></td></tr>
<tr><td>1086</td><td>46%|3, 8, +6> + 40%|3, 8, −6></td></tr>
<tr><td>1381</td><td>42%|3, 8, −5> + 41%|3, 8, +5></td><td>42%|15/2, 8, −5> + 41%|15/2, 8, +5></td></tr>
<tr><td>1388</td><td>43%|3, 8, +5> + 42%|3, 8, −5></td><td>43%|15/2, 8, +5> + 43%|15/2, 8, −5></td></tr>
<tr><td>1625</td><td>42%|3, 8, −4> + 42%|3, 8, +4></td><td>42%|15/2, 8, −4> + 42%|15/2, 8, +4></td></tr>
<tr><td>1646</td><td>45%|3, 8, +4> + 45%|3, 8, −4></td><td>45%|15/2, 8, +4> + 45%|15/2, 8, −4></td></tr>
<tr><td>1721</td><td>30%|3, 8, −3> + 30%|3, 8, +3> + 16%|3, 8, −1> + 16%|3, 8, +1></td><td>30%|15/2, 8, −3> + 30%|15/2, 8, +3> + 16%|15/2, 8, −1> + 16%|15/2, 8, +1></td></tr>
<tr><td>1760</td><td>33%|3, 8, −2> + 33%|3, 8, +2> + 31%|3, 8, 0></td><td>33%|15/2, 8, −2> + 33%|15/2, 8, +2> + 31%|15/2, 8, 0></td></tr>
<tr><td>1834</td><td>43%|3, 8, +3> + 43%|3, 8, −3></td><td>43%|15/2, 8, +3> + 43%|15/2, 8, −3></td></tr>
<tr><td>1918</td><td>33%|5, 3, 8, −1> + 33%|3, 8, +1> + 12%|3, 8, +3> + 12%|3, 8, −3></td><td>33%|15/2, 8, −1> + 33%|15/2, 8, +1> + 12%|15/2, 8, +3> + 12%|15/2, 8, −3></td></tr>
<tr><td>1961</td><td>45%|3, 8, +2> + 45%|3, 8, −2></td><td>45%|15/2, 8, +2> + 45%|15/2, 8, −2></td></tr>
<tr><td>2363</td><td>64%|3, 8, 0> + 14%|3, 8, −2> + 14%|3, 8, +2></td><td>64%|15/2, 8, 0> + 14%|15/2, 8, −2> + 14%|15/2, 8, +2></td></tr>
<tr><td>2364</td><td>46%|3, 8, +1> + 46%|3, 8, −1></td><td>46%|15/2, 8, +1> + 46%|15/2, 8, −1></td></tr>
<tr><td>2481</td><td rowspan="2">34%|2, 7, ±7> + 33%|3, 7, ±7> + 11%|2, 7, ∓7> + 11%|3, 7, ∓7></td><td rowspan="2">55%|15/2, 7, ±7> + 18%|15/2, 7, ∓7> + 12%|13/2, 7, ±7></td></tr>
<tr><td>2481</td></tr>
<tr><td>2711</td><td>37%|3, 7, ±6> + 21%|2, 7, ±6> + 19%|3, 7, ∓6> + 11%|2, 7, ∓6></td><td>41%|15/2, 7, ±6> + 21%|15/2, 7, ∓6> + 17%|13/2, 7, ±6></td></tr>
</table>

<table>
<tr><td>2712</td><td></td><td></td></tr>
<tr><td>3035</td><td rowspan="2">28%|3, 7, ±5> + 26%|3, 7, ∓5> + 19%|2, 7, ±5> + 18%|2, 7, ∓5></td><td rowspan="2">35%|15/2, 7, ±5> + 32%|15/2, 7, ∓5> + 12%3|13/2, 7, ±5>
+ 11%|13/2, 7, ∓5></td></tr>
<tr><td>3036</td></tr>
<tr><td>3344</td><td>25%|3, 7, −4> + 25%|3, 7, +4> + 22%|2, 7, −4> + 22%|2, 7, +4></td><td rowspan="2">37%|15/2, 7, ±4> + 37%|15/2, 7, ∓4> + 10%|13/2, 7, ±4> + 10%|13/2, 7, ∓
4></td></tr>
<tr><td>3351</td><td>24%|3, 7, +4> + 24%|3, 7, −4> + 22%|2, 7, +4> + 22%|2, 7, −4></td></tr>
<tr><td>3549</td><td>24%|3, 7, −3> + 24%|3, 7, +3> + 21%|2, 7, −3> + 21%|2, 7, +3></td><td>36%|15/2, 7, −3> + 36%|15/2, 7, +3> + 10%|13/2, 7, −3> + 10%|13/2, 7, +3></td></tr>
<tr><td>3580</td><td>25%|3, 7, +3> + 25%|3, 7, −3> + 22%|2, 7, +3> + 22%|2, 7, −3></td><td>37%|15/2, 7, +3> + 37%|15/2, 7, −3> + 10%|13/2, 7, +3> + 10%|13/2, 7, −3></td></tr>
<tr><td>3716</td><td>25%|3, 7, −2> + 25%|3, 7, +2> + 21%|2, 7, −2> + 21%|2, 7, +2></td><td>36%|15/2, 7, −2> + 36%|15/2, 7, +2> + 10%|13/2, 7, −2> + 10%|13/2, 7, +2></td></tr>
<tr><td>3739</td><td>23%|3, 7, +2> + 23%|3, 7, −2> + 17%|2, 7, +2> + 17%|2, 7, −2>
+ 10%|3, 7, 0></td><td>30%|15/2, 7, +2> + 30%|15/2, 7, −2> + 12%|15/2, 7, 0> + 10%|13/2, 7, +2>
+ 10%|13/2, 7, −2></td></tr>
<tr><td>3809</td><td>29%|3, 7, −1> + 29%|3, 7, +1> + 19%|2, 7, −1> + 19%|2, 7, +1></td><td>35%|15/2, 7, −1> + 35%|15/2, 7, +1> + 13%|13/2, 7, −1> + 13%|13/2, 7, +1></td></tr>
<tr><td>3949</td><td>30%|3, 7, +1> + 30%|3, 7, −1> + 16%|2, 7, +1> + 16%|2, 7, −1></td><td>32%|15/2, 7, +1> + 32%|15/2, 7, −1> + 14%|13/2, 7, +1> + 14%|13/2, 7, −1></td></tr>
<tr><td>3959</td><td>51%|3, 7, 0> + 27%|2, 7, 0></td><td>53%|15/2, 7, 0> + 25%|13/2, 7, 0></td></tr>
<tr><td>4267</td><td rowspan="2">49%|2, 7, ±7> + 43%|3, 7, ±7></td><td rowspan="2">72%|13/2, 7, ±7> + 20%|15/2, 7, ±7></td></tr>
<tr><td>4267</td></tr>
</table>

Table S43 Percentage contributions to the singly-occupied (non-4f) molecular orbital (SOMO) from CASSCF calculations. The state-average values correspond to the percentage breakdown in the atomic natural orbital basis of the SOMO using Lödwin orthogonalization. Mulliken values are derived from the Mulliken spin populations of the ground state spin-free root of highest multiplicity, which dominates the spin-orbit ground state in both cases. Unaccounted for contributions are distributed on ligand functions.

**Ln Atomic Orbital**	**3-Tb**		**3-Dy**	
	**State-average**	**Mulliken (root 1)**	**State-average**	**Mulliken (root 1)**
**5d**	52.8	52.1	51.1	49.9
**6s**	16.0	46.0	16.6	49.1
**6d**	2.5	2.6	2.8	2.9
**7s**	5.0	0.7	4.7	0.5

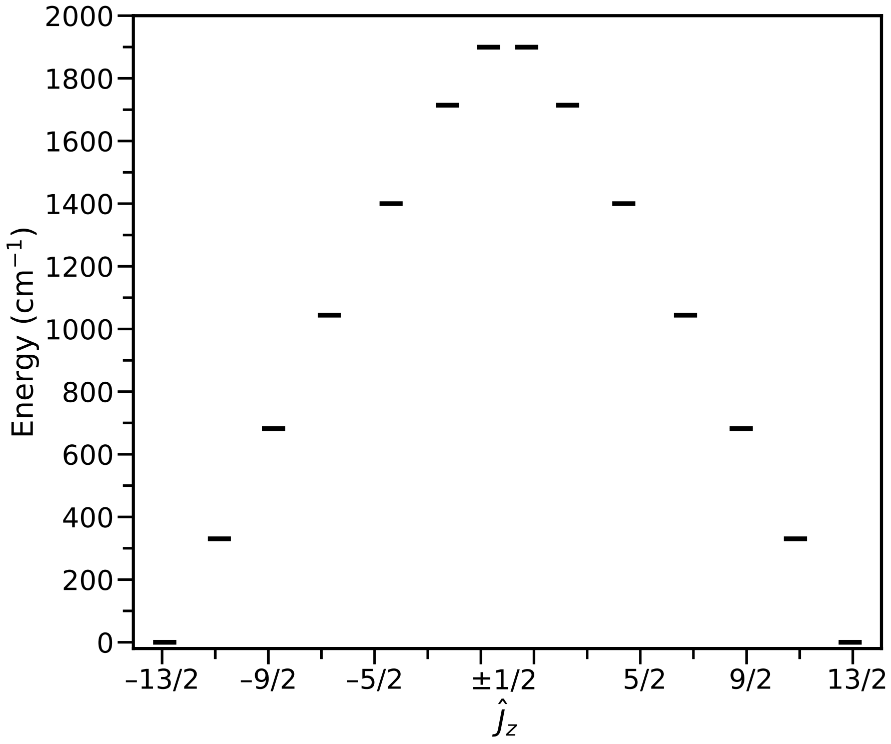

Figure S159 Energy vs. <J_z> for **3-Tb** from CASSCF-SO calculations.

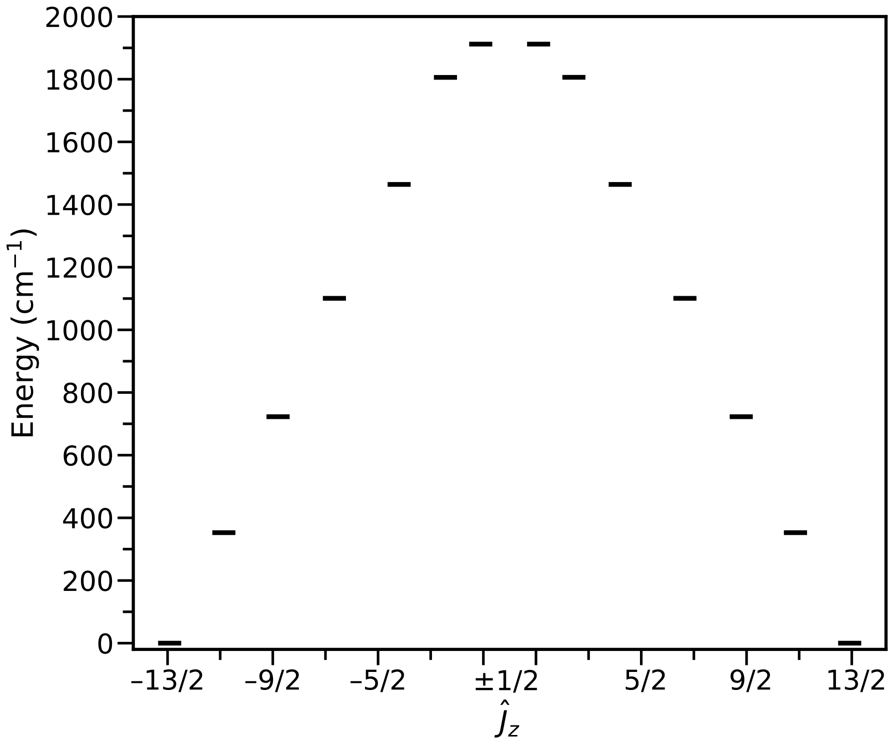

Figure S160 Energy vs. <J_z> for **3-Tb** from CASPT2-SO calculations.

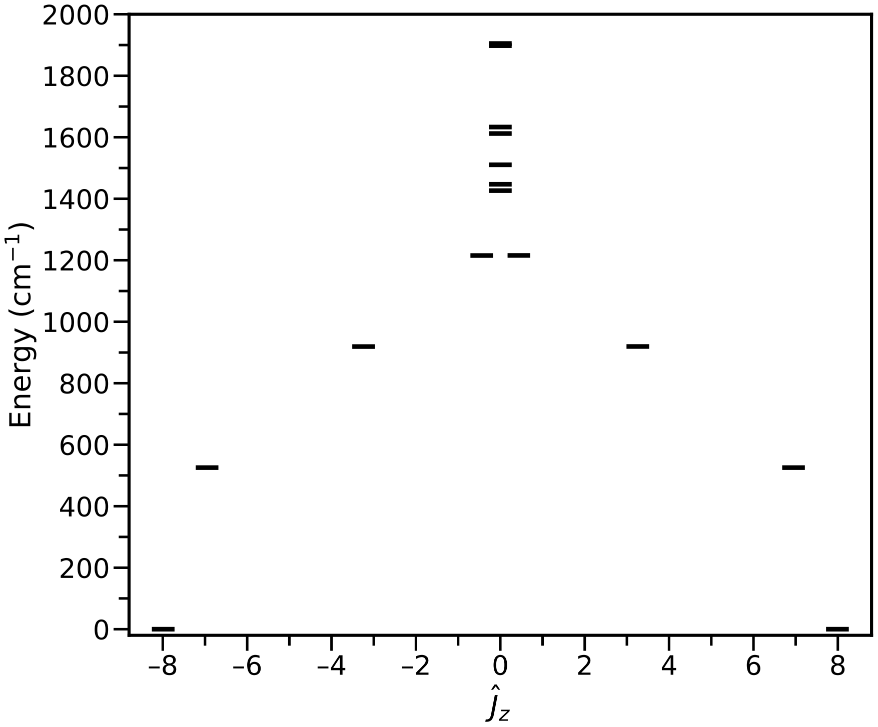

Figure S161 Energy vs. <J_z> for **3-Dy** from CASSCF-SO calculations.

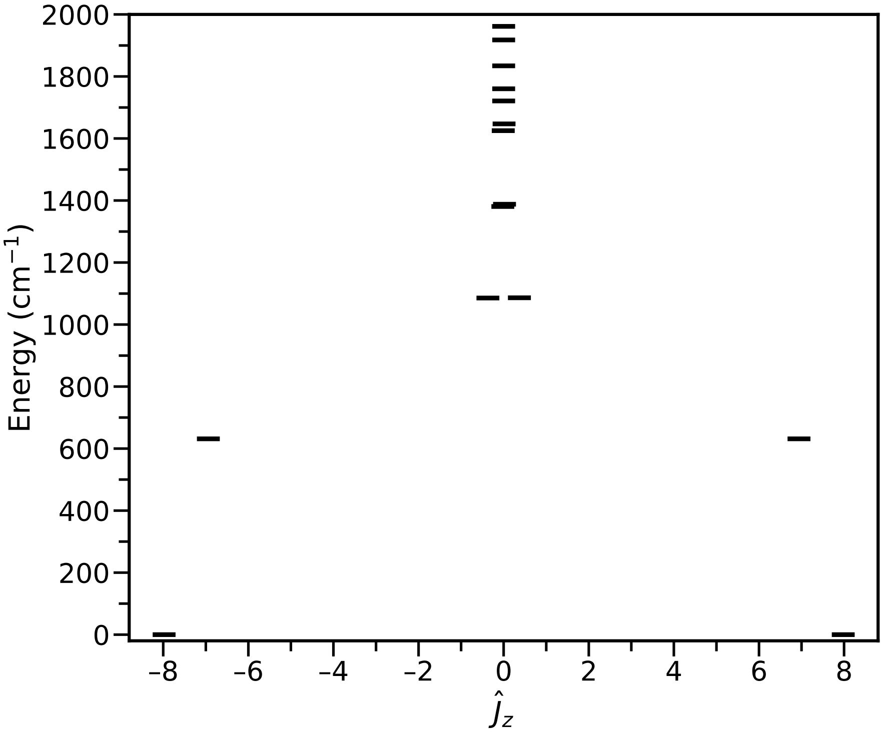

Figure S162 Energy vs. <J_z> for **3-Dy** from CASPT2-SO calculations.

9. ICP-MS data

Table S44 Selected ICP-MS data for samples of **1-Ln**, **2-Ln** and **3-Ln** digested in $HNO_{3(aq)}$. Only trace concentrations of 1 ppb or higher shown.

		**Detected concentrations**		**Trace concentrations**					
**Complex**	**Mass, mg**	**Tb, mg/L**	**Dy, mg/L**	**Tb, ppb**	**Dy, ppb**	**Tb abundance, %**	**Dy abundance, %**	**Theoretical Ln, %**	**Standard error, %**
**1-Tb**	13.5	1.165				0.17259		0.14125	22.2
**1-Dy**	10.8		0.85745				0.15879	0.14397	10.3
**2-Tb**	9.8	0.43708			1	0.08920		0.09475	–5.9
**2-Dy**	5.6		0.28611	2			0.10218	0.09668	5.7
**3-Tb**	8.4	0.64876			7	0.15447		0.15921	–3.0
**3-Dy**	9.1		0.76877				0.16896	0.16220	4.2

10. References

1. Reta, D.; Chilton, N. F. Uncertainty Estimates for Magnetic Relaxation Times and Magnetic Relaxation Parameters. *Phys. Chem. Chem. Phys.* **2019**, *21* (42), 23567–23575.

2. Pohl, I. A. M.; Westin, L. G.; Kritikos, M. Preparation, Structure, and Properties of a New Giant Manganese Oxo-Alkoxide Wheel, $[Mn_{19}O_{12}(OC_2H_4OCH_3)_{14}(HOC_2H_4OCH_3)_{10}]\cdot HOC_2H_4OCH_3$. *Chem. Eur. J.* **2001**,*7* (16), 3438–3445.

3. Gatteschi, D.; Sessoli, R. Quantum Tunneling of Magnetization and Related Phenomena in Molecular Materials. *Angew. Chem. Int. Ed.* **2003**, *42* (3), 268–297.

4. *OriginPro*; OriginLab Corporation: Northampton, England, 2022.

5. Gould, C. A.; McClain, K. R.; Yu, J. M.; Groshens, T. J.; Furche, F.; Harvey, B. G.; Long, J. R. Synthesis and Magnetism of Neutral, Linear Metallocene Complexes of Terbium(II) and Dysprosium(II). *J. Am. Chem. Soc.* **2019**, *141* (33), 12967–12973.

6. Blackmore, W. A.; Gransbury, G. K.; Evans, P.; Mills, D. P.; Chilton, N. F. Characterisation of Magnetic Relaxation on Extremely Long Timescales. *Phys. Chem. Chem. Phys.* **2023**, 25, 16735–16744.

7. Gransbury, G. K.; Corner, S. C.; Kragskow, J. G. C.; Evans, P.; Yeung, H. M.; Blackmore, W. J. A.; Whitehead, G. F. S.; Vitorica-Yrezabal, I. J.; Chilton, N. F.; Mills, D. P. *AtomAccess*: A Predictive Tool for Molecular Design and Its Application to the Targeted Synthesis of Dysprosium Single-Molecule Magnets. *ChemRxiv* **2023**, DOI:10.26434/chemrxiv-2023-28z84.

8. Zorn, R. Logarithmic Moments of Relaxation Time Distributions. *J. Chem. Phys.* **2002**, *116* (8), 3204–3209.
